# Discontinuation of First-Line Disease-Modifying Therapy in Patients With Stable Multiple Sclerosis: The DOT-MS Randomized Clinical Trial

**DOI:** 10.1001/jamaneurol.2024.4164

**Published:** 2024-12-09

**Authors:** Eline M. E. Coerver, Wing Hee Fung, Janet de Beukelaar, Willem H. Bouvy, Leo R. Canta, Oliver H. H. Gerlach, Elske Hoitsma, Erwin L. J. Hoogervorst, Brigit A. de Jong, Nynke F. Kalkers, Zoé L. E. van Kempen, Harry Lövenich, Caspar E. P. van Munster, Bob W. van Oosten, Joost Smolders, Anke Vennegoor, Esther M. P. E. Zeinstra, Mar Barrantes-Cepas, Gijs Kooij, Menno M. Schoonheim, Birgit I. Lissenberg-Witte, Charlotte E. Teunissen, Bastiaan Moraal, Frederik Barkhof, Bernard M. J. Uitdehaag, Jop Mostert, Joep Killestein, Eva M. M. Strijbis

**Affiliations:** 1 Multiple Sclerosis Center Amsterdam, Neurology, Vrije Universiteit Amsterdam, Amsterdam Neuroscience, Amsterdam University Medical Center location VUmc, Amsterdam, the Netherlands; 2 Neurology, Albert Schweitzer Hospital, Dordrecht, the Netherlands; 3 Neurology, Diakonessenhuis, Utrecht, the Netherlands; 4 Neurology, Catharina Hospital, Eindhoven, the Netherlands; 5 Neurology, Zuyderland Medical Center, Sittard-Geleen, the Netherlands; 6 School for Mental Health and Neuroscience, Maastricht University, Maastricht, the Netherlands; 7 Neurology, Alrijne Hospital, Leiden, the Netherlands; 8 Neurology, St Antonius Hospital, Utrecht, the Netherlands; 9 Neurology, OLVG, Amsterdam, the Netherlands; 10 Neurology, St Jans Gasthuis, Weert, the Netherlands; 11 Neurology, Amphia Hospital, Breda, the Netherlands; 12 MS Center ErasMS, Neurology & Immunology, Erasmus Medical Center, Rotterdam, the Netherlands; 13 Neurology, Flevoziekenhuis, Almere, the Netherlands; 14 Neurology, Isala Hospital, Meppel, the Netherlands; 15 MS Center Amsterdam, Anatomy and Neurosciences, Amsterdam Neuroscience, Amsterdam University Medical Center, location VUmc, Vrije Universiteit Amsterdam, Amsterdam, the Netherlands; 16 MS Center Amsterdam, Molecular Cell Biology and Immunology, Amsterdam Neuroscience, Amsterdam University Medical Center, location VUmc, Vrije Universiteit Amsterdam, Amsterdam, the Netherlands; 17 Department of Epidemiology and Data Science, Amsterdam University Medical Center, Vrije Universiteit Amsterdam, Amsterdam, the Netherlands; 18 Neurochemistry Laboratory, Department of Clinical Chemistry, Amsterdam Neuroscience, Amsterdam University Medical Center, Vrije Universiteit Amsterdam, Amsterdam, the Netherlands; 19 MS Center Amsterdam, Radiology & Nuclear Medicine, Amsterdam Neuroscience, Amsterdam University Medical Center location VUmc, Vrije Universiteit Amsterdam, Amsterdam, the Netherlands; 20 Queen Square Institute of Neurology and Centre for Medical Image Computing, University College London, London, United Kingdom.; 21 Neurology, Rijnstate Hospital, Arnhem, the Netherlands

## Abstract

**Importance:**

Increasing numbers of people with multiple sclerosis (MS) use disease-modifying therapy (DMT). Long-term stable disease while taking such medications provides a rationale for considering DMT discontinuation given patient burden, costs, and potential adverse effects of immunomodulating therapy.

**Objective:**

To investigate whether first-line DMT can be safely discontinued in patients with long-term stable MS.

**Design, Setting, and Participants:**

This multicenter, rater-blinded, noninferiority randomized clinical trial was conducted between July 1, 2020, and March 20, 2023, at 14 Dutch centers. Data analysis was performed between July 2023 and January 2024. Key inclusion criteria were relapse-onset MS, aged 18 years or older, without relapses, and without substantial magnetic resonance imaging (MRI) activity in the previous 5 years under first-line DMT. Participants were randomized 1:1 to discontinue or continue first-line DMT.

**Intervention:**

Discontinuation of first-line DMT.

**Main Outcome and Measure:**

The primary outcome was significant inflammatory disease activity, defined as relapse and/or 3 or more new T2 lesions or 2 or more contrast-enhancing lesions on brain MRI.

**Results:**

Of 163 potentially eligible participants, 89 participants were included in the trial at the moment of early termination. Forty-four participants (49.4%) were assigned to the continue group and 45 participants (50.6%) were assigned to the discontinue group. Median (IQR) age was 54.0 (49.0-59.0) years, and 60 participants (67.4%) were female. Two participants in the continue group were lost to follow-up. After a median (IQR) follow-up time of 15.3 (11.4-23.9) months, the trial was prematurely terminated because of inflammatory disease activity recurrence above the predefined limit. In total, 8 of 45 participants in the discontinue group (17.8%) vs 0 of 44 participants in the continue group reached the primary end point and had recurrent, mostly radiological inflammation. Two of these 8 participants had a clinical relapse. Median (IQR) time to disease activity was 12.0 (6.0-12.0) months.

**Conclusions and Relevance:**

In this randomized clinical trial, even in patients with long-term MS stable for over 5 years, first-line DMT discontinuation can lead to recurrence of inflammatory disease activity. Although this study cohort was relatively small, the recurrence of inflammation in the discontinue group was significantly higher than in the continue group and also higher than in the previously published DISCOMS trial, which only included individuals aged 55 years or older. This study provides additional data, especially in a younger population and including longitudinal biomarker measurements, for informed decision-making in cases when treatment discontinuation is considered.

**Trial Registration:**

ClinicalTrials.gov Identifier: NCT04260711

## Key Points

**Question:**

Can first-line disease-modifying therapy (DMT) be safely discontinued in patients with long-term stable multiple sclerosis (MS) aged 18 years and older?

**Findings:**

In this multicenter, rater-blinded, noninferiority randomized clinical trial that included 89 participants with relapse-onset MS, 8 of 45 participants in the discontinue group (17.8%) vs 0 of 44 participants in the continue group had recurrent, mostly radiological inflammation. The trial was prematurely terminated because of inflammatory disease activity recurrence above the predefined limit.

**Meaning:**

First-line DMT discontinuation can lead to recurrence of inflammatory disease activity in approximately 20% of cases, even in patients with long-term stable MS.

## Teaser

This randomized clinical trial investigates whether first-line disease-modifying therapy can be safely discontinued in patients with long-term stable multiple sclerosis.

## Introduction

An increasing number of people with stable multiple sclerosis (MS) are treated with disease-modifying therapy (DMT). The aim of starting DMT in people with relapse-onset MS is to prevent focal inflammatory disease activity, detected by relapses and new or enhancing lesions on magnetic resonance imaging (MRI) examination of the brain and spinal cord,^1^ to achieve a status of complete clinical and radiological control of inflammatory events and a lower risk of future disability. With increasing sensitivity of the diagnostic criteria for MS, therapy is initiated at earlier disease stages, sometimes even before a definitive diagnosis is made or when demyelination is asymptomatic, such as in radiologically isolated syndrome.^2^ While this approach aims to achieve early control of disease, it inherently carries the risk of overtreatment in patients. On the other hand, evidence suggests that the risk-benefit profile of anti-inflammatory therapy changes over the disease course. As individuals age, the risk of relapses and inflammatory activity on MRI decreases, leading to a lower relative efficacy of therapy, while immunosenescence may increase the risk of adverse effects.^3,4,5^ Taken together, there is an increasing need to know if and when treatment can potentially be discontinued.

Multiple observational studies have investigated the risk of DMT discontinuation and possible predictors for the recurrence of focal inflammatory disease activity after discontinuation. Higher age and longer duration of stable disease have been associated with a lower risk of inflammatory disease activity after discontinuation.^6,7,8,9,10,11^ However, very limited evidence is available on DMT discontinuation from randomized clinical trials. The DISCOMS (Discontinuation of Disease Modifying Therapies in Multiple Sclerosis) trial, a 2023 randomized clinical trial investigating DMT discontinuation in participants with stable disease older than 55 years, observed very limited disease reactivation after DMT discontinuation, but could not determine noninferiority of DMT discontinuation compared to continuation in this study population.^12^ Another randomized clinical trial (STOP-I-SEP [NCT03653273]) conducted in people with secondary progressive MS aged 50 years and older is presently underway.

Here, we report the results of the DOT-MS trial (NCT04260711), a randomized clinical trial of DMT discontinuation in patients with relapse-onset MS older than 18 years with stable inflammatory MS over the past 5 years. The primary objective was to identify whether DMT discontinuation is safe to consider for adult patients with MS that have been stable for many years.

## Methods

### Study Design

The DOT-MS trial was an investigator-initiated, multicenter clinical trial with randomized treatment group assignments, open-label treatment, and masked end point evaluation. DMT continuation was compared to discontinuation in participants with long-term stable relapse-onset MS. The original planned duration of the trial was 3.5 years, from March 1, 2020, to January 1, 2024. Data analysis was performed between July 2023 and January 2024. The study protocol can be found in Supplement 1.

### Participants

The DOT-MS trial was conducted at 14 centers in the Netherlands. Participants were aged 18 years or older with relapse-onset MS (relapsing-remitting MS or secondary progressive MS), used first-line DMT, and had neither clinical relapses nor substantial radiological disease activity (defined as no new contrast-enhancing lesions and 1 or more new T2 lesions on MRI) for at least 5 years before inclusion.^13^ In case the last available MRI scan was conducted 10 or more years ago, no more than 3 new T2 lesions suggestive of demyelination in the last 10 years were accepted. New lesions detected across different MRI scans were cumulative. Methodological details of the inclusion and exclusion criteria are given in eMethods 1 and 2 in Supplement 2.

All participants provided written informed consent, and data usage was approved by the medical ethics committee of the Amsterdam University Medical Center, location VUmc (Protocol identifier, NL71260.029.19; ClinicalTrials.gov identifier, NCT04260711).

### Randomization

After inclusion, participants were randomly assigned 1:1 to either continue or discontinue their current DMT using the randomization procedure with block sizes of 4 and 6 in Castor version 2023.4.1.3. Age (categorized as <55 years or ≥55 years) was used as a stratification factor to exclude potential bias.

### Procedures, Visit Schedule, and Masking

The brain MRI scans from previous years were reviewed by the local principal investigator and radiologist to confirm stable disease. In addition, a screening brain MRI, obtained within a year before enrollment, was compared with the last available brain MRI and reviewed by the local principal investigator and radiologist to confirm absence of substantial change (≤1 new T2 lesion on MRI for at least 5 years before inclusion or ≤3 new T2 lesions suggestive of demyelination in the last 10 years).

Participants underwent clinical evaluations and standardized protocol brain MRI scans with gadolinium at baseline and at months 3, 6, 12, 18, and 24. Unscheduled visits were arranged if deemed necessary by the physician. Participants had Expanded Disability Status Scale (EDSS) and Multiple Sclerosis Functional Composite (MSFC) examination by a masked rater. MSFC examination included the oral version of the Symbol Digit Modalities Test (SDMT), the Timed 25-Foot Walk (T25-FW), and the Nine-Hole Peg Test (9-HPT). Scans at site were evaluated by experienced, local board-certified neuroradiologists who were masked for treatment allocation, and scans were centrally reviewed and validated by the main investigation center (Amsterdam UMC, location VUmc) for ambiguous cases or in case of scans that showed any MRI activity. Spinal cord imaging was limited to suspected spinal cord–related relapses. Participants completed patient-reported outcome questionnaires at all visits, including the 29-item Multiple Sclerosis Impact Scale (MSIS-29),^14^ the Checklist Individual Strength (CIS20r),^15^ and the 36-item Health Status Questionnaire (SF-36),^16^ and responded to the question “How satisfied are you with your present DMT or lack of DMT?” on a 7-point Likert scale. Blood was collected for biobank storage and analysis, and adverse events were documented at each visit.

### Outcomes

The primary outcome measure of the DOT-MS trial was significant disease activity during follow-up, defined as any confirmed relapse and/or significant MRI activity. Relapses were defined according to the definition most often used in MS phase 3 trials (see eMethods 3 in Supplement 2). Relapses were also presented and discussed with the data safety monitoring board (DSMB). Significant MRI activity was defined as 3 or more new T2 lesions or 2 or more contrast-enhancing lesions on brain MRI results. New lesions detected across different MRI scans during the trial were considered cumulative in meeting the criteria for significant MRI activity.^17,18,19^

Secondary outcome measures included any MRI activity (here also including 1 or 2 new T2 lesions 1 contrast-enhancing lesion, or enlarged T2 lesions), significant confirmed disability progression, and change on the patient-reported outcome measures. See eMethods 4 in Supplement 2 for specific definitions. Additionally, neurofilament light (NfL) and glial fibrillary acidic protein (GFAP) levels were assessed in serum using a single-molecule array assay on a HD-X analyzer (Quanterix) as described in detail elsewhere.^20,21,22^

### Interim Analysis and Premature Termination of the Trial

An independent DSMB was appointed to assess the safety of the discontinuation of therapy during the trial. During the predefined interim analyses, the DSMB could recommend to terminate the trial if there were more participants with significant disease activity in the discontinue group than in the continue group and if the 95% confidence interval of the difference in the proportion of participants with significant disease activity between the 2 groups did not include 0. Exact binomial methods were used to test the primary end point and calculate 95% confidence intervals.

### Statistical Analysis

The primary statistical analysis was a noninferiority test with a margin of 7.5% for the proportions with significant disease activity in the continue and discontinue groups. This noninferiority margin was set based on the balance of statistical power, clinical relevance, and feasibility. It was estimated that the proportion of stable participants in the continue group would be around 97.5% of participants. A preliminary power calculation based on the noninferiority principle was performed using PASS version 12 (NCSS) (1-sided *z* test [unpooled]; significance level, .05) and showed a necessary sample size of 54 participants per group to achieve 80% power. The total sample size was set at 130 participants, taking a 20% dropout rate into account. Statistical significance was set as *P* < .05 with 2-tailed tests.

The longitudinal changes of NfL and GFAP levels were analyzed using a linear mixed-effects model. The change of NfL and GFAP levels for participants with confirmed disability progression were analyzed using 1-sample *t* test. To evaluate the magnitude of NfL level elevations, participants who did not develop significant disease activity and any MRI activity were used to calculate a 95th percentile threshold for percentage change from baseline.^23^ See eMethods 5 in Supplement 2 for the analysis of other secondary end points.

The conditional power was calculated post hoc, which is the probability of rejecting the null hypothesis that discontinuation is inferior to continuation under the assumption that the trial continued with the same event rates observed at the time of analysis.^24^ To account for the variation in total follow-up duration between the continue and discontinue groups, the incidence rate of disease activity was calculated by dividing the number of events by the total follow-up duration for each group. Poisson regression analysis was used to compare the incidence rates of a disease event in the continue and discontinue group. These analyses also included relapse and significant MRI activity separately and participants with any MRI activity.

Latest available data were used if an end-of-study (EoS) visit was absent during analysis due to delayed scheduling. Results for primary, secondary, and post hoc outcomes were not corrected for multiple testing. Analyses were performed in the intention-to-treat population using R version 4.2.1 (R Foundation).

## Results

**Figure 1.**
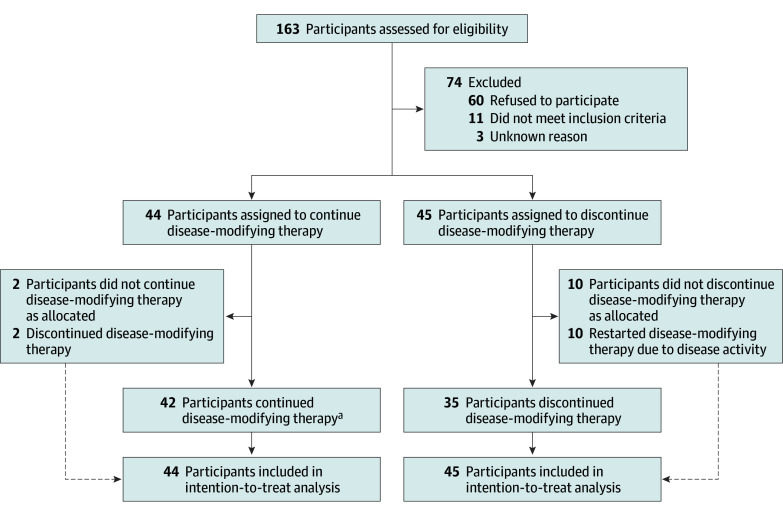
Study Profile ^a^Includes 2 participants who were lost to follow-up. In addition to the scheduled visits and procedures, there were 17 unscheduled visits: 7 in the continue group due to new complaints and 10 in the discontinue group due to 3 with new complaints, 1 on request, and 6 for follow-up after disease activity.

Between July 1, 2020, and March 20, 2023, 44 participants (49.4%) were randomized to the continue group and 45 (50.6%) to the discontinue group (Figure 1). Participants were predominately female (60 of 89 participants [67.4%]), and 80 participants (89.9%) had relapsing-remitting MS (Table 1). The median (IQR) age at enrollment was 54.0 years (49.0-59.0). The median (IQR) time since the last documented relapse was 9.4 years (6.9-13.2). There were 58 participants (65.1%) receiving injectable drugs (glatiramer acetate or interferon beta). The characteristics of the participants in the continue and discontinue group did not differ at baseline (Table 1). The trial was prematurely terminated on March 20, 2023, because the recurrence of significant disease activity in the discontinue group exceeded predefined limits. A post hoc conditional power analysis showed that the probability of rejecting the null hypothesis that discontinuation was inferior to continuation was <0.001 in the case of hypothetical continuation of the trial (Table 2), also in the case of using any MRI activity as criterion. Since the 2-year follow-up was not completed, and taking the variation of total follow-up duration between the continue and discontinue groups into account, the incidence rates of significant disease activity and any MRI activity were calculated (shown in eTable 1 in Supplement 2). The planned noninferiority analyses could not be performed, as the trial was terminated early, so the primary outcome was not tested for the noninferiority analysis.

**Table 1.** Baseline Characteristics

Characteristic	Participants, No. (%)	
	Continuation (n = 44)	Discontinuation (n = 45)
Age, median (IQR), y	55.0 (50.0-59.0)	54.0 (47.0-58.0)
Sex		
Female	28 (63.6)	32 (71.1)
Male	16 (36.4)	13 (28.9)
Time since symptom onset, median (IQR), y	13.3 (9.9-22.2)	14.1 (9.4-19.6)
Time since last documented relapse, median (IQR), y	9.8 (6.8-13.3)	9.4 (7.1-12.3)
Multiple sclerosis subtype		
Relapsing-remitting	39 (88.6)	41 (91.1)
Secondary progressive	5 (11.4)	4 (8.9)
Total duration of disease-modifying therapy use, median (IQR), y	11.4 (7.7-17.9)	11.1 (7.8-13.8)
Disease-modifying therapy at randomization		
Interferon beta	18 (40.9)	17 (37.8)
Glatiramer acetate	11 (25.0)	12 (26.7)
Teriflunomide	8 (18.2)	4 (8.9)
Dimethyl fumarate	7 (15.9)	12 (26.7)
Expanded Disability Status Scale score, mean (SD)	3.1 (1.6)	3.1 (2.0)
Symbol Digit Modalities Test score, mean (SD)^a^	51.6 (11.4)	51.7 (13.3)
Timed 25-Foot Walk test score, mean (SD)^b^	5.4 (1.3)	5.2 (1.0)
Nine-Hole Peg Test, mean (SD)	23.2 (6.1)	23.5 (7.8)
Neurofilament light chain, mean (SD), pg/mL	12.0 (5.9)	11.0 (5.0)
Glial fibrillary acidic protein, mean (SD), pg/mL	87.3 (47.0)	83.8 (34.5)

^a^ Baseline Symbol Digit Modalities Test score was missing for 1 participant in the continue group.

^b^ Baseline Timed 25-Foot Walk score was missing for 2 participants in the discontinue group.

**Table 2.** Number of Participants With an Inflammatory Event

Event	No./total No. (%)		Conditional power
	Continuation	Discontinuation	
Primary outcome event (relapse or significant MRI activity)	0/44 (0)	8/45 (17.8)	<.001
Relapse	0/44 (0)	2/45 (4.4)	NA
Significant MRI activity^a^	0/44 (0)	7/45 (15.6)	NA
Any MRI activity^b^	1/44 (2.3)	11/45 (24.4)	<.001

Abbreviations: MRI, magnetic resonance imaging; NA, not applicable.

^a^ Significant MRI activity was defined as ≥3 new T2 lesions or ≥2 contrast-enhancing lesions.

^b^ Any MRI activity included participants with 1 or 2 new T2 lesions, 1 contrast-enhancing lesion, or enlarged T2 lesions, in addition to the participants that met the significant disease activity criteria.

An EoS visit after premature termination was completed by 86 of 89 total participants (96.6%). Two participants in the continue group were lost to follow-up, and 1 participant in the continue group declined the EoS visit. The results were analyzed from baseline until EoS.

The median (IQR) follow-up time at trial discontinuation was 15.3 months (11.4-23.9; eTable 2 in the Supplement). In the discontinue group, 8 of 45 participants (17.8%) had significant disease activity, compared with 0 of 44 participants (0%) in the continue group (Table 2). The difference in the proportion of participants with significant disease activity between the discontinue and continue groups did not include 0 (95% CI, 0.09-0.32). Of the 8 primary outcome events, 6 of 45 participants (13.3%) had 3 or more new T2 lesions or 2 or more contrast-enhancing lesions without relapse, and 2 of 45 participants (4.4%) had a relapse with inflammatory disease activity on MRI examination (eTable 3 in Supplement 2). No participants experienced clinical relapse without inflammatory MRI activity. Among those who had significant disease activity, the median (IQR) time to reaching the primary end point was 12.0 months (6.0-12.0). Participants with significant disease activity had a median (IQR) age of 46.0 years (43.5-58.5), while those without significant disease activity had a median (IQR) age of 54.0 years (50.0-59.0), but the difference was not significant (*P* = .19). Baseline demographic characteristics among those who did or did not meet the primary outcome measures were similar (eTable 4 in Supplement 2). Case descriptions for participants with disease activity are given in eTable 5 in Supplement 2.

In our analysis of any MRI activity, 11 of 45 participants in the discontinue group (24.4%) and 1 of 44 participants in the continue group (2.3%) had any MRI activity. This indicated that 4 additional participants with MRI activity did not meet the criteria for significant disease activity and consequently did not reach the primary study end point. All 4 participants had 1 new contrast-enhancing lesion on MRI examination (1 in the continue group and 3 in the discontinue group; eTable 3 in Supplement 2). There were no significant differences at baseline between participants with and without any MRI activity (eTable 6 in Supplement 2).

Participants had the opportunity to restart DMT during the trial. Until the end of the study, 35 of 45 participants in the discontinue group (77.8%) remained off treatment. Among those with any MRI activity in the discontinue group, only 1 participant did not restart DMT. Within a 6-month follow-up, 10 of 12 participants with any MRI activity were clinically and MRI stable. Of note, 2 had persistent disease activity on MRI despite restart of DMT.

**Figure 2.**
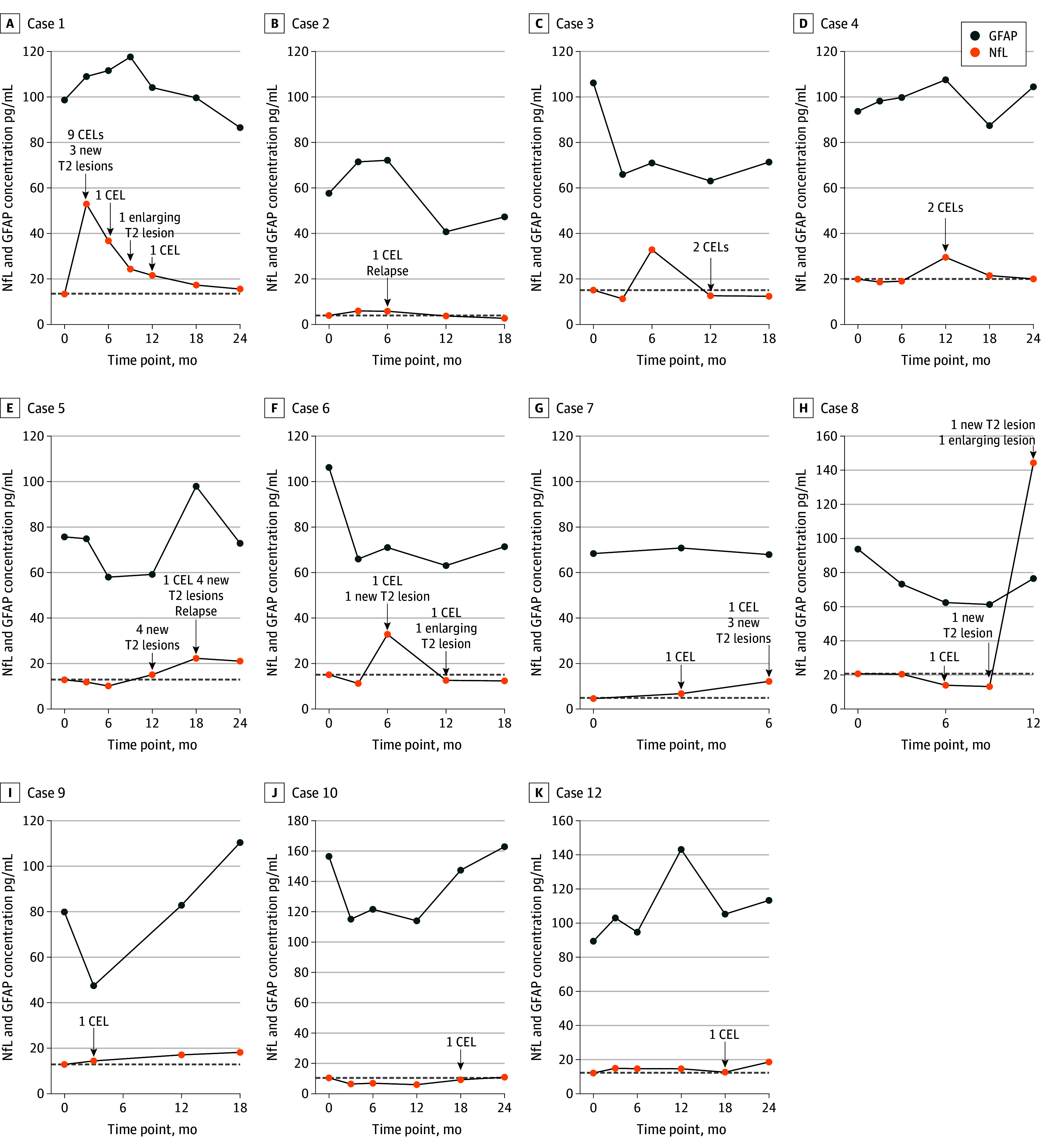
Serum Neurofilament Light (NfL) and Glial Fibrillary Acidic Protein (GFAP) Levels of Participants With Significant Disease Activity and Any Magnetic Resonance Imaging Activity Cases 1-8 (A-H) are participants (all in the discontinue group) with significant disease activity (relapse and/or ≥3 new T2 lesions or ≥2 contrast-enhancing lesions [CELs]). Cases 9-12 (I-K) are participants with MRI activity (≤2 new T2 lesions, 1 contrast-enhancing lesion, or enlarged T2 lesions) other than the criteria for significant MRI activity. Cases 9 and 10 (I and J) are in the discontinue group and case 12 is (K) in the continue group. Case 11 is not presented, as the blood sample during disease activity was not collected. The gray dashed line represents the baseline NfL level per case.

Baseline NfL and GFAP levels did not differ between the continue and discontinue groups (*P* = .59 and *P* = .90, respectively) or between participants that remained stable and those that developed significant disease activity (*P* = .42 and *P* = .20, respectively). Figure 2 illustrates the longitudinal course of NfL and GFAP levels of participants with significant disease activity and any MRI activity. Longitudinally, linear mixed-effects models showed that overall absolute NfL levels were not different between the continue and discontinue groups (β, 1.23; 95% CI, −1.50 to 3.92; *P* = .37; eTable 7 in Supplement 2), but in participants with significant disease activity, longitudinal NfL levels were higher compared to participants without significant disease activity (β, 6.92; 95% CI, 2.55-11.29; *P* = .003; eTable 8 in Supplement 2). This was more pronounced when using a 3-month time frame around specific visits with significant disease activity (β, 9.11; 95% CI, 5.0-13.34; *P* < .001; eTable 9 in Supplement 2). The 95th percentile threshold of percentage change from baseline was 46.47% for significant MRI activity. Only 1 of 8 participants with significant disease activity exceeded this. Similar results were found for the participants with any MRI activity compared to those without (eTables 10-11 in Supplement 2). For any MRI activity, the 95th percentile threshold of percentage change from baseline was 45.85%. Only 1 of 12 participants exceeded the 95th percentile threshold for percentage change from baseline. Longitudinal GFAP levels showed no differences between the continue and discontinue groups or between participants with and without significant disease activity or any MRI activity (eTables 12-16 in Supplement 2).

The median EDSS, 9-HPT, and T25-FW scores and mean SDMT score (eTables 17-22 in Supplement 2) were not different between the continue and discontinue groups at baseline and EoS. There were no differences between groups in EDSS-based confirmed disability progression (eTable 18 in Supplement 2). There were no associations between biomarker levels and clinical disability (eTable 19 in Supplement 2).

Median MSIS-29, CIS20r, and SF-36 scores, treatment satisfaction, and the proportions of significant changes herein were not different between the continue and discontinue groups (eTables 23-29 in Supplement 2). Regarding treatment satisfaction, all participants with significant MRI activity remained satisfied with the discontinuation of DMT by EoS. Among those with any MRI activity, 2 participants were dissatisfied with the absence of DMT. One participant with any MRI activity shifted from dissatisfaction to satisfaction by the study’s conclusion.

The numbers of adverse events and serious adverse events were comparable between groups (Table 3). In the continue group, 3 serious adverse events were reported: 1 liver abscess due to diverticulitis and 2 myocardial infarctions, all unrelated to the study.

**Table 3.** Adverse Events

Event	Continue group (n = 44)		Discontinue group (n = 45)	
	No. of events	No. of participants (%)	No. of events	No. of participants (%)
Overall				
Adverse events				
Mild (grade 1)	26	11 (25.0)	17	12 (26.7)
Moderate (grade 2)	7	5 (11.4)	8	7 (15.6)
Severe (grade ≥3)	4	3 (6.8)	4	2 (4.4)
Serious adverse events^a^	3	3 (6.8)	0	0
Common adverse events or treatment-related adverse events^b^				
COVID-19	5	4 (9.1)	6	6 (13.3)
Influenza	1	1 (2.3)	3	3 (6.7)
Fatigue	3	2 (4.5)	0	0
Fall	2	2 (4.5)	1	1 (2.2)
Treatment adverse effects	1	1 (2.3)	0	0
Abnormal white blood cell count	1	1 (2.3)	0	0

^a^ No serious adverse events were deemed to be treatment related.

^b^ Adverse events occurring in 3% of participants or more. Any adverse events related to treatment are reported, even if they did not exceed the 3% threshold.

## Discussion

The DOT-MS trial was terminated early due to a significantly increased risk of inflammatory disease activity after discontinuing first-line DMT, surpassing the predefined safety limit. A post hoc conditional power analysis showed that the probability of disproving the null hypothesis (that discontinuation is inferior to continuation) was <0.001 if the trial had continued. In this study, 8 of 45 participants who discontinued DMT (17.8%) had significant inflammatory disease activity compared to no participants in the continuation group. This suggests a higher risk of active disease after first-line DMT discontinuation in people with relapse-onset MS with a median (IQR) age of 46.0 (43.5-58.5) years, despite being stable for at least 5 years prior to stopping DMT.

Age has consistently been shown to predict inflammatory disease activity, with older age linked to a natural decline.^25,26,27,28,29,30^ Observational studies have shown that DMT discontinuation after age 45 years is generally associated with stable disease, while younger patients tend to relapse or develop MRI disease activity. In this trial, participants with significant disease activity were generally younger (median [IQR] age 46.0 [43.5-58.5] vs 54.0 [50.0-59.0] years), although 5 of 45 participants with significant disease activity (11.1%) were 45 years or older, including some aged over 55 years. This suggests that even older patients face some risk of recurrence after discontinuation. The risk in younger patients, which was unknown prior to the DOT-MS trial, was significantly higher compared to those continuing DMT in the previously published DISCOMS trial that only included individuals aged 55 years or older.

The DISCOMS trial (focusing on participants aged ≥55 years) reported a 12.2% recurrence rate (defined as new relapse, new or enlarged T2 lesions, or both) after DMT discontinuation, compared to 24.4% (11 of 45 participants) in the younger DOT-MS population using the criterium of any disease activity.^12^ Additionally, the median time to disease recurrence was shorter in the DOT-MS trial than the DISCOMS trial (6.0 months vs 16.3 months, respectively). Using this study’s criteria of significant disease activity, 7 of 131 participants in the discontinue group (5.3%) and 1 of 128 participants in the continue group (0.8%) in the DISCOMS trial would have had significant disease activity. A major difference between the trials, by design, lies in the study populations—DISCOMS included older participants (median age 63.0 vs 54.0 years), with a longer duration since last relapse (13.9 vs 9.4 years). MRI protocols also varied, especially with regard to contrast enhancement, potentially leading to a (slight) underestimation of disease activity in DISCOMS. However, in the DOT-MS trial there were no participants with contrast-enhancing lesions without any new lesions on T2/fluid-attenuated inversion recovery images.

A key observation in both trials is the low occurrence of clinical relapses after discontinuation, with disease activity mostly detected through routine MRI scans. Upon resuming DMT, most participants became clinically stable within 6 months. Ultimately, the decision to stop therapy should be individualized, and age and disease stability are critical factors. In addition to the DISCOMS and DOT-MS data, real-world data and the VIAADISC risk score suggest minimal risk of disease reactivation in patients aged 55 to 60 years and older, especially those with long-term disease stability (≥8 years).^11,31^ However, discontinuation of therapies like natalizumab or S1P modulators remains risky due to potential rebound activity.

Serum biomarkers reveal subclinical disease activity through distinct mechanisms, with NfL reflecting neuroaxonal injury and GFAP reflecting astrocyte activation.^32,33^ In the DOT-MS trial, serum biomarkers (NfL and GFAP) did not predict subsequent disease activity, which is in line with a previous study that investigated the temporal relationship between NfL and new disease activity.^23^ However, NfL levels did increase during and sometimes beyond episodes of significant disease activity. For example, in case 3 (Figure 2), NfL increased before contrast-enhancing lesions at 6 months. In addition, case 5 had persistent increased NfL level at 18 months. Seven of 8 participants with significant disease activity had an increase in their NfL levels during disease activity, while that increase was less profound in participants with any MRI activity (cases 9, 10, and 12 in Figure 2).

### Limitations

Despite the clarity of the primary outcome and confirmatory secondary outcome measures, including longitudinal NfL measures, the premature trial termination imposed some limitations. Planned analyses, including the noninferiority test and cost analysis, could not be performed as intended due to an insufficient sample size and incomplete follow-up. Due to the small number of events, no further subgroup analyses were performed. Nonetheless, a conditional power analysis indicated less than 0.001 probability of rejecting the null hypothesis.^24^ While follow-up time varied, this was accounted for in the incidence rate analysis. Also, spinal MRI scans were not routinely performed in this trial according to the current MAGNIMS guidelines, which advise against the use of routine spinal cord MRIs and recommend them only in a few special circumstances.^19^ Lastly, not all MRI scans were centrally reread, which could potentially lead to an underestimation of reported disease activity on MRI, even though the evaluation was performed by trained neuroradiologists at the study sites.

The DOT-MS trial will continue in an observational design. Participants who discontinued their DMT were offered to restart and will be followed-up for 2 years. Long-term consequences of DMT discontinuation and time to status of no evident disease activity will be studied.

## Conclusions

In conclusion, this study shows that first-line DMT discontinuation led to disease recurrence (mainly radiological) in a significant proportion of participants with relapse-onset MS, even in those who were inflammatory stable for 5 or more years. However, over 75% of participants had no disease recurrence after DMT discontinuation. We believe that an attempt to discontinue first-line DMT in long-term stable patients with MS is still a viable option, but close clinical, radiological, and perhaps biomarker-based monitoring is mandatory. Study data, in addition to the DISCOMS data, enable informed decision-making in cases where treatment discontinuation is considered.

## Notes

Supplement 1. Trial Protocol

Supplement 2. **eMethods 1.** Inclusion Criteria **eMethods 2.** Exclusion Criteria **eMethods 3.** Definition of Relapse **eMethods 4.** Secondary End Points **eMethods 5.** Statistical Analysis of Secondary End Points **eTable 1.** Incidence Rates of Disease Activity **eTable 2.** Length of Follow-Up **eTable 3.** Participants With Relapse and/or MRI Activity **eTable 4.** Baseline Characteristics of Participants With and Without "Significant" Disease Activity **eTable 5.** Case Descriptions of Participants With “Significant” Disease Activity or “Any” MRI Activity **eTable 6.** Baseline Characteristics of Participants With and Without "Any" MRI Activity **eTable 7.** Linear Mixed-Effects Model Regarding Absolute Serum NfL Level in the Continue and Discontinue Group **eTable 8.** Linear Mixed-Effects Model Regarding Absolute Serum NfL Level in “Significant" Disease Activity at Participant Level **eTable 9.** Linear Mixed-Effects Model Regarding Absolute Serum NfL Level in "Significant" Disease Activity at Visit Level **eTable 10.** Linear Mixed-Effects Model Regarding Absolute Serum NfL Level in "Any" MRI Activity at Participant Level **eTable 11.** Linear Mixed-Effects Model Regarding Absolute Serum NfL Level in "Any" MRI Activity Disease Activity at Visit Level **eTable 12.** Linear Mixed-Effects Model Regarding Absolute Serum GFAP Level in the Continue and Discontinue Group **eTable 13.** Linear Mixed-Effects Model Regarding Absolute Serum GFAP Level in "Significant" Disease Activity at Participant Level **eTable 14.** Linear Mixed-Effects Model Regarding Absolute Serum GFAP Level in "Significant" Disease Activity at Visit Level **eTable 15.** Linear Mixed-Effects Model Regarding Absolute Serum GFAP Level in “Any” MRI Activity at Participant Level **eTable 16.** Linear Mixed-Effects Model Regarding Absolute Serum GFAP Level in “Any” MRI Activity Disease Activity Visit Level **eTable 17.** Median (IQR) Expanded Disability Status Scale (EDSS) Change by Group **eTable 18.** Proportions of Significant Confirmed Disability Progression **eTable 19.** Mean Percentage (SD) Change of NfL and GFAP Levels of Participants With Confirmed Disability Progression **eTable 20.** Mean (SD) Symbol Digit Modalities Test (SDMT) Change by Group **eTable 21.** Median (IQR) Timed 25-Foot Walk (T25-FW) Change by Group **eTable 22.** Median (IQR) 9-Hole Peg Test (9-HPT) Change by Group **eTable 23.** Median (IQR) MS Impact Scales (MSIS-29) by Group **eTable 24.** Significant Change in MS Impact Scales (MSIS-29) by Group **eTable 25.** Median (IQR) Checklist Individual Strength (CIS20R) by Group **eTable 26.** Significant Change in Fatigue Severity in Checklist Individual Strength (CIS20R) **eTable 27.** Median (IQR) Short Form Health Survey (SF-36) by Group **eTable 28.** Significant Change in Short Form Health Survey (SF-36) by Group **eTable 29.** Treatment Satisfaction by Group
eReferences.

Supplement 3. Data Sharing Statement
