# Supplementary material for: Discontinuation of First-Line Disease-Modifying Therapy in Patients With Stable Multiple Sclerosis: The DOT-MS Randomized Clinical Trial
Source: JAMA Neurol. 2024 Dec 9;82(2):123–31. doi: 10.1001/jamaneurol.2024.4164 (PMC11811793; doi:10.1001/jamaneurol.2024.4164)
Supplement: Supplement 1. — Trial Protocol [file jamaneurol-e244164-s001.pdf]

Discontinuation of first-line disease-modifying therapy in stable multiple sclerosis (DOT-MS)

Results of a randomized controlled trial

DOT-MS Trial Protocols

Table of contents

Original trial protocol – version 2

Trial protocol amendment 1 – version 3

Trial protocol amendment 2 – version 4

Trial protocol amendment 3 – version 5

Trial protocol amendment 4 – version 6

Trial protocol amendment 5 – version 7

Trial protocol amendment 6 – version 8

RESEARCH PROTOCOL

The safety and cost-effectiveness of discontinuing disease-modifying therapies in stable relapsing-onset multiple sclerosis (DOT-MS): a randomized rater-blinded multicenter trial.

(Version 2, December 2019)

TABLE OF CONTENTS

1. INTRODUCTION AND RATIONALE 11
2. OBJECTIVES 15
3. STUDY DESIGN 17
4. STUDY POPULATION 18
4.1 Population (base) 18
4.2 Inclusion criteria 19
4.3 Exclusion criteria 19
4.4 Sample size calculation 20
5. TREATMENT OF SUBJECTS 21
5.1 Investigational product/treatment 21
5.2 Use of co-intervention 21
5.3 Escape medication 21
6. INVESTIGATIONAL PRODUCT 21
7. NON-INVESTIGATIONAL PRODUCT 21
8. METHODS 21
8.1 Study parameters/endpoints 21
8.1.1 Main study parameter/endpoint 21
8.1.2 Secondary study parameters/endpoints (if applicable) 22
8.1.3 Other study parameters (if applicable) 22
8.2 Randomisation, blinding and treatment allocation 23
8.3 Study procedures 23
8.4 Withdrawal of individual subjects 25
8.4.1 Specific criteria for withdrawal (if applicable) 25
8.5 Replacement of individual subjects after withdrawal 26
8.6 Follow-up of subjects withdrawn from treatment 26
8.7 Premature termination of the study 26
9. SAFETY REPORTING 27
9.1 Temporary halt for reasons of subject safety 27
9.2 AEs, SAEs and SUSARs 27
9.2.1 Adverse events (AEs) 27
9.2.2 Serious adverse events (SAEs) 27
9.2.3 Suspected unexpected serious adverse reactions (SUSARs) 28
9.3 Annual safety report 28
9.4 Follow-up of adverse events 28
9.5 Data Safety Monitoring Board (DSMB) 28
10. STATISTICAL ANALYSIS 30
10.1 Primary study parameter(s) 30
10.2 Secondary study parameter(s) 31
10.3 Other study parameters 31
10.4 Interim analysis 31
11. ETHICAL CONSIDERATIONS 35

11.1 Regulation statement 35
11.2 Recruitment and consent....................35
11.3 Objection by minors or incapacitated subjects (if applicable)....................35
11.4 Benefits and risks assessment, group relatedness....................35
11.5 Compensation for injury 36
11.6 Incentives (if applicable)....................36
12. ADMINISTRATIVE ASPECTS, MONITORING AND PUBLICATION 37
12.1 Handling and storage of data and documents 37
12.2 Monitoring and Quality Assurance....................38
12.3 Amendments....................39
12.4 Annual progress report....................39
12.5 Temporary halt and (prematurely) end of study report....................39
12.6 Public disclosure and publication policy....................40
13. STRUCTURED RISK ANALYSIS....................41
13.1 Potential issues of concern....................41
13.2 Synthesis 41
14. REFERENCES 42

LIST OF ABBREVIATIONS AND RELEVANT DEFINITIONS

9HPT	9-Hole Peg Test
ABR	General Assessment and Registration form (ABR form), the application form that is required for submission to the accredited Ethics Committee; in Dutch: Algemeen Beoordelings- en Registratieformulier (ABR-formulier)
AE	Adverse Event
AR	Adverse Reaction
CA	Competent Authority
CCMO	Central Committee on Research Involving Human Subjects; in Dutch: Centrale Commissie Mensgebonden Onderzoek
CIS	Clinically Isolated Syndrome
CIS20r	Checklist Individual Strength
CV	Curriculum Vitae
DMT	Disease Modifying Therapy
DSMB	Data Safety Monitoring Board
EDSS	Expanded Disability Status Scale
EQ-5D-5L	EuroQol 5 Dimensions Questionnaire
EU	European Union
FLAIR	Fluid Attenuation Inversion Recovery
GCP	Good Clinical Practice
GDPR	General Data Protection Regulation; in Dutch: Algemene Verordening Gegevensbescherming (AVG)
IC	Informed Consent
iMCQ	Medical Consumption Questionnaire
iPCQ	Productivity Costs Questionnaire
METC	Medical research ethics committee (MREC); in Dutch: medisch-ethische toetsingscommissie (METC)
MRI	Magnetic Resonance Imaging
MS	Multiple Sclerosis
MSFC	Multiple Sclerosis Functional Composite
MSIS-29	Multiple Sclerosis Impact Scale
RRMS	Relapsing Remitting Multiple Sclerosis
(S)AE	(Serious) Adverse Event
SDMT	Symbol Digits Modalities Test

**Sponsor**	**The sponsor is the party that commissions the organisation or performance of the research, for example a pharmaceutical company, academic hospital, scientific organisation or investigator. A party that provides funding for a study but does not commission it is not regarded as the sponsor, but referred to as a subsidising party.**
**SF-36**	**Short Form Health Survey**
**SPMS**	**Secondary Progressive Multiple Sclerosis**
**SUSAR**	**Suspected Unexpected Serious Adverse Reaction**
**T25fW**	**Timed 25-foot Walk**
**TSQM**	**Treatment Satisfaction Questionnaire for Medication**
**UAVG**	**Dutch Act on Implementation of the General Data Protection Regulation; in Dutch: Uitvoeringswet AVG**
**WMO**	**Medical Research Involving Human Subjects Act; in Dutch: Wet Medisch-wetenschappelijk Onderzoek met Mensen**

SUMMARY

Rationale: The past few years, several new effective drugs have come onto the market for the treatment of relapsing remitting MS (RRMS), all of which have potentially serious side effects. The arrival of these drugs has led to a new aim for treating MS patients: achieving a status of complete clinical and radiological control of inflammatory events, also described as a status of no evident disease activity (NEDA-3). With these adjusted goals, medication is often started at an earlier stage and the disease is treated more aggressively. This leads to better control of the disease, but also to increased exposure to possible (serious) side effects. A considerable group of patients with a fully stable-disease under treatment merely have a benign or less inflammatory disease course rather than a necessity for treatment to prevent inflammation. This raises the question whether and when patients who have been stable under medication for years can safely discontinue the treatment. The hypothesis of this study is that discontinuing medication after >5 years without evidence of inflammatory disease activity does not result in return of inflammatory disease activity.

Objective: The aim of this study is to identify whether it is possible to safely discontinue treatment in MS patients who have shown no evidence of active inflammation in the years prior to inclusion clinically and/or radiologically. The secondary objectives address the questions whether the discontinuation of first-line treatment has an effect on disability progression and whether the discontinuation of first-line treatment improves the quality of life for the patient. Furthermore, blood collections will be included to assess whether it is possible to retrospectively predict possible return of inflammatory activity with biomarkers such as neurofilament light (NFL) or patient characteristics such as disease activity prior to disease modifying therapy (DMT). In case of emerging disease activity after the cessation of therapy we will assess if reinitiation will lead to NEDA again, and if there are long-term consequences. If possible, post-hoc analysis are performed for the different types of treatment compounds.

Study design: Multi-center randomized and controlled, rater-blinded trial in the Netherlands. 130 patients with relapse onset MS will be assigned to either discontinue the previously used DMT or to continue their DMT.

Study population: MS patients who are treated with one of the first-line treatments (any of the interferons, glatiramer acetate, dimethylfumarate, teriflunomide) and who had a complete absence of inflammatory activity (no relapses, no new-T2 lesions and no contrast-enhancing lesions) for 5 consecutive years under first-line treatment will be eligible for inclusion.

Intervention (if applicable): discontinuation of the previously used DMT.

Main study parameters/endpoints: The primary endpoint is the return of inflammatory disease activity after 2 years: either relapses, new or enlarging lesions on T2-weighted MRI and gadolinium-enhancing lesions on post-contrast T1-weighted MRI. Secondary end-points

are EDSS and MSFC progression (combined: EDSS plus), number of relapses, individual MRI-parameters (such as lesion numbers), quality of life measurements and biomarker measurements.

Nature and extent of the burden and risks associated with participation, benefit and group relatedness: The burden of participation consists of assessments during visits at baseline, 3, 6, 12, 18 and 24 months. Every follow-up visit of both patient groups will consist of clinical and radiological measurements, quality of life questionnaires and blood collection. The data on the discontinuation of therapy in long-term inflammatory stable MS patients is reassuring. One large retrospective cohort study has shown in 1200 patients that stable RRMS patients who stop treatment don't have an increased relapse rate compared to patients who continue treatment (Kister et al). There was a slightly higher disability progression of the discontinuation group, presumably based on the discontinuation of treatment in patients with progressive MS. Also, there seems to be no rebound inflammation after discontinuation of therapy in progressive MS, and a similar relapse rate as the years prior to discontinuation (Bonenfant et al). When disease activity emerges, patients are treated according to the current standard of care. The discontinuation of medication can be beneficial for the patient, as the side effects of medications can be significant for some patients.

1. INTRODUCTION AND RATIONALE

In recent decades, the therapeutic landscape of multiple sclerosis (MS) has changed dramatically. Coming from an era where virtually no therapies were available, there are currently more than 12 first- and second-line disease modifying treatment (DMT) options for the prevention of focal inflammatory demyelinating lesions in the brain and spinal cord. Clinically MS can be devastating; it affects roughly 1 in 1000 persons in the Netherlands usually diagnosed in the prime of their lives with a mean age at diagnosis around 30[1]. Two major disease phenotypes exist. The most important is relapsing-onset MS (80% MS patients), including patients with a clinically isolated syndrome (CIS), relapsing remitting MS (RRMS) and secondary progressive MS (SPMS). The other 20% suffers from a primary progressive MS (PPMS), a disease phenotype with more distinct neurodegeneration. Untreated, 50% of patients will need assistance walking small distances after 10-20 years after diagnosis[2].The main pathological hallmark in the first stages (CIS and RRMS) is recurrent focal inflammation of the brain and spinal cord leading to demyelination[3]. The first years after the diagnosis patients usually experience 2-3 relapses annually. The severity of neurological disability depends on the localisation of the inflammation. With increasing age, the amount of inflammation tends to diminish and an unknown neurodegenerative pathology drives the disease course. Clinically there is a progressive decline in neurological functioning; i.e. the “progressive” phase (or the secondary progressive (SPMS) disease course).

There has been great change in the timing of diagnosing MS and evaluating disease activity with the introduction of magnetic resonance imaging (MRI). In the early days the disease course was solely evaluated based on new relapses and/or progression of disability. Now, the arrival of MRI has led to a revised aim for treating MS patients: achieving a status of “no evident disease activity” (NEDA-3); complete clinical and radiological control of inflammatory events and no significant increase in diseability[4]. To date an estimated 10% of patients have a status of NEDA-3 under long-term first-line therapy, implying a full control of focal inflammation in these patients[5-7]. In recent years there has been a growing trend of starting treatment earlier and to treat more aggressively, partly based on the concept of NEDA. Treatment is almost always initiated directly after diagnosis but sometimes even before a definite diagnosis of RRMS is made[8]. There is however a substantial percentage of patients with a more benign disease course, described in a very broad range of 6-64% of MS patients[9-12]. At the moment of diagnosis, it is not known how the disease course will develop and based on the substantial group of “benign” MS cases, it is likely that a considerable group of patients that have long-term and fully stable-disease under DMT is unnecessarily

treated. In addition, there is the group of patients who experience disease progression despite their therapy. Also, these patients probably do not benefit from their therapy.

Exposure to treatment is not without risks and costs. Side-effects of MS medication are frequently present. Data on the proportion of patients discontinuing first-line DMT's demonstrates a discontinuation rate of 20-40% during an observation period over 1 year, with the occurrence of side effects and poor tolerability as the most common reason for drug withdrawal[13-15]. A large proportion of the patient population is confronted with side effects for both oral (teriflunomide, dimethylfumarate) and injectable (interferons, glatiramer acetate) DMT's. For example, the results of the post-approval clinical trials on the safety of teriflunomide compared to interferon beta-1a showed that 93-96% of the patients experienced side effects[16]. Each injectable first-line DMT can lead to mild cutaneous adverse events such as erythema and swelling, but also to more severe and persisting effects such as lipoatrophy, infections and even necrosis. Patients with a cutaneous reaction appeared to have a lower dermatology-specific health-related quality of life[17]. Serious events rates were also high ranging from 7% (interferon beta-1a) to 12% (teriflunomide)[16].

Each first-line DMT has a different mode of administration and specific side-effects. Interferons are administered biweekly s.c. (Plegridy), weekly i.m. (Avonex), second daily s.c. (Betaferon) or thrice weekly s.c. (Rebif). Flu-like symptoms are the most often reported side effects of interferon ß injections and are particularly challenging for MS patients[18,19]. But also allergic reactions, elevated liver enzymes leading to severe hepatic injury, thyroid autoimmunity, hypothyroidism and hematologic abnormalities might occur[18]. Glatiramer acetate (Copaxone) is injected subcutaneously daily or thrice weekly. Patterned reactions are most commonly reported in patients using glatiramer acetate, consisting of flushing, chest pain, palpitations, urticaria, anxiety and dyspnoea with a relative risk of 3.27. This patterned reaction unpredictably occur within minutes of injection and spontaneously resolve before 30 minutes[20]. Teriflunomide (Aubagio) is an oral drug and is administered daily. Hair thinning, increased blood pressure, fatigue, diarrhoea, sensory disturbances, elevated liver enzymes, and renal failure are adverse effects that have been reported in patients using teriflunomide[18]. The fourth agent in the first-line DMT group, dimethylfumarate (Tecfidera) is taken orally twice daily. Clinical phase 3 trials reported mild or moderate flushing and gastrointestinal (GI) adverse events, 36% and 42%, respectively as most common adverse effects[21-23]. Consequently, this led to treatment discontinuation in both trials. Serious side effects include urosepsis (interferons), hepatotoxicity (glatiramer acetate), but also progressive multifocal leukoencephalopathy (dimethyl fumarate), which are all potentially lethal.

Furthermore, there is a great burden of costs to society. The costs for a year of first-line immunomodulating drugs range from 12.000 – 15.000 euros annually[24]. The discontinuation of therapy in appropriate patients therefore also has a very significant effect with a potential cost-reduction of 2-2.5 million euros annually in The Netherlands. Costs due to side-effects (such as treatment and absence of work) are not even included here.

The data on the discontinuation of therapy in long-term inflammatory stable MS patients is reassuring. One large retrospective cohort study has shown in 1200 patients that stable RRMS patients who stop treatment don't have an increased relapse rate compared to patients who continue treatment[25]. There was a slightly higher disability progression of the discontinuation group, presumably based on the discontinuation of treatment in patients with progressive MS. Also, there seems to be no rebound inflammation after discontinuation of therapy in progressive MS, and a similar relapse rate as the years prior to discontinuation[26]. Lastly, one study has showed that patients 45 years or older, or patients with a DMT intake of 4 or more years without evidence of clinical or radiological disease activity showed a high likelihood of remaining relapse-free after discontinuation and absence of contrast enhancing lesions[27]. All studies where however hampered by either its retrospective nature, or incompleteness on for examples reasons for discontinuation or a sufficiently matched control group. Obviously, definite conclusions can only be drawn after a well-designed controlled trial. Currently, two trials with a similar question to ours are underway (DISCOMS; NCT03073603 and STOP-I-SEP; NCT03653273). These studies are different compared to our proposal in that the inclusion criteria only allow for older patients (older than 55 years and older than 50 years respectively) to be included.

The question of whether or not to discontinue therapy is increasingly present during our outpatient clinics [28,29]. As stated, an estimated 10% of patients have a status of NEDA-3 under long-term first-line DMT and could benefit from the results of this study. To give an indication of the number of patients concerned: in the Netherlands alone an estimated number of 7000-7500 patients with MS use first-line DMT, which means 700-750 patients would be eligible for this study and would benefit from results of this study (numbers are based on market shares and sales of each DMT, data not published). An enquiry amongst Dutch MS neurologists (data not published) shows that every neurologist struggles with the question on average 5-10 times per year. The Dutch 2012 CBO guideline " Multiple Sclerosis" recommends the discontinuation of DMT in patients who experience severe side-effects and who had secondary progressive MS for at least 3 years[30] but this is solely based on expert opinion. Also, internationally there are no guidelines guiding patients and neurologists in this question. Very recently in 2017, the committee of care evaluation of

neurology (‘Zorgevaluatie Neurologie’ (ZEN), part of the ‘Dutch society of Neurologists (Nederlandse Vereniging voor Neurologie; NVN) and supported by the Dutch Federation of medical specialists (FMS) and the Dutch Federation of Patients) has ranked this lack of evidence regarding the discontinuation of immunomodulating drugs in MS one of the most important science/knowledge gaps within daily neurological practice in the Netherlands. It listed this topic in its “ Kennisagenda 2018-2022” which prioritizes the 12 most urgent topics for scientific research in the field of neurology in the coming 4 years to improve the effectiveness and safety of our daily medical practice[31]. We have validated this support by means of a questionnaire amongst all 88 members of the MS Taskforce (Part of the NVN). All (100%) respondents (response-rate was 49%) indicated that they found the issue frequently present in their clinics and important tot research. Also, internationally there is strong call for more evidence-based guidelines and consensus regarding the discontinuation of treatment in long-term stable MS patients[32]. This underlines the national (and international) need for systematic research regarding this subject.

2. OBJECTIVES

With this study we will bring first evidence to this important issue by identifying whether it is possible to safely discontinue treatment in MS patients who have shown no evidence of active inflammation in the years prior to inclusion. Importantly, we include quality of life measurements to evaluate if this also translates to an increased perception of health. If present, it will optimize the treatment paradigm for individual MS patients through identifying unnecessary exposure to medication while proving the discontinuation of medication to be safe, and beneficial for the daily lives of individual patients in terms of an increased quality of life. In addition, it greatly benefits the general society since it also provides a more efficient use of funds with the significant cost-reduction it brings. The impact of the trial is immediate, within 5 years, and since all large MS centers will participate there is a direct line into the daily offices of treating neurologists, and into the development of adjusted guidelines regarding the treatment of MS. Most importantly, every possible result deriving from this trial will have a significant impact on (inter)national treatment strategies. Besides the primary question of the evaluation of safety we also incorporate validated measures of quality of life and disease perception to evaluate potential changes in quality of life. Furthermore, we include blood collections for the monitoring of neurofilament light (currently the only validated and clinically applied biomarker for the return of inflammatory activity) that could potentially serve as indicator for subclinical return of inflammatory activity. See also methods section and literature review for a full description.

Research questions

<u>Primary research question:</u>

Can we safely discontinue first-line medication in MS patients with long-term absence of inflammation, without the return of *inflammatory disease activity* clinically and radiologically?

<u>Other research questions:</u>

- Does the discontinuation of first-line treatment have an effect on disability progression?
- Does the discontinuation of first-line treatment improve the quality of life for the patient?
- What is the effect of discontinuation of first-line treatment on individual MRI outcome measures such as lesion load and atrophy measurements?
- Is it possible to predict possible return of inflammatory activity with biomarkers such as neurofilament light (NFL) or patient characteristics such as disease activity prior to DMT?

- In case of emerging disease activity after cessation of DMT, will a restart of DMT result in NEDA again and if so, how long does it take?
- In case of emerging disease activity after treatment cessation, are there any differences between the different DMT compounds?
- What is the cost-effectiveness of discontinuation of DMT in The Netherlands?

3. STUDY DESIGN

The study design is a multi-center rater-blinded randomized controlled trial in the Netherlands. The project will take place over period of 5 years (60 months) depending on the progress of inclusion. The lead and monitoring of the trial will be performed by the MS center Amsterdam (MSCA). Participating centers are listed in an appendix (I1).

Study-population: 130 relapse onset MS patients who are treated with one of the first-line treatments (any of the interferons, glatiramer acetate, dimethylfumarate, teriflunomide) *and* who had a complete absence of objective inflammatory activity (no objectified relapses, no significant number (2 or more) of new-T2 lesions and no contrast-enhancing lesions) for 5 consecutive years under first-line treatment will be eligible for inclusion. Patients may not have switched between first-line drugs over the two years prior to inclusion. If a switch has occurred this should not have been due to ineffectivity of the first DMT but due to side-effects or by a personal preference of the patient (such as the wish to switch to oral therapies). In the case of previous use of interferons patients must be negative for neutralizing antibodies (NAbs). Inclusion will take place after informed consent. This will be obtained after careful and extensive information about the possible risks according to local ethical review board requirements.

Intervention: The intervention is the discontinuation of the previously used DMT. Based on the pharmacological profile of the abovementioned drugs that are eligible for inclusion, there is no need for a tapering of dosage before complete discontinuation

Follow-up frequency: Patients in the continuation group are expected to remain stable throughout the study period based on their proven stable status for 5 consecutive years prior to enrolment. The minimum evaluation that must take place should have a frequency according to the current standard of care (yearly evaluation of every patient treated with immunomodulating therapy; i.e. baseline (BL), 12 and 24 months). Timely recognition of recurrence of any (subclinical) disease activity in the discontinuation group is secured by more frequent clinical and radiological assessment during extra routine study visits at 3, 6 and 18 months. For optimal comparability and to overcome potential bias, both groups will be followed with a complete assessment including MRI at BL, 3, 6, 12, 18 and 24 months.

After these two years of follow-up, the patients in the continuation group are offered to discontinue their DMT with a follow-up similar to the discontinuation group.

4. STUDY POPULATION

4.1 Population (base)

We will include 130 participants with the diagnosis relapse onset MS with a minimum age of 18 years. 65 patients will be assigned to the discontinuation group and 65 patients will be assigned to the continuation group. MS patients will be recruited through the VUmc outpatient clinic and the outpatient neurology departments of all participating centers. Because this research has a multicenter national design, MS patients of the majority of regions in the Netherlands will be included, including urban as well as rural areas. We believe that represents a valid cross-section of the average MS patient population. It is important to note however, as MS affects 2-2.5x as many women as men we expect to include more women than men. We will include a randomization algorithm to match both groups for sex to exclude potential bias.

We have estimated 700-750 patients to be potentially eligible for inclusion in The Netherlands. Currently an estimated number of 7000-7500 patients use first-line DMT for their MS. This number is estimated on data that was provided by all different pharmaceutical companies of each of the first-line therapies. Numbers are based on market shares and actual sales of each of the treatments (data not published).

The percentage of “stability” derives from the treatment effects described in the pivotal phase III trials of the currently available first-line treatments and 3 cohort studies with a longer follow-up that the average 2 years in the phase III trials. For our calculation of possible eligible patients, we have used the most “negative” scenario based on the results of these trials. The most recent phase III studies report on NEDA as outcome measure (Havrdova et al 2017, Arnold et al 2017), reflecting the new treatment concept of MS: complete stabilization of the disease process. In those cohorts NEDA ranges from 25-40% (Havrdova et al 2017, Arnold et al 2014 & 2017, Miller et al 2014, Nygaard et al 2015). It is important to note however, that the concept of NEDA also includes progressive neurological decline (such as is due to progressive disease and neurodegeneration). The percentage of patients that is free from inflammation is therefore somewhat underestimated in the numbers of these studies. Furthermore, most studies comprise on average a relatively short observation period of 2 years.

Three cohort studies exist with a longer follow-up. De Stefano et al, Uher et al and Rotstein et al published cohort studies that describe a longitudinal follow-up of NEDA status of ~ 200 patients for 7-10 years[33-35]. They showed that a fairly low percentage of patients remained

NEDA after 5 years (10-15%). In the cases of emerging inflammatory activity under treatment, most patients showed disease activity in the first 1-2 years after the start of treatment. Only a small minority of patients lose their NEDA status after 5 years (4%). It is very important to note that in the Rotstein-study the loss of NEDA status was in a large proportion due to disease progression rather than new inflammation. This causes a relative underestimation of patients who remain inflammatory stable. Taken together and based on this data, we assume that in the of all first-line DMT users 10% show no signs of inflammation for 5 years, and only a very small percentage of patients shows additional disease activity while staying on treatment (5%). The majority (95%) of patients remain inflammatory stable while continuing their medication.

This makes 700-750 patients eligible for inclusion.

4.2 Inclusion criteria

In order to be eligible to participate in this study, a subject must meet all of the following criteria:

1. A minimum age of 18 years
2. Ability to understand the purpose and risks of the study and provide signed and dated informed consent and authorization to use protected health information (PHI) in accordance with national and local privacy regulations.
3. Definite diagnosis of relapsing-onset MS according to the revised McDonald 2017 criteria
4. All relapsing-onset MS patients treated with one of the first-line treatments: any of the interferons, glatiramer acetate, dimethylfumarate, teriflunomide
5. Complete absence of inflammatory activity (no objectively defined and confirmed relapses, no significant number (2 or more) of new-T2 lesions and no contrast-enhancing lesions) for 5 consecutive years under first-line treatment

4.3 Exclusion criteria

A potential subject who meets any of the following criteria will be excluded from participation in this study:

1. A switch between first-line disease modifying therapy over two years prior to inclusion, in case the switch has been due to in effectivity of the first DMT. In case the switch

has been due to side-effects or by a personal preference of the patient (such as the wish to switch to oral therapies), this is not considered as an exclusion criterium.

2. Women who want to discontinue medication because of a pregnancy wish and women who are pregnant or expect to become pregnant during the study period
3. Patients that have previously used interferon-beta and have been tested positive for neutralizing antibodies (NAbs). This is determined by measuring MxA-bioactivity and is a test that is part of routine follow-up in patients that use interferon-beta. The reason for this is that development of NAbs has been shown to affect interferon-beta treatment efficacy.

4.4 Sample size calculation

The stability of patients in the continuation group is estimated to be at least 97,5%. Based on a non-inferiority margin of 7,5%, a preliminary power-calculation based on the non-inferiority principle was performed (PASS v12, one-sided Z-test (unpooled), significance level 0.05) and showed a necessary sample size of 54 per group to achieve 80% power. Taking 20% drop out into consideration, the total sample size needed for this study is 130.

5. TREATMENT OF SUBJECTS

5.1 Investigational product/treatment

The intervention consists of the discontinuation of the previously used DMT (either interferons, glatiramer acetate, dimethylfumarate or teriflunomide) Based on the pharmacological profile of the abovementioned drugs that are eligible for inclusion, there is no need for a tapering of dosage before complete discontinuation.

5.2 Use of co-intervention

During the intervention period patients are asked not to participate in any other scientific studies. Patients are allowed to use all types of co-medication, except for immunomodulating drugs such as prescribed for the treatment of multiple sclerosis and/or other auto-immune diseases.

5.3 Escape medication

When disease activity emerges, patients are treated according to the current standard of care (including intravenous methylprednisolone if deemed necessary) and disease modifying treatment will be reinitiated. Unscheduled visits including an MRI-scan are planned for each patient with any new neurological complaints, as is standard clinical procedure.

6. INVESTIGATIONAL PRODUCT

N/A

7. NON-INVESTIGATIONAL PRODUCT

N/A

8. METHODS

8.1 Study parameters/endpoints

8.1.1 Main study parameter/endpoint

The primary endpoint is number of patients with return of inflammatory disease activity after 2 years based on: a clinically confirmed relapse (defined according to the definition most often used in MS phase-III trials: the onset of new or recurrent symptoms that last > 24 hours, that are accompanied by new objective abnormalities on a neurological examination and that are not explained by non-MS processes such as fever, infection, severe stress or drug toxicity (Gold et al NEJM 2012)) , or any emerging subclinical disease activity proven to be due to active disease/new inflammation (defined as 3 or more lesions on T2—weighted

images or 2 or more gadolinium enhancing lesions on T1-weighted post-contrast MRI) in the discontinuation group.

8.1.2 Secondary study parameters/endpoints (if applicable)

Secondary end-points are

- Changes in neurological functioning
  - EDSS change (Including individual functional systems)
  - MSFC changes
    - Timed 25-foot Walk (T25fW)
    - 9-Hole Peg Test (9HPT)
    - Symbol digits modalities test (SDMT)
- Individual MRI-parameters
  - T1 post-contrast lesion numbers and volumes
  - T2 lesion numbers and volumes
  - Whole brain volume
  - Normalized white matter volume
  - Grey matter volume
- Changes in quality of life measurements
  - Multiple Sclerosis Impact Scale (MSIS-29)
  - Short Form health survey (SF-36)
  - Checklist Individual Strength (CIS20r)
  - Treatment Satisfaction Questionnaire for Medication (TSQM)
- Cost measurements
  - EuroQol 5 dimensions questionnaire (EQ-5D-5L)
  - Medical consumption questionnaire (iMCQ)
  - Productivity costs questionnaire (iPCQ)
- Changes in biomarker measurements
  - Neurofilament levels

8.1.3 Other study parameters (if applicable)

Vitamin D, smoking high body-mass index (BMI) are considered as potential confounders. Therefore, vitamin D will be determined and patients will be asked about smoking behaviour during every visit. To avoid any potential bias, we will also collect data (if present in the individual patients) on disease activity prior to the initiation of DMT, such date of diagnosis, time from first symptoms to diagnosis, EDSS scores/MS severity at the moment of DMT initiation.

8.2 Randomisation, blinding and treatment allocation

Since MS affects 2-2.5x as many women as men, we expect to include more women than men. A randomization algorithm will be included to match both groups for sex and age to exclude potential bias.

Outcome measurements will be performed by assessors who are blind to the intervention assignment. For reasons of consistency and to exclude possible bias, all scans will be centrally reviewed in VUmc by a radiologist blinded to allocation to the intervention group. In the current set-up of the trial patients and their treating neurologists are unblinded to the randomization group. The currently available first-line disease modifying treatments consist of the various (peg)interferons, glatiramer acetate, teriflunomide and dimethylfumarate. It comes in a total of more 10 different forms with different packaging, different modes of injection (some subcutaneously, some intramuscular and some oral) and different frequencies of intake (ranging from twice daily to once every two weeks). We have set up the trial so that it is rater-blinded; all MRI-scan and clinical evaluations (such as the neurological examination) will be performed blinded to “treatment” allocation. The primary and majority of secondary outcome measures are derived from these blinded assessments. This approach is frequently chosen in MS research (even in the phase-III trials) for 2 very important reasons. Due to the very distinct nature of side-effects (flushing, gastro-intestinal problems, flu-like symptoms), patients know when they receive placebo instead of the actual active compound. The invasive nature of receiving placebo-injections would greatly enhance the possibility of patients not participating. In addition, we believe it is too costly to create a placebo-control for each of the 10 different forms of medication.

8.3 Study procedures

No study procedures will take place before having obtained informed consent which will be gained following current METc/CCMO standards.

Follow-up frequency

Patients in the continuation group are expected to remain stable throughout the study period based on their proven stable status for 5 consecutive years prior to enrolment. The minimum evaluation that must take place should have a frequency according to the current standard of care (yearly evaluation of every patient treated with immunomodulating therapy; i.e. baseline (BL), 12 and 24 months). Timely recognition of recurrence of any (subclinical) disease activity in the discontinuation group is secured by more frequent clinical and radiological assessment during extra routine study visits at 3, 6 and 18 months. For optimal comparability

and to overcome potential bias , both groups will be followed with a complete assessment including MRI at BL, 3, 6, 12, 18 and 24 moths.

Clinical evaluation

Duration: 1 hour

Clinical evaluation will consist of a careful medical history: current and past medication, adverse events, number of intravenous methylprednisolone treatments, number of relapses, date of last relapse and signs of symptom progression. A relapse is defined according to the definition most often used in MS phase-III trials: the onset of new or recurrent symptoms that last > 24 hours, that are accompanied by new objective abnormalities on a neurological examination and that are not explained by non-MS processes such as fever, infection, severe stress or drug toxicity[21]. Furthermore, the Expanded Disability Status Scale (EDSS) and Multiple Sclerosis Functional Composite (MSFC)-measurements will be performed, consisting of the Timed 25-foot Walk (T25fW), 9-Hole Peg Test (9HPT) and the symbol digits modalities test (SDMT). This will be done by a blinded investigator.

Radiological evaluation

Duration: 45 min

Radiological evaluation will consist of repeated brain MRI investigations that consist of conventional pre- and post-contrast (T2-weighted, T1-weighted pre and post contrast, FLAIR) images. All scan protocols are available in general and academic hospitals since they form the basis on which MS is diagnosed and treatment is monitored. Although it is expected that new inflammatory lesions can be captured by repeated T2-weighted/FLAIR MRI-scans, a gadolinium-scan is included to not miss any contrast enhancement in previously present lesions. A 3DT1 and 3DFLAIR image will also be made for atrophy measurements. For reasons of consistency and to exclude possible bias, all scans will be centrally reviewed in VUmc by a radiologist blinded to allocation to the intervention group.

Questionnaires

Duration: 1 hour

For the evaluation of disease burden and MS related symptoms, we will use the validated and in clinical studies often used Multiple Sclerosis Impact Scale (MSIS-29)[36] Short Form health survey (SF-36)[37] and CIS20r[38]. The perceived impact of treatment, convenience, satisfaction and side-effects will be monitored using the Treatment Satisfaction Questionnaire for Medication (TSQM)[39]. For the evaluation of health related quality of life (HRQoL) and the link between symptoms HRQoL and costs, we use the EDSS for the objective measurement of changes in neurological functioning (which has a clear relation to

HRQoL assessed as utility and costs[40]) and the number of patients with return of inflammatory activity. We also use the EQ5D-5L) for cost-utility analysis[41]. Furthermore, we will ask patients to keep a diary describing changes in healthcare consumption (which will be defined per item in a questionnaire (such as hospitalisations, consultations with doctors, use of care at home, use of specialized transportation etc). Lastly patients will be asked for their employment situation and short-term and long-term sick absence because of MS (or MS related treatment) using the iMCQ and iPCQ questionnaires[42]. Questionnaires are filled in digitally either at home or during the hospital visit. Help of a carer or the study-nurse is allowed in cases of the inability using a computer.

Samples

Duration: 15 minutes

Blood collection will take place in both patient groups at every visit. It will consist blood collection for the purpose of biobanking and for diagnostics in the case of any –unforeseen-clinical events. Furthermore, we will retrospectively measure neurofilament light in serum using Simoa.

All participants will visit the hospital for 6 times over a time frame of 2 years. Each of these visits that take place will take approximately 2,5-3 hours (clinical assessment and MRI-scan). Also 1-hour questionnaires which can be completed at home digitally or at the hospital on paper will take place before/during each visit.

All samples will be collected, processed and stored according to the Standard Operating Procedures (SOP's) as described in the Parelsnoer Biobankprotocol version 8.0. (15) Samples will be stored at the Biobank VUmc. To ensure patient privacy all samples will be coded. A peripheral blood sample will be collected, in total 8 tubes of blood will be drawn (5x EDTA 4 ml for plasma, cells and DNA isolation, 2x serum 5 ml, 1x PAXgene tube 2 ml), adding up to a total volume of 32 ml. Blood will be centrifuged, divided in aliquots of 0.5 ml and then stored at -80 °C.

8.4 Withdrawal of individual subjects

Subjects can leave the study at any time for any reason if they wish to do so without any consequences. The investigator can decide to withdraw a subject from the study for urgent medical reasons.

8.4.1 Specific criteria for withdrawal (if applicable)

There are no specific criteria for withdrawal from the study.

8.5 Replacement of individual subjects after withdrawal

There will be no replacement of individual subjects after withdrawal.

8.6 Follow-up of subjects withdrawn from treatment

If a patient is withdrawn from the study, we will still perform follow-up measurements in case the patient is willing and able to cooperate.

8.7 Premature termination of the study

The procedure in case of premature termination of the study is described in section 10.4 and 12.5.

9. SAFETY REPORTING

9.1 Temporary halt for reasons of subject safety

In accordance to section 10, subsection 4, of the WMO, the sponsor will suspend the study if there is sufficient ground that continuation of the study will jeopardise subject health or safety. The sponsor will notify the accredited METC without undue delay of a temporary halt including the reason for such an action. The study will be suspended pending a further positive decision by the accredited METC. The investigator will take care that all subjects are kept informed.

More information about temporary halt for reasons of subject safety is provided in section 10.4 and 12.5.

9.2 AEs, SAEs and SUSARs

9.2.1 Adverse events (AEs)

Adverse events are defined as any undesirable experience occurring to a subject during the study, whether or not considered related to the experimental intervention. All adverse events that are reported spontaneously by the subject or observed by the investigator or his staff and that are relevant to the study will be recorded.

9.2.2 Serious adverse events (SAEs)

A serious adverse event is any untoward medical occurrence or effect that

- results in death;
- is life threatening (at the time of the event);
- requires hospitalisation or prolongation of existing inpatients' hospitalisation;
- results in persistent or significant disability or incapacity;
- is a congenital anomaly or birth defect; or
- any other important medical event that did not result in any of the outcomes listed above due to medical or surgical intervention but could have been based upon appropriate judgement by the investigator.

The investigator will report all SAEs to the sponsor without undue delay after obtaining knowledge of the *events*. The sponsor will report the SAEs through the web portal *ToetsingOnline* to the accredited METC that approved the protocol, within 7 days of first knowledge for SAEs that result in death or are life threatening followed by a period of maximum of 8 days to complete the initial preliminary report. All other SAEs will be reported

within a period of maximum 15 days after the sponsor has first knowledge of the serious adverse events.

9.2.3 Suspected unexpected serious adverse reactions (SUSARs)

This section is not applicable since this study does not investigational medicinal products.

9.3 Annual safety report

This section is not applicable since this study does not investigational medicinal products.

9.4 Follow-up of adverse events

All AEs will be followed until they have abated, or until a stable situation has been reached. Depending on the event, follow up may require additional tests or medical procedures as indicated, and/or referral to the general physician or a medical specialist.
SAEs need to be reported until the end of study, as defined in the protocol in section 9.2.2.

9.5 Data Safety Monitoring Board (DSMB)

For optimal safety we will appoint an independent data safety monitoring board (DSMB)which will monitor trial data on a regular basis. The aim of the committee is to safeguard the interests of trial participants and assess the safety of the discontinuation of therapy during the trial. The specific role of the DSMB consists of monitoring evidence for harm due to the intervention (discontinuing medication). The DSMB may advice to terminate the trial prematurely if disease activity exceeds above mentioned thresholds (see section 10.4 for interim analyses).

The DSMB consists of 4 members who do not have conflict of interest with the sponsor or the study. In addition to the permanent members of the DSMB two external members are added to the DSMB with expertise in the relevant field of research (yet to be determined).

The DSMB will conduct interim analyses in a predetermined manner and at predetermined times (see section 10.4), to see whether the relationship between clinical benefit and burden remains acceptable to the subject during the study. After each interim analysis, the DSMB reports to the study coordinator, with reports to the METc and the study sponsor, i.e. the Board of Directors (Raad van Bestuur). The DSMB can give advice to continue, change or

stop (parts of) the study. The DSMB will also ensure the quality and safety of research in the participating centers.

10. STATISTICAL ANALYSIS

All data is quantitative and will be presented in tables and graphs. Baseline data will be collected to detect any potential differences between the two investigated groups for which we have not corrected with the randomization procedure (sex and age). Possible other confounders include smoking habits, vitamin D levels but also previous disease course in terms of differences in disease duration, number of relapses prior to stability, years of use of treatment.

The primary endpoint is the number of patients with return of inflammatory disease activity after 2 years based on: a clinically confirmed relapse or any emerging subclinical disease activity proven to be due to active disease/new inflammation (defined as 3 or more lesions on T2—weighted images or 2 or more gadolinium enhancing lesions on T1-weighted post-contrast MRI) in the discontinuation group. Secondary end-points are: 1. Changes in neurological functioning (EDSS/MSFC changes including individual functional systems and MSFC subtests), 2. Individual MRI parameters (T2 and T1 post-contrast lesion numbers), 3. changes in quality of life measurements (SF-36, CIS20r, TSQM, EQ5D-5L, iMCQ and iPCQ) and 4. changes in biomarker measurements (neurofilament levels).

In the case of confirmation of our hypothesis (discontinuing medication after >5 years without evidence of inflammatory disease activity does not result in return of inflammatory disease activity), we will extend the trial with a follow-up of two years where the group that continued their treatment gets offered the possibility to discontinue under similar safety measures (with similar follow-up frequencies and endpoints as the primary trial).

10.1 Primary study parameter(s)

For the primary endpoint, the return of inflammatory disease activity after 2 years, a2x2 contingency table will be used to estimate the risk difference for the return of inflammatory disease activity after 2 years (yes/no) between the two groups. The risk difference will be calculated for discontinuation relative to continuation. If the lower bound of the corresponding two-sided 90% confidence interval exceeds -7.5% we conclude non-inferiority of discontinuing medication For the primary endpoint both a per protocol analysis and a intention-to-treat analysis will be performed.

The primary endpoint is the number of patients with return of inflammatory disease activity after 2 years based on: a clinically confirmed relapse or any emerging subclinical disease activity proven to be due to active disease/new inflammation (defined as 3 or more lesions on

T2—weighted images or 2 or more gadolinium enhancing lesions on T1-weighted post-contrast MRI) in the discontinuation group.

A relapse is defined according to the definition most often used in MS phase-III trials: the onset of new or recurrent symptoms that last > 24 hours, that are accompanied by new objective abnormalities on a neurological examination and that are not explained by non-MS processes such as fever, infection, severe stress or drug toxicity[21]

Furthermore, a survival analysis (with an intention-to-treat approach) regarding the time to return of inflammatory activity will be included.

10.2 Secondary study parameter(s)

For all the secondary endpoints regression analysis (either linear of logistic, depending on the type of variable) will be performed correcting for possible confounders.

10.3 Other study parameters

N/A

10.4 Interim analysis

We will build in a safety-strategy (go-no-go strategy) during the first 1,5 year of the study to control for emerging disease activity (and patients safety) in the discontinuation group. This implicates that after every visit, patients are evaluated for (sub)clinical and radiological disease activity. For this safety strategy, we will make a distinction between patients <55 years old and patients ≥55 years old based on mechanistic differences of recurrent inflammation in both groups. In case of an objectified MS relapse, or any emerging subclinical disease activity proven to be due to active disease/new inflammation (defined as 3 or more lesions on T2—weighted images of 2 or more gadolinium enhancing lesions on T1-weighted post-contrast MRI) in the discontinuation group in:

- ≥5% of patients in the first 6 months of the study in either one of both age groups (<55 years old or ≥55 years old) or;
- ≥10% of either one of both age groups within the first 12 months of the study, or;
- ≥15% of either one of both age groups within the first 18 months of follow-up,

we may prematurely stop the study for a specific age group. Distribution within a specific age group will determine whether the entire age group will be advised to restart DMT or that restart may be restricted to a specific subgroup. In that case, we will continue the study for the other patients according to the protocol. In case of a premature stop of the trial for a

specific age group of patients that discontinued their DMT, we will follow the patient group for which the trial was discontinued in an open label extension study with follow-up frequencies and assessments similar to the trial.

For optimal safety the DSMB will monitor the decision making on premature termination every 3 months. The DSMB may advice to terminate the trial prematurely if disease activity exceeds above mentioned thresholds. See section 9.5 for a more detailed description of the DSMB.

The procedure in case of premature termination of the study is described in section 12.5.

10.5 Cost-analysis

Alongside this trial, we will conduct a cost-effectiveness analysis and a cost-utility analysis over a 2-year follow-up. These will be performed in accordance with the recommendations of the Dutch guideline for health economic evaluations. For the cost-effectiveness analysis, the return of inflammatory disease activity (either relapse or new of enlarging lesions) will serve as the effectiveness measure. The cost-utility analysis will focus on quality of life measured with the EQ-5D-5L, which is often used in MS research. Quality-adjusted life-years (QALYs) will be calculated by multiplying the utility scores belonging to a health state by the amount of time spent in this health state using linear interpolation between time points.

In both analyses, costs will be measured from a societal perspective including health-care costs (such as the costs for a year of DMT, costs for the extra surveillance including additional medical tests as MRI, costs for visits to other medical doctors etc), direct nonmedical costs (costs that patients make for travelling to and from the hospital, private payments for extra health-care consumption etc) and indirect nonmedical costs (costs due to loss of production and short or long-term sick absence). The latter is important as previous research has shown that productivity losses are an important cost driver in severe MS. Health-care costs and direct nonmedical costs will be measured using the iMTA Medical Consumption Questionnaire (iMCQ) at 3 months, 6, months, 12 months, 18 months and 24 months. The iMCQ measures the health-care costs in the last three months. As these patients are assumed to be stable regarding their disease progression, these follow-up moments will provide an adequate estimation of their health-care use. Primary and secondary health-care costs will be valued using Dutch standard costs. If unavailable, tariffs or costs reported by the literature will be used. Medication will be valued using

www.medicijnkosten.nl whereas informal care will be valued based on the costs of household care as reported by the Centraal Administratie Kantoor.
For indirect nonmedical costs, patients will be asked for their employment situation and both short-term and long-term sick absence because of MS (or MS related treatment) using the iMTA Productivity Cost Questionnaire (iPCQ) at 3 months, 6, months, 12 months, 18 months and 24 months. The iPCQ measures productivity loss in the last four weeks which will give an adequate overview of the productivity losses between each time point as these patients are assumed to be stable. Costs of absenteeism from paid work will be calculated according to both the human capital and friction cost approach. Costs of presenteeism will be calculated by asking participants how many working hours should have been replaced due to less productivity at work. Lost productivity due to either absenteeism or presenteeism will be valued using the mean age-, and sex specific income of the Dutch population. Costs of productivity losses due to absenteeism from unpaid work and informal care will be calculated using the standard wage of a professional housekeeper. All costs will be indexed to the year at which the trial ended. Missing data on costs and effects will be imputed using multiple imputations. In addition, costs and effects will both be discounted using a 3% discount rate.

For the cost-effectiveness analysis, we will calculate incremental cost-effectiveness ratios (ICERs) which is defined as the difference in mean costs divided by the difference in mean effects between the treatment continuation group and the treatment discontinuation group. For the cost-utility analysis, we will calculate incremental cost-utility ratios (ICURs) which is the difference in mean costs divided by the difference in mean QALYs. Bootstrapping with 5,000 replications will be used to estimate 95% credibility intervals around the ICERs and ICURs. The bootstrapped cost-effect pairs will be plotted on a cost-effectiveness plane and used to estimate cost-effectiveness acceptability curves (CEACs). CEACs show the probability that the intervention is cost-effective in comparison with the control treatment for a range of ceiling ratios. The ceiling ratio is defined as the willingness-to-pay, which is the amount of money society is willing to pay to gain one unit of effect.

In a sensitivity analysis, we will repeat all analyses using a healthcare payer perspective. In this analysis, only direct healthcare costs will be included. In addition, we will conduct subgroup analysis in which we will stratify individuals based on the presence of blood-based markers predictive for return of inflammatory disease activity.

Furthermore, we will conduct a budget impact analysis. A budget impact analysis (BIA) focuses on the expected changes in the expenditure of a health care system after the adoption of a new standard of care. In this BIA, we aim to estimate the future yearly budget

impact of discontinuation of first line medication in patients with long-term stable relapsing-onset from a Dutch perspective. The BIA will be performed according to the BIA framework of the International Society for Pharmacoeconomics and Outcomes Research (ISPOR). This framework consists of several standard aspects: target population, scenario distribution based on hospital types, resource utilisation, costs per unit, total costs, and sensitivity analyses.

11. ETHICAL CONSIDERATIONS

11.1 Regulation statement

The study will be conducted according to the principles of the Declaration of Helsinki (World Medical Association, 2013, Brazil) and in accordance with the Medical Research Involving Human Subjects Act (WMO) and the Good Clinical Practice guidelines.

11.2 Recruitment and consent

Patients will be informed about the study in different ways. Patients can be notified by their treating doctor during outpatient consultations at the VUmc or one of the other participating centres. In addition, a notification will be placed on the website of the MS Centre Amsterdam and the websites of the MS Vereniging Nederland (MSVN) and MS Web with information about the study. Patients can then discuss potential interest in the study with their treating neurologist. Inclusion is possible in one of the participating centers. Potential participants who express their wish to participate will receive additional information on paper about the purpose, intervention, duration and content of the study. They will also receive an informed consent form with careful and extensive information about the possible risks (according to local ethical review board requirements). In case there are any questions about the study, the study coordinator can be contacted. Moreover, an independent doctor (dr. Pijnenburg) can be contacted for additional questions.

Inclusion will take place after the written informed consent form has been returned to the trial coordinator. A copy of the informed consent form will be given to the participant and to the responsible physician in one of the participating centers in case the patient is recruited through one of the centers outside the VUmc. This consent can be revoked at any time without citing reasons. Patients will be given a minimum of 2 weeks to consider their decision. The maximum time will be as long as the inclusion of patients is ongoing and inclusion and exclusion criteria are met.

11.3 Objection by minors or incapacitated subjects (if applicable)

Not applicable, all participants will be adult and legally competent

11.4 Benefits and risks assessment, group relatedness

In the non-intervention group (continuation group) participants are expected to remain stable throughout the study period based on their proven stable status for 5 consecutive years prior to enrolment. Therefore, this group will face no potential risks and no direct benefit other than the usual medical care. The potential value of the outcome of the research will outweigh the

burden of participation for the study. Also, patients in the continuation group are offered the possibility to discontinue their medication under similar surveillance measures as the discontinuation group after 24-month follow-up period has ended.

The data on the discontinuation of therapy in long-term inflammatory stable MS patients is reassuring. One large retrospective cohort study has shown in 1200 patients that stable RRMS patients who stop treatment don't have an increased relapse rate compared to patients who continue treatment[25]. There was a slightly higher disability progression of the discontinuation group, presumably based on the discontinuation of treatment in patients with progressive MS. Also, there seems to be no rebound inflammation after discontinuation of therapy in progressive MS, and a similar relapse rate as the years prior to discontinuation[26]. Lastly, one study has showed that patients 45 years or older, or patients with a DMT intake of 4 or more years without evidence of clinical or radiological disease activity showed a high likelihood of remaining relapse-free after discontinuation and absence of contrast enhancing lesions[27]

11.5 Compensation for injury

According to article 7 from the 'Wet medisch-wetenschappelijk onderzoek met mensen' (Staatsblad 1998, 161) an insurance is obtained by the VUmc. In case of injury or death of the participants because of the study, this insurance will compensate for injury or cover the cost caused by death or injury from the participants. The insurance is obtained by the Onderlinge Waarborgmaatschappij Centramed b.a., Postbus 7374, 2701 AJ Zoetermeer. The insurance company and the insurance accede to the decree mandatory insurance for 'medisch-wetenschappelijk onderzoek met mensen (Staatsblad 2003, 266). Written information about the insurance will be provided for the participants.

11.6 Incentives (if applicable)

According to the current standard of care, the evaluation of every patient treated with immunomodulating therapy is at baseline, 12 and 24 months. Since visits are also scheduled at 3, 6 and 18 months, travel expenses and parking costs for these extra visits will be compensated. Participants will be compensated for their time and effort for study participation: they will receive a gift certificate of €25 upon study completion.

12. ADMINISTRATIVE ASPECTS, MONITORING AND PUBLICATION

12.1 Handling and storage of data and documents

Data will be handled confidentially . After collection, all data will be correctly labeled and securely stored. A subject identification code (SIC) will be used to link data to the subject. The SIC will consist of numbers and will not be based on the patient initials and birth-date. The key to the code will be kept separately from the coded data. The only people who have access to this code will be the principal investigator, the coordinating investigator and the corresponding investigator. No other people will have access to the link information. Great care will be taken to ensure that there is no link between SIC and information on which an individual can be identified. The handling of personal data in the database complies with the General Data Protection Regulation (De Algemene Verordening Gegevensbescherming) Potential data exchange with other countries will only take place after consent of the patient and handling of data will comply with the General Data Protection Regulation. The procedure for handling data includes data encryption, coding, secure storage, establishing limited access or varying levels of access to the biobank, removing identifying information from bio specimens and data. The infrastructure will consist of both hardware and software components, to prevent unauthorized access to databases.

An electronic case report form (CRF) will be developed to document the data collected in the study. This database will include demographic and patients characteristics (without birth date) and all outcomes of the study measures. Other investigators can request permission to get access to (a part) of this database for the purpose of research only, and only when the principal investigator gives permission. These investigators will not get access to the separate database which includes the participants' names, other identifiers and the SIC. All data will be stored on a computer protected with a password on the VUmc computer network. And access to the database will also be secured by a code. Only the trial coordinator and the principal investigator will know the code that gives access to the database with the link information.

After finalizing the study, the originals of all source documents will be stored for a period of 15 years in a locked room. Data that is collected and stored for the Biobank, will be stored for a period of 50 years. This period of storage has been determined to ensure that a follow-up study might be possible. In case of a follow-up study, a new protocol will be submitted to the METC and participants will have to sign a new informed consent form. Importantly, participants will only be approached for a follow-up study if they have indicated on the

informed consent form of the current study that they can be approached for a follow-up study.

The collection of data for medical research in the Netherlands is subject to the Personal Data Protection Act and in particular to the Medical Treatment Contracts Act.

12.2 Monitoring and Quality Assurance

An independent monitor, the Clinical Research Bureau (CRB) of the VUmc, will monitor the proposed study according to Good Clinical Practice (GCP). For a selection of candidates Informed consent is to be checked by the CRB. Besides that, source data verification is performed during the onsite monitoring. The conformity of the data used for analysis and the information in the patient files will be checked by the CRB. The intensity of the verification will be related to the risk arisen by the research. Inclusion and exclusion criteria will be checked as well as the main outcome measures. The CRB will check if the (S)AE's and SUSAR's are reported conforming the schedule as required by laws and regulations.

The quality assurance team under the leadership of a quality assurance manager (QAM) is responsible for providing an effective and efficient quality assurance system and counsel for the clinical research sites. In this quality assurance system, the QAM is responsible for ensuring appropriate global and affiliate-specific quality documents are developed and tracked, making sure they maintain an up-to-date overall inventory of all quality documents. Furthermore, the QAM and its team are responsible for ensuring all personnel involved in the clinical trial are properly qualified and trained for the job roles for which they are responsible. They are responsible for giving the personnel trainings and constantly assessing further opportunities for education and additional training. The quality assurance team is also responsible for checking compliance with the protocol, SOPs, GCP, and/or applicable regulatory requirement(s) and checking of the quality in all stages of data handling to ensure that all data are reliable and have been processed correctly. Moreover, the quality assurance team is responsible for auditing the various investigational sites.
If noncompliance with the protocol, SOPs, GCP, and/or applicable regulatory requirement(s) by and investigator/institution, or by member(s) or the sponsor's staff is detected during a quality assurance activity or audit, it is the responsibility of the QAM to report this to the trial's sponsor and principal investigator.

Quality assurance procedures:

Quality assurance is the systematic and independent examination of all clinical trial-related activities and documentations. The quality assurance procedure focuses on clinical investigator audits and audits of clinical trial documentation.

1. Document audits:

During the document audits, the quality assurance team oversees the documents that are generated before, during or at the end of the conduct of the clinical trial. For each document, a checklist is developed based on the relevant regulatory and organizational standards and SOPs. The aim of the audits is to ensure that the information and data in the documents are complete, clear, reliable and consistent. Documents reviewed in the document audits include the clinical study protocol, the investigator's brochure and the clinical study report.

2. Clinical investigator audits:

The clinical investigator audits concern audits of the different research sites of the trial. They are performed to assess the site's regulatory compliance and clinical data quality (including adherence to the protocol). Paragraph 3 will address the procedures concerning these audits.

12.3 Amendments

Amendments are changes made to the research after a favourable opinion by the accredited METC has been given. All amendments will be notified to the METC that gave a favourable opinion. All amendments will be notified to the METC and to the competent authority.

12.4 Annual progress report

The investigator will submit a summary of the progress of the trial to the accredited METC once a year. Information will be provided on the date of inclusion of the first subject, numbers of subjects included and numbers of subjects that have completed the trial, serious adverse events/ serious adverse reactions, other problems, and amendments. The METC will also be informed on the start and end date of the trial.

12.5 Temporary halt and (prematurely) end of study report

The investigator/sponsor will notify the accredited METC of the end of the study within a period of 8 weeks. The end of the study is defined as the last patient's last visit.
The sponsor will notify the METC immediately of a temporary halt of the study, including the reason of such an action.

In case the study is ended prematurely, the sponsor will notify the accredited METC within 15 days, including the reasons for the premature termination.
Within one year after the end of the study, the investigator/sponsor will submit a final study report with the results of the study, including any publications/abstracts of the study, to the accredited METC.

12.6 Public disclosure and publication policy

This study will be registered in het Nederlands Trial Register (NTR) http://www.trialregister.nl and www.clinicaltrials.gov. Publication will be in accordance with the basic principles of CCMO statement on publication policy. The results will be presented at (inter)national scientific meetings. The results will be published in a medical scientific journal. In none of the publication forms, participant identity will be disclosed.

13. STRUCTURED RISK ANALYSIS

13.1 Potential issues of concern

Paragraph 13.1 is not applicable.

13.2 Synthesis

The intervention in this study is the discontinuation of previously used DMT. No new products or agents are administered, nor will there be any dosage adjustments in the group that will continue their therapy. The specific DMT’s that patients use prior to discontinuation, and that are used in the control group are all registered with the authorities and widely used for this specific indication. Although previous studies suggest that the risk of return of inflammatory activity after discontinuing DMT will be low in long-term stable RRMS patients (as also described in section 1), this is the main risk of the intervention. To monitor return of inflammatory activity, a safety strategy is built in, which is described in section 10.4. If safety criteria are exceeded, the study will be discontinued and DMT’s will be reinitiated (in one patient group or in all patients, see section 10.4). A DSMB is appointed that will monitor the decision making on premature termination every 3 months (section 9.5).

14. REFERENCES

1 Goodin, D. S. The epidemiology of multiple sclerosis: insights to disease pathogenesis. *Handb Clin Neurol* **122**, 231-266, doi:10.1016/B978-0-444-52001-2.00010-8 (2014).
2 Weinshenker, B. G. Natural history of multiple sclerosis. *Ann Neurol* **36 Suppl**, S6-11 (1994).
3 Reich, D. S., Lucchinetti, C. F. & Calabresi, P. A. Multiple Sclerosis. *N Engl J Med* **378**, 169-180, doi:10.1056/NEJMra1401483 (2018).
4 Giovannoni, G. *et al.* Is it time to target no evident disease activity (NEDA) in multiple sclerosis? *Mult Scler Relat Disord* **4**, 329-333, doi:10.1016/j.msard.2015.04.006 (2015).
5 Arnold, D. L. *et al.* Peginterferon beta-1a improves MRI measures and increases the proportion of patients with no evidence of disease activity in relapsing-remitting multiple sclerosis: 2-year results from the ADVANCE randomized controlled trial. *BMC Neurol* **17**, 29, doi:10.1186/s12883-017-0799-0 (2017).
6 Havrdova, E. *et al.* Effect of delayed-release dimethyl fumarate on no evidence of disease activity in relapsing-remitting multiple sclerosis: integrated analysis of the phase III DEFINE and CONFIRM studies. *Eur J Neurol* **24**, 726-733, doi:10.1111/ene.13272 (2017).
7 Miller, A. E. *et al.* Oral teriflunomide for patients with a first clinical episode suggestive of multiple sclerosis (TOPIC): a randomised, double-blind, placebo-controlled, phase 3 trial. *Lancet Neurol* **13**, 977-986, doi:10.1016/S1474-4422(14)70191-7 (2014).
8 Freedman, M. S. *et al.* Moving toward earlier treatment of multiple sclerosis: Findings from a decade of clinical trials and implications for clinical practice. *Mult Scler Relat Disord* **3**, 147-155, doi:10.1016/j.msard.2013.07.001 (2014).
9 Ramsaransing, G. S. & De Keyser, J. Benign course in multiple sclerosis: a review. *Acta Neurol Scand* **113**, 359-369, doi:10.1111/j.1600-0404.2006.00637.x (2006).
10 Sartori, A., Abdoli, M. & Freedman, M. S. Can we predict benign multiple sclerosis? Results of a 20-year long-term follow-up study. *J Neurol* **264**, 1068-1075, doi:10.1007/s00415-017-8487-y (2017).
11 Benedikz, J. *et al.* The natural history of untreated multiple sclerosis in Iceland. A total population-based 50 year prospective study. *Clin Neurol Neurosurg* **104**, 208-210 (2002).
12 Perini, P., Tagliaferri, C., Belloni, M., Biasi, G. & Gallo, P. The HLA-DR13 haplotype is associated with "benign" multiple sclerosis in northeast Italy. *Neurology* **57**, 158-159 (2001).
13 Johnson, K. M., Zhou, H., Lin, F., Ko, J. J. & Herrera, V. Real-World Adherence and Persistence to Oral Disease-Modifying Therapies in Multiple Sclerosis Patients Over 1 Year. *J Manag Care Spec Pharm* **23**, 844-852, doi:10.18553/jmcp.2017.23.8.844 (2017).
14 Lattanzi, S. *et al.* Persistence to oral disease-modifying therapies in multiple sclerosis patients. *Journal of neurology* **264**, 2325-2329, doi:10.1007/s00415-017-8595-8 (2017).
15 Lanzillo, R. *et al.* A multicentRE observational analysiS of PErsistenCe to Treatment in the new multiple sclerosis era: the RESPECT study. *Journal of neurology* **265**, 1174-1183, doi:10.1007/s00415-018-8831-x (2018).
16 Vermersch, P. *et al.* Teriflunomide versus subcutaneous interferon beta-1a in patients with relapsing multiple sclerosis: a randomised, controlled phase 3 trial. *Multiple sclerosis (Houndmills, Basingstoke, England)* **20**, 705-716, doi:10.1177/1352458513507821 (2014).
17 Balak, D. M. *et al.* Prevalence of cutaneous adverse events associated with long-term disease-modifying therapy and their impact on health-related quality of life in patients with multiple sclerosis: a cross-sectional study. *BMC neurology* **13**, 146, doi:10.1186/1471-2377-13-146 (2013).
18 Rommer, P. S. & Zettl, U. K. Managing the side effects of multiple sclerosis therapy: pharmacotherapy options for patients. *Expert opinion on pharmacotherapy* **19**, 483-498, doi:10.1080/14656566.2018.1446944 (2018).
19 Lee Mortensen, G. & Rasmussen, P. V. The impact of quality of life on treatment preferences in multiple sclerosis patients. *Patient Prefer Adherence* **11**, 1789-1796, doi:10.2147/ppa.S142373 (2017).
20 La Mantia, L., Munari, L. M. & Lovati, R. Glatiramer acetate for multiple sclerosis. *Cochrane Database Syst Rev*, Cd004678, doi:10.1002/14651858.CD004678.pub2 (2010).
21 Gold, R. *et al.* Placebo-controlled phase 3 study of oral BG-12 for relapsing multiple sclerosis. *N Engl J Med* **367**, 1098-1107, doi:10.1056/NEJMoa1114287 (2012).

22 Fox, R. J. *et al.* Placebo-controlled phase 3 study of oral BG-12 or glatiramer in multiple sclerosis. *N Engl J Med* **367**, 1087-1097, doi:10.1056/NEJMoa1206328 (2012).
23 Sejbaek, T., Nybo, M., Petersen, T. & Illes, Z. Real-life persistence and tolerability with dimethyl fumarate. *Mult Scler Relat Disord* **24**, 42-46, doi:10.1016/j.msard.2018.05.007 (2018).
24 CIBG; ministerie van Volksgezondheid, W. e. S. *Prijsvorming*, <www.farmatec.nl> (z.d.).
25 Kister, I. *et al.* Discontinuing disease-modifying therapy in MS after a prolonged relapse-free period: a propensity score-matched study. *J Neurol Neurosurg Psychiatry* **87**, 1133-1137, doi:10.1136/jnnp-2016-313760 (2016).
26 Bonenfant, J. *et al.* Can we stop immunomodulatory treatments in secondary progressive multiple sclerosis? *Eur J Neurol* **24**, 237-244, doi:10.1111/ene.13181 (2017).
27 Bsteh, G. *et al.* Discontinuation of disease-modifying therapies in multiple sclerosis - Clinical outcome and prognostic factors. *Mult Scler* **23**, 1241-1248, doi:10.1177/1352458516675751 (2017).
28 O'Rourke, K. E. & Hutchinson, M. Stopping beta-interferon therapy in multiple sclerosis: an analysis of stopping patterns. *Mult Scler* **11**, 46-50, doi:10.1191/1352458505ms1131oa (2005).
29 Rio, J. *et al.* Factors related with treatment adherence to interferon beta and glatiramer acetate therapy in multiple sclerosis. *Mult Scler* **11**, 306-309, doi:10.1191/1352458505ms1173oa (2005).
30 CBO richtlijn (2012) Immunomodulerende en immunosuppressieve behandeling bij multipele sclerose. (2012).
31 Kennisagenda neurologie 2017: https://gallery.mailchimp.com/29087cdad5c58a12bd346e83f/files/3b5692f1-3840-48e2-99e7-405edb9a895f/Kennisagenda_Neurologie_eindversie_16_12_2017.pdf.
32 Kister, I. & Corboy, J. R. Reducing costs while enhancing quality of care in MS. *Neurology* **87**, 1617-1622, doi:10.1212/WNL.0000000000003113 (2016).
33 Uher, T. *et al.* Is no evidence of disease activity an achievable goal in MS patients on intramuscular interferon beta-1a treatment over long-term follow-up? *Mult Scler* **23**, 242-252, doi:10.1177/1352458516650525 (2017).
34 Rotstein, D. L., Healy, B. C., Malik, M. T., Chitnis, T. & Weiner, H. L. Evaluation of no evidence of disease activity in a 7-year longitudinal multiple sclerosis cohort. *JAMA Neurol* **72**, 152-158, doi:10.1001/jamaneurol.2014.3537 (2015).
35 De Stefano, N. *et al.* Long-term assessment of no evidence of disease activity in relapsing-remitting MS. *Neurology* **85**, 1722-1723, doi:10.1212/WNL.0000000000002105 (2015).
36 Gray, O., McDonnell, G. & Hawkins, S. Tried and tested: the psychometric properties of the multiple sclerosis impact scale (MSIS-29) in a population-based study. *Mult Scler* **15**, 75-80, doi:10.1177/1352458508096872 (2009).
37 Pfennings, L. E. *et al.* A health-related quality of life questionnaire for multiple sclerosis patients. *Acta Neurol Scand* **100**, 148-155 (1999).
38 Rietberg, M. B., Van Wegen, E. E. & Kwakkel, G. Measuring fatigue in patients with multiple sclerosis: reproducibility, responsiveness and concurrent validity of three Dutch self-report questionnaires. *Disabil Rehabil* **32**, 1870-1876, doi:10.3109/09638281003734458 (2010).
39 Eagle, T. *et al.* Treatment satisfaction across injectable, infusion, and oral disease-modifying therapies for multiple sclerosis. *Mult Scler Relat Disord* **18**, 196-201, doi:10.1016/j.msard.2017.10.002 (2017).
40 Kobelt, G., Berg, J., Lindgren, P. & Jonsson, B. Costs and quality of life in multiple sclerosis in Europe: method of assessment and analysis. *Eur J Health Econ* **7 Suppl 2**, S5-13, doi:10.1007/s10198-006-0365-y (2006).
41 Janssen, M. F. *et al.* Measurement properties of the EQ-5D-5L compared to the EQ-5D-3L across eight patient groups: a multi-country study. *Qual Life Res* **22**, 1717-1727, doi:10.1007/s11136-012-0322-4 (2013).
42 Bouwmans, C. *et al.* The iMTA Productivity Cost Questionnaire: A Standardized Instrument for Measuring and Valuing Health-Related Productivity Losses. *Value Health* **18**, 753-758, doi:10.1016/j.jval.2015.05.009 (2015).

RESEARCH PROTOCOL

The safety and cost-effectiveness of discontinuing disease-modifying therapies in stable relapsing-onset multiple sclerosis (DOT-MS): a randomized rater-blinded multicenter trial.

Version 3, March 2020

TABLE OF CONTENTS

1. INTRODUCTION AND RATIONALE 11
2. OBJECTIVES 15
3. STUDY DESIGN 17
4. STUDY POPULATION 18
4.1 Population (base) 18
4.2 Inclusion criteria 19
4.3 Exclusion criteria 19
4.4 Sample size calculation 20
5. TREATMENT OF SUBJECTS 21
5.1 Investigational product/treatment 21
5.2 Use of co-intervention 21
5.3 Escape medication 21
6. INVESTIGATIONAL PRODUCT 21
7. NON-INVESTIGATIONAL PRODUCT 21
8. METHODS 21
8.1 Study parameters/endpoints 21
8.1.1 Main study parameter/endpoint 21
8.1.2 Secondary study parameters/endpoints (if applicable) 22
8.1.3 Other study parameters (if applicable) 23
8.2 Randomisation, blinding and treatment allocation 23
8.3 Study procedures 23
8.4 Withdrawal of individual subjects 27
8.4.1 Specific criteria for withdrawal (if applicable) 27
8.5 Replacement of individual subjects after withdrawal 27
8.6 Follow-up of subjects withdrawn from treatment 27
8.7 Premature termination of the study 28
9. SAFETY REPORTING 29
9.1 Temporary halt for reasons of subject safety 29
9.2 AEs, SAEs and SUSARs 29
9.2.1 Adverse events (AEs) 29
9.2.2 Serious adverse events (SAEs) 29
9.2.3 Suspected unexpected serious adverse reactions (SUSARs) 30
9.3 Annual safety report 30
9.4 Follow-up of adverse events 30
9.5 Data Safety Monitoring Board (DSMB) 30
10. STATISTICAL ANALYSIS 32
10.1 Primary study parameter(s) 32
10.2 Secondary study parameter(s) 33
10.3 Other study parameters 33
10.4 Interim analysis 33
11. ETHICAL CONSIDERATIONS 37

11.1 Regulation statement 37
11.2 Recruitment and consent........ 37
11.3 Objection by minors or incapacitated subjects (if applicable)........ 37
11.4 Benefits and risks assessment, group relatedness 37
11.5 Compensation for injury 38
11.6 Incentives (if applicable)........ 38
12. ADMINISTRATIVE ASPECTS, MONITORING AND PUBLICATION 39
12.1 Handling and storage of data and documents 39
12.2 Monitoring and Quality Assurance........ 40
12.3 Amendments........ 42
12.4 Annual progress report........ 42
12.5 Temporary halt and (prematurely) end of study report........ 42
12.6 Public disclosure and publication policy........ 42
13. STRUCTURED RISK ANALYSIS........ 44
13.1 Potential issues of concern........ 44
13.2 Synthesis 44
14. REFERENCES 45

LIST OF ABBREVIATIONS AND RELEVANT DEFINITIONS

9HPT	9-Hole Peg Test
ABR	General Assessment and Registration form (ABR form), the application form that is required for submission to the accredited Ethics Committee; in Dutch: Algemeen Beoordelings- en Registratieformulier (ABR-formulier)
AE	Adverse Event
AR	Adverse Reaction
CA	Competent Authority
CCMO	Central Committee on Research Involving Human Subjects; in Dutch: Centrale Commissie Mensgebonden Onderzoek
CIS	Clinically Isolated Syndrome
CIS20r	Checklist Individual Strength
CV	Curriculum Vitae
DMT	Disease Modifying Therapy
DSMB	Data Safety Monitoring Board
EDSS	Expanded Disability Status Scale
EQ-5D-5L	EuroQol 5 Dimensions Questionnaire
EU	European Union
FLAIR	Fluid Attenuation Inversion Recovery
GCP	Good Clinical Practice
GDPR	General Data Protection Regulation; in Dutch: Algemene Verordening Gegevensbescherming (AVG)
IC	Informed Consent
iMCQ	Medical Consumption Questionnaire
iPCQ	Productivity Costs Questionnaire
METC	Medical research ethics committee (MREC); in Dutch: medisch-ethische toetsingscommissie (METC)
MRI	Magnetic Resonance Imaging
MS	Multiple Sclerosis
MSFC	Multiple Sclerosis Functional Composite
MSIS-29	Multiple Sclerosis Impact Scale
RRMS	Relapsing Remitting Multiple Sclerosis
(S)AE	(Serious) Adverse Event
SDMT	Symbol Digits Modalities Test

**Sponsor**	**The sponsor is the party that commissions the organisation or performance of the research, for example a pharmaceutical company, academic hospital, scientific organisation or investigator. A party that provides funding for a study but does not commission it is not regarded as the sponsor, but referred to as a subsidising party.**
**SF-36**	**Short Form Health Survey**
**SPMS**	**Secondary Progressive Multiple Sclerosis**
**SUSAR**	**Suspected Unexpected Serious Adverse Reaction**
**T25fW**	**Timed 25-foot Walk**
**TSQM**	**Treatment Satisfaction Questionnaire for Medication**
**UAVG**	**Dutch Act on Implementation of the General Data Protection Regulation; in Dutch: Uitvoeringswet AVG**
**WMO**	**Medical Research Involving Human Subjects Act; in Dutch: Wet Medisch-wetenschappelijk Onderzoek met Mensen**

SUMMARY

Rationale: The past few years, several new effective drugs have come onto the market for the treatment of relapsing remitting MS (RRMS), all of which have potentially serious side effects. The arrival of these drugs has led to a new aim for treating MS patients: achieving a status of complete clinical and radiological control of inflammatory events, also described as a status of no evident disease activity (NEDA-3). With these adjusted goals, medication is often started at an earlier stage and the disease is treated more aggressively. This leads to better control of the disease, but also to increased exposure to possible (serious) side effects. A considerable group of patients with a fully stable-disease under treatment merely have a benign or less inflammatory disease course rather than a necessity for treatment to prevent inflammation. This raises the question whether and when patients who have been stable under medication for years can safely discontinue the treatment. The hypothesis of this study is that discontinuing medication after >5 years without evidence of inflammatory disease activity does not result in return of inflammatory disease activity.

Objective: The aim of this study is to identify whether it is possible to safely discontinue treatment in MS patients who have shown no evidence of active inflammation in the years prior to inclusion clinically and/or radiologically. The secondary objectives address the questions whether the discontinuation of first-line treatment has an effect on disability progression and whether the discontinuation of first-line treatment improves the quality of life for the patient and if this can be measured in a daily setting using digital biomarkers.. Furthermore, blood collections will be included to assess whether it is possible to retrospectively predict possible return of inflammatory activity with biomarkers such as neurofilament light (NFL) or patient characteristics such as disease activity prior to disease modifying therapy (DMT). In case of emerging disease activity after the cessation of therapy we will assess if reinitiation will lead to NEDA again, and if there are long-term consequences. If possible, post-hoc analysis are performed for the different types of treatment compounds.

Study design: Multi-center randomized and controlled, rater-blinded trial in the Netherlands. 130 patients with relapse onset MS will be assigned to either discontinue the previously used DMT or to continue their DMT.

Study population: MS patients who are treated with one of the first-line treatments (any of the interferons, glatiramer acetate, dimethylfumarate, teriflunomide) and who had a complete absence of inflammatory activity (no relapses, no new-T2 lesions and no contrast-enhancing lesions) for 5 consecutive years under first-line treatment will be eligible for inclusion.

Intervention (if applicable): discontinuation of the previously used DMT.

Main study parameters/endpoints: The primary endpoint is the return of inflammatory disease activity after 2 years: either relapses, new or enlarging lesions on T2-weighted MRI

and gadolinium-enhancing lesions on post-contrast T1-weighted MRI. Secondary end-points are EDSS and MSFC progression (combined: EDSS plus), number of relapses, individual MRI-parameters (such as lesion numbers), quality of life measurements and (digital) biomarker measurements.

Nature and extent of the burden and risks associated with participation, benefit and group relatedness: The burden of participation consists of assessments during visits at baseline, 3, 6, 12, 18 and 24 months. Every follow-up visit of both patient groups will consist of clinical and radiological measurements, quality of life questionnaires and blood collection. Additional data will be collected via mobile applications MS sherpa and Neurokeys, that will be installed on patients' smartphones. For this, patients will be asked to perform tasks on their phones, measuring walking ability, hand function, cognition and fatigue. This takes approximately five minutes every two weeks and is performed at home. The data on the discontinuation of therapy in long-term inflammatory stable MS patients is reassuring. One large retrospective cohort study has shown in 1200 patients that stable RRMS patients who stop treatment don't have an increased relapse rate compared to patients who continue treatment (Kister et al). There was a slightly higher disability progression of the discontinuation group, presumably based on the discontinuation of treatment in patients with progressive MS. Also, there seems to be no rebound inflammation after discontinuation of therapy in progressive MS, and a similar relapse rate as the years prior to discontinuation (Bonenfant et al). When disease activity emerges, patients are treated according to the current standard of care. The discontinuation of medication can be beneficial for the patient, as the side effects of medications can be significant for some patients.

1. INTRODUCTION AND RATIONALE

In recent decades, the therapeutic landscape of multiple sclerosis (MS) has changed dramatically. Coming from an era where virtually no therapies were available, there are currently more than 12 first- and second-line disease modifying treatment (DMT) options for the prevention of focal inflammatory demyelinating lesions in the brain and spinal cord. Clinically MS can be devastating; it affects roughly 1 in 1000 persons in the Netherlands usually diagnosed in the prime of their lives with a mean age at diagnosis around 30[1]. Two major disease phenotypes exist. The most important is relapsing-onset MS (80% MS patients), including patients with a clinically isolated syndrome (CIS), relapsing remitting MS (RRMS) and secondary progressive MS (SPMS). The other 20% suffers from a primary progressive MS (PPMS), a disease phenotype with more distinct neurodegeneration. Untreated, 50% of patients will need assistance walking small distances after 10-20 years after diagnosis[2].The main pathological hallmark in the first stages (CIS and RRMS) is recurrent focal inflammation of the brain and spinal cord leading to demyelination[3]. The first years after the diagnosis patients usually experience 2-3 relapses annually. The severity of neurological disability depends on the localisation of the inflammation. With increasing age, the amount of inflammation tends to diminish and an unknown neurodegenerative pathology drives the disease course. Clinically there is a progressive decline in neurological functioning; i.e. the “progressive” phase (or the secondary progressive (SPMS) disease course).

There has been great change in the timing of diagnosing MS and evaluating disease activity with the introduction of magnetic resonance imaging (MRI). In the early days the disease course was solely evaluated based on new relapses and/or progression of disability. Now, the arrival of MRI has led to a revised aim for treating MS patients: achieving a status of “no evident disease activity” (NEDA-3); complete clinical and radiological control of inflammatory events and no significant increase in diseability[4]. To date an estimated 10% of patients have a status of NEDA-3 under long-term first-line therapy, implying a full control of focal inflammation in these patients[5-7]. In recent years there has been a growing trend of starting treatment earlier and to treat more aggressively, partly based on the concept of NEDA. Treatment is almost always initiated directly after diagnosis but sometimes even before a definite diagnosis of RRMS is made[8]. There is however a substantial percentage of patients with a more benign disease course, described in a very broad range of 6-64% of MS patients[9-12]. At the moment of diagnosis, it is not known how the disease course will develop and based on the substantial group of “benign” MS cases, it is likely that a considerable group of patients that have long-term and fully stable-disease under DMT is unnecessarily

treated. In addition, there is the group of patients who experience disease progression despite their therapy. Also, these patients probably do not benefit from their therapy.

Exposure to treatment is not without risks and costs. Side-effects of MS medication are frequently present. Data on the proportion of patients discontinuing first-line DMT's demonstrates a discontinuation rate of 20-40% during an observation period over 1 year, with the occurrence of side effects and poor tolerability as the most common reason for drug withdrawal[13-15]. A large proportion of the patient population is confronted with side effects for both oral (teriflunomide, dimethylfumarate) and injectable (interferons, glatiramer acetate) DMT's. For example, the results of the post-approval clinical trials on the safety of teriflunomide compared to interferon beta-1a showed that 93-96% of the patients experienced side effects[16]. Each injectable first-line DMT can lead to mild cutaneous adverse events such as erythema and swelling, but also to more severe and persisting effects such as lipoatrophy, infections and even necrosis. Patients with a cutaneous reaction appeared to have a lower dermatology-specific health-related quality of life[17]. Serious events rates were also high ranging from 7% (interferon beta-1a) to 12% (teriflunomide)[16].

Each first-line DMT has a different mode of administration and specific side-effects. Interferons are administered biweekly s.c. (Plegridy), weekly i.m. (Avonex), second daily s.c. (Betaferon) or thrice weekly s.c. (Rebif). Flu-like symptoms are the most often reported side effects of interferon ß injections and are particularly challenging for MS patients[18,19]. But also allergic reactions, elevated liver enzymes leading to severe hepatic injury, thyroid autoimmunity, hypothyroidism and hematologic abnormalities might occur[18]. Glatiramer acetate (Copaxone) is injected subcutaneously daily or thrice weekly. Patterned reactions are most commonly reported in patients using glatiramer acetate, consisting of flushing, chest pain, palpitations, urticaria, anxiety and dyspnoea with a relative risk of 3.27. This patterned reaction unpredictably occur within minutes of injection and spontaneously resolve before 30 minutes[20]. Teriflunomide (Aubagio) is an oral drug and is administered daily. Hair thinning, increased blood pressure, fatigue, diarrhoea, sensory disturbances, elevated liver enzymes, and renal failure are adverse effects that have been reported in patients using teriflunomide[18]. The fourth agent in the first-line DMT group, dimethylfumarate (Tecfidera) is taken orally twice daily. Clinical phase 3 trials reported mild or moderate flushing and gastrointestinal (GI) adverse events, 36% and 42%, respectively as most common adverse effects[21-23]. Consequently, this led to treatment discontinuation in both trials. Serious side effects include urosepsis (interferons), hepatotoxicity (glatiramer acetate), but also progressive multifocal leukoencephalopathy (dimethyl fumarate), which are all potentially lethal.

Furthermore, there is a great burden of costs to society. The costs for a year of first-line immunomodulating drugs range from 12.000 – 15.000 euros annually[24]. The discontinuation of therapy in appropriate patients therefore also has a very significant effect with a potential cost-reduction of 2-2.5 million euros annually in The Netherlands. Costs due to side-effects (such as treatment and absence of work) are not even included here.

The data on the discontinuation of therapy in long-term inflammatory stable MS patients is reassuring. One large retrospective cohort study has shown in 1200 patients that stable RRMS patients who stop treatment don't have an increased relapse rate compared to patients who continue treatment[25]. There was a slightly higher disability progression of the discontinuation group, presumably based on the discontinuation of treatment in patients with progressive MS. Also, there seems to be no rebound inflammation after discontinuation of therapy in progressive MS, and a similar relapse rate as the years prior to discontinuation[26]. Lastly, one study has showed that patients 45 years or older, or patients with a DMT intake of 4 or more years without evidence of clinical or radiological disease activity showed a high likelihood of remaining relapse-free after discontinuation and absence of contrast enhancing lesions[27]. All studies where however hampered by either its retrospective nature, or incompleteness on for examples reasons for discontinuation or a sufficiently matched control group. Obviously, definite conclusions can only be drawn after a well-designed controlled trial. Currently, two trials with a similar question to ours are underway (DISCOMS; NCT03073603 and STOP-I-SEP; NCT03653273). These studies are different compared to our proposal in that the inclusion criteria only allow for older patients (older than 55 years and older than 50 years respectively) to be included.

The question of whether or not to discontinue therapy is increasingly present during our outpatient clinics [28,29]. As stated, an estimated 10% of patients have a status of NEDA-3 under long-term first-line DMT and could benefit from the results of this study. To give an indication of the number of patients concerned: in the Netherlands alone an estimated number of 7000-7500 patients with MS use first-line DMT, which means 700-750 patients would be eligible for this study and would benefit from results of this study (numbers are based on market shares and sales of each DMT, data not published). An enquiry amongst Dutch MS neurologists (data not published) shows that every neurologist struggles with the question on average 5-10 times per year. The Dutch 2012 CBO guideline " Multiple Sclerosis" recommends the discontinuation of DMT in patients who experience severe side-effects and who had secondary progressive MS for at least 3 years[30] but this is solely based on expert opinion. Also, internationally there are no guidelines guiding patients and neurologists in this question. Very recently in 2017, the committee of care evaluation of

neurology (‘Zorgevaluatie Neurologie’ (ZEN), part of the ‘Dutch society of Neurologists (Nederlandse Vereniging voor Neurologie; NVN) and supported by the Dutch Federation of medical specialists (FMS) and the Dutch Federation of Patients) has ranked this lack of evidence regarding the discontinuation of immunomodulating drugs in MS one of the most important science/knowledge gaps within daily neurological practice in the Netherlands. It listed this topic in its “ Kennisagenda 2018-2022” which prioritizes the 12 most urgent topics for scientific research in the field of neurology in the coming 4 years to improve the effectiveness and safety of our daily medical practice[31]. We have validated this support by means of a questionnaire amongst all 88 members of the MS Taskforce (Part of the NVN). All (100%) respondents (response-rate was 49%) indicated that they found the issue frequently present in their clinics and important tot research. Also, internationally there is strong call for more evidence-based guidelines and consensus regarding the discontinuation of treatment in long-term stable MS patients[32]. This underlines the national (and international) need for systematic research regarding this subject.

2. OBJECTIVES

With this study we will bring first evidence to this important issue by identifying whether it is possible to safely discontinue treatment in MS patients who have shown no evidence of active inflammation in the years prior to inclusion. Importantly, we include quality of life measurements to evaluate if this also translates to an increased perception of health. If present, it will optimize the treatment paradigm for individual MS patients through identifying unnecessary exposure to medication while proving the discontinuation of medication to be safe, and beneficial for the daily lives of individual patients in terms of an increased quality of life. In addition, it greatly benefits the general society since it also provides a more efficient use of funds with the significant cost-reduction it brings. The impact of the trial is immediate, within 5 years, and since all large MS centers will participate there is a direct line into the daily offices of treating neurologists, and into the development of adjusted guidelines regarding the treatment of MS. Most importantly, every possible result deriving from this trial will have a significant impact on (inter)national treatment strategies. Besides the primary question of the evaluation of safety we also incorporate validated measures of quality of life and disease perception to evaluate potential changes in quality of life. Furthermore, we include blood collections for the monitoring of neurofilament light (currently the only validated and clinically applied biomarker for the return of inflammatory activity) that could potentially serve as indicator for subclinical return of inflammatory activity.

Primary outcome measures used in this study are clinical and radiological outcome measures that are already part of routine clinical practice, which ensures that results of this trial can be directly and easily implemented in standard clinical care. In addition to these outcome measures, it would be of interest to determine optimal measurements for potential return of disease activity. A promising method in this regard is the use of mobile applications on patients' smartphones, because of their non-invasive nature (in contrast to for example MRI-scans) and the potential for more continuous measurements in a real-world setting (i.e. at home). Measurements with mobile applications MS sherpa and Neurokeys will be included for a subgroup of patients (depending on the availability of a smartphone), to investigate if return of inflammatory disease activity can be measured with these applications. These mobile applications will be installed on patients' smartphones.

See also methods section for a full description.

Research questions

<u>Primary research question:</u>

Can we safely discontinue first-line medication in MS patients with long-term absence of inflammation, without the return of *inflammatory disease activity* clinically and radiologically?

Other research questions:

- Does the discontinuation of first-line treatment have an effect on disability progression?
- Does the discontinuation of first-line treatment improve the quality of life for the patient?
- What is the effect of discontinuation of first-line treatment on individual MRI outcome measures such as lesion load and atrophy measurements?
- Is it possible to predict possible return of inflammatory activity with biomarkers such as neurofilament light (NFL) or patient characteristics such as disease activity prior to DMT?
- In case of emerging disease activity after cessation of DMT, will a restart of DMT result in NEDA again and if so, how long does it take?
- In case of emerging disease activity after treatment cessation, are there any differences between the different DMT compounds?
- What is the cost-effectiveness of discontinuation of DMT in The Netherlands?
- Is it possible to detect and predict (return of) inflammatory disease activity and disease progression with digital biomarkers using mobile applications such as MS sherpa and Neurokeys?

3. STUDY DESIGN

The study design is a multi-center rater-blinded randomized controlled trial in the Netherlands. The project will take place over period of 5 years (60 months) depending on the progress of inclusion. The lead and monitoring of the trial will be performed by the MS center Amsterdam (MSCA). Participating centers are listed in an appendix (I1).

Study-population: 130 relapse onset MS patients who are treated with one of the first-line treatments (any of the interferons, glatiramer acetate, dimethylfumarate, teriflunomide) *and* who had a complete absence of objective inflammatory activity (no objectified relapses, no significant number (2 or more) of new-T2 lesions and no contrast-enhancing lesions) for 5 consecutive years under first-line treatment will be eligible for inclusion. Patients may not have switched between first-line drugs over the two years prior to inclusion. If a switch has occurred this should not have been due to ineffectivity of the first DMT but due to side-effects or by a personal preference of the patient (such as the wish to switch to oral therapies). In the case of previous use of interferons patients must be negative for neutralizing antibodies (NAbs). Inclusion will take place after informed consent. This will be obtained after careful and extensive information about the possible risks according to local ethical review board requirements.

Intervention: The intervention is the discontinuation of the previously used DMT. Based on the pharmacological profile of the abovementioned drugs that are eligible for inclusion, there is no need for a tapering of dosage before complete discontinuation

Follow-up frequency: Patients in the continuation group are expected to remain stable throughout the study period based on their proven stable status for 5 consecutive years prior to enrolment. The minimum evaluation that must take place should have a frequency according to the current standard of care (yearly evaluation of every patient treated with immunomodulating therapy; i.e. baseline (BL), 12 and 24 months). Timely recognition of recurrence of any (subclinical) disease activity in the discontinuation group is secured by more frequent clinical and radiological assessment during extra routine study visits at 3, 6 and 18 months. For optimal comparability and to overcome potential bias, both groups will be followed with a complete assessment including MRI at BL, 3, 6, 12, 18 and 24 months.

After these two years of follow-up, the patients in the continuation group are offered to discontinue their DMT with a follow-up similar to the discontinuation group.

4. STUDY POPULATION

4.1 Population (base)

We will include 130 participants with the diagnosis relapse onset MS with a minimum age of 18 years. 65 patients will be assigned to the discontinuation group and 65 patients will be assigned to the continuation group. MS patients will be recruited through the VUmc outpatient clinic and the outpatient neurology departments of all participating centers. Because this research has a multicenter national design, MS patients of the majority of regions in the Netherlands will be included, including urban as well as rural areas. We believe that represents a valid cross-section of the average MS patient population. It is important to note however, as MS affects 2-2.5x as many women as men we expect to include more women than men. We will include a randomization algorithm to match both groups for sex to exclude potential bias.

We have estimated 700-750 patients to be potentially eligible for inclusion in The Netherlands. Currently an estimated number of 7000-7500 patients use first-line DMT for their MS. This number is estimated on data that was provided by all different pharmaceutical companies of each of the first-line therapies. Numbers are based on market shares and actual sales of each of the treatments (data not published).

The percentage of “stability” derives from the treatment effects described in the pivotal phase III trials of the currently available first-line treatments and 3 cohort studies with a longer follow-up that the average 2 years in the phase III trials. For our calculation of possible eligible patients, we have used the most “negative” scenario based on the results of these trials. The most recent phase III studies report on NEDA as outcome measure (Havrdova et al 2017, Arnold et al 2017), reflecting the new treatment concept of MS: complete stabilization of the disease process. In those cohorts NEDA ranges from 25-40% (Havrdova et al 2017, Arnold et al 2014 & 2017, Miller et al 2014, Nygaard et al 2015). It is important to note however, that the concept of NEDA also includes progressive neurological decline (such as is due to progressive disease and neurodegeneration). The percentage of patients that is free from inflammation is therefore somewhat underestimated in the numbers of these studies. Furthermore, most studies comprise on average a relatively short observation period of 2 years.

Three cohort studies exist with a longer follow-up. De Stefano et al, Uher et al and Rotstein et al published cohort studies that describe a longitudinal follow-up of NEDA status of ~ 200 patients for 7-10 years[33-35]. They showed that a fairly low percentage of patients remained

NEDA after 5 years (10-15%). In the cases of emerging inflammatory activity under treatment, most patients showed disease activity in the first 1-2 years after the start of treatment. Only a small minority of patients lose their NEDA status after 5 years (4%). It is very important to note that in the Rotstein-study the loss of NEDA status was in a large proportion due to disease progression rather than new inflammation. This causes a relative underestimation of patients who remain inflammatory stable. Taken together and based on this data, we assume that in the of all first-line DMT users 10% show no signs of inflammation for 5 years, and only a very small percentage of patients shows additional disease activity while staying on treatment (5%). The majority (95%) of patients remain inflammatory stable while continuing their medication.

This makes 700-750 patients eligible for inclusion.

4.2 Inclusion criteria

In order to be eligible to participate in this study, a subject must meet all of the following criteria:

1. A minimum age of 18 years
2. Ability to understand the purpose and risks of the study and provide signed and dated informed consent and authorization to use protected health information (PHI) in accordance with national and local privacy regulations.
3. Definite diagnosis of relapsing-onset MS according to the revised McDonald 2017 criteria
4. All relapsing-onset MS patients treated with one of the first-line treatments: any of the interferons, glatiramer acetate, dimethylfumarate, teriflunomide
5. Complete absence of inflammatory activity (no objectively defined and confirmed relapses, no significant number (2 or more) of new-T2 lesions and no contrast-enhancing lesions) for 5 consecutive years under first-line treatment
6. Daily use of a smartphone with Android (5.0 or higher) or iOS (10 or higher) operating system.

4.3 Exclusion criteria

A potential subject who meets any of the following criteria will be excluded from participation in this study:

1. A switch between first-line disease modifying therapy over two years prior to inclusion, in case the switch has been due to in effectivity of the first DMT. In case the switch has been due to side-effects or by a personal preference of the patient (such as the wish to switch to oral therapies), this is not considered as an exclusion criterium.
2. Women who want to discontinue medication because of a pregnancy wish and women who are pregnant or expect to become pregnant during the study period
3. Patients that have previously used interferon-beta and have been tested positive for neutralizing antibodies (NAbs). This is determined by measuring MxA-bioactivity and is a test that is part of routine follow-up in patients that use interferon-beta. The reason for this is that development of NAbs has been shown to affect interferon-beta treatment efficacy.

4.4 Sample size calculation

The stability of patients in the continuation group is estimated to be at least 97,5%. Based on a non-inferiority margin of 7,5%, a preliminary power-calculation based on the non-inferiority principle was performed (PASS v12, one-sided Z-test (unpooled), significance level 0.05) and showed a necessary sample size of 54 per group to achieve 80% power. Taking 20% drop out into consideration, the total sample size needed for this study is 130.

The applications MS Sherpa and Neurokeys will be added to collect digital biomarkers regarding (return of) inflammatory disease activity. Based on previous experience, our estimate is that the current sample-size can provide some conclusive trends on the association between digital biomarkers and (return of) inflammatory disease activity.

5. TREATMENT OF SUBJECTS

5.1 Investigational product/treatment

The intervention consists of the discontinuation of the previously used DMT (either interferons, glatiramer acetate, dimethylfumarate or teriflunomide) Based on the pharmacological profile of the abovementioned drugs that are eligible for inclusion, there is no need for a tapering of dosage before complete discontinuation.

5.2 Use of co-intervention

During the intervention period patients are asked not to participate in any other scientific studies. Patients are allowed to use all types of co-medication, except for immunomodulating drugs such as prescribed for the treatment of multiple sclerosis and/or other auto-immune diseases.

5.3 Escape medication

When disease activity emerges, patients are treated according to the current standard of care (including intravenous methylprednisolone if deemed necessary) and disease modifying treatment will be reinitiated. Unscheduled visits including an MRI-scan are planned for each patient with any new neurological complaints, as is standard clinical procedure.

6. INVESTIGATIONAL PRODUCT

N/A

7. NON-INVESTIGATIONAL PRODUCT

MS Sherpa and Neurokeys are CE-certified medical devices. See for the relevant details appendices “D6 - Aanvullende productgegevens MS sherpa” and “D6 – Aanvullende productgegevens Neurokeys”.

8. METHODS

8.1 Study parameters/endpoints

8.1.1 Main study parameter/endpoint

The primary endpoint is number of patients with return of inflammatory disease activity after 2 years based on: a clinically confirmed relapse (defined according to the definition most often used in MS phase-III trials: the onset of new or recurrent symptoms that last > 24 hours, that are accompanied by new objective abnormalities on a neurological examination and that are not explained by non-MS processes such as fever, infection, severe stress or

drug toxicity (Gold et al NEJM 2012)) , or any emerging subclinical disease activity proven to be due to active disease/new inflammation (defined as 3 or more lesions on T2—weighted images or 2 or more gadolinium enhancing lesions on T1-weighted post-contrast MRI) in the discontinuation group.

8.1.2 Secondary study parameters/endpoints (if applicable)

Secondary end-points are

- Changes in neurological functioning
    - EDSS change (Including individual functional systems)
    - MSFC changes
        - Timed 25-foot Walk (T25fW)
        - 9-Hole Peg Test (9HPT)
        - Symbol digits modalities test (SDMT)
- Individual MRI-parameters
    - T1 post-contrast lesion numbers and volumes
    - T2 lesion numbers and volumes
    - Whole brain volume
    - Normalized white matter volume
    - Grey matter volume
- Changes in quality of life measurements
    - Multiple Sclerosis Impact Scale (MSIS-29)
    - Short Form health survey (SF-36)
    - Multiple Sclerosis Self-Efficacy scale (MSSE)
    - Checklist Individual Strength (CIS20r)
    - Treatment Satisfaction Questionnaire for Medication (TSQM)
- Cost measurements
    - EuroQol 5 dimensions questionnaire (EQ-5D-5L)
    - Medical consumption questionnaire (iMCQ)
    - Productivity costs questionnaire (iPCQ)
- Changes in biomarker measurements
    - Neurofilament levels
- Changes in digital biomarkers using the NeuroKeys (CE) and MS sherpa (CE) mobile applications that measure:
    - Walking test (2-minute walking test)
    - Cognition test (similar to SDMT)
    - MS sherpa questionnaires (including fatigue)

- o Keystroke data

8.1.3 Other study parameters (if applicable)

Vitamin D, smoking high body-mass index (BMI) are considered as potential confounders. Therefore, vitamin D will be determined and patients will be asked about smoking behaviour during every visit. To avoid any potential bias, we will also collect data (if present in the individual patients) on disease activity prior to the initiation of DMT, such date of diagnosis, time from first symptoms to diagnosis, EDSS scores/MS severity at the moment of DMT initiation.

8.2 Randomisation, blinding and treatment allocation

Since MS affects 2-2.5x as many women as men, we expect to include more women than men. A randomization algorithm will be included to match both groups for sex and age to exclude potential bias.

Outcome measurements will be performed by assessors who are blind to the intervention assignment. For reasons of consistency and to exclude possible bias, all scans will be centrally reviewed in VUmc by a radiologist blinded to allocation to the intervention group. In the current set-up of the trial patients and their treating neurologists are unblinded to the randomization group. The currently available first-line disease modifying treatments consist of the various (peg)interferons, glatiramer acetate, teriflunomide and dimethylfumarate. It comes in a total of more 10 different forms with different packaging, different modes of injection (some subcutaneously, some intramuscular and some oral) and different frequencies of intake (ranging from twice daily to once every two weeks). We have set up the trial so that it is rater-blinded; all MRI-scan and clinical evaluations (such as the neurological examination) will be performed blinded to “treatment” allocation. The primary and majority of secondary outcome measures are derived from these blinded assessments. This approach is frequently chosen in MS research (even in the phase-III trials) for 2 very important reasons. Due to the very distinct nature of side-effects (flushing, gastro-intestinal problems, flu-like symptoms), patients know when they receive placebo instead of the actual active compound. The invasive nature of receiving placebo-injections would greatly enhance the possibility of patients not participating. In addition, we believe it is too costly to create a placebo-control for each of the 10 different forms of medication.

8.3 Study procedures

No study procedures will take place before having obtained informed consent which will be gained following current METc/CCMO standards.

Follow-up frequency

Patients in the continuation group are expected to remain stable throughout the study period based on their proven stable status for 5 consecutive years prior to enrolment. The minimum evaluation that must take place should have a frequency according to the current standard of care (yearly evaluation of every patient treated with immunomodulating therapy; i.e. baseline (BL), 12 and 24 months). Timely recognition of recurrence of any (subclinical) disease activity in the discontinuation group is secured by more frequent clinical and radiological assessment during extra routine study visits at 3, 6 and 18 months. For optimal comparability and to overcome potential bias , both groups will be followed with a complete assessment including MRI at BL, 3, 6, 12, 18 and 24 moths. For the patients in both groups mobile applications MS sherpa and Neurokeys will be installed. With the MS sherpa application, patients will be asked to perform tests every two weeks during the 24 months of follow-up. The Neurokeys application will collect data on the background of the standard use of mobile phones during this time period.

Clinical evaluation

Duration: 1 hour

Clinical evaluation will consist of a careful medical history: current and past medication, adverse events, number of intravenous methylprednisolone treatments, number of relapses, date of last relapse and signs of symptom progression. A relapse is defined according to the definition most often used in MS phase-III trials: the onset of new or recurrent symptoms that last > 24 hours, that are accompanied by new objective abnormalities on a neurological examination and that are not explained by non-MS processes such as fever, infection, severe stress or drug toxicity[21]. Furthermore, the Expanded Disability Status Scale (EDSS) and Multiple Sclerosis Functional Composite (MSFC)-measurements will be performed, consisting of the Timed 25-foot Walk (T25fW), 9-Hole Peg Test (9HPT) and the symbol digits modalities test (SDMT). This will be done by a blinded investigator.

Radiological evaluation

Duration: 45 min

Radiological evaluation will consist of repeated brain MRI investigations that consist of conventional pre- and post-contrast (T2-weighted, T1-weighted pre and post contrast, FLAIR) images. All scan protocols are available in general and academic hospitals since they form the basis on which MS is diagnosed and treatment is monitored. Although it is expected that new inflammatory lesions can be captured by repeated T2-weighted/FLAIR MRI-scans, a gadolinium-scan is included to not miss any contrast enhancement in previously present

lesions. A 3DT1 and 3DFLAIR image will also be made for atrophy measurements. For reasons of consistency and to exclude possible bias, all scans will be centrally reviewed in VUmc by a radiologist blinded to allocation to the intervention group.

Questionnaires

Duration: 1 hour

For the evaluation of disease burden and MS related symptoms, we will use the validated and in clinical studies often used Multiple Sclerosis Impact Scale (MSIS-29)[36] Short Form health survey (SF-36)[37] and CIS20r[38]. The perceived impact of treatment, convenience, satisfaction and side-effects will be monitored using the Treatment Satisfaction Questionnaire for Medication (TSQM)[39]. For the evaluation of health related quality of life (HRQoL) and the link between symptoms HRQoL and costs, we use the EDSS for the objective measurement of changes in neurological functioning (which has a clear relation to HRQoL assessed as utility and costs[40]) and the number of patients with return of inflammatory activity. We also use the EQ5D-5L) for cost-utility analysis[41]. Furthermore, we will ask patients to keep a diary describing changes in healthcare consumption (which will be defined per item in a questionnaire (such as hospitalisations, consultations with doctors, use of care at home, use of specialized transportation etc). Lastly patients will be asked for their employment situation and short-term and long-term sick absence because of MS (or MS related treatment) using the iMCQ and iPCQ questionnaires[42]. Questionnaires are filled in digitally either at home or during the hospital visit. Help of a carer or the study-nurse is allowed in cases of the inability using a computer.

Samples

Duration: 15 minutes

Blood collection will take place in both patient groups at every visit. It will consist blood collection for the purpose of biobanking and for diagnostics in the case of any –unforeseen- clinical events. Furthermore, we will retrospectively measure neurofilament light in serum using Simoa.

All participants will visit the hospital for 6 times over a time frame of 2 years. Each of these visits that take place will take approximately 2,5-3 hours (clinical assessment and MRI-scan). Also 1-hour questionnaires which can be completed at home digitally or at the hospital on paper will take place before/during each visit.

All samples will be collected, processed and stored according to the Standard Operating Procedures (SOP's) as described in the Parelsnoer Biobankprotocol version 8.0. (15)

Samples will be stored at the Biobank VUmc. To ensure patient privacy all samples will be coded. A peripheral blood sample will be collected, in total 8 tubes of blood will be drawn (5x EDTA 4 ml for plasma, cells and DNA isolation, 2x serum 5 ml, 1x PAXgene tube 2 ml), adding up to a total volume of 32 ml. Blood will be centrifuged, divided in aliquots of 0.5 ml and then stored at -80 °C.

Mobile applications measurements
Duration: 5 minutes
Frequency: once every two weeks.

For eligible patients (based on regular smartphone use), mobile applications MS-Sherpa and Neurokeys will be installed on their smartphones and data will be collected via these applications.

MS sherpa mobile application
Via the MS sherpa application the patient will be asked to perform walking tests, cognition tests and the MS sherpa questionnaire (including fatigue).

- Cognitive task: similar to the SDMT, the participant is requested to assign numbers to corresponding symbols according to a specific displayed coding.
- Motor task: 2-minute walk test (2MWT): the participant is requested to walk (either unassisted or with a walking aid) for two minutes. The walking distance is measured through the location data. Patients who are unable to walk for two minutes will not be requested to do the 2MWT.
- MS sherpa questionnaire: patient reported outcomes on Likert scales (amongst others about fatigue and the impact of MS on daily activities).

These tests can be completed in approximately 5 minutes and patients will be asked to complete these tests once every two weeks.

NeuroKeys mobile application
NeuroKeys replaces the standard keyboard of the patient's smartphone. Data is collected from regular use of the keyboard, and no additional action from patients is needed. After 24 hours of inactivity of the keyboard of NeuroKeys, either intentionally or unintentionally, a push notification is automatically send utilizing Amazon Simple Notification Service (SNS). NeuroKeys will collect keystroke data general profile information (gender, year of birth, and MS type), and metadata (iOS/Android version and NeuroKeys version). The data will be

collected from the phone continuously in a retrospective fashion, when the keyboard is activated the data from the previous keyboard typing session is sent.

- Keystroke data: the start of a message is defined as the keyboard flipping up and the end of a message is marked when the keyboard flips down. Specific keys will be logged and timestamped to be able to accurately calculate parameters expected to be associated to fatigue. The keys logged are: delete or backspace key, dot key, space bar, semicolon, colon, parentheses, capitalized characters, numbers and punctuation marks denouncing the ending of a sentence. All numbers and all punctuation marks will be logged as the same number or punctuation event (e.g. we do not make a distinction between the number 3 and 8 or the comma and dollar sign). Parameters such as word count, amount of words comprised of six characters or more and latency between key presses will be calculated on the basis of the logged keys. In addition to ASCII keys, the unicodes of emojis will also be logged.
- Sensor data: Data from the location sensor, ambient light sensor, gyroscope, motion and accelerometers of the smartphone will be collected when the keyboard is in use. With the location sensor (longitude and latitude), the keystroke data can be combined to open source databases (e.g. weather data from the Royal Netherlands Meteorological Institute, KNMI) to examine the influence of external factors (Davis et al. 2010). Existing and future open source databases provided by public or governmental institutes can be accessed for this purpose. Ambient light sensor can detect environmental brightness which could impact typing behaviour. Kinematic sensors (gyroscope, motion and accelerometers) may be used to infer posture of the user (Lamonaca et al. 2015).

8.4 Withdrawal of individual subjects

Subjects can leave the study at any time for any reason if they wish to do so without any consequences. The investigator can decide to withdraw a subject from the study for urgent medical reasons.

8.4.1 Specific criteria for withdrawal (if applicable)

There are no specific criteria for withdrawal from the study.

8.5 Replacement of individual subjects after withdrawal

There will be no replacement of individual subjects after withdrawal.

8.6 Follow-up of subjects withdrawn from treatment

If a patient is withdrawn from the study, we will still perform follow-up measurements in case the patient is willing and able to cooperate.

8.7 Premature termination of the study

The procedure in case of premature termination of the study is described in section 10.4 and 12.5.

9. SAFETY REPORTING

9.1 Temporary halt for reasons of subject safety

In accordance to section 10, subsection 4, of the WMO, the sponsor will suspend the study if there is sufficient ground that continuation of the study will jeopardise subject health or safety. The sponsor will notify the accredited METC without undue delay of a temporary halt including the reason for such an action. The study will be suspended pending a further positive decision by the accredited METC. The investigator will take care that all subjects are kept informed.
More information about temporary halt for reasons of subject safety is provided in section 10.4 and 12.5.

9.2 AEs, SAEs and SUSARs

9.2.1 Adverse events (AEs)

Adverse events are defined as any undesirable experience occurring to a subject during the study, whether or not considered related to the experimental intervention. All adverse events that are reported spontaneously by the subject or observed by the investigator or his staff and that are relevant to the study will be recorded.

Adverse Device Effect (ADE)

An ADE is an adverse event related to the use of an investigational medical device. This includes any adverse event resulting from insufficiencies or inadequacies in the instructions of use, the deployment, the installation, the operation, or any malfunction of the investigational medical device. This also includes any event that is a result of a use error or intentional misuse.

9.2.2 Serious adverse events (SAEs)

A serious adverse event is any untoward medical occurrence or effect that

- results in death;
- is life threatening (at the time of the event);
- requires hospitalisation or prolongation of existing inpatients' hospitalisation;
- results in persistent or significant disability or incapacity;
- is a congenital anomaly or birth defect; or
- any other important medical event that did not result in any of the outcomes listed above due to medical or surgical intervention but could have been based upon appropriate judgement by the investigator.

A SADE is an ADE that has resulted in any of the consequences characteristic of a serious adverse event.

The investigator will report all SAEs and SADEs to the sponsor without undue delay after obtaining knowledge of the *events*. The sponsor will report the SAEs through the web portal *ToetsingOnline* to the accredited METC that approved the protocol, within 7 days of first knowledge for SAEs that result in death or are life threatening followed by a period of maximum of 8 days to complete the initial preliminary report. All other SAEs will be reported within a period of maximum 15 days after the sponsor has first knowledge of the serious adverse events.

9.2.3 Suspected unexpected serious adverse reactions (SUSARs)

This section is not applicable since this study does not investigational medicinal products.

9.3 Annual safety report

This section is not applicable since this study does not investigational medicinal products.

9.4 Follow-up of adverse events

All AEs will be followed until they have abated, or until a stable situation has been reached. Depending on the event, follow up may require additional tests or medical procedures as indicated, and/or referral to the general physician or a medical specialist.
SAEs need to be reported until the end of study, as defined in the protocol in section 9.2.2.

9.5 Data Safety Monitoring Board (DSMB)

For optimal safety we will appoint an independent data safety monitoring board (DSMB)which will monitor trial data on a regular basis. The aim of the committee is to safeguard the interests of trial participants and assess the safety of the discontinuation of therapy during the trial. The specific role of the DSMB consists of monitoring evidence for harm due to the intervention (discontinuing medication). The DSMB may advice to terminate the trial prematurely if disease activity exceeds above mentioned thresholds (see section 10.4 for interim analyses).

The DSMB consists of 4 members who do not have conflict of interest with the sponsor or the study. In addition to the permanent members of the DSMB two external members are added to the DSMB with expertise in the relevant field of research (yet to be determined).

The DSMB will conduct interim analyses in a predetermined manner and at predetermined times (see section 10.4), to see whether the relationship between clinical benefit and burden remains acceptable to the subject during the study. After each interim analysis, the DSMB reports to the study coordinator, with reports to the METc and the study sponsor, i.e. the Board of Directors (Raad van Bestuur). The DSMB can give advice to continue, change or stop (parts of) the study. The DSMB will also ensure the quality and safety of research in the participating centers.

10. STATISTICAL ANALYSIS

All data is quantitative and will be presented in tables and graphs. Baseline data will be collected to detect any potential differences between the two investigated groups for which we have not corrected with the randomization procedure (sex and age). Possible other confounders include smoking habits, vitamin D levels but also previous disease course in terms of differences in disease duration, number of relapses prior to stability, years of use of treatment.

The primary endpoint is the number of patients with return of inflammatory disease activity after 2 years based on: a clinically confirmed relapse or any emerging subclinical disease activity proven to be due to active disease/new inflammation (defined as 3 or more lesions on T2—weighted images or 2 or more gadolinium enhancing lesions on T1-weighted post-contrast MRI) in the discontinuation group. Secondary end-points are: 1. Changes in neurological functioning (EDSS/MSFC changes including individual functional systems and MSFC subtests), 2. Individual MRI parameters (T2 and T1 post-contrast lesion numbers), 3. changes in quality of life measurements (SF-36, CIS20r, TSQM, EQ5D-5L, iMCQ and iPCQ) and 4. changes in biomarker measurements (neurofilament levels).

In the case of confirmation of our hypothesis (discontinuing medication after >5 years without evidence of inflammatory disease activity does not result in return of inflammatory disease activity), we will extend the trial with a follow-up of two years where the group that continued their treatment gets offered the possibility to discontinue under similar safety measures (with similar follow-up frequencies and endpoints as the primary trial) and including the possibility to use MS sherpa and Neurokeys.

10.1 Primary study parameter(s)

For the primary endpoint, the return of inflammatory disease activity after 2 years, a2x2 contingency table will be used to estimate the risk difference for the return of inflammatory disease activity after 2 years (yes/no) between the two groups. The risk difference will be calculated for discontinuation relative to continuation. If the lower bound of the corresponding two-sided 90% confidence interval exceeds -7.5% we conclude non-inferiority of discontinuing medication For the primary endpoint both a per protocol analysis and a intention-to-treat analysis will be performed.

The primary endpoint is the number of patients with return of inflammatory disease activity after 2 years based on: a clinically confirmed relapse or any emerging subclinical disease

activity proven to be due to active disease/new inflammation (defined as 3 or more lesions on T2—weighted images or 2 or more gadolinium enhancing lesions on T1-weighted post-contrast MRI) in the discontinuation group.

A relapse is defined according to the definition most often used in MS phase-III trials: the onset of new or recurrent symptoms that last > 24 hours, that are accompanied by new objective abnormalities on a neurological examination and that are not explained by non-MS processes such as fever, infection, severe stress or drug toxicity[21]

Furthermore, a survival analysis (with an intention-to-treat approach) regarding the time to return of inflammatory activity will be included.

10.2 Secondary study parameter(s)

For all the secondary endpoints correlation and regression analysis (either linear of logistic, depending on the type of variable) will be performed correcting for possible confounders. On the app data, classification analyses and machine learning models will be used next to the more conventional analyses.

10.3 Other study parameters

N/A

10.4 Interim analysis

We will build in a safety-strategy (go-no-go strategy) during the first 1,5 year of the study to control for emerging disease activity (and patients safety) in the discontinuation group. This implicates that after every visit, patients are evaluated for (sub)clinical and radiological disease activity. For this safety strategy, we will make a distinction between patients <55 years old and patients ≥55 years old based on mechanistic differences of recurrent inflammation in both groups. In case of an objectified MS relapse, or any emerging subclinical disease activity proven to be due to active disease/new inflammation (defined as 3 or more lesions on T2—weighted images of 2 or more gadolinium enhancing lesions on T1-weighted post-contrast MRI) in the discontinuation group in:

- ≥5% of patients in the first 6 months of the study in either one of both age groups (<55 years old or ≥55 years old) or;
- ≥10% of either one of both age groups within the first 12 months of the study, or;
- ≥15% of either one of both age groups within the first 18 months of follow-up,

we may prematurely stop the study for a specific age group. Distribution within a specific age group will determine whether the entire age group will be advised to restart DMT or that restart may be restricted to a specific subgroup. In that case, we will continue the study for the other patients according to the protocol. In case of a premature stop of the trial for a specific age group of patients that discontinued their DMT, we will follow the patient group for which the trial was discontinued in an open label extension study with follow-up frequencies and assessments similar to the trial.

For optimal safety the DSMB will monitor the decision making on premature termination every 3 months. The DSMB may advice to terminate the trial prematurely if disease activity exceeds above mentioned thresholds. See section 9.5 for a more detailed description of the DSMB.

The procedure in case of premature termination of the study is described in section 12.5.

10.5 Cost-analysis

Alongside this trial, we will conduct a cost-effectiveness analysis and a cost-utility analysis over a 2-year follow-up. These will be performed in accordance with the recommendations of the Dutch guideline for health economic evaluations. For the cost-effectiveness analysis, the return of inflammatory disease activity (either relapse or new of enlarging lesions) will serve as the effectiveness measure. The cost-utility analysis will focus on quality of life measured with the EQ-5D-5L, which is often used in MS research. Quality-adjusted life-years (QALYs) will be calculated by multiplying the utility scores belonging to a health state by the amount of time spent in this health state using linear interpolation between time points.

In both analyses, costs will be measured from a societal perspective including health-care costs (such as the costs for a year of DMT, costs for the extra surveillance including additional medical tests as MRI, costs for visits to other medical doctors etc), direct nonmedical costs (costs that patients make for travelling to and from the hospital, private payments for extra health-care consumption etc) and indirect nonmedical costs (costs due to loss of production and short or long-term sick absence). The latter is important as previous research has shown that productivity losses are an important cost driver in severe MS. Health-care costs and direct nonmedical costs will be measured using the iMTA Medical Consumption Questionnaire (iMCQ) at 3 months, 6, months, 12 months, 18 months and 24 months. The iMCQ measures the health-care costs in the last three months. As these patients are assumed to be stable regarding their disease progression, these follow-up

moments will provide an adequate estimation of their health-care use. Primary and secondary health-care costs will be valued using Dutch standard costs. If unavailable, tariffs or costs reported by the literature will be used. Medication will be valued using www.medicijnkosten.nl whereas informal care will be valued based on the costs of household care as reported by the Centraal Administratie Kantoor.
For indirect nonmedical costs, patients will be asked for their employment situation and both short-term and long-term sick absence because of MS (or MS related treatment) using the iMTA Productivity Cost Questionnaire (iPCQ) at 3 months, 6, months, 12 months, 18 months and 24 months. The iPCQ measures productivity loss in the last four weeks which will give an adequate overview of the productivity losses between each time point as these patients are assumed to be stable. Costs of absenteeism from paid work will be calculated according to both the human capital and friction cost approach. Costs of presenteeism will be calculated by asking participants how many working hours should have been replaced due to less productivity at work. Lost productivity due to either absenteeism or presenteeism will be valued using the mean age-, and sex specific income of the Dutch population. Costs of productivity losses due to absenteeism from unpaid work and informal care will be calculated using the standard wage of a professional housekeeper. All costs will be indexed to the year at which the trial ended. Missing data on costs and effects will be imputed using multiple imputations. In addition, costs and effects will both be discounted using a 3% discount rate.

For the cost-effectiveness analysis, we will calculate incremental cost-effectiveness ratios (ICERs) which is defined as the difference in mean costs divided by the difference in mean effects between the treatment continuation group and the treatment discontinuation group. For the cost-utility analysis, we will calculate incremental cost-utility ratios (ICURs) which is the difference in mean costs divided by the difference in mean QALYs. Bootstrapping with 5,000 replications will be used to estimate 95% credibility intervals around the ICERs and ICURs. The bootstrapped cost-effect pairs will be plotted on a cost-effectiveness plane and used to estimate cost-effectiveness acceptability curves (CEACs). CEACs show the probability that the intervention is cost-effective in comparison with the control treatment for a range of ceiling ratios. The ceiling ratio is defined as the willingness-to-pay, which is the amount of money society is willing to pay to gain one unit of effect.

In a sensitivity analysis, we will repeat all analyses using a healthcare payer perspective. In this analysis, only direct healthcare costs will be included. In addition, we will conduct subgroup analysis in which we will stratify individuals based on the presence of blood-based markers predictive for return of inflammatory disease activity.

Furthermore, we will conduct a budget impact analysis. A budget impact analysis (BIA) focuses on the expected changes in the expenditure of a health care system after the adoption of a new standard of care. In this BIA, we aim to estimate the future yearly budget impact of discontinuation of first line medication in patients with long-term stable relapsing-onset from a Dutch perspective. The BIA will be performed according to the BIA framework of the International Society for Pharmacoeconomics and Outcomes Research (ISPOR). This framework consists of several standard aspects: target population, scenario distribution based on hospital types, resource utilisation, costs per unit, total costs, and sensitivity analyses.

11. ETHICAL CONSIDERATIONS

11.1 Regulation statement

The study will be conducted according to the principles of the Declaration of Helsinki (World Medical Association, 2013, Brazil) and in accordance with the Medical Research Involving Human Subjects Act (WMO) and the Good Clinical Practice guidelines.

11.2 Recruitment and consent

Patients will be informed about the study in different ways. Patients can be notified by their treating doctor during outpatient consultations at the VUmc or one of the other participating centres. In addition, a notification will be placed on the website of the MS Centre Amsterdam and the websites of the MS Vereniging Nederland (MSVN) and MS Web with information about the study. Patients can then discuss potential interest in the study with their treating neurologist. Inclusion is possible in one of the participating centers. Potential participants who express their wish to participate will receive additional information on paper about the purpose, intervention, duration and content of the study. They will also receive an informed consent form with careful and extensive information about the possible risks (according to local ethical review board requirements). In case there are any questions about the study, the study coordinator can be contacted. Moreover, an independent doctor (dr. Pijnenburg) can be contacted for additional questions.

Inclusion will take place after the written informed consent form has been returned to the trial coordinator. A copy of the informed consent form will be given to the participant and to the responsible physician in one of the participating centers in case the patient is recruited through one of the centers outside the VUmc. This consent can be revoked at any time without citing reasons. Patients will be given a minimum of 2 weeks to consider their decision. The maximum time will be as long as the inclusion of patients is ongoing and inclusion and exclusion criteria are met.

11.3 Objection by minors or incapacitated subjects (if applicable)

Not applicable, all participants will be adult and legally competent

11.4 Benefits and risks assessment, group relatedness

In the non-intervention group (continuation group) participants are expected to remain stable throughout the study period based on their proven stable status for 5 consecutive years prior to enrolment. Therefore, this group will face no potential risks and no direct benefit other than the usual medical care. The potential value of the outcome of the research will outweigh the

burden of participation for the study. Also, patients in the continuation group are offered the possibility to discontinue their medication under similar surveillance measures as the discontinuation group after 24-month follow-up period has ended.

The data on the discontinuation of therapy in long-term inflammatory stable MS patients is reassuring. One large retrospective cohort study has shown in 1200 patients that stable RRMS patients who stop treatment don't have an increased relapse rate compared to patients who continue treatment[25]. There was a slightly higher disability progression of the discontinuation group, presumably based on the discontinuation of treatment in patients with progressive MS. Also, there seems to be no rebound inflammation after discontinuation of therapy in progressive MS, and a similar relapse rate as the years prior to discontinuation[26]. Lastly, one study has showed that patients 45 years or older, or patients with a DMT intake of 4 or more years without evidence of clinical or radiological disease activity showed a high likelihood of remaining relapse-free after discontinuation and absence of contrast enhancing lesions[27]

11.5 Compensation for injury

According to article 7 from the 'Wet medisch-wetenschappelijk onderzoek met mensen' (Staatsblad 1998, 161) an insurance is obtained by the VUmc. In case of injury or death of the participants because of the study, this insurance will compensate for injury or cover the cost caused by death or injury from the participants. The insurance is obtained by the Onderlinge Waarborgmaatschappij Centramed b.a., Postbus 7374, 2701 AJ Zoetermeer. The insurance company and the insurance accede to the decree mandatory insurance for 'medisch-wetenschappelijk onderzoek met mensen (Staatsblad 2003, 266). Written information about the insurance will be provided for the participants.

11.6 Incentives (if applicable)

According to the current standard of care, the evaluation of every patient treated with immunomodulating therapy is at baseline, 12 and 24 months. Since visits are also scheduled at 3, 6 and 18 months, travel expenses and parking costs for these extra visits will be compensated. Participants will be compensated for their time and effort for study participation: they will receive a gift certificate of €25 upon study completion.

12. ADMINISTRATIVE ASPECTS, MONITORING AND PUBLICATION

12.1 Handling and storage of data and documents

Data will be handled confidentially . After collection, all data will be correctly labeled and securely stored. A subject identification code (SIC) will be used to link data to the subject. The SIC will consist of numbers and will not be based on the patient initials and birth-date. The key to the code will be kept separately from the coded data. The only people who have access to this code will be the principal investigator, the coordinating investigator and the corresponding investigator. No other people will have access to the link information. Great care will be taken to ensure that there is no link between SIC and information on which an individual can be identified. The handling of personal data in the database complies with the General Data Protection Regulation (De Algemene Verordening Gegevensbescherming) Potential data exchange with other countries will only take place after consent of the patient and handling of data will comply with the General Data Protection Regulation. The procedure for handling data includes data encryption, coding, secure storage, establishing limited access or varying levels of access to the biobank, removing identifying information from bio specimens and data. The infrastructure will consist of both hardware and software components, to prevent unauthorized access to databases.

An electronic case report form (CRF) will be developed to document the data collected in the study. This database will include demographic and patients characteristics (without birth date) and all outcomes of the study measures. Other investigators can request permission to get access to (a part) of this database for the purpose of research only, and only when the principal investigator gives permission. These investigators will not get access to the separate database which includes the participants' names, other identifiers and the SIC. All data will be stored on a computer protected with a password on the VUmc computer network. And access to the database will also be secured by a code. Only the trial coordinator and the principal investigator will know the code that gives access to the database with the link information.

After finalizing the study, the originals of all source documents will be stored for a period of 15 years in a locked room. Data that is collected and stored for the Biobank, will be stored for a period of 50 years. This period of storage has been determined to ensure that a follow-up study might be possible. In case of a follow-up study, a new protocol will be submitted to the METC and participants will have to sign a new informed consent form. Importantly, participants will only be approached for a follow-up study if they have indicated on the

informed consent form of the current study that they can be approached for a follow-up study.

The collection of data for medical research in the Netherlands is subject to the Personal Data Protection Act and in particular to the Medical Treatment Contracts Act.

Data storage Neurokeys

Data collected by NeuroKeys will be stored using Amazon AWS S3. There will be no identifiable information in this database, all keystroke and sensor data are logged with ID numbers only. A separate database, Amazon RDS (SQL server), will be used to store the verification code and personal information such as gender and year of birth, which can be used to send users push notifications. Both Amazon AWS S3 and Amazon RDS databases are located in Frankfurt, Germany, and are ISO 27001/27017/27018 compliant. All data is AES-256 encrypted in transit and at rest, a tokenization approach is used in which a sensitive data element is replaced by a non-sensitive equivalent and send by using a secure SSL link. Decryption keys are stored in a private encrypted environment. For iOS users, each time the NeuroKeys' keyboard is 'activated' (i.e. a new message is started), data of the previous keyboard session is uploaded to the database. Data of only one message is saved on the mobile phone, until a new message has started. For Android users data is uploaded to the database approximately every 4 hours.

Data storage MS sherpa

Data collected by MS sherpa will be stored using MongoDB Atlas, whose infrastructure runs on top of Amazon Web Services (ISO 27001/27017/27018 compliant) in Dublin, Ireland. General profile information (such as gender, age, length, weight, and e-mail address) is directly saved on AWS servers, in Dublin and AWS S3 in Frankfurt, Germany. Auth0 is used for user authentication and authorisation (ISO 27001/27018 compliant) and its EU office is based in London, UK. MongoDB Atlas and Auth0 achieved key compliance controls and objectives, as demonstrated by the completion of a Type 1 SOC 2 Report: Security. MongoDB, Inc. and Auth0 are also certified under the EU-US Privacy Shield. Data gathered via MS sherpa will be transferred to the database immediately after the data has been collected.

12.2 Monitoring and Quality Assurance

An independent monitor, the Clinical Research Bureau (CRB) of the VUmc, will monitor the proposed study according to Good Clinical Practice (GCP). For a selection of candidates

Informed consent is to be checked by the CRB. Besides that, source data verification is performed during the onsite monitoring. The conformity of the data used for analysis and the information in the patient files will be checked by the CRB. The intensity of the verification will be related to the risk arisen by the research. Inclusion and exclusion criteria will be checked as well as the main outcome measures. The CRB will check if the (S)AE's and SUSAR's are reported conforming the schedule as required by laws and regulations.

The quality assurance team under the leadership of a quality assurance manager (QAM) is responsible for providing an effective and efficient quality assurance system and counsel for the clinical research sites. In this quality assurance system, the QAM is responsible for ensuring appropriate global and affiliate-specific quality documents are developed and tracked, making sure they maintain an up-to-date overall inventory of all quality documents. Furthermore, the QAM and its team are responsible for ensuring all personnel involved in the clinical trial are properly qualified and trained for the job roles for which they are responsible. They are responsible for giving the personnel trainings and constantly assessing further opportunities for education and additional training. The quality assurance team is also responsible for checking compliance with the protocol, SOPs, GCP, and/or applicable regulatory requirement(s) and checking of the quality in all stages of data handling to ensure that all data are reliable and have been processed correctly. Moreover, the quality assurance team is responsible for auditing the various investigational sites.
If noncompliance with the protocol, SOPs, GCP, and/or applicable regulatory requirement(s) by and investigator/institution, or by member(s) or the sponsor's staff is detected during a quality assurance activity or audit, it is the responsibility of the QAM to report this to the trial's sponsor and principal investigator.

Quality assurance procedures:

Quality assurance is the systematic and independent examination of all clinical trial-related activities and documentations. The quality assurance procedure focuses on clinical investigator audits and audits of clinical trial documentation.

1. Document audits:

During the document audits, the quality assurance team oversees the documents that are generated before, during or at the end of the conduct of the clinical trial. For each document, a checklist is developed based on the relevant regulatory and organizational standards and SOPs. The aim of the audits is to ensure that the information and data in the documents are

complete, clear, reliable and consistent. Documents reviewed in the document audits include the clinical study protocol, the investigator's brochure and the clinical study report.

2. Clinical investigator audits:

The clinical investigator audits concern audits of the different research sites of the trial. They are performed to assess the site's regulatory compliance and clinical data quality (including adherence to the protocol). Paragraph 3 will address the procedures concerning these audits.

12.3 Amendments

Amendments are changes made to the research after a favourable opinion by the accredited METC has been given. All amendments will be notified to the METC that gave a favourable opinion. All amendments will be notified to the METC and to the competent authority.

12.4 Annual progress report

The investigator will submit a summary of the progress of the trial to the accredited METC once a year. Information will be provided on the date of inclusion of the first subject, numbers of subjects included and numbers of subjects that have completed the trial, serious adverse events/ serious adverse reactions, other problems, and amendments. The METC will also be informed on the start and end date of the trial.

12.5 Temporary halt and (prematurely) end of study report

The investigator/sponsor will notify the accredited METC of the end of the study within a period of 8 weeks. The end of the study is defined as the last patient's last visit.
The sponsor will notify the METC immediately of a temporary halt of the study, including the reason of such an action.
In case the study is ended prematurely, the sponsor will notify the accredited METC within 15 days, including the reasons for the premature termination.
Within one year after the end of the study, the investigator/sponsor will submit a final study report with the results of the study, including any publications/abstracts of the study, to the accredited METC.

12.6 Public disclosure and publication policy

This study will be registered in het Nederlands Trial Register (NTR) http://www.trialregister.nl and www.clinicaltrials.gov. Publication will be in accordance with the basic principles of

CCMO statement on publication policy. The results will be presented at (inter)national scientific meetings. The results will be published in a medical scientific journal. In none of the publication forms, participant identity will be disclosed.

13. STRUCTURED RISK ANALYSIS

13.1 Potential issues of concern

Paragraph 13.1 is not applicable.

13.2 Synthesis

The intervention in this study is the discontinuation of previously used DMT. No new products or agents are administered, nor will there be any dosage adjustments in the group that will continue their therapy. The specific DMT’s that patients use prior to discontinuation, and that are used in the control group are all registered with the authorities and widely used for this specific indication. Although previous studies suggest that the risk of return of inflammatory activity after discontinuing DMT will be low in long-term stable RRMS patients (as also described in section 1), this is the main risk of the intervention. To monitor return of inflammatory activity, a safety strategy is built in, which is described in section 10.4. If safety criteria are exceeded, the study will be discontinued and DMT’s will be reinitiated (in one patient group or in all patients, see section 10.4). A DSMB is appointed that will monitor the decision making on premature termination every 3 months (section 9.5).

14. REFERENCES

1 Goodin, D. S. The epidemiology of multiple sclerosis: insights to disease pathogenesis. *Handb Clin Neurol* **122**, 231-266, doi:10.1016/B978-0-444-52001-2.00010-8 (2014).
2 Weinshenker, B. G. Natural history of multiple sclerosis. *Ann Neurol* **36 Suppl**, S6-11 (1994).
3 Reich, D. S., Lucchinetti, C. F. & Calabresi, P. A. Multiple Sclerosis. *N Engl J Med* **378**, 169-180, doi:10.1056/NEJMra1401483 (2018).
4 Giovannoni, G. *et al.* Is it time to target no evident disease activity (NEDA) in multiple sclerosis? *Mult Scler Relat Disord* **4**, 329-333, doi:10.1016/j.msard.2015.04.006 (2015).
5 Arnold, D. L. *et al.* Peginterferon beta-1a improves MRI measures and increases the proportion of patients with no evidence of disease activity in relapsing-remitting multiple sclerosis: 2-year results from the ADVANCE randomized controlled trial. *BMC Neurol* **17**, 29, doi:10.1186/s12883-017-0799-0 (2017).
6 Havrdova, E. *et al.* Effect of delayed-release dimethyl fumarate on no evidence of disease activity in relapsing-remitting multiple sclerosis: integrated analysis of the phase III DEFINE and CONFIRM studies. *Eur J Neurol* **24**, 726-733, doi:10.1111/ene.13272 (2017).
7 Miller, A. E. *et al.* Oral teriflunomide for patients with a first clinical episode suggestive of multiple sclerosis (TOPIC): a randomised, double-blind, placebo-controlled, phase 3 trial. *Lancet Neurol* **13**, 977-986, doi:10.1016/S1474-4422(14)70191-7 (2014).
8 Freedman, M. S. *et al.* Moving toward earlier treatment of multiple sclerosis: Findings from a decade of clinical trials and implications for clinical practice. *Mult Scler Relat Disord* **3**, 147-155, doi:10.1016/j.msard.2013.07.001 (2014).
9 Ramsaransing, G. S. & De Keyser, J. Benign course in multiple sclerosis: a review. *Acta Neurol Scand* **113**, 359-369, doi:10.1111/j.1600-0404.2006.00637.x (2006).
10 Sartori, A., Abdoli, M. & Freedman, M. S. Can we predict benign multiple sclerosis? Results of a 20-year long-term follow-up study. *J Neurol* **264**, 1068-1075, doi:10.1007/s00415-017-8487-y (2017).
11 Benedikz, J. *et al.* The natural history of untreated multiple sclerosis in Iceland. A total population-based 50 year prospective study. *Clin Neurol Neurosurg* **104**, 208-210 (2002).
12 Perini, P., Tagliaferri, C., Belloni, M., Biasi, G. & Gallo, P. The HLA-DR13 haplotype is associated with "benign" multiple sclerosis in northeast Italy. *Neurology* **57**, 158-159 (2001).
13 Johnson, K. M., Zhou, H., Lin, F., Ko, J. J. & Herrera, V. Real-World Adherence and Persistence to Oral Disease-Modifying Therapies in Multiple Sclerosis Patients Over 1 Year. *J Manag Care Spec Pharm* **23**, 844-852, doi:10.18553/jmcp.2017.23.8.844 (2017).
14 Lattanzi, S. *et al.* Persistence to oral disease-modifying therapies in multiple sclerosis patients. *Journal of neurology* **264**, 2325-2329, doi:10.1007/s00415-017-8595-8 (2017).
15 Lanzillo, R. *et al.* A multicentRE observational analysiS of PErsistenCe to Treatment in the new multiple sclerosis era: the RESPECT study. *Journal of neurology* **265**, 1174-1183, doi:10.1007/s00415-018-8831-x (2018).
16 Vermersch, P. *et al.* Teriflunomide versus subcutaneous interferon beta-1a in patients with relapsing multiple sclerosis: a randomised, controlled phase 3 trial. *Multiple sclerosis (Houndmills, Basingstoke, England)* **20**, 705-716, doi:10.1177/1352458513507821 (2014).
17 Balak, D. M. *et al.* Prevalence of cutaneous adverse events associated with long-term disease-modifying therapy and their impact on health-related quality of life in patients with multiple sclerosis: a cross-sectional study. *BMC neurology* **13**, 146, doi:10.1186/1471-2377-13-146 (2013).
18 Rommer, P. S. & Zettl, U. K. Managing the side effects of multiple sclerosis therapy: pharmacotherapy options for patients. *Expert opinion on pharmacotherapy* **19**, 483-498, doi:10.1080/14656566.2018.1446944 (2018).
19 Lee Mortensen, G. & Rasmussen, P. V. The impact of quality of life on treatment preferences in multiple sclerosis patients. *Patient Prefer Adherence* **11**, 1789-1796, doi:10.2147/ppa.S142373 (2017).
20 La Mantia, L., Munari, L. M. & Lovati, R. Glatiramer acetate for multiple sclerosis. *Cochrane Database Syst Rev*, Cd004678, doi:10.1002/14651858.CD004678.pub2 (2010).
21 Gold, R. *et al.* Placebo-controlled phase 3 study of oral BG-12 for relapsing multiple sclerosis. *N Engl J Med* **367**, 1098-1107, doi:10.1056/NEJMoa1114287 (2012).

22 Fox, R. J. *et al.* Placebo-controlled phase 3 study of oral BG-12 or glatiramer in multiple sclerosis. *N Engl J Med* **367**, 1087-1097, doi:10.1056/NEJMoa1206328 (2012).

23 Sejbaek, T., Nybo, M., Petersen, T. & Illes, Z. Real-life persistence and tolerability with dimethyl fumarate. *Mult Scler Relat Disord* **24**, 42-46, doi:10.1016/j.msard.2018.05.007 (2018).

24 CIBG; ministerie van Volksgezondheid, W. e. S. *Prijsvorming*, <www.farmatec.nl> (z.d.).

25 Kister, I. *et al.* Discontinuing disease-modifying therapy in MS after a prolonged relapse-free period: a propensity score-matched study. *J Neurol Neurosurg Psychiatry* **87**, 1133-1137, doi:10.1136/jnnp-2016-313760 (2016).

26 Bonenfant, J. *et al.* Can we stop immunomodulatory treatments in secondary progressive multiple sclerosis? *Eur J Neurol* **24**, 237-244, doi:10.1111/ene.13181 (2017).

27 Bsteh, G. *et al.* Discontinuation of disease-modifying therapies in multiple sclerosis - Clinical outcome and prognostic factors. *Mult Scler* **23**, 1241-1248, doi:10.1177/1352458516675751 (2017).

28 O'Rourke, K. E. & Hutchinson, M. Stopping beta-interferon therapy in multiple sclerosis: an analysis of stopping patterns. *Mult Scler* **11**, 46-50, doi:10.1191/1352458505ms1131oa (2005).

29 Rio, J. *et al.* Factors related with treatment adherence to interferon beta and glatiramer acetate therapy in multiple sclerosis. *Mult Scler* **11**, 306-309, doi:10.1191/1352458505ms1173oa (2005).

30 CBO richtlijn (2012) Immunomodulerende en immunosuppressieve behandeling bij multipele sclerose. (2012).

31 Kennisagenda neurologie 2017: https://gallery.mailchimp.com/29087cdad5c58a12bd346e83f/files/3b5692f1-3840-48e2-99e7-405edb9a895f/Kennisagenda_Neurologie_eindversie_16_12_2017.pdf.

32 Kister, I. & Corboy, J. R. Reducing costs while enhancing quality of care in MS. *Neurology* **87**, 1617-1622, doi:10.1212/WNL.0000000000003113 (2016).

33 Uher, T. *et al.* Is no evidence of disease activity an achievable goal in MS patients on intramuscular interferon beta-1a treatment over long-term follow-up? *Mult Scler* **23**, 242-252, doi:10.1177/1352458516650525 (2017).

34 Rotstein, D. L., Healy, B. C., Malik, M. T., Chitnis, T. & Weiner, H. L. Evaluation of no evidence of disease activity in a 7-year longitudinal multiple sclerosis cohort. *JAMA Neurol* **72**, 152-158, doi:10.1001/jamaneurol.2014.3537 (2015).

35 De Stefano, N. *et al.* Long-term assessment of no evidence of disease activity in relapsing-remitting MS. *Neurology* **85**, 1722-1723, doi:10.1212/WNL.0000000000002105 (2015).

36 Gray, O., McDonnell, G. & Hawkins, S. Tried and tested: the psychometric properties of the multiple sclerosis impact scale (MSIS-29) in a population-based study. *Mult Scler* **15**, 75-80, doi:10.1177/1352458508096872 (2009).

37 Pfennings, L. E. *et al.* A health-related quality of life questionnaire for multiple sclerosis patients. *Acta Neurol Scand* **100**, 148-155 (1999).

38 Rietberg, M. B., Van Wegen, E. E. & Kwakkel, G. Measuring fatigue in patients with multiple sclerosis: reproducibility, responsiveness and concurrent validity of three Dutch self-report questionnaires. *Disabil Rehabil* **32**, 1870-1876, doi:10.3109/09638281003734458 (2010).

39 Eagle, T. *et al.* Treatment satisfaction across injectable, infusion, and oral disease-modifying therapies for multiple sclerosis. *Mult Scler Relat Disord* **18**, 196-201, doi:10.1016/j.msard.2017.10.002 (2017).

40 Kobelt, G., Berg, J., Lindgren, P. & Jonsson, B. Costs and quality of life in multiple sclerosis in Europe: method of assessment and analysis. *Eur J Health Econ* **7 Suppl 2**, S5-13, doi:10.1007/s10198-006-0365-y (2006).

41 Janssen, M. F. *et al.* Measurement properties of the EQ-5D-5L compared to the EQ-5D-3L across eight patient groups: a multi-country study. *Qual Life Res* **22**, 1717-1727, doi:10.1007/s11136-012-0322-4 (2013).

42 Bouwmans, C. *et al.* The iMTA Productivity Cost Questionnaire: A Standardized Instrument for Measuring and Valuing Health-Related Productivity Losses. *Value Health* **18**, 753-758, doi:10.1016/j.jval.2015.05.009 (2015).

RESEARCH PROTOCOL

The safety and cost-effectiveness of discontinuing disease-modifying therapies in stable relapsing-onset multiple sclerosis (DOT-MS): a randomized rater-blinded multicenter trial.

Version 4, October 2020

TABLE OF CONTENTS

1. INTRODUCTION AND RATIONALE 11
2. OBJECTIVES 15
3. STUDY DESIGN 17
4. STUDY POPULATION 18
4.1 Population (base) 18
4.2 Inclusion criteria 19
4.3 Exclusion criteria 20
4.4 Sample size calculation 20
5. TREATMENT OF SUBJECTS 21
5.1 Investigational product/treatment 21
5.2 Use of co-intervention 21
5.3 Escape medication 21
6. INVESTIGATIONAL PRODUCT 21
7. NON-INVESTIGATIONAL PRODUCT 21
8. METHODS 21
8.1 Study parameters/endpoints 21
8.1.1 Main study parameter/endpoint 21
8.1.2 Secondary study parameters/endpoints (if applicable) 22
8.1.3 Other study parameters (if applicable) 23
8.2 Randomisation, blinding and treatment allocation 23
8.3 Study procedures 24
8.4 Withdrawal of individual subjects 28
8.4.1 Specific criteria for withdrawal (if applicable) 28
8.5 Replacement of individual subjects after withdrawal 28
8.6 Follow-up of subjects withdrawn from treatment 28
8.7 Premature termination of the study 28
9. SAFETY REPORTING 29
9.1 Temporary halt for reasons of subject safety 29
9.2 AEs, SAEs and SUSARs 29
9.2.1 Adverse events (AEs) 29
9.2.2 Serious adverse events (SAEs) 29
9.2.3 Suspected unexpected serious adverse reactions (SUSARs) 30
9.3 Annual safety report 30
9.4 Follow-up of adverse events 30
9.5 Data Safety Monitoring Board (DSMB) 30
10. STATISTICAL ANALYSIS 32
10.1 Primary study parameter(s) 32
10.2 Secondary study parameter(s) 33
10.3 Other study parameters 33
10.4 Interim analysis 33
11. ETHICAL CONSIDERATIONS 37

11.1 Regulation statement 37
11.2 Recruitment and consent....37
11.3 Objection by minors or incapacitated subjects (if applicable)....37
11.4 Benefits and risks assessment, group relatedness....37
11.5 Compensation for injury 38
11.6 Incentives (if applicable)....38
12. ADMINISTRATIVE ASPECTS, MONITORING AND PUBLICATION 39
12.1 Handling and storage of data and documents 39
12.2 Monitoring and Quality Assurance....40
12.3 Amendments....42
12.4 Annual progress report....42
12.5 Temporary halt and (prematurely) end of study report....42
12.6 Public disclosure and publication policy....42
13. STRUCTURED RISK ANALYSIS....44
13.1 Potential issues of concern....44
13.2 Synthesis 44
14. REFERENCES 45

LIST OF ABBREVIATIONS AND RELEVANT DEFINITIONS

**9HPT**	**9-Hole Peg Test**
**ABR**	**General Assessment and Registration form (ABR form), the application form that is required for submission to the accredited Ethics Committee; in Dutch: Algemeen Beoordelings- en Registratieformulier (ABR-formulier)**
**AE**	**Adverse Event**
**AR**	**Adverse Reaction**
**CA**	**Competent Authority**
**CCMO**	**Central Committee on Research Involving Human Subjects; in Dutch: Centrale Commissie Mensgebonden Onderzoek**
**CIS**	**Clinically Isolated Syndrome**
**CIS20r**	**Checklist Individual Strength**
**CV**	**Curriculum Vitae**
**DMT**	**Disease Modifying Therapy**
**DSMB**	**Data Safety Monitoring Board**
**EDSS**	**Expanded Disability Status Scale**
**EQ-5D-5L**	**EuroQol 5 Dimensions Questionnaire**
**EU**	**European Union**
**FLAIR**	**Fluid Attenuation Inversion Recovery**
**GCP**	**Good Clinical Practice**
**GDPR**	**General Data Protection Regulation; in Dutch: Algemene Verordening Gegevensbescherming (AVG)**
**IC**	**Informed Consent**
**iMCQ**	**Medical Consumption Questionnaire**
**iPCQ**	**Productivity Costs Questionnaire**
**METC**	**Medical research ethics committee (MREC); in Dutch: medisch-ethische toetsingscommissie (METC)**
**MRI**	**Magnetic Resonance Imaging**
**MS**	**Multiple Sclerosis**
**MSFC**	**Multiple Sclerosis Functional Composite**
**MSIS-29**	**Multiple Sclerosis Impact Scale**
**RRMS**	**Relapsing Remitting Multiple Sclerosis**
**(S)AE**	**(Serious) Adverse Event**
**SDMT**	**Symbol Digits Modalities Test**

**Sponsor**	**The sponsor is the party that commissions the organisation or performance of the research, for example a pharmaceutical company, academic hospital, scientific organisation or investigator. A party that provides funding for a study but does not commission it is not regarded as the sponsor, but referred to as a subsidising party.**
**SF-36**	**Short Form Health Survey**
**SPMS**	**Secondary Progressive Multiple Sclerosis**
**SUSAR**	**Suspected Unexpected Serious Adverse Reaction**
**T25fW**	**Timed 25-foot Walk**
**TSQM**	**Treatment Satisfaction Questionnaire for Medication**
**UAVG**	**Dutch Act on Implementation of the General Data Protection Regulation; in Dutch: Uitvoeringswet AVG**
**WMO**	**Medical Research Involving Human Subjects Act; in Dutch: Wet Medisch-wetenschappelijk Onderzoek met Mensen**

SUMMARY

Rationale: The past few years, several new effective drugs have come onto the market for the treatment of relapsing remitting MS (RRMS), all of which have potentially serious side effects. The arrival of these drugs has led to a new aim for treating MS patients: achieving a status of complete clinical and radiological control of inflammatory events, also described as a status of no evident disease activity (NEDA-3). With these adjusted goals, medication is often started at an earlier stage and the disease is treated more aggressively. This leads to better control of the disease, but also to increased exposure to possible (serious) side effects. A considerable group of patients with a fully stable-disease under treatment merely have a benign or less inflammatory disease course rather than a necessity for treatment to prevent inflammation. This raises the question whether and when patients who have been stable under medication for years can safely discontinue the treatment. The hypothesis of this study is that discontinuing medication after >5 years without evidence of inflammatory disease activity does not result in return of inflammatory disease activity.

Objective: The aim of this study is to identify whether it is possible to safely discontinue treatment in MS patients who have shown no evidence of active inflammation in the years prior to inclusion clinically and/or radiologically. The secondary objectives address the questions whether the discontinuation of first-line treatment has an effect on disability progression and whether the discontinuation of first-line treatment improves the quality of life for the patient and if this can be measured in a daily setting using digital biomarkers.. Furthermore, blood collections will be included to assess whether it is possible to retrospectively predict possible return of inflammatory activity with biomarkers such as neurofilament light (NFL) or patient characteristics such as disease activity prior to disease modifying therapy (DMT). In case of emerging disease activity after the cessation of therapy we will assess if reinitiation will lead to NEDA again, and if there are long-term consequences. If possible, post-hoc analysis are performed for the different types of treatment compounds.

Study design: Multi-center randomized and controlled, rater-blinded trial in the Netherlands. 130 patients with relapse onset MS will be assigned to either discontinue the previously used DMT or to continue their DMT.

Study population: MS patients who are treated with one of the first-line treatments (any of the interferons, glatiramer acetate, dimethylfumarate, teriflunomide) and who had a complete absence of inflammatory activity (no relapses, no new-T2 lesions and no contrast-enhancing lesions) for 5 consecutive years under first-line treatment will be eligible for inclusion.

Intervention (if applicable): discontinuation of the previously used DMT.

Main study parameters/endpoints: The primary endpoint is the return of inflammatory disease activity after 2 years: either relapses, new or enlarging lesions on T2-weighted MRI

and gadolinium-enhancing lesions on post-contrast T1-weighted MRI. Secondary end-points are EDSS and MSFC progression (combined: EDSS plus), number of relapses, individual MRI-parameters (such as lesion numbers), quality of life measurements, optical coherence tomography (OCT) and eye movement measurements, and (digital) biomarker measurements.

Nature and extent of the burden and risks associated with participation, benefit and group relatedness: The burden of participation consists of assessments during visits at baseline, 3, 6, 12, 18 and 24 months. Every follow-up visit of both patient groups will consist of clinical and radiological measurements, quality of life questionnaires and blood collection. Additional data will be collected via mobile applications MS sherpa and Neurokeys, that will be installed on patients' smartphones. For this, patients will be asked to perform tasks on their phones, measuring walking ability, hand function, cognition and fatigue. This takes approximately five minutes every two weeks and is performed at home. The data on the discontinuation of therapy in long-term inflammatory stable MS patients is reassuring. One large retrospective cohort study has shown in 1200 patients that stable RRMS patients who stop treatment don't have an increased relapse rate compared to patients who continue treatment (Kister et al). There was a slightly higher disability progression of the discontinuation group, presumably based on the discontinuation of treatment in patients with progressive MS. Also, there seems to be no rebound inflammation after discontinuation of therapy in progressive MS, and a similar relapse rate as the years prior to discontinuation (Bonenfant et al). When disease activity emerges, patients are treated according to the current standard of care. The discontinuation of medication can be beneficial for the patient, as the side effects of medications can be significant for some patients.

1. INTRODUCTION AND RATIONALE

In recent decades, the therapeutic landscape of multiple sclerosis (MS) has changed dramatically. Coming from an era where virtually no therapies were available, there are currently more than 12 first- and second-line disease modifying treatment (DMT) options for the prevention of focal inflammatory demyelinating lesions in the brain and spinal cord. Clinically MS can be devastating; it affects roughly 1 in 1000 persons in the Netherlands usually diagnosed in the prime of their lives with a mean age at diagnosis around 30[1]. Two major disease phenotypes exist. The most important is relapsing-onset MS (80% MS patients), including patients with a clinically isolated syndrome (CIS), relapsing remitting MS (RRMS) and secondary progressive MS (SPMS). The other 20% suffers from a primary progressive MS (PPMS), a disease phenotype with more distinct neurodegeneration. Untreated, 50% of patients will need assistance walking small distances after 10-20 years after diagnosis[2].The main pathological hallmark in the first stages (CIS and RRMS) is recurrent focal inflammation of the brain and spinal cord leading to demyelination[3]. The first years after the diagnosis patients usually experience 2-3 relapses annually. The severity of neurological disability depends on the localisation of the inflammation. With increasing age, the amount of inflammation tends to diminish and an unknown neurodegenerative pathology drives the disease course. Clinically there is a progressive decline in neurological functioning; i.e. the “progressive” phase (or the secondary progressive (SPMS) disease course).

There has been great change in the timing of diagnosing MS and evaluating disease activity with the introduction of magnetic resonance imaging (MRI). In the early days the disease course was solely evaluated based on new relapses and/or progression of disability. Now, the arrival of MRI has led to a revised aim for treating MS patients: achieving a status of “no evident disease activity” (NEDA-3); complete clinical and radiological control of inflammatory events and no significant increase in diseability[4]. To date an estimated 10% of patients have a status of NEDA-3 under long-term first-line therapy, implying a full control of focal inflammation in these patients[5-7]. In recent years there has been a growing trend of starting treatment earlier and to treat more aggressively, partly based on the concept of NEDA. Treatment is almost always initiated directly after diagnosis but sometimes even before a definite diagnosis of RRMS is made[8]. There is however a substantial percentage of patients with a more benign disease course, described in a very broad range of 6-64% of MS patients[9-12]. At the moment of diagnosis, it is not known how the disease course will develop and based on the substantial group of “benign” MS cases, it is likely that a considerable group of patients that have long-term and fully stable-disease under DMT is unnecessarily

treated. In addition, there is the group of patients who experience disease progression despite their therapy. Also, these patients probably do not benefit from their therapy.

Exposure to treatment is not without risks and costs. Side-effects of MS medication are frequently present. Data on the proportion of patients discontinuing first-line DMT's demonstrates a discontinuation rate of 20-40% during an observation period over 1 year, with the occurrence of side effects and poor tolerability as the most common reason for drug withdrawal[13-15]. A large proportion of the patient population is confronted with side effects for both oral (teriflunomide, dimethylfumarate) and injectable (interferons, glatiramer acetate) DMT's. For example, the results of the post-approval clinical trials on the safety of teriflunomide compared to interferon beta-1a showed that 93-96% of the patients experienced side effects[16]. Each injectable first-line DMT can lead to mild cutaneous adverse events such as erythema and swelling, but also to more severe and persisting effects such as lipoatrophy, infections and even necrosis. Patients with a cutaneous reaction appeared to have a lower dermatology-specific health-related quality of life[17]. Serious events rates were also high ranging from 7% (interferon beta-1a) to 12% (teriflunomide)[16].

Each first-line DMT has a different mode of administration and specific side-effects. Interferons are administered biweekly s.c. (Plegridy), weekly i.m. (Avonex), second daily s.c. (Betaferon) or thrice weekly s.c. (Rebif). Flu-like symptoms are the most often reported side effects of interferon ß injections and are particularly challenging for MS patients[18,19]. But also allergic reactions, elevated liver enzymes leading to severe hepatic injury, thyroid autoimmunity, hypothyroidism and hematologic abnormalities might occur[18]. Glatiramer acetate (Copaxone) is injected subcutaneously daily or thrice weekly. Patterned reactions are most commonly reported in patients using glatiramer acetate, consisting of flushing, chest pain, palpitations, urticaria, anxiety and dyspnoea with a relative risk of 3.27. This patterned reaction unpredictably occur within minutes of injection and spontaneously resolve before 30 minutes[20]. Teriflunomide (Aubagio) is an oral drug and is administered daily. Hair thinning, increased blood pressure, fatigue, diarrhoea, sensory disturbances, elevated liver enzymes, and renal failure are adverse effects that have been reported in patients using teriflunomide[18]. The fourth agent in the first-line DMT group, dimethylfumarate (Tecfidera) is taken orally twice daily. Clinical phase 3 trials reported mild or moderate flushing and gastrointestinal (GI) adverse events, 36% and 42%, respectively as most common adverse effects[21-23]. Consequently, this led to treatment discontinuation in both trials. Serious side effects include urosepsis (interferons), hepatotoxicity (glatiramer acetate), but also progressive multifocal leukoencephalopathy (dimethyl fumarate), which are all potentially lethal.

Furthermore, there is a great burden of costs to society. The costs for a year of first-line immunomodulating drugs range from 12.000 – 15.000 euros annually[24]. The discontinuation of therapy in appropriate patients therefore also has a very significant effect with a potential cost-reduction of 2-2.5 million euros annually in The Netherlands. Costs due to side-effects (such as treatment and absence of work) are not even included here.

The data on the discontinuation of therapy in long-term inflammatory stable MS patients is reassuring. One large retrospective cohort study has shown in 1200 patients that stable RRMS patients who stop treatment don't have an increased relapse rate compared to patients who continue treatment[25]. There was a slightly higher disability progression of the discontinuation group, presumably based on the discontinuation of treatment in patients with progressive MS. Also, there seems to be no rebound inflammation after discontinuation of therapy in progressive MS, and a similar relapse rate as the years prior to discontinuation[26]. Lastly, one study has showed that patients 45 years or older, or patients with a DMT intake of 4 or more years without evidence of clinical or radiological disease activity showed a high likelihood of remaining relapse-free after discontinuation and absence of contrast enhancing lesions[27]. All studies where however hampered by either its retrospective nature, or incompleteness on for examples reasons for discontinuation or a sufficiently matched control group. Obviously, definite conclusions can only be drawn after a well-designed controlled trial. Currently, two trials with a similar question to ours are underway (DISCOMS; NCT03073603 and STOP-I-SEP; NCT03653273). These studies are different compared to our proposal in that the inclusion criteria only allow for older patients (older than 55 years and older than 50 years respectively) to be included.

The question of whether or not to discontinue therapy is increasingly present during our outpatient clinics [28,29]. As stated, an estimated 10% of patients have a status of NEDA-3 under long-term first-line DMT and could benefit from the results of this study. To give an indication of the number of patients concerned: in the Netherlands alone an estimated number of 7000-7500 patients with MS use first-line DMT, which means 700-750 patients would be eligible for this study and would benefit from results of this study (numbers are based on market shares and sales of each DMT, data not published). An enquiry amongst Dutch MS neurologists (data not published) shows that every neurologist struggles with the question on average 5-10 times per year. The Dutch 2012 CBO guideline " Multiple Sclerosis" recommends the discontinuation of DMT in patients who experience severe side-effects and who had secondary progressive MS for at least 3 years[30] but this is solely based on expert opinion. Also, internationally there are no guidelines guiding patients and neurologists in this question. Very recently in 2017, the committee of care evaluation of

neurology (‘Zorgevaluatie Neurologie’ (ZEN), part of the ‘Dutch society of Neurologists (Nederlandse Vereniging voor Neurologie; NVN) and supported by the Dutch Federation of medical specialists (FMS) and the Dutch Federation of Patients) has ranked this lack of evidence regarding the discontinuation of immunomodulating drugs in MS one of the most important science/knowledge gaps within daily neurological practice in the Netherlands. It listed this topic in its “ Kennisagenda 2018-2022” which prioritizes the 12 most urgent topics for scientific research in the field of neurology in the coming 4 years to improve the effectiveness and safety of our daily medical practice[31]. We have validated this support by means of a questionnaire amongst all 88 members of the MS Taskforce (Part of the NVN). All (100%) respondents (response-rate was 49%) indicated that they found the issue frequently present in their clinics and important tot research. Also, internationally there is strong call for more evidence-based guidelines and consensus regarding the discontinuation of treatment in long-term stable MS patients[32]. This underlines the national (and international) need for systematic research regarding this subject.

2. OBJECTIVES

With this study we will bring first evidence to this important issue by identifying whether it is possible to safely discontinue treatment in MS patients who have shown no evidence of active inflammation in the years prior to inclusion. Importantly, we include quality of life measurements to evaluate if this also translates to an increased perception of health. If present, it will optimize the treatment paradigm for individual MS patients through identifying unnecessary exposure to medication while proving the discontinuation of medication to be safe, and beneficial for the daily lives of individual patients in terms of an increased quality of life. In addition, it greatly benefits the general society since it also provides a more efficient use of funds with the significant cost-reduction it brings. The impact of the trial is immediate, within 5 years, and since all large MS centers will participate there is a direct line into the daily offices of treating neurologists, and into the development of adjusted guidelines regarding the treatment of MS. Most importantly, every possible result deriving from this trial will have a significant impact on (inter)national treatment strategies. Besides the primary question of the evaluation of safety we also incorporate validated measures of quality of life and disease perception to evaluate potential changes in quality of life. Furthermore, we include blood collections for the monitoring of neurofilament light (currently the only validated and clinically applied biomarker for the return of inflammatory activity) that could potentially serve as indicator for subclinical return of inflammatory activity. In addition, we will include optical coherence tomography (OCT) and eye movement measurements for patients participating in Amsterdam UMC. OCT measurements (especially retinal nerve fiber layer (RNFL) thickness) are known to be associated with disability in MS patients, and thus can be seen as a measure for disease progression.

Primary outcome measures used in this study are clinical and radiological outcome measures that are already part of routine clinical practice, which ensures that results of this trial can be directly and easily implemented in standard clinical care. In addition to these outcome measures, it would be of interest to determine optimal measurements for potential return of disease activity. A promising method in this regard is the use of mobile applications on patients' smartphones, because of their non-invasive nature (in contrast to for example MRI-scans) and the potential for more continuous measurements in a real-world setting (i.e. at home). Measurements with mobile applications MS sherpa and Neurokeys will be included for a subgroup of patients (depending on the availability of a smartphone), to investigate if return of inflammatory disease activity can be measured with these applications. These mobile applications will be installed on patients' smartphones.

See also methods section for a full description.

Research questions

Primary research question:

Can we safely discontinue first-line medication in MS patients with long-term absence of inflammation, without the return of *inflammatory disease activity* clinically and radiologically?

Other research questions:

- Does the discontinuation of first-line treatment have an effect on disability progression?
- Does the discontinuation of first-line treatment improve the quality of life for the patient?
- What is the effect of discontinuation of first-line treatment on individual MRI outcome measures such as lesion load and atrophy measurements?
- Is it possible to predict possible return of inflammatory activity with biomarkers such as neurofilament light (NFL) or patient characteristics such as disease activity prior to DMT?
- In case of emerging disease activity after cessation of DMT, will a restart of DMT result in NEDA again and if so, how long does it take?
- In case of emerging disease activity after treatment cessation, are there any differences between the different DMT compounds?
- What is the cost-effectiveness of discontinuation of DMT in The Netherlands?
- Is discontinuation of first-line DMT associated with OCT measurements and eye movement measurements?
- Is it possible to detect and predict (return of) inflammatory disease activity and disease progression with digital biomarkers using mobile applications such as MS sherpa and Neurokeys?

3. STUDY DESIGN

The study design is a multi-center rater-blinded randomized controlled trial in the Netherlands. The project will take place over period of 5 years (60 months) depending on the progress of inclusion. The lead and monitoring of the trial will be performed by the MS center Amsterdam (MSCA). Participating centers are listed in an appendix (I1).

Study-population: 130 relapse onset MS patients who are treated with one of the first-line treatments (any of the interferons, glatiramer acetate, dimethylfumarate, teriflunomide) *and* who had a complete absence of objective inflammatory activity (no objectified relapses, no significant number (2 or more) of new-T2 lesions and no contrast-enhancing lesions) for 5 consecutive years under first-line treatment will be eligible for inclusion. Patients may not have switched between first-line drugs over the two years prior to inclusion. If a switch has occurred this should not have been due to ineffectivity of the first DMT but due to side-effects or by a personal preference of the patient (such as the wish to switch to oral therapies). In the case of previous use of interferons patients must be negative for neutralizing antibodies (NAbs). Inclusion will take place after informed consent. This will be obtained after careful and extensive information about the possible risks according to local ethical review board requirements.

Intervention: The intervention is the discontinuation of the previously used DMT. Based on the pharmacological profile of the abovementioned drugs that are eligible for inclusion, there is no need for a tapering of dosage before complete discontinuation

Follow-up frequency: Patients in the continuation group are expected to remain stable throughout the study period based on their proven stable status for 5 consecutive years prior to enrolment. The minimum evaluation that must take place should have a frequency according to the current standard of care (yearly evaluation of every patient treated with immunomodulating therapy; i.e. baseline (BL), 12 and 24 months). Timely recognition of recurrence of any (subclinical) disease activity in the discontinuation group is secured by more frequent clinical and radiological assessment during extra routine study visits at 3, 6 and 18 months. For optimal comparability and to overcome potential bias, both groups will be followed with a complete assessment including MRI at BL, 3, 6, 12, 18 and 24 months.

After these two years of follow-up, the patients in the continuation group are offered to discontinue their DMT with a follow-up similar to the discontinuation group.

4. STUDY POPULATION

4.1 Population (base)

We will include 130 participants with the diagnosis relapse onset MS with a minimum age of 18 years. 65 patients will be assigned to the discontinuation group and 65 patients will be assigned to the continuation group. MS patients will be recruited through the VUmc outpatient clinic and the outpatient neurology departments of all participating centers. Because this research has a multicenter national design, MS patients of the majority of regions in the Netherlands will be included, including urban as well as rural areas. We believe that represents a valid cross-section of the average MS patient population. It is important to note however, as MS affects 2-2.5x as many women as men we expect to include more women than men. We will include a randomization algorithm to match both groups for sex to exclude potential bias.

We have estimated 700-750 patients to be potentially eligible for inclusion in The Netherlands. Currently an estimated number of 7000-7500 patients use first-line DMT for their MS. This number is estimated on data that was provided by all different pharmaceutical companies of each of the first-line therapies. Numbers are based on market shares and actual sales of each of the treatments (data not published).

The percentage of “stability” derives from the treatment effects described in the pivotal phase III trials of the currently available first-line treatments and 3 cohort studies with a longer follow-up that the average 2 years in the phase III trials. For our calculation of possible eligible patients, we have used the most “negative” scenario based on the results of these trials. The most recent phase III studies report on NEDA as outcome measure (Havrdova et al 2017, Arnold et al 2017), reflecting the new treatment concept of MS: complete stabilization of the disease process. In those cohorts NEDA ranges from 25-40% (Havrdova et al 2017, Arnold et al 2014 & 2017, Miller et al 2014, Nygaard et al 2015). It is important to note however, that the concept of NEDA also includes progressive neurological decline (such as is due to progressive disease and neurodegeneration). The percentage of patients that is free from inflammation is therefore somewhat underestimated in the numbers of these studies. Furthermore, most studies comprise on average a relatively short observation period of 2 years.

Three cohort studies exist with a longer follow-up. De Stefano et al, Uher et al and Rotstein et al published cohort studies that describe a longitudinal follow-up of NEDA status of ~ 200 patients for 7-10 years[33-35]. They showed that a fairly low percentage of patients remained

NEDA after 5 years (10-15%). In the cases of emerging inflammatory activity under treatment, most patients showed disease activity in the first 1-2 years after the start of treatment. Only a small minority of patients lose their NEDA status after 5 years (4%). It is very important to note that in the Rotstein-study the loss of NEDA status was in a large proportion due to disease progression rather than new inflammation. This causes a relative underestimation of patients who remain inflammatory stable. Taken together and based on this data, we assume that in the of all first-line DMT users 10% show no signs of inflammation for 5 years, and only a very small percentage of patients shows additional disease activity while staying on treatment (5%). The majority (95%) of patients remain inflammatory stable while continuing their medication.

This makes 700-750 patients eligible for inclusion.

4.2 Inclusion criteria

In order to be eligible to participate in this study, a subject must meet all of the following criteria:

1. A minimum age of 18 years
2. Ability to understand the purpose and risks of the study and provide signed and dated informed consent and authorization to use protected health information (PHI) in accordance with national and local privacy regulations.
3. Definite diagnosis of relapsing-onset MS according to the revised McDonald 2017 criteria
4. All relapsing-onset MS patients treated with one of the first-line treatments: any of the interferons, glatiramer acetate, dimethylfumarate, teriflunomide
5. Complete absence of inflammatory activity (no objectively defined and confirmed relapses, no significant number (2 or more) of new-T2 lesions suggestive of demyelination and no contrast-enhancing lesions) suggestive of demyelination for 5 consecutive years under first-line treatment. In case the last available MRI-scan was conducted 10 or more years ago, no more than 3 new T2-lesions suggestive of demyelination in the last 10 years are accepted.
6. Daily use of a smartphone with Android (5.0 or higher) or iOS (10 or higher) operating system.

4.3 Exclusion criteria

A potential subject who meets any of the following criteria will be excluded from participation in this study:

1. A switch between first-line disease modifying therapy over two years prior to inclusion, in case the switch has been due to in effectivity of the first DMT. In case the switch has been due to side-effects or by a personal preference of the patient (such as the wish to switch to oral therapies), this is not considered as an exclusion criterium.
2. Women who want to discontinue medication because of a pregnancy wish and women who are pregnant or expect to become pregnant during the study period
3. Patients that have previously used interferon-beta and have been tested positive for neutralizing antibodies (NAbs). This is determined by measuring MxA-bioactivity and is a test that is part of routine follow-up in patients that use interferon-beta. The reason for this is that development of NAbs has been shown to affect interferon-beta treatment efficacy.

4.4 Sample size calculation

The stability of patients in the continuation group is estimated to be at least 97,5%. Based on a non-inferiority margin of 7,5%, a preliminary power-calculation based on the non-inferiority principle was performed (PASS v12, one-sided Z-test (unpooled), significance level 0.05) and showed a necessary sample size of 54 per group to achieve 80% power. Taking 20% drop out into consideration, the total sample size needed for this study is 130.

The applications MS Sherpa and Neurokeys will be added to collect digital biomarkers regarding (return of) inflammatory disease activity. Based on previous experience, our estimate is that the current sample-size can provide some conclusive trends on the association between digital biomarkers and (return of) inflammatory disease activity.

5. TREATMENT OF SUBJECTS

5.1 Investigational product/treatment

The intervention consists of the discontinuation of the previously used DMT (either interferons, glatiramer acetate, dimethylfumarate or teriflunomide) Based on the pharmacological profile of the abovementioned drugs that are eligible for inclusion, there is no need for a tapering of dosage before complete discontinuation.

5.2 Use of co-intervention

During the intervention period patients are asked not to participate in any other scientific studies. Patients are allowed to use all types of co-medication, except for immunomodulating drugs such as prescribed for the treatment of multiple sclerosis and/or other auto-immune diseases.

5.3 Escape medication

When disease activity emerges, patients are treated according to the current standard of care (including intravenous methylprednisolone if deemed necessary) and disease modifying treatment will be reinitiated. Unscheduled visits including an MRI-scan are planned for each patient with any new neurological complaints, as is standard clinical procedure.

6. INVESTIGATIONAL PRODUCT

N/A

7. NON-INVESTIGATIONAL PRODUCT

MS Sherpa and Neurokeys are CE-certified medical devices. See for the relevant details appendices “D6 - Aanvullende productgegevens MS sherpa” and “D6 – Aanvullende productgegevens Neurokeys”.

8. METHODS

8.1 Study parameters/endpoints

8.1.1 Main study parameter/endpoint

The primary endpoint is number of patients with return of inflammatory disease activity after 2 years based on: a clinically confirmed relapse (defined according to the definition most often used in MS phase-III trials: the onset of new or recurrent symptoms that last > 24 hours, that are accompanied by new objective abnormalities on a neurological examination and that are not explained by non-MS processes such as fever, infection, severe stress or

drug toxicity (Gold et al NEJM 2012)) , or any emerging subclinical disease activity proven to be due to active disease/new inflammation (defined as 3 or more lesions on T2—weighted images or 2 or more gadolinium enhancing lesions on T1-weighted post-contrast MRI suggestive of demyelination) in the discontinuation group.

8.1.2 Secondary study parameters/endpoints (if applicable)

Secondary end-points are

- Changes in neurological functioning
    - EDSS change (Including individual functional systems)
    - MSFC changes
        - Timed 25-foot Walk (T25fW)
        - 9-Hole Peg Test (9HPT)
        - Symbol digits modalities test (SDMT)
- Individual MRI-parameters
    - T1 post-contrast lesion numbers and volumes
    - T2 lesion numbers and volumes
    - Whole brain volume
    - Normalized white matter volume
    - Grey matter volume
- Changes in quality of life measurements
    - Multiple Sclerosis Impact Scale (MSIS-29)
    - Short Form health survey (SF-36)
    - Multiple Sclerosis Self-Efficacy scale (MSSE)
    - Checklist Individual Strength (CIS20r)
    - Treatment Satisfaction Questionnaire for Medication (TSQM)
- Cost measurements
    - EuroQol 5 dimensions questionnaire (EQ-5D-5L)
    - Medical consumption questionnaire (iMCQ)
    - Productivity costs questionnaire (iPCQ)
- Changes in biomarker measurements
    - Neurofilament levels
- OCT and eye movement measurements
    - Peri-papillary retinal nerve fiber (RNFL) thickness
    - Macular ganglion cell-layer inner plexiform layer (GCL-IPL) thickness
    - Eye movement measurements

- Changes in digital biomarkers using the NeuroKeys (CE) and MS sherpa (CE) mobile applications that measure:
  - Walking test (2-minute walking test)
  - Cognition test (similar to SDMT)
  - MS sherpa questionnaires (including fatigue)
  - Keystroke data

8.1.3 Other study parameters (if applicable)

Vitamin D, smoking high body-mass index (BMI) are considered as potential confounders. Therefore, vitamin D will be determined and patients will be asked about smoking behaviour during every visit. To avoid any potential bias, we will also collect data (if present in the individual patients) on disease activity prior to the initiation of DMT, such date of diagnosis, time from first symptoms to diagnosis, EDSS scores/MS severity at the moment of DMT initiation.

8.2 Randomisation, blinding and treatment allocation

Since MS affects 2-2.5x as many women as men, we expect to include more women than men. A randomization algorithm will be included to match both groups for sex and age to exclude potential bias.

Outcome measurements will be performed by assessors who are blind to the intervention assignment. For reasons of consistency and to exclude possible bias, all scans will be centrally reviewed in VUmc by a radiologist blinded to allocation to the intervention group. In the current set-up of the trial patients and their treating neurologists are unblinded to the randomization group. The currently available first-line disease modifying treatments consist of the various (peg)interferons, glatiramer acetate, teriflunomide and dimethylfumarate. It comes in a total of more 10 different forms with different packaging, different modes of injection (some subcutaneously, some intramuscular and some oral) and different frequencies of intake (ranging from twice daily to once every two weeks). We have set up the trial so that it is rater-blinded; all MRI-scan and clinical evaluations (such as the neurological examination) will be performed blinded to “treatment” allocation. The primary and majority of secondary outcome measures are derived from these blinded assessments. This approach is frequently chosen in MS research (even in the phase-III trials) for 2 very important reasons. Due to the very distinct nature of side-effects (flushing, gastro-intestinal problems, flu-like symptoms), patients know when they receive placebo instead of the actual active compound. The invasive nature of receiving placebo-injections would greatly enhance the possibility of patients not participating. In addition, we believe it is too costly to create a placebo-control for each of the 10 different forms of medication.

8.3 Study procedures

No study procedures will take place before having obtained informed consent which will be gained following current METc/CCMO standards.

Follow-up frequency

Patients in the continuation group are expected to remain stable throughout the study period based on their proven stable status for 5 consecutive years prior to enrolment. The minimum evaluation that must take place should have a frequency according to the current standard of care (yearly evaluation of every patient treated with immunomodulating therapy; i.e. baseline (BL), 12 and 24 months). Timely recognition of recurrence of any (subclinical) disease activity in the discontinuation group is secured by more frequent clinical and radiological assessment during extra routine study visits at 3, 6 and 18 months. For optimal comparability and to overcome potential bias , both groups will be followed with a complete assessment including MRI at BL, 3, 6, 12, 18 and 24 moths. For the patients in both groups mobile applications MS sherpa and Neurokeys will be installed. With the MS sherpa application, patients will be asked to perform tests every two weeks during the 24 months of follow-up. The Neurokeys application will collect data on the background of the standard use of mobile phones during this time period.

Clinical evaluation

Duration: 1 hour

Clinical evaluation will consist of a careful medical history: current and past medication, adverse events, number of intravenous methylprednisolone treatments, number of relapses, date of last relapse and signs of symptom progression. A relapse is defined according to the definition most often used in MS phase-III trials: the onset of new or recurrent symptoms that last > 24 hours, that are accompanied by new objective abnormalities on a neurological examination and that are not explained by non-MS processes such as fever, infection, severe stress or drug toxicity[21]. Furthermore, the Expanded Disability Status Scale (EDSS) and Multiple Sclerosis Functional Composite (MSFC)-measurements will be performed, consisting of the Timed 25-foot Walk (T25fW), 9-Hole Peg Test (9HPT) and the symbol digits modalities test (SDMT). This will be done by a blinded investigator.

Radiological evaluation

Duration: 45 min

Radiological evaluation will consist of repeated brain MRI investigations that consist of conventional pre- and post-contrast (T2-weighted, T1-weighted pre and post contrast, FLAIR) images. All scan protocols are available in general and academic hospitals since they form the basis on which MS is diagnosed and treatment is monitored. Although it is expected that new inflammatory lesions can be captured by repeated T2-weighted/FLAIR MRI-scans, a gadolinium-scan is included to not miss any contrast enhancement in previously present lesions. A 3DT1 and 3DFLAIR image will also be made for atrophy measurements. For reasons of consistency and to exclude possible bias, all scans will be centrally reviewed in VUmc by a radiologist blinded to allocation to the intervention group.

Questionnaires

Duration: 1 hour

For the evaluation of disease burden and MS related symptoms, we will use the validated and in clinical studies often used Multiple Sclerosis Impact Scale (MSIS-29)[36] Short Form health survey (SF-36)[37] and CIS20r[38]. The perceived impact of treatment, convenience, satisfaction and side-effects will be monitored using the Treatment Satisfaction Questionnaire for Medication (TSQM)[39]. For the evaluation of health related quality of life (HRQoL) and the link between symptoms HRQoL and costs, we use the EDSS for the objective measurement of changes in neurological functioning (which has a clear relation to HRQoL assessed as utility and costs[40]) and the number of patients with return of inflammatory activity. We also use the EQ5D-5L) for cost-utility analysis[41]. Furthermore, we will ask patients to keep a diary describing changes in healthcare consumption (which will be defined per item in a questionnaire (such as hospitalisations, consultations with doctors, use of care at home, use of specialized transportation etc). Lastly patients will be asked for their employment situation and short-term and long-term sick absence because of MS (or MS related treatment) using the iMCQ and iPCQ questionnaires[42]. Questionnaires are filled in digitally either at home or during the hospital visit. Help of a carer or the study-nurse is allowed in cases of the inability using a computer.

Samples

Duration: 15 minutes

Blood collection will take place in both patient groups at every visit. It will consist blood collection for the purpose of biobanking and for diagnostics in the case of any –unforeseen- clinical events. Furthermore, we will retrospectively measure neurofilament light in serum using Simoa.

All participants will visit the hospital for 6 times over a time frame of 2 years. Each of these visits that take place will take approximately 2,5-3 hours (clinical assessment and MRI-scan). Also 1-hour questionnaires which can be completed at home digitally or at the hospital on paper will take place before/during each visit.

All samples will be collected, processed and stored according to the Standard Operating Procedures (SOP's) as described in the Parelsnoer Biobankprotocol version 8.0. (15) Samples will be stored at the Biobank VUmc. To ensure patient privacy all samples will be coded. A peripheral blood sample will be collected, in total 8 tubes of blood will be drawn (5x EDTA 4 ml for plasma, cells and DNA isolation, 2x serum 5 ml, 1x PAXgene tube 2 ml), adding up to a total volume of 32 ml. Blood will be centrifuged, divided in aliquots of 0.5 ml and then stored at -80 °C.

Optical coherence tomography (OCT)

Duration: 15 minutes

Frequency: yearly (at baseline or month 3, month 12 and month 24)

All OCT measurements will be performed by a certified person (not necessarily a physician) on a spectral-domain OCT machine (Spectralis by Heidelberg engineering). Scans to be performed in both eyes:

- Circular scan, centered on optic nerve head (ONH)
- Volume scan, centered on macula.
- Volume scan, centered on optic nerve head (ONH)

Eye movement examination

Frequency: yearly (at baseline or month 3, month 12 and month 24)

Duration: 15 minutes

Eye movement measurements will be performed using Eyelink 1000 Plus Eye Tracker (SR Research). Built-in algorithms provided by the eye tracker are used for calibration and validation procedures. The experiment consists of the following assessments:

1. Fixation
2. Pro-saccades
3. Anti-saccades

Mobile applications measurements

Duration: 5 minutes

Frequency: once every two weeks.

For eligible patients (based on regular smartphone use), mobile applications MS-Sherpa and Neurokeys will be installed on their smartphones and data will be collected via these applications.

MS sherpa mobile application

Via the MS sherpa application the patient will be asked to perform walking tests, cognition tests and the MS sherpa questionnaire (including fatigue).

- Cognitive task: similar to the SDMT, the participant is requested to assign numbers to corresponding symbols according to a specific displayed coding.
- Motor task: 2-minute walk test (2MWT): the participant is requested to walk (either unassisted or with a walking aid) for two minutes. The walking distance is measured through the location data. Patients who are unable to walk for two minutes will not be requested to do the 2MWT.
- MS sherpa questionnaire: patient reported outcomes on Likert scales (amongst others about fatigue and the impact of MS on daily activities).

These tests can be completed in approximately 5 minutes and patients will be asked to complete these tests once every two weeks.

NeuroKeys mobile application

NeuroKeys replaces the standard keyboard of the patient's smartphone. Data is collected from regular use of the keyboard, and no additional action from patients is needed. After 24 hours of inactivity of the keyboard of NeuroKeys, either intentionally or unintentionally, a push notification is automatically send utilizing Amazon Simple Notification Service (SNS). NeuroKeys will collect keystroke data general profile information (gender, year of birth, and MS type), and metadata (iOS/Android version and NeuroKeys version). The data will be collected from the phone continuously in a retrospective fashion, when the keyboard is activated the data from the previous keyboard typing session is sent.

- Keystroke data: the start of a message is defined as the keyboard flipping up and the end of a message is marked when the keyboard flips down. Specific keys will be logged and timestamped to be able to accurately calculate parameters expected to be associated to fatigue. The keys logged are: delete or backspace key, dot key, space bar, semicolon, colon, parentheses, capitalized characters, numbers and punctuation marks denouncing the ending of a sentence. All numbers and all punctuation marks will be logged as the same number or punctuation event (e.g. we do not make a distinction

between the number 3 and 8 or the comma and dollar sign). Parameters such as word count, amount of words comprised of six characters or more and latency between key presses will be calculated on the basis of the logged keys. In addition to ASCII keys, the unicodes of emojis will also be logged.

- Sensor data: Data from the location sensor, ambient light sensor, gyroscope, motion and accelerometers of the smartphone will be collected when the keyboard is in use. With the location sensor (longitude and latitude), the keystroke data can be combined to open source databases (e.g. weather data from the Royal Netherlands Meteorological Institute, KNMI) to examine the influence of external factors (Davis et al. 2010). Existing and future open source databases provided by public or governmental institutes can be accessed for this purpose. Ambient light sensor can detect environmental brightness which could impact typing behaviour. Kinematic sensors (gyroscope, motion and accelerometers) may be used to infer posture of the user (Lamonaca et al. 2015).

8.4 Withdrawal of individual subjects

Subjects can leave the study at any time for any reason if they wish to do so without any consequences. The investigator can decide to withdraw a subject from the study for urgent medical reasons.

8.4.1 Specific criteria for withdrawal (if applicable)

There are no specific criteria for withdrawal from the study.

8.5 Replacement of individual subjects after withdrawal

There will be no replacement of individual subjects after withdrawal.

8.6 Follow-up of subjects withdrawn from treatment

If a patient is withdrawn from the study, we will still perform follow-up measurements in case the patient is willing and able to cooperate.

8.7 Premature termination of the study

The procedure in case of premature termination of the study is described in section 10.4 and 12.5.

9. SAFETY REPORTING

9.1 Temporary halt for reasons of subject safety

In accordance to section 10, subsection 4, of the WMO, the sponsor will suspend the study if there is sufficient ground that continuation of the study will jeopardise subject health or safety. The sponsor will notify the accredited METC without undue delay of a temporary halt including the reason for such an action. The study will be suspended pending a further positive decision by the accredited METC. The investigator will take care that all subjects are kept informed.
More information about temporary halt for reasons of subject safety is provided in section 10.4 and 12.5.

9.2 AEs, SAEs and SUSARs

9.2.1 Adverse events (AEs)

Adverse events are defined as any undesirable experience occurring to a subject during the study, whether or not considered related to the experimental intervention. Adverse events that are reported spontaneously by the subject or observed by the investigator or his staff and that are relevant to the study will be recorded. Relevant adverse events are possible side-effects of the DMT used and the occurrence of relapses.

Adverse Device Effect (ADE)
An ADE is an adverse event related to the use of an investigational medical device. This includes any adverse event resulting from insufficiencies or inadequacies in the instructions of use, the deployment, the installation, the operation, or any malfunction of the investigational medical device. This also includes any event that is a result of a use error or intentional misuse.

9.2.2 Serious adverse events (SAEs)

A serious adverse event is any untoward medical occurrence or effect that

- results in death;
- is life threatening (at the time of the event);
- requires hospitalisation or prolongation of existing inpatients' hospitalisation;
- results in persistent or significant disability or incapacity;
- is a congenital anomaly or birth defect; or

- any other important medical event that did not result in any of the outcomes listed above due to medical or surgical intervention but could have been based upon appropriate judgement by the investigator.

A SADE is an ADE that has resulted in any of the consequences characteristic of a serious adverse event.

The investigator will report all SAEs and SADEs to the sponsor without undue delay after obtaining knowledge of the *events*. The sponsor will report the SAEs through the web portal *ToetsingOnline* to the accredited METC that approved the protocol, within 7 days of first knowledge for SAEs that result in death or are life threatening followed by a period of maximum of 8 days to complete the initial preliminary report. All other SAEs will be reported within a period of maximum 15 days after the sponsor has first knowledge of the serious adverse events.

9.2.3 Suspected unexpected serious adverse reactions (SUSARs)

This section is not applicable since this study does not investigational medicinal products.

9.3 Annual safety report

This section is not applicable since this study does not investigational medicinal products.

9.4 Follow-up of adverse events

All AEs will be followed until they have abated, or until a stable situation has been reached. Depending on the event, follow up may require additional tests or medical procedures as indicated, and/or referral to the general physician or a medical specialist.
SAEs need to be reported until the end of study, as defined in the protocol in section 9.2.2.

9.5 Data Safety Monitoring Board (DSMB)

For optimal safety we will appoint an independent data safety monitoring board (DSMB)which will monitor trial data on a regular basis. The aim of the committee is to safeguard the interests of trial participants and assess the safety of the discontinuation of therapy during the trial. The specific role of the DSMB consists of monitoring evidence for harm due to the intervention (discontinuing medication). The DSMB may advice to terminate the trial prematurely if disease activity exceeds above mentioned thresholds (see section 10.4 for interim analyses).

The DSMB consists of 4 members who do not have conflict of interest with the sponsor or the study. In addition to the permanent members of the DSMB two external members are added to the DSMB with expertise in the relevant field of research (yet to be determined).

The DSMB will conduct interim analyses in a predetermined manner and at predetermined times (see section 10.4), to see whether the relationship between clinical benefit and burden remains acceptable to the subject during the study. After each interim analysis, the DSMB reports to the study coordinator, with reports to the METc and the study sponsor, i.e. the Board of Directors (Raad van Bestuur). The DSMB can give advice to continue, change or stop (parts of) the study. The DSMB will also ensure the quality and safety of research in the participating centers.

10. STATISTICAL ANALYSIS

All data is quantitative and will be presented in tables and graphs. Baseline data will be collected to detect any potential differences between the two investigated groups for which we have not corrected with the randomization procedure (sex and age). Possible other confounders include smoking habits, vitamin D levels but also previous disease course in terms of differences in disease duration, number of relapses prior to stability, years of use of treatment.

The primary endpoint is the number of patients with return of inflammatory disease activity after 2 years based on: a clinically confirmed relapse or any emerging subclinical disease activity proven to be due to active disease/new inflammation (defined as 3 or more lesions on T2—weighted images or 2 or more gadolinium enhancing lesions on T1-weighted post-contrast MRI) in the discontinuation group. Secondary end-points are: 1. Changes in neurological functioning (EDSS/MSFC changes including individual functional systems and MSFC subtests), 2. Individual MRI parameters (T2 and T1 post-contrast lesion numbers), 3. changes in quality of life measurements (SF-36, CIS20r, TSQM, EQ5D-5L, iMCQ and iPCQ) and 4. changes in biomarker measurements (neurofilament levels).

In the case of confirmation of our hypothesis (discontinuing medication after >5 years without evidence of inflammatory disease activity does not result in return of inflammatory disease activity), we will extend the trial with a follow-up of two years where the group that continued their treatment gets offered the possibility to discontinue under similar safety measures (with similar follow-up frequencies and endpoints as the primary trial) and including the possibility to use MS sherpa and Neurokeys.

10.1 Primary study parameter(s)

For the primary endpoint, the return of inflammatory disease activity after 2 years, a2x2 contingency table will be used to estimate the risk difference for the return of inflammatory disease activity after 2 years (yes/no) between the two groups. The risk difference will be calculated for discontinuation relative to continuation. If the lower bound of the corresponding two-sided 90% confidence interval exceeds -7.5% we conclude non-inferiority of discontinuing medication For the primary endpoint both a per protocol analysis and a intention-to-treat analysis will be performed.

The primary endpoint is the number of patients with return of inflammatory disease activity after 2 years based on: a clinically confirmed relapse or any emerging subclinical disease activity proven to be due to active disease/new inflammation (defined as 3 or more lesions on T2—weighted images or 2 or more gadolinium enhancing lesions on T1-weighted post-contrast MRI suggestive of demyelination) in the discontinuation group.

A relapse is defined according to the definition most often used in MS phase-III trials: the onset of new or recurrent symptoms that last > 24 hours, that are accompanied by new objective abnormalities on a neurological examination and that are not explained by non-MS processes such as fever, infection, severe stress or drug toxicity[21]

Furthermore, a survival analysis (with an intention-to-treat approach) regarding the time to return of inflammatory activity will be included.

10.2 Secondary study parameter(s)

For all the secondary endpoints correlation and regression analysis (either linear of logistic, depending on the type of variable) will be performed correcting for possible confounders. On the app data, classification analyses and machine learning models will be used next to the more conventional analyses.

10.3 Other study parameters

N/A

10.4 Interim analysis

We will build in a safety-strategy (go-no-go strategy) during the first 1,5 year of the study to control for emerging disease activity (and patients safety). After 6, 12 and 18 months, the number of patients with inflammatory disease activity – defined as an objectified MS relapse or 3 or more lesions on T2-weighted images of 2 or more gadolinium enhancing lesions on T1-weighted post-contrast MRI suggestive of demyelination– will be counted. If there are >2 patients *more* in the discontinuation group than in the continuation group that experience return of clinical or radiological disease activity, we will discuss premature ending of the study with the DSMB.

For this analysis, we will make a distinction between patients <55 years old and patients ≥55 years old based on mechanistic differences of recurrent inflammation in both groups. Above mentioned analysis will be done for both age groups separately. Analysis of the age

distribution of patients that experience return of disease activity will determine whether the entire age group will be advised to restart DMT or that restart may be restricted to a specific subgroup. In that case, we will continue the study for the other patients according to the protocol. In case of a premature stop of the trial for a specific age group of patients that discontinued their DMT, we will follow the patient group for which the trial was discontinued in an open label extension study with follow-up frequencies and assessments similar to the trial.

For optimal safety the DSMB will monitor the decision making on premature termination every 3 months. The DSMB may advice to terminate the trial prematurely if disease activity exceeds above mentioned thresholds. See section 9.5 for a more detailed description of the DSMB.

The procedure in case of premature termination of the study is described in section 12.5.

10.5 Cost-analysis

Alongside this trial, we will conduct a cost-effectiveness analysis and a cost-utility analysis over a 2-year follow-up. These will be performed in accordance with the recommendations of the Dutch guideline for health economic evaluations. For the cost-effectiveness analysis, the return of inflammatory disease activity (either relapse or new of enlarging lesions) will serve as the effectiveness measure. The cost-utility analysis will focus on quality of life measured with the EQ-5D-5L, which is often used in MS research. Quality-adjusted life-years (QALYs) will be calculated by multiplying the utility scores belonging to a health state by the amount of time spent in this health state using linear interpolation between time points.

In both analyses, costs will be measured from a societal perspective including health-care costs (such as the costs for a year of DMT, costs for the extra surveillance including additional medical tests as MRI, costs for visits to other medical doctors etc), direct nonmedical costs (costs that patients make for travelling to and from the hospital, private payments for extra health-care consumption etc) and indirect nonmedical costs (costs due to loss of production and short or long-term sick absence). The latter is important as previous research has shown that productivity losses are an important cost driver in severe MS. Health-care costs and direct nonmedical costs will be measured using the iMTA Medical Consumption Questionnaire (iMCQ) at 3 months, 6, months, 12 months, 18 months and 24 months. The iMCQ measures the health-care costs in the last three months. As these patients are assumed to be stable regarding their disease progression, these follow-up

moments will provide an adequate estimation of their health-care use. Primary and secondary health-care costs will be valued using Dutch standard costs. If unavailable, tariffs or costs reported by the literature will be used. Medication will be valued using www.medicijnkosten.nl whereas informal care will be valued based on the costs of household care as reported by the Centraal Administratie Kantoor.

For indirect nonmedical costs, patients will be asked for their employment situation and both short-term and long-term sick absence because of MS (or MS related treatment) using the iMTA Productivity Cost Questionnaire (iPCQ) at 3 months, 6, months, 12 months, 18 months and 24 months. The iPCQ measures productivity loss in the last four weeks which will give an adequate overview of the productivity losses between each time point as these patients are assumed to be stable. Costs of absenteeism from paid work will be calculated according to both the human capital and friction cost approach. Costs of presenteeism will be calculated by asking participants how many working hours should have been replaced due to less productivity at work. Lost productivity due to either absenteeism or presenteeism will be valued using the mean age-, and sex specific income of the Dutch population. Costs of productivity losses due to absenteeism from unpaid work and informal care will be calculated using the standard wage of a professional housekeeper. All costs will be indexed to the year at which the trial ended. Missing data on costs and effects will be imputed using multiple imputations. In addition, costs and effects will both be discounted using a 3% discount rate.

For the cost-effectiveness analysis, we will calculate incremental cost-effectiveness ratios (ICERs) which is defined as the difference in mean costs divided by the difference in mean effects between the treatment continuation group and the treatment discontinuation group. For the cost-utility analysis, we will calculate incremental cost-utility ratios (ICURs) which is the difference in mean costs divided by the difference in mean QALYs. Bootstrapping with 5,000 replications will be used to estimate 95% credibility intervals around the ICERs and ICURs. The bootstrapped cost-effect pairs will be plotted on a cost-effectiveness plane and used to estimate cost-effectiveness acceptability curves (CEACs). CEACs show the probability that the intervention is cost-effective in comparison with the control treatment for a range of ceiling ratios. The ceiling ratio is defined as the willingness-to-pay, which is the amount of money society is willing to pay to gain one unit of effect.

In a sensitivity analysis, we will repeat all analyses using a healthcare payer perspective. In this analysis, only direct healthcare costs will be included. In addition, we will conduct subgroup analysis in which we will stratify individuals based on the presence of blood-based markers predictive for return of inflammatory disease activity.

Furthermore, we will conduct a budget impact analysis. A budget impact analysis (BIA) focuses on the expected changes in the expenditure of a health care system after the adoption of a new standard of care. In this BIA, we aim to estimate the future yearly budget impact of discontinuation of first line medication in patients with long-term stable relapsing-onset from a Dutch perspective. The BIA will be performed according to the BIA framework of the International Society for Pharmacoeconomics and Outcomes Research (ISPOR). This framework consists of several standard aspects: target population, scenario distribution based on hospital types, resource utilisation, costs per unit, total costs, and sensitivity analyses.

11. ETHICAL CONSIDERATIONS

11.1 Regulation statement

The study will be conducted according to the principles of the Declaration of Helsinki (World Medical Association, 2013, Brazil) and in accordance with the Medical Research Involving Human Subjects Act (WMO) and the Good Clinical Practice guidelines.

11.2 Recruitment and consent

Patients will be informed about the study in different ways. Patients can be notified by their treating doctor during outpatient consultations at the VUmc or one of the other participating centres. In addition, a notification will be placed on the website of the MS Centre Amsterdam and the websites of the MS Vereniging Nederland (MSVN) and MS Web with information about the study. Patients can then discuss potential interest in the study with their treating neurologist. Inclusion is possible in one of the participating centers. Potential participants who express their wish to participate will receive additional information on paper about the purpose, intervention, duration and content of the study. They will also receive an informed consent form with careful and extensive information about the possible risks (according to local ethical review board requirements). In case there are any questions about the study, the study coordinator can be contacted. Moreover, an independent doctor (dr. Pijnenburg) can be contacted for additional questions.

Inclusion will take place after the written informed consent form has been returned to the trial coordinator. A copy of the informed consent form will be given to the participant and to the responsible physician in one of the participating centers in case the patient is recruited through one of the centers outside the VUmc. This consent can be revoked at any time without citing reasons. Patients will be given a minimum of 2 weeks to consider their decision. The maximum time will be as long as the inclusion of patients is ongoing and inclusion and exclusion criteria are met.

11.3 Objection by minors or incapacitated subjects (if applicable)

Not applicable, all participants will be adult and legally competent

11.4 Benefits and risks assessment, group relatedness

In the non-intervention group (continuation group) participants are expected to remain stable throughout the study period based on their proven stable status for 5 consecutive years prior to enrolment. Therefore, this group will face no potential risks and no direct benefit other than the usual medical care. The potential value of the outcome of the research will outweigh the

burden of participation for the study. Also, patients in the continuation group are offered the possibility to discontinue their medication under similar surveillance measures as the discontinuation group after 24-month follow-up period has ended.

The data on the discontinuation of therapy in long-term inflammatory stable MS patients is reassuring. One large retrospective cohort study has shown in 1200 patients that stable RRMS patients who stop treatment don't have an increased relapse rate compared to patients who continue treatment[25]. There was a slightly higher disability progression of the discontinuation group, presumably based on the discontinuation of treatment in patients with progressive MS. Also, there seems to be no rebound inflammation after discontinuation of therapy in progressive MS, and a similar relapse rate as the years prior to discontinuation[26]. Lastly, one study has showed that patients 45 years or older, or patients with a DMT intake of 4 or more years without evidence of clinical or radiological disease activity showed a high likelihood of remaining relapse-free after discontinuation and absence of contrast enhancing lesions[27]

11.5 Compensation for injury

According to article 7 from the 'Wet medisch-wetenschappelijk onderzoek met mensen' (Staatsblad 1998, 161) an insurance is obtained by the VUmc. In case of injury or death of the participants because of the study, this insurance will compensate for injury or cover the cost caused by death or injury from the participants. The insurance is obtained by the Onderlinge Waarborgmaatschappij Centramed b.a., Postbus 7374, 2701 AJ Zoetermeer. The insurance company and the insurance accede to the decree mandatory insurance for 'medisch-wetenschappelijk onderzoek met mensen (Staatsblad 2003, 266). Written information about the insurance will be provided for the participants.

11.6 Incentives (if applicable)

According to the current standard of care, the evaluation of every patient treated with immunomodulating therapy is at baseline, 12 and 24 months. Since visits are also scheduled at 3, 6 and 18 months, travel expenses and parking costs for these extra visits will be compensated. Participants will be compensated for their time and effort for study participation: they will receive a gift certificate of €25 upon study completion.

12. ADMINISTRATIVE ASPECTS, MONITORING AND PUBLICATION

12.1 Handling and storage of data and documents

Data will be handled confidentially . After collection, all data will be correctly labeled and securely stored. A subject identification code (SIC) will be used to link data to the subject. The SIC will consist of numbers and will not be based on the patient initials and birth-date. The key to the code will be kept separately from the coded data. The only people who have access to this code will be the principal investigator, the coordinating investigator and the corresponding investigator. No other people will have access to the link information. Great care will be taken to ensure that there is no link between SIC and information on which an individual can be identified. The handling of personal data in the database complies with the General Data Protection Regulation (De Algemene Verordening Gegevensbescherming) Potential data exchange with other countries will only take place after consent of the patient and handling of data will comply with the General Data Protection Regulation. The procedure for handling data includes data encryption, coding, secure storage, establishing limited access or varying levels of access to the biobank, removing identifying information from bio specimens and data. The infrastructure will consist of both hardware and software components, to prevent unauthorized access to databases.

An electronic case report form (CRF) will be developed to document the data collected in the study. This database will include demographic and patients characteristics (without birth date) and all outcomes of the study measures. Other investigators can request permission to get access to (a part) of this database for the purpose of research only, and only when the principal investigator gives permission. These investigators will not get access to the separate database which includes the participants' names, other identifiers and the SIC. All data will be stored on a computer protected with a password on the VUmc computer network. And access to the database will also be secured by a code. Only the trial coordinator and the principal investigator will know the code that gives access to the database with the link information.

After finalizing the study, the originals of all source documents will be stored for a period of 15 years in a locked room. Data that is collected and stored for the Biobank, will be stored for a period of 50 years. This period of storage has been determined to ensure that a follow-up study might be possible. In case of a follow-up study, a new protocol will be submitted to the METC and participants will have to sign a new informed consent form. Importantly, participants will only be approached for a follow-up study if they have indicated on the

informed consent form of the current study that they can be approached for a follow-up study.

The collection of data for medical research in the Netherlands is subject to the Personal Data Protection Act and in particular to the Medical Treatment Contracts Act.

Data storage Neurokeys

Data collected by NeuroKeys will be stored using Amazon AWS S3. There will be no identifiable information in this database, all keystroke and sensor data are logged with ID numbers only. A separate database, Amazon RDS (SQL server), will be used to store the verification code and personal information such as gender and year of birth, which can be used to send users push notifications. Both Amazon AWS S3 and Amazon RDS databases are located in Frankfurt, Germany, and are ISO 27001/27017/27018 compliant. All data is AES-256 encrypted in transit and at rest, a tokenization approach is used in which a sensitive data element is replaced by a non-sensitive equivalent and send by using a secure SSL link. Decryption keys are stored in a private encrypted environment. For iOS users, each time the NeuroKeys' keyboard is 'activated' (i.e. a new message is started), data of the previous keyboard session is uploaded to the database. Data of only one message is saved on the mobile phone, until a new message has started. For Android users data is uploaded to the database approximately every 4 hours.

Data storage MS sherpa

Data collected by MS sherpa will be stored using MongoDB Atlas, whose infrastructure runs on top of Amazon Web Services (ISO 27001/27017/27018 compliant) in Dublin, Ireland. General profile information (such as gender, age, length, weight, and e-mail address) is directly saved on AWS servers, in Dublin and AWS S3 in Frankfurt, Germany. Auth0 is used for user authentication and authorisation (ISO 27001/27018 compliant) and its EU office is based in London, UK. MongoDB Atlas and Auth0 achieved key compliance controls and objectives, as demonstrated by the completion of a Type 1 SOC 2 Report: Security. MongoDB, Inc. and Auth0 are also certified under the EU-US Privacy Shield. Data gathered via MS sherpa will be transferred to the database immediately after the data has been collected.

12.2 Monitoring and Quality Assurance

An independent monitor, the Clinical Research Bureau (CRB) of the VUmc, will monitor the proposed study according to Good Clinical Practice (GCP). For a selection of candidates

Informed consent is to be checked by the CRB. Besides that, source data verification is performed during the onsite monitoring. The conformity of the data used for analysis and the information in the patient files will be checked by the CRB. The intensity of the verification will be related to the risk arisen by the research. Inclusion and exclusion criteria will be checked as well as the main outcome measures. The CRB will check if the (S)AE's and SUSAR's are reported conforming the schedule as required by laws and regulations.

The quality assurance team under the leadership of a quality assurance manager (QAM) is responsible for providing an effective and efficient quality assurance system and counsel for the clinical research sites. In this quality assurance system, the QAM is responsible for ensuring appropriate global and affiliate-specific quality documents are developed and tracked, making sure they maintain an up-to-date overall inventory of all quality documents. Furthermore, the QAM and its team are responsible for ensuring all personnel involved in the clinical trial are properly qualified and trained for the job roles for which they are responsible. They are responsible for giving the personnel trainings and constantly assessing further opportunities for education and additional training. The quality assurance team is also responsible for checking compliance with the protocol, SOPs, GCP, and/or applicable regulatory requirement(s) and checking of the quality in all stages of data handling to ensure that all data are reliable and have been processed correctly. Moreover, the quality assurance team is responsible for auditing the various investigational sites.
If noncompliance with the protocol, SOPs, GCP, and/or applicable regulatory requirement(s) by and investigator/institution, or by member(s) or the sponsor's staff is detected during a quality assurance activity or audit, it is the responsibility of the QAM to report this to the trial's sponsor and principal investigator.

Quality assurance procedures:

Quality assurance is the systematic and independent examination of all clinical trial-related activities and documentations. The quality assurance procedure focuses on clinical investigator audits and audits of clinical trial documentation.

1. Document audits:

During the document audits, the quality assurance team oversees the documents that are generated before, during or at the end of the conduct of the clinical trial. For each document, a checklist is developed based on the relevant regulatory and organizational standards and SOPs. The aim of the audits is to ensure that the information and data in the documents are

complete, clear, reliable and consistent. Documents reviewed in the document audits include the clinical study protocol, the investigator's brochure and the clinical study report.

2. Clinical investigator audits:

The clinical investigator audits concern audits of the different research sites of the trial. They are performed to assess the site's regulatory compliance and clinical data quality (including adherence to the protocol). Paragraph 3 will address the procedures concerning these audits.

12.3 Amendments

Amendments are changes made to the research after a favourable opinion by the accredited METC has been given. All amendments will be notified to the METC that gave a favourable opinion. All amendments will be notified to the METC and to the competent authority.

12.4 Annual progress report

The investigator will submit a summary of the progress of the trial to the accredited METC once a year. Information will be provided on the date of inclusion of the first subject, numbers of subjects included and numbers of subjects that have completed the trial, serious adverse events/ serious adverse reactions, other problems, and amendments. The METC will also be informed on the start and end date of the trial.

12.5 Temporary halt and (prematurely) end of study report

The investigator/sponsor will notify the accredited METC of the end of the study within a period of 8 weeks. The end of the study is defined as the last patient's last visit.
The sponsor will notify the METC immediately of a temporary halt of the study, including the reason of such an action.
In case the study is ended prematurely, the sponsor will notify the accredited METC within 15 days, including the reasons for the premature termination.
Within one year after the end of the study, the investigator/sponsor will submit a final study report with the results of the study, including any publications/abstracts of the study, to the accredited METC.

12.6 Public disclosure and publication policy

This study will be registered in het Nederlands Trial Register (NTR) http://www.trialregister.nl and www.clinicaltrials.gov. Publication will be in accordance with the basic principles of

CCMO statement on publication policy. The results will be presented at (inter)national scientific meetings. The results will be published in a medical scientific journal. In none of the publication forms, participant identity will be disclosed.

13. STRUCTURED RISK ANALYSIS

13.1 Potential issues of concern

Paragraph 13.1 is not applicable.

13.2 Synthesis

The intervention in this study is the discontinuation of previously used DMT. No new products or agents are administered, nor will there be any dosage adjustments in the group that will continue their therapy. The specific DMT's that patients use prior to discontinuation, and that are used in the control group are all registered with the authorities and widely used for this specific indication. Although previous studies suggest that the risk of return of inflammatory activity after discontinuing DMT will be low in long-term stable RRMS patients (as also described in section 1), this is the main risk of the intervention. To monitor return of inflammatory activity, a safety strategy is built in, which is described in section 10.4. If safety criteria are exceeded, the study will be discontinued and DMT's will be reinitiated (in one patient group or in all patients, see section 10.4). A DSMB is appointed that will monitor the decision making on premature termination every 3 months (section 9.5).

14. REFERENCES

1 Goodin, D. S. The epidemiology of multiple sclerosis: insights to disease pathogenesis. *Handb Clin Neurol* **122**, 231-266, doi:10.1016/B978-0-444-52001-2.00010-8 (2014).

2 Weinshenker, B. G. Natural history of multiple sclerosis. *Ann Neurol* **36 Suppl**, S6-11 (1994).

3 Reich, D. S., Lucchinetti, C. F. & Calabresi, P. A. Multiple Sclerosis. *N Engl J Med* **378**, 169-180, doi:10.1056/NEJMra1401483 (2018).

4 Giovannoni, G. *et al.* Is it time to target no evident disease activity (NEDA) in multiple sclerosis? *Mult Scler Relat Disord* **4**, 329-333, doi:10.1016/j.msard.2015.04.006 (2015).

5 Arnold, D. L. *et al.* Peginterferon beta-1a improves MRI measures and increases the proportion of patients with no evidence of disease activity in relapsing-remitting multiple sclerosis: 2-year results from the ADVANCE randomized controlled trial. *BMC Neurol* **17**, 29, doi:10.1186/s12883-017-0799-0 (2017).

6 Havrdova, E. *et al.* Effect of delayed-release dimethyl fumarate on no evidence of disease activity in relapsing-remitting multiple sclerosis: integrated analysis of the phase III DEFINE and CONFIRM studies. *Eur J Neurol* **24**, 726-733, doi:10.1111/ene.13272 (2017).

7 Miller, A. E. *et al.* Oral teriflunomide for patients with a first clinical episode suggestive of multiple sclerosis (TOPIC): a randomised, double-blind, placebo-controlled, phase 3 trial. *Lancet Neurol* **13**, 977-986, doi:10.1016/S1474-4422(14)70191-7 (2014).

8 Freedman, M. S. *et al.* Moving toward earlier treatment of multiple sclerosis: Findings from a decade of clinical trials and implications for clinical practice. *Mult Scler Relat Disord* **3**, 147-155, doi:10.1016/j.msard.2013.07.001 (2014).

9 Ramsaransing, G. S. & De Keyser, J. Benign course in multiple sclerosis: a review. *Acta Neurol Scand* **113**, 359-369, doi:10.1111/j.1600-0404.2006.00637.x (2006).

10 Sartori, A., Abdoli, M. & Freedman, M. S. Can we predict benign multiple sclerosis? Results of a 20-year long-term follow-up study. *J Neurol* **264**, 1068-1075, doi:10.1007/s00415-017-8487-y (2017).

11 Benedikz, J. *et al.* The natural history of untreated multiple sclerosis in Iceland. A total population-based 50 year prospective study. *Clin Neurol Neurosurg* **104**, 208-210 (2002).

12 Perini, P., Tagliaferri, C., Belloni, M., Biasi, G. & Gallo, P. The HLA-DR13 haplotype is associated with "benign" multiple sclerosis in northeast Italy. *Neurology* **57**, 158-159 (2001).

13 Johnson, K. M., Zhou, H., Lin, F., Ko, J. J. & Herrera, V. Real-World Adherence and Persistence to Oral Disease-Modifying Therapies in Multiple Sclerosis Patients Over 1 Year. *J Manag Care Spec Pharm* **23**, 844-852, doi:10.18553/jmcp.2017.23.8.844 (2017).

14 Lattanzi, S. *et al.* Persistence to oral disease-modifying therapies in multiple sclerosis patients. *Journal of neurology* **264**, 2325-2329, doi:10.1007/s00415-017-8595-8 (2017).

15 Lanzillo, R. *et al.* A multicentRE observational analysiS of PErsistenCe to Treatment in the new multiple sclerosis era: the RESPECT study. *Journal of neurology* **265**, 1174-1183, doi:10.1007/s00415-018-8831-x (2018).

16 Vermersch, P. *et al.* Teriflunomide versus subcutaneous interferon beta-1a in patients with relapsing multiple sclerosis: a randomised, controlled phase 3 trial. *Multiple sclerosis (Houndmills, Basingstoke, England)* **20**, 705-716, doi:10.1177/1352458513507821 (2014).

17 Balak, D. M. *et al.* Prevalence of cutaneous adverse events associated with long-term disease-modifying therapy and their impact on health-related quality of life in patients with multiple sclerosis: a cross-sectional study. *BMC neurology* **13**, 146, doi:10.1186/1471-2377-13-146 (2013).

18 Rommer, P. S. & Zettl, U. K. Managing the side effects of multiple sclerosis therapy: pharmacotherapy options for patients. *Expert opinion on pharmacotherapy* **19**, 483-498, doi:10.1080/14656566.2018.1446944 (2018).

19 Lee Mortensen, G. & Rasmussen, P. V. The impact of quality of life on treatment preferences in multiple sclerosis patients. *Patient Prefer Adherence* **11**, 1789-1796, doi:10.2147/ppa.S142373 (2017).

20 La Mantia, L., Munari, L. M. & Lovati, R. Glatiramer acetate for multiple sclerosis. *Cochrane Database Syst Rev*, Cd004678, doi:10.1002/14651858.CD004678.pub2 (2010).

21 Gold, R. *et al.* Placebo-controlled phase 3 study of oral BG-12 for relapsing multiple sclerosis. *N Engl J Med* **367**, 1098-1107, doi:10.1056/NEJMoa1114287 (2012).

22 Fox, R. J. *et al.* Placebo-controlled phase 3 study of oral BG-12 or glatiramer in multiple sclerosis. *N Engl J Med* **367**, 1087-1097, doi:10.1056/NEJMoa1206328 (2012).

23 Sejbaek, T., Nybo, M., Petersen, T. & Illes, Z. Real-life persistence and tolerability with dimethyl fumarate. *Mult Scler Relat Disord* **24**, 42-46, doi:10.1016/j.msard.2018.05.007 (2018).

24 CIBG; ministerie van Volksgezondheid, W. e. S. *Prijsvorming*, <www.farmatec.nl> (z.d.).

25 Kister, I. *et al.* Discontinuing disease-modifying therapy in MS after a prolonged relapse-free period: a propensity score-matched study. *J Neurol Neurosurg Psychiatry* **87**, 1133-1137, doi:10.1136/jnnp-2016-313760 (2016).

26 Bonenfant, J. *et al.* Can we stop immunomodulatory treatments in secondary progressive multiple sclerosis? *Eur J Neurol* **24**, 237-244, doi:10.1111/ene.13181 (2017).

27 Bsteh, G. *et al.* Discontinuation of disease-modifying therapies in multiple sclerosis - Clinical outcome and prognostic factors. *Mult Scler* **23**, 1241-1248, doi:10.1177/1352458516675751 (2017).

28 O'Rourke, K. E. & Hutchinson, M. Stopping beta-interferon therapy in multiple sclerosis: an analysis of stopping patterns. *Mult Scler* **11**, 46-50, doi:10.1191/1352458505ms1131oa (2005).

29 Rio, J. *et al.* Factors related with treatment adherence to interferon beta and glatiramer acetate therapy in multiple sclerosis. *Mult Scler* **11**, 306-309, doi:10.1191/1352458505ms1173oa (2005).

30 CBO richtlijn (2012) Immunomodulerende en immunosuppressieve behandeling bij multipele sclerose. (2012).

31 Kennisagenda neurologie 2017: https://gallery.mailchimp.com/29087cdad5c58a12bd346e83f/files/3b5692f1-3840-48e2-99e7-405edb9a895f/Kennisagenda_Neurologie_eindversie_16_12_2017.pdf.

32 Kister, I. & Corboy, J. R. Reducing costs while enhancing quality of care in MS. *Neurology* **87**, 1617-1622, doi:10.1212/WNL.0000000000003113 (2016).

33 Uher, T. *et al.* Is no evidence of disease activity an achievable goal in MS patients on intramuscular interferon beta-1a treatment over long-term follow-up? *Mult Scler* **23**, 242-252, doi:10.1177/1352458516650525 (2017).

34 Rotstein, D. L., Healy, B. C., Malik, M. T., Chitnis, T. & Weiner, H. L. Evaluation of no evidence of disease activity in a 7-year longitudinal multiple sclerosis cohort. *JAMA Neurol* **72**, 152-158, doi:10.1001/jamaneurol.2014.3537 (2015).

35 De Stefano, N. *et al.* Long-term assessment of no evidence of disease activity in relapsing-remitting MS. *Neurology* **85**, 1722-1723, doi:10.1212/WNL.0000000000002105 (2015).

36 Gray, O., McDonnell, G. & Hawkins, S. Tried and tested: the psychometric properties of the multiple sclerosis impact scale (MSIS-29) in a population-based study. *Mult Scler* **15**, 75-80, doi:10.1177/1352458508096872 (2009).

37 Pfennings, L. E. *et al.* A health-related quality of life questionnaire for multiple sclerosis patients. *Acta Neurol Scand* **100**, 148-155 (1999).

38 Rietberg, M. B., Van Wegen, E. E. & Kwakkel, G. Measuring fatigue in patients with multiple sclerosis: reproducibility, responsiveness and concurrent validity of three Dutch self-report questionnaires. *Disabil Rehabil* **32**, 1870-1876, doi:10.3109/09638281003734458 (2010).

39 Eagle, T. *et al.* Treatment satisfaction across injectable, infusion, and oral disease-modifying therapies for multiple sclerosis. *Mult Scler Relat Disord* **18**, 196-201, doi:10.1016/j.msard.2017.10.002 (2017).

40 Kobelt, G., Berg, J., Lindgren, P. & Jonsson, B. Costs and quality of life in multiple sclerosis in Europe: method of assessment and analysis. *Eur J Health Econ* **7 Suppl 2**, S5-13, doi:10.1007/s10198-006-0365-y (2006).

41 Janssen, M. F. *et al.* Measurement properties of the EQ-5D-5L compared to the EQ-5D-3L across eight patient groups: a multi-country study. *Qual Life Res* **22**, 1717-1727, doi:10.1007/s11136-012-0322-4 (2013).

42 Bouwmans, C. *et al.* The iMTA Productivity Cost Questionnaire: A Standardized Instrument for Measuring and Valuing Health-Related Productivity Losses. *Value Health* **18**, 753-758, doi:10.1016/j.jval.2015.05.009 (2015).

RESEARCH PROTOCOL

The safety and cost-effectiveness of discontinuing disease-modifying therapies in stable relapsing-onset multiple sclerosis (DOT-MS): a randomized rater-blinded multicenter trial.

Version 5, December 2020

TABLE OF CONTENTS

1. INTRODUCTION AND RATIONALE 11
2. OBJECTIVES 15
3. STUDY DESIGN 17
4. STUDY POPULATION 18
4.1 Population (base) 18
4.2 Inclusion criteria 19
4.3 Exclusion criteria 20
4.4 Sample size calculation 20
5. TREATMENT OF SUBJECTS 21
5.1 Investigational product/treatment 21
5.2 Use of co-intervention 21
5.3 Escape medication 21
6. INVESTIGATIONAL PRODUCT 21
7. NON-INVESTIGATIONAL PRODUCT 21
8. METHODS 21
8.1 Study parameters/endpoints 21
8.1.1 Main study parameter/endpoint 21
8.1.2 Secondary study parameters/endpoints (if applicable) 22
8.1.3 Other study parameters (if applicable) 23
8.2 Randomisation, blinding and treatment allocation 23
8.3 Study procedures 24
8.4 Withdrawal of individual subjects 28
8.4.1 Specific criteria for withdrawal (if applicable) 28
8.5 Replacement of individual subjects after withdrawal 28
8.6 Follow-up of subjects withdrawn from treatment 28
8.7 Premature termination of the study 28
9. SAFETY REPORTING 29
9.1 Temporary halt for reasons of subject safety 29
9.2 AEs, SAEs and SUSARs 29
9.2.1 Adverse events (AEs) 29
9.2.2 Serious adverse events (SAEs) 29
9.2.3 Suspected unexpected serious adverse reactions (SUSARs) 30
9.3 Annual safety report 30
9.4 Follow-up of adverse events 30
9.5 Data Safety Monitoring Board (DSMB) 30
10. STATISTICAL ANALYSIS 32
10.1 Primary study parameter(s) 32
10.2 Secondary study parameter(s) 33
10.3 Other study parameters 33
10.4 Interim analysis 33
11. ETHICAL CONSIDERATIONS 37

11.1 Regulation statement 37
11.2 Recruitment and consent..37
11.3 Objection by minors or incapacitated subjects (if applicable)..................................37
11.4 Benefits and risks assessment, group relatedness..37
11.5 Compensation for injury 38
11.6 Incentives (if applicable)...38
12. ADMINISTRATIVE ASPECTS, MONITORING AND PUBLICATION 39
12.1 Handling and storage of data and documents 39
12.2 Monitoring and Quality Assurance..40
12.3 Amendments...42
12.4 Annual progress report...42
12.5 Temporary halt and (prematurely) end of study report...42
12.6 Public disclosure and publication policy..42
13. STRUCTURED RISK ANALYSIS...44
13.1 Potential issues of concern..44
13.2 Synthesis 44
14. REFERENCES 45

LIST OF ABBREVIATIONS AND RELEVANT DEFINITIONS

9HPT	9-Hole Peg Test
ABR	General Assessment and Registration form (ABR form), the application form that is required for submission to the accredited Ethics Committee; in Dutch: Algemeen Beoordelings- en Registratieformulier (ABR-formulier)
AE	Adverse Event
AR	Adverse Reaction
CA	Competent Authority
CCMO	Central Committee on Research Involving Human Subjects; in Dutch: Centrale Commissie Mensgebonden Onderzoek
CIS	Clinically Isolated Syndrome
CIS20r	Checklist Individual Strength
CV	Curriculum Vitae
DMT	Disease Modifying Therapy
DSMB	Data Safety Monitoring Board
EDSS	Expanded Disability Status Scale
EQ-5D-5L	EuroQol 5 Dimensions Questionnaire
EU	European Union
FLAIR	Fluid Attenuation Inversion Recovery
GCP	Good Clinical Practice
GDPR	General Data Protection Regulation; in Dutch: Algemene Verordening Gegevensbescherming (AVG)
IC	Informed Consent
iMCQ	Medical Consumption Questionnaire
iPCQ	Productivity Costs Questionnaire
METC	Medical research ethics committee (MREC); in Dutch: medisch-ethische toetsingscommissie (METC)
MRI	Magnetic Resonance Imaging
MS	Multiple Sclerosis
MSFC	Multiple Sclerosis Functional Composite
MSIS-29	Multiple Sclerosis Impact Scale
RRMS	Relapsing Remitting Multiple Sclerosis
(S)AE	(Serious) Adverse Event
SDMT	Symbol Digits Modalities Test

**Sponsor**	**The sponsor is the party that commissions the organisation or performance of the research, for example a pharmaceutical company, academic hospital, scientific organisation or investigator. A party that provides funding for a study but does not commission it is not regarded as the sponsor, but referred to as a subsidising party.**
**SF-36**	**Short Form Health Survey**
**SPMS**	**Secondary Progressive Multiple Sclerosis**
**SUSAR**	**Suspected Unexpected Serious Adverse Reaction**
**T25fW**	**Timed 25-foot Walk**
**TSQM**	**Treatment Satisfaction Questionnaire for Medication**
**UAVG**	**Dutch Act on Implementation of the General Data Protection Regulation; in Dutch: Uitvoeringswet AVG**
**WMO**	**Medical Research Involving Human Subjects Act; in Dutch: Wet Medisch-wetenschappelijk Onderzoek met Mensen**

SUMMARY

Rationale: The past few years, several new effective drugs have come onto the market for the treatment of relapsing remitting MS (RRMS), all of which have potentially serious side effects. The arrival of these drugs has led to a new aim for treating MS patients: achieving a status of complete clinical and radiological control of inflammatory events, also described as a status of no evident disease activity (NEDA-3). With these adjusted goals, medication is often started at an earlier stage and the disease is treated more aggressively. This leads to better control of the disease, but also to increased exposure to possible (serious) side effects. A considerable group of patients with a fully stable-disease under treatment merely have a benign or less inflammatory disease course rather than a necessity for treatment to prevent inflammation. This raises the question whether and when patients who have been stable under medication for years can safely discontinue the treatment. The hypothesis of this study is that discontinuing medication after >5 years without evidence of inflammatory disease activity does not result in return of inflammatory disease activity.

Objective: The aim of this study is to identify whether it is possible to safely discontinue treatment in MS patients who have shown no evidence of active inflammation in the years prior to inclusion clinically and/or radiologically. The secondary objectives address the questions whether the discontinuation of first-line treatment has an effect on disability progression and whether the discontinuation of first-line treatment improves the quality of life for the patient and if this can be measured in a daily setting using digital biomarkers.. Furthermore, blood collections will be included to assess whether it is possible to retrospectively predict possible return of inflammatory activity with biomarkers such as neurofilament light (NFL) or patient characteristics such as disease activity prior to disease modifying therapy (DMT). In case of emerging disease activity after the cessation of therapy we will assess if reinitiation will lead to NEDA again, and if there are long-term consequences. If possible, post-hoc analysis are performed for the different types of treatment compounds.

Study design: Multi-center randomized and controlled, rater-blinded trial in the Netherlands. 130 patients with relapse onset MS will be assigned to either discontinue the previously used DMT or to continue their DMT.

Study population: MS patients who are treated with one of the first-line treatments (any of the interferons, glatiramer acetate, dimethylfumarate, teriflunomide) and who had a complete absence of inflammatory activity (no relapses, no new-T2 lesions and no contrast-enhancing lesions) for 5 consecutive years under first-line treatment will be eligible for inclusion.

Intervention (if applicable): discontinuation of the previously used DMT.

Main study parameters/endpoints: The primary endpoint is the return of inflammatory disease activity after 2 years: either relapses, new or enlarging lesions on T2-weighted MRI

and gadolinium-enhancing lesions on post-contrast T1-weighted MRI. Secondary end-points are EDSS and MSFC progression (combined: EDSS plus), number of relapses, individual MRI-parameters (such as lesion numbers), quality of life measurements, optical coherence tomography (OCT) and eye movement measurements, and (digital) biomarker measurements.

Nature and extent of the burden and risks associated with participation, benefit and group relatedness: The burden of participation consists of assessments during visits at baseline, 3, 6, 12, 18 and 24 months. Every follow-up visit of both patient groups will consist of clinical and radiological measurements, quality of life questionnaires and blood collection. Additional data will be collected via mobile applications MS sherpa and Neurokeys, that will be installed on patients' smartphones. For this, patients will be asked to perform tasks on their phones, measuring walking ability, hand function, cognition and fatigue. This takes approximately five minutes every two weeks and is performed at home. The data on the discontinuation of therapy in long-term inflammatory stable MS patients is reassuring. One large retrospective cohort study has shown in 1200 patients that stable RRMS patients who stop treatment don't have an increased relapse rate compared to patients who continue treatment (Kister et al). There was a slightly higher disability progression of the discontinuation group, presumably based on the discontinuation of treatment in patients with progressive MS. Also, there seems to be no rebound inflammation after discontinuation of therapy in progressive MS, and a similar relapse rate as the years prior to discontinuation (Bonenfant et al). When disease activity emerges, patients are treated according to the current standard of care. The discontinuation of medication can be beneficial for the patient, as the side effects of medications can be significant for some patients.

1. INTRODUCTION AND RATIONALE

In recent decades, the therapeutic landscape of multiple sclerosis (MS) has changed dramatically. Coming from an era where virtually no therapies were available, there are currently more than 12 first- and second-line disease modifying treatment (DMT) options for the prevention of focal inflammatory demyelinating lesions in the brain and spinal cord. Clinically MS can be devastating; it affects roughly 1 in 1000 persons in the Netherlands usually diagnosed in the prime of their lives with a mean age at diagnosis around 30[1]. Two major disease phenotypes exist. The most important is relapsing-onset MS (80% MS patients), including patients with a clinically isolated syndrome (CIS), relapsing remitting MS (RRMS) and secondary progressive MS (SPMS). The other 20% suffers from a primary progressive MS (PPMS), a disease phenotype with more distinct neurodegeneration. Untreated, 50% of patients will need assistance walking small distances after 10-20 years after diagnosis[2].The main pathological hallmark in the first stages (CIS and RRMS) is recurrent focal inflammation of the brain and spinal cord leading to demyelination[3]. The first years after the diagnosis patients usually experience 2-3 relapses annually. The severity of neurological disability depends on the localisation of the inflammation. With increasing age, the amount of inflammation tends to diminish and an unknown neurodegenerative pathology drives the disease course. Clinically there is a progressive decline in neurological functioning; i.e. the “progressive” phase (or the secondary progressive (SPMS) disease course).

There has been great change in the timing of diagnosing MS and evaluating disease activity with the introduction of magnetic resonance imaging (MRI). In the early days the disease course was solely evaluated based on new relapses and/or progression of disability. Now, the arrival of MRI has led to a revised aim for treating MS patients: achieving a status of “no evident disease activity” (NEDA-3); complete clinical and radiological control of inflammatory events and no significant increase in diseability[4]. To date an estimated 10% of patients have a status of NEDA-3 under long-term first-line therapy, implying a full control of focal inflammation in these patients[5-7]. In recent years there has been a growing trend of starting treatment earlier and to treat more aggressively, partly based on the concept of NEDA. Treatment is almost always initiated directly after diagnosis but sometimes even before a definite diagnosis of RRMS is made[8]. There is however a substantial percentage of patients with a more benign disease course, described in a very broad range of 6-64% of MS patients[9-12]. At the moment of diagnosis, it is not known how the disease course will develop and based on the substantial group of “benign” MS cases, it is likely that a considerable group of patients that have long-term and fully stable-disease under DMT is unnecessarily

treated. In addition, there is the group of patients who experience disease progression despite their therapy. Also, these patients probably do not benefit from their therapy.

Exposure to treatment is not without risks and costs. Side-effects of MS medication are frequently present. Data on the proportion of patients discontinuing first-line DMT's demonstrates a discontinuation rate of 20-40% during an observation period over 1 year, with the occurrence of side effects and poor tolerability as the most common reason for drug withdrawal[13-15]. A large proportion of the patient population is confronted with side effects for both oral (teriflunomide, dimethylfumarate) and injectable (interferons, glatiramer acetate) DMT's. For example, the results of the post-approval clinical trials on the safety of teriflunomide compared to interferon beta-1a showed that 93-96% of the patients experienced side effects[16]. Each injectable first-line DMT can lead to mild cutaneous adverse events such as erythema and swelling, but also to more severe and persisting effects such as lipoatrophy, infections and even necrosis. Patients with a cutaneous reaction appeared to have a lower dermatology-specific health-related quality of life[17]. Serious events rates were also high ranging from 7% (interferon beta-1a) to 12% (teriflunomide)[16].

Each first-line DMT has a different mode of administration and specific side-effects. Interferons are administered biweekly s.c. (Plegridy), weekly i.m. (Avonex), second daily s.c. (Betaferon) or thrice weekly s.c. (Rebif). Flu-like symptoms are the most often reported side effects of interferon ß injections and are particularly challenging for MS patients[18,19]. But also allergic reactions, elevated liver enzymes leading to severe hepatic injury, thyroid autoimmunity, hypothyroidism and hematologic abnormalities might occur[18]. Glatiramer acetate (Copaxone) is injected subcutaneously daily or thrice weekly. Patterned reactions are most commonly reported in patients using glatiramer acetate, consisting of flushing, chest pain, palpitations, urticaria, anxiety and dyspnoea with a relative risk of 3.27. This patterned reaction unpredictably occur within minutes of injection and spontaneously resolve before 30 minutes[20]. Teriflunomide (Aubagio) is an oral drug and is administered daily. Hair thinning, increased blood pressure, fatigue, diarrhoea, sensory disturbances, elevated liver enzymes, and renal failure are adverse effects that have been reported in patients using teriflunomide[18]. The fourth agent in the first-line DMT group, dimethylfumarate (Tecfidera) is taken orally twice daily. Clinical phase 3 trials reported mild or moderate flushing and gastrointestinal (GI) adverse events, 36% and 42%, respectively as most common adverse effects[21-23]. Consequently, this led to treatment discontinuation in both trials. Serious side effects include urosepsis (interferons), hepatotoxicity (glatiramer acetate), but also progressive multifocal leukoencephalopathy (dimethyl fumarate), which are all potentially lethal.

Furthermore, there is a great burden of costs to society. The costs for a year of first-line immunomodulating drugs range from 12.000 – 15.000 euros annually[24]. The discontinuation of therapy in appropriate patients therefore also has a very significant effect with a potential cost-reduction of 2-2.5 million euros annually in The Netherlands. Costs due to side-effects (such as treatment and absence of work) are not even included here.

The data on the discontinuation of therapy in long-term inflammatory stable MS patients is reassuring. One large retrospective cohort study has shown in 1200 patients that stable RRMS patients who stop treatment don't have an increased relapse rate compared to patients who continue treatment[25]. There was a slightly higher disability progression of the discontinuation group, presumably based on the discontinuation of treatment in patients with progressive MS. Also, there seems to be no rebound inflammation after discontinuation of therapy in progressive MS, and a similar relapse rate as the years prior to discontinuation[26]. Lastly, one study has showed that patients 45 years or older, or patients with a DMT intake of 4 or more years without evidence of clinical or radiological disease activity showed a high likelihood of remaining relapse-free after discontinuation and absence of contrast enhancing lesions[27]. All studies where however hampered by either its retrospective nature, or incompleteness on for examples reasons for discontinuation or a sufficiently matched control group. Obviously, definite conclusions can only be drawn after a well-designed controlled trial. Currently, two trials with a similar question to ours are underway (DISCOMS; NCT03073603 and STOP-I-SEP; NCT03653273). These studies are different compared to our proposal in that the inclusion criteria only allow for older patients (older than 55 years and older than 50 years respectively) to be included.

The question of whether or not to discontinue therapy is increasingly present during our outpatient clinics [28,29]. As stated, an estimated 10% of patients have a status of NEDA-3 under long-term first-line DMT and could benefit from the results of this study. To give an indication of the number of patients concerned: in the Netherlands alone an estimated number of 7000-7500 patients with MS use first-line DMT, which means 700-750 patients would be eligible for this study and would benefit from results of this study (numbers are based on market shares and sales of each DMT, data not published). An enquiry amongst Dutch MS neurologists (data not published) shows that every neurologist struggles with the question on average 5-10 times per year. The Dutch 2012 CBO guideline " Multiple Sclerosis" recommends the discontinuation of DMT in patients who experience severe side-effects and who had secondary progressive MS for at least 3 years[30] but this is solely based on expert opinion. Also, internationally there are no guidelines guiding patients and neurologists in this question. Very recently in 2017, the committee of care evaluation of

neurology (‘Zorgevaluatie Neurologie’ (ZEN), part of the ‘Dutch society of Neurologists (Nederlandse Vereniging voor Neurologie; NVN) and supported by the Dutch Federation of medical specialists (FMS) and the Dutch Federation of Patients) has ranked this lack of evidence regarding the discontinuation of immunomodulating drugs in MS one of the most important science/knowledge gaps within daily neurological practice in the Netherlands. It listed this topic in its “ Kennisagenda 2018-2022” which prioritizes the 12 most urgent topics for scientific research in the field of neurology in the coming 4 years to improve the effectiveness and safety of our daily medical practice[31]. We have validated this support by means of a questionnaire amongst all 88 members of the MS Taskforce (Part of the NVN). All (100%) respondents (response-rate was 49%) indicated that they found the issue frequently present in their clinics and important tot research. Also, internationally there is strong call for more evidence-based guidelines and consensus regarding the discontinuation of treatment in long-term stable MS patients[32]. This underlines the national (and international) need for systematic research regarding this subject.

2. OBJECTIVES

With this study we will bring first evidence to this important issue by identifying whether it is possible to safely discontinue treatment in MS patients who have shown no evidence of active inflammation in the years prior to inclusion. Importantly, we include quality of life measurements to evaluate if this also translates to an increased perception of health. If present, it will optimize the treatment paradigm for individual MS patients through identifying unnecessary exposure to medication while proving the discontinuation of medication to be safe, and beneficial for the daily lives of individual patients in terms of an increased quality of life. In addition, it greatly benefits the general society since it also provides a more efficient use of funds with the significant cost-reduction it brings. The impact of the trial is immediate, within 5 years, and since all large MS centers will participate there is a direct line into the daily offices of treating neurologists, and into the development of adjusted guidelines regarding the treatment of MS. Most importantly, every possible result deriving from this trial will have a significant impact on (inter)national treatment strategies. Besides the primary question of the evaluation of safety we also incorporate validated measures of quality of life and disease perception to evaluate potential changes in quality of life. Furthermore, we include blood collections for the monitoring of neurofilament light (currently the only validated and clinically applied biomarker for the return of inflammatory activity) that could potentially serve as indicator for subclinical return of inflammatory activity. In addition, we will include optical coherence tomography (OCT) and eye movement measurements for patients participating in Amsterdam UMC. OCT measurements (especially retinal nerve fiber layer (RNFL) thickness) are known to be associated with disability in MS patients, and thus can be seen as a measure for disease progression.

Primary outcome measures used in this study are clinical and radiological outcome measures that are already part of routine clinical practice, which ensures that results of this trial can be directly and easily implemented in standard clinical care. In addition to these outcome measures, it would be of interest to determine optimal measurements for potential return of disease activity. A promising method in this regard is the use of mobile applications on patients' smartphones, because of their non-invasive nature (in contrast to for example MRI-scans) and the potential for more continuous measurements in a real-world setting (i.e. at home). Measurements with mobile applications MS sherpa and Neurokeys will be included for a subgroup of patients (depending on the availability of a smartphone), to investigate if return of inflammatory disease activity can be measured with these applications. These mobile applications will be installed on patients' smartphones.

See also methods section for a full description.

Research questions

Primary research question:

Can we safely discontinue first-line medication in MS patients with long-term absence of inflammation, without the return of *inflammatory disease activity* clinically and radiologically?

Other research questions:

- Does the discontinuation of first-line treatment have an effect on disability progression?
- Does the discontinuation of first-line treatment improve the quality of life for the patient?
- What is the effect of discontinuation of first-line treatment on individual MRI outcome measures such as lesion load and atrophy measurements?
- Is it possible to predict possible return of inflammatory activity with biomarkers such as neurofilament light (NFL) or patient characteristics such as disease activity prior to DMT?
- In case of emerging disease activity after cessation of DMT, will a restart of DMT result in NEDA again and if so, how long does it take?
- In case of emerging disease activity after treatment cessation, are there any differences between the different DMT compounds?
- What is the cost-effectiveness of discontinuation of DMT in The Netherlands?
- Is discontinuation of first-line DMT associated with OCT measurements and eye movement measurements?
- Is it possible to detect and predict (return of) inflammatory disease activity and disease progression with digital biomarkers using mobile applications such as MS sherpa and Neurokeys?

3. STUDY DESIGN

The study design is a multi-center rater-blinded randomized controlled trial in the Netherlands. The project will take place over period of 5 years (60 months) depending on the progress of inclusion. The lead and monitoring of the trial will be performed by the MS center Amsterdam (MSCA). Participating centers are listed in an appendix (I1).

Study-population: 130 relapse onset MS patients who are treated with one of the first-line treatments (any of the interferons, glatiramer acetate, dimethylfumarate, teriflunomide) *and* who had a complete absence of objective inflammatory activity (no objectified relapses, no significant number (2 or more) of new-T2 lesions and no contrast-enhancing lesions) for 5 consecutive years under first-line treatment will be eligible for inclusion. Patients may not have switched between first-line drugs over the two years prior to inclusion. If a switch has occurred this should not have been due to ineffectivity of the first DMT but due to side-effects or by a personal preference of the patient (such as the wish to switch to oral therapies). In the case of previous use of interferons patients must be negative for neutralizing antibodies (NAbs). Inclusion will take place after informed consent. This will be obtained after careful and extensive information about the possible risks according to local ethical review board requirements.

Intervention: The intervention is the discontinuation of the previously used DMT. Based on the pharmacological profile of the abovementioned drugs that are eligible for inclusion, there is no need for a tapering of dosage before complete discontinuation

Follow-up frequency: Patients in the continuation group are expected to remain stable throughout the study period based on their proven stable status for 5 consecutive years prior to enrolment. The minimum evaluation that must take place should have a frequency according to the current standard of care (yearly evaluation of every patient treated with immunomodulating therapy; i.e. baseline (BL), 12 and 24 months). Timely recognition of recurrence of any (subclinical) disease activity in the discontinuation group is secured by more frequent clinical and radiological assessment during extra routine study visits at 3, 6 and 18 months. For optimal comparability and to overcome potential bias, both groups will be followed with a complete assessment including MRI at BL, 3, 6, 12, 18 and 24 months.

After these two years of follow-up, the patients in the continuation group are offered to discontinue their DMT with a follow-up similar to the discontinuation group.

4. STUDY POPULATION

4.1 Population (base)

We will include 130 participants with the diagnosis relapse onset MS with a minimum age of 18 years. 65 patients will be assigned to the discontinuation group and 65 patients will be assigned to the continuation group. MS patients will be recruited through the VUmc outpatient clinic and the outpatient neurology departments of all participating centers. Because this research has a multicenter national design, MS patients of the majority of regions in the Netherlands will be included, including urban as well as rural areas. We believe that represents a valid cross-section of the average MS patient population. It is important to note however, as MS affects 2-2.5x as many women as men we expect to include more women than men. We will include a randomization algorithm to match both groups for sex to exclude potential bias.

We have estimated 700-750 patients to be potentially eligible for inclusion in The Netherlands. Currently an estimated number of 7000-7500 patients use first-line DMT for their MS. This number is estimated on data that was provided by all different pharmaceutical companies of each of the first-line therapies. Numbers are based on market shares and actual sales of each of the treatments (data not published).

The percentage of “stability” derives from the treatment effects described in the pivotal phase III trials of the currently available first-line treatments and 3 cohort studies with a longer follow-up that the average 2 years in the phase III trials. For our calculation of possible eligible patients, we have used the most “negative” scenario based on the results of these trials. The most recent phase III studies report on NEDA as outcome measure (Havrdova et al 2017, Arnold et al 2017), reflecting the new treatment concept of MS: complete stabilization of the disease process. In those cohorts NEDA ranges from 25-40% (Havrdova et al 2017, Arnold et al 2014 & 2017, Miller et al 2014, Nygaard et al 2015). It is important to note however, that the concept of NEDA also includes progressive neurological decline (such as is due to progressive disease and neurodegeneration). The percentage of patients that is free from inflammation is therefore somewhat underestimated in the numbers of these studies. Furthermore, most studies comprise on average a relatively short observation period of 2 years.

Three cohort studies exist with a longer follow-up. De Stefano et al, Uher et al and Rotstein et al published cohort studies that describe a longitudinal follow-up of NEDA status of ~ 200 patients for 7-10 years[33-35]. They showed that a fairly low percentage of patients remained

NEDA after 5 years (10-15%). In the cases of emerging inflammatory activity under treatment, most patients showed disease activity in the first 1-2 years after the start of treatment. Only a small minority of patients lose their NEDA status after 5 years (4%). It is very important to note that in the Rotstein-study the loss of NEDA status was in a large proportion due to disease progression rather than new inflammation. This causes a relative underestimation of patients who remain inflammatory stable. Taken together and based on this data, we assume that in the of all first-line DMT users 10% show no signs of inflammation for 5 years, and only a very small percentage of patients shows additional disease activity while staying on treatment (5%). The majority (95%) of patients remain inflammatory stable while continuing their medication.

This makes 700-750 patients eligible for inclusion.

4.2 Inclusion criteria

In order to be eligible to participate in this study, a subject must meet all of the following criteria:

1. A minimum age of 18 years
2. Ability to understand the purpose and risks of the study and provide signed and dated informed consent and authorization to use protected health information (PHI) in accordance with national and local privacy regulations.
3. Definite diagnosis of relapsing-onset MS according to the revised McDonald 2017 criteria
4. All relapsing-onset MS patients treated with one of the first-line treatments: any of the interferons, glatiramer acetate, dimethylfumarate, teriflunomide
5. Complete absence of inflammatory activity (no objectively defined and confirmed relapses, no significant number (2 or more) of new-T2 lesions suggestive of demyelination and no contrast-enhancing lesions) suggestive of demyelination for 5 consecutive years under first-line treatment. In case the last available MRI-scan was conducted 10 or more years ago, no more than 3 new T2-lesions suggestive of demyelination in the last 10 years are accepted.
6. Daily use of a smartphone with Android (5.0 or higher) or iOS (10 or higher) operating system.

4.3 Exclusion criteria

A potential subject who meets any of the following criteria will be excluded from participation in this study:

1. A switch between first-line disease modifying therapy over two years prior to inclusion, in case the switch has been due to in effectivity of the first DMT. In case the switch has been due to side-effects or by a personal preference of the patient (such as the wish to switch to oral therapies), this is not considered as an exclusion criterium.
2. Women who want to discontinue medication because of a pregnancy wish and women who are pregnant or expect to become pregnant during the study period
3. Patients that have previously used interferon-beta and have been tested positive for neutralizing antibodies (NAbs). This is determined by measuring MxA-bioactivity and is a test that is part of routine follow-up in patients that use interferon-beta. The reason for this is that development of NAbs has been shown to affect interferon-beta treatment efficacy.

4.4 Sample size calculation

The stability of patients in the continuation group is estimated to be at least 97,5%. Based on a non-inferiority margin of 7,5%, a preliminary power-calculation based on the non-inferiority principle was performed (PASS v12, one-sided Z-test (unpooled), significance level 0.05) and showed a necessary sample size of 54 per group to achieve 80% power. Taking 20% drop out into consideration, the total sample size needed for this study is 130.

The applications MS Sherpa and Neurokeys will be added to collect digital biomarkers regarding (return of) inflammatory disease activity. Based on previous experience, our estimate is that the current sample-size can provide some conclusive trends on the association between digital biomarkers and (return of) inflammatory disease activity.

5. TREATMENT OF SUBJECTS

5.1 Investigational product/treatment

The intervention consists of the discontinuation of the previously used DMT (either interferons, glatiramer acetate, dimethylfumarate or teriflunomide) Based on the pharmacological profile of the abovementioned drugs that are eligible for inclusion, there is no need for a tapering of dosage before complete discontinuation.

5.2 Use of co-intervention

During the intervention period patients are asked not to participate in any other scientific studies. Patients are allowed to use all types of co-medication, except for immunomodulating drugs such as prescribed for the treatment of multiple sclerosis and/or other auto-immune diseases.

5.3 Escape medication

When disease activity emerges, patients are treated according to the current standard of care (including intravenous methylprednisolone if deemed necessary) and disease modifying treatment will be reinitiated. Unscheduled visits including an MRI-scan are planned for each patient with any new neurological complaints, as is standard clinical procedure.

6. INVESTIGATIONAL PRODUCT

N/A

7. NON-INVESTIGATIONAL PRODUCT

MS Sherpa and Neurokeys are CE-certified medical devices. See for the relevant details appendices “D6 - Aanvullende productgegevens MS sherpa” and “D6 – Aanvullende productgegevens Neurokeys”.

8. METHODS

8.1 Study parameters/endpoints

8.1.1 Main study parameter/endpoint

The primary endpoint is number of patients with return of inflammatory disease activity after 2 years based on: a clinically confirmed relapse (defined according to the definition most often used in MS phase-III trials: the onset of new or recurrent symptoms that last > 24 hours, that are accompanied by new objective abnormalities on a neurological examination and that are not explained by non-MS processes such as fever, infection, severe stress or

drug toxicity (Gold et al NEJM 2012)) , or any emerging subclinical disease activity proven to be due to active disease/new inflammation (defined as 3 or more lesions on T2—weighted images or 2 or more gadolinium enhancing lesions on T1-weighted post-contrast MRI suggestive of demyelination) in the discontinuation group.

8.1.2 Secondary study parameters/endpoints (if applicable)

Secondary end-points are

- Changes in neurological functioning
  - EDSS change (Including individual functional systems)
  - MSFC changes
    - Timed 25-foot Walk (T25fW)
    - 9-Hole Peg Test (9HPT)
    - Symbol digits modalities test (SDMT)
- Individual MRI-parameters
  - T1 post-contrast lesion numbers and volumes
  - T2 lesion numbers and volumes
  - Whole brain volume
  - Normalized white matter volume
  - Grey matter volume
- Changes in quality of life measurements
  - Multiple Sclerosis Impact Scale (MSIS-29)
  - Short Form health survey (SF-36)
  - Multiple Sclerosis Self-Efficacy scale (MSSE)
  - Checklist Individual Strength (CIS20r)
  - Treatment Satisfaction Questionnaire for Medication (TSQM)
- Cost measurements
  - EuroQol 5 dimensions questionnaire (EQ-5D-5L)
  - Medical consumption questionnaire (iMCQ)
  - Productivity costs questionnaire (iPCQ)
- Changes in biomarker measurements
  - Neurofilament levels
- OCT and eye movement measurements
  - Peri-papillary retinal nerve fiber (RNFL) thickness
  - Macular ganglion cell-layer inner plexiform layer (GCL-IPL) thickness
  - Eye movement measurements

- Changes in digital biomarkers using the NeuroKeys (CE) and MS sherpa (CE) mobile applications that measure:
  - Walking test (2-minute walking test)
  - Cognition test (similar to SDMT)
  - MS sherpa questionnaires (including fatigue)
  - Keystroke data

8.1.3 Other study parameters (if applicable)

Vitamin D, smoking high body-mass index (BMI) are considered as potential confounders. Therefore, vitamin D will be determined and patients will be asked about smoking behaviour during every visit. To avoid any potential bias, we will also collect data (if present in the individual patients) on disease activity prior to the initiation of DMT, such date of diagnosis, time from first symptoms to diagnosis, EDSS scores/MS severity at the moment of DMT initiation.

8.2 Randomisation, blinding and treatment allocation

Since MS affects 2-2.5x as many women as men, we expect to include more women than men. A randomization algorithm will be included to match both groups for sex and age to exclude potential bias.

Outcome measurements will be performed by assessors who are blind to the intervention assignment. For reasons of consistency and to exclude possible bias, all scans will be centrally reviewed in VUmc by a radiologist blinded to allocation to the intervention group. In the current set-up of the trial patients and their treating neurologists are unblinded to the randomization group. The currently available first-line disease modifying treatments consist of the various (peg)interferons, glatiramer acetate, teriflunomide and dimethylfumarate. It comes in a total of more 10 different forms with different packaging, different modes of injection (some subcutaneously, some intramuscular and some oral) and different frequencies of intake (ranging from twice daily to once every two weeks). We have set up the trial so that it is rater-blinded; all MRI-scan and clinical evaluations (such as the neurological examination) will be performed blinded to “treatment” allocation. The primary and majority of secondary outcome measures are derived from these blinded assessments. This approach is frequently chosen in MS research (even in the phase-III trials) for 2 very important reasons. Due to the very distinct nature of side-effects (flushing, gastro-intestinal problems, flu-like symptoms), patients know when they receive placebo instead of the actual active compound. The invasive nature of receiving placebo-injections would greatly enhance the possibility of patients not participating. In addition, we believe it is too costly to create a placebo-control for each of the 10 different forms of medication.

8.3 Study procedures

No study procedures will take place before having obtained informed consent which will be gained following current METc/CCMO standards.

Follow-up frequency

Patients in the continuation group are expected to remain stable throughout the study period based on their proven stable status for 5 consecutive years prior to enrolment. The minimum evaluation that must take place should have a frequency according to the current standard of care (yearly evaluation of every patient treated with immunomodulating therapy; i.e. baseline (BL), 12 and 24 months). Timely recognition of recurrence of any (subclinical) disease activity in the discontinuation group is secured by more frequent clinical and radiological assessment during extra routine study visits at 3, 6 and 18 months. For optimal comparability and to overcome potential bias , both groups will be followed with a complete assessment including MRI at BL, 3, 6, 12, 18 and 24 moths. For the patients in both groups mobile applications MS sherpa and Neurokeys will be installed. With the MS sherpa application, patients will be asked to perform tests every two weeks during the 24 months of follow-up. The Neurokeys application will collect data on the background of the standard use of mobile phones during this time period.

Clinical evaluation

Duration: 1 hour

Clinical evaluation will consist of a careful medical history: current and past medication, adverse events, number of intravenous methylprednisolone treatments, number of relapses, date of last relapse and signs of symptom progression. A relapse is defined according to the definition most often used in MS phase-III trials: the onset of new or recurrent symptoms that last > 24 hours, that are accompanied by new objective abnormalities on a neurological examination and that are not explained by non-MS processes such as fever, infection, severe stress or drug toxicity[21]. Furthermore, the Expanded Disability Status Scale (EDSS) and Multiple Sclerosis Functional Composite (MSFC)-measurements will be performed, consisting of the Timed 25-foot Walk (T25fW), 9-Hole Peg Test (9HPT) and the symbol digits modalities test (SDMT). This will be done by a blinded investigator.

Radiological evaluation

Duration: 45 min

Radiological evaluation will consist of repeated brain MRI investigations that consist of conventional pre- and post-contrast (T2-weighted, T1-weighted pre and post contrast, FLAIR) images. All scan protocols are available in general and academic hospitals since they form the basis on which MS is diagnosed and treatment is monitored. Although it is expected that new inflammatory lesions can be captured by repeated T2-weighted/FLAIR MRI-scans, a gadolinium-scan is included to not miss any contrast enhancement in previously present lesions. A 3DT1 and 3DFLAIR image will also be made for atrophy measurements. For reasons of consistency and to exclude possible bias, all scans will be centrally reviewed in VUmc by a radiologist blinded to allocation to the intervention group.

Questionnaires

Duration: 1 hour

For the evaluation of disease burden and MS related symptoms, we will use the validated and in clinical studies often used Multiple Sclerosis Impact Scale (MSIS-29)[36] Short Form health survey (SF-36)[37] and CIS20r[38]. The perceived impact of treatment, convenience, satisfaction and side-effects will be monitored using the Treatment Satisfaction Questionnaire for Medication (TSQM)[39]. For the evaluation of health related quality of life (HRQoL) and the link between symptoms HRQoL and costs, we use the EDSS for the objective measurement of changes in neurological functioning (which has a clear relation to HRQoL assessed as utility and costs[40]) and the number of patients with return of inflammatory activity. We also use the EQ5D-5L) for cost-utility analysis[41]. Furthermore, we will ask patients to keep a diary describing changes in healthcare consumption (which will be defined per item in a questionnaire (such as hospitalisations, consultations with doctors, use of care at home, use of specialized transportation etc). Lastly patients will be asked for their employment situation and short-term and long-term sick absence because of MS (or MS related treatment) using the iMCQ and iPCQ questionnaires[42]. Questionnaires are filled in digitally either at home or during the hospital visit. Help of a carer or the study-nurse is allowed in cases of the inability using a computer.

Samples

Duration: 15 minutes

Blood collection will take place in both patient groups at every visit. It will consist blood collection for the purpose of biobanking and for diagnostics in the case of any –unforeseen-clinical events. Furthermore, we will retrospectively measure neurofilament light in serum using Simoa.

All participants will visit the hospital for 6 times over a time frame of 2 years. Each of these visits that take place will take approximately 2,5-3 hours (clinical assessment and MRI-scan). Also 1-hour questionnaires which can be completed at home digitally or at the hospital on paper will take place before/during each visit.

All samples will be collected, processed and stored according to the Standard Operating Procedures (SOP's) as described in the Parelsnoer Biobankprotocol version 8.0. (15) Samples will be stored at the Biobank VUmc. To ensure patient privacy all samples will be coded. A peripheral blood sample will be collected, in total 8 tubes of blood will be drawn (5x EDTA 4 ml for plasma, cells and DNA isolation, 2x serum 5 ml, 1x PAXgene tube 2 ml), adding up to a total volume of 32 ml. Blood will be centrifuged, divided in aliquots of 0.5 ml and then stored at -80 °C.

Optical coherence tomography (OCT)

Duration: 15 minutes

Frequency: yearly (at baseline or month 3, month 12 and month 24)

All OCT measurements will be performed by a certified person (not necessarily a physician) on a spectral-domain OCT machine (Spectralis by Heidelberg engineering). Scans to be performed in both eyes:

- Circular scan, centered on optic nerve head (ONH)
- Volume scan, centered on macula.
- Volume scan, centered on optic nerve head (ONH)

Eye movement examination

Frequency: yearly (at baseline or month 3, month 12 and month 24)

Duration: 15 minutes

Eye movement measurements will be performed using Eyelink 1000 Plus Eye Tracker (SR Research). Built-in algorithms provided by the eye tracker are used for calibration and validation procedures. The experiment consists of the following assessments:

1. Fixation
2. Pro-saccades
3. Anti-saccades

Mobile applications measurements

Duration: 5 minutes

Frequency: once every two weeks.

For eligible patients (based on regular smartphone use), mobile applications MS-Sherpa and Neurokeys will be installed on their smartphones and data will be collected via these applications.

MS sherpa mobile application

Via the MS sherpa application the patient will be asked to perform walking tests, cognition tests and the MS sherpa questionnaire (including fatigue).

- Cognitive task: similar to the SDMT, the participant is requested to assign numbers to corresponding symbols according to a specific displayed coding.
- Motor task: 2-minute walk test (2MWT): the participant is requested to walk (either unassisted or with a walking aid) for two minutes. The walking distance is measured through the location data. Patients who are unable to walk for two minutes will not be requested to do the 2MWT.
- MS sherpa questionnaire: patient reported outcomes on Likert scales (amongst others about fatigue and the impact of MS on daily activities).

These tests can be completed in approximately 5 minutes and patients will be asked to complete these tests once every two weeks.

NeuroKeys mobile application

NeuroKeys replaces the standard keyboard of the patient's smartphone. Data is collected from regular use of the keyboard, and no additional action from patients is needed. After 24 hours of inactivity of the keyboard of NeuroKeys, either intentionally or unintentionally, a push notification is automatically send utilizing Amazon Simple Notification Service (SNS). NeuroKeys will collect keystroke data general profile information (gender, year of birth, and MS type), and metadata (iOS/Android version and NeuroKeys version). The data will be collected from the phone continuously in a retrospective fashion, when the keyboard is activated the data from the previous keyboard typing session is sent.

- Keystroke data: the start of a message is defined as the keyboard flipping up and the end of a message is marked when the keyboard flips down. Specific keys will be logged and timestamped to be able to accurately calculate parameters expected to be associated to fatigue. The keys logged are: delete or backspace key, dot key, space bar, semicolon, colon, parentheses, capitalized characters, numbers and punctuation marks denouncing the ending of a sentence. All numbers and all punctuation marks will be logged as the same number or punctuation event (e.g. we do not make a distinction

between the number 3 and 8 or the comma and dollar sign). Parameters such as word count, amount of words comprised of six characters or more and latency between key presses will be calculated on the basis of the logged keys. In addition to ASCII keys, the unicodes of emojis will also be logged.

- Sensor data: Data from the location sensor, ambient light sensor, gyroscope, motion and accelerometers of the smartphone will be collected when the keyboard is in use. With the location sensor (longitude and latitude), the keystroke data can be combined to open source databases (e.g. weather data from the Royal Netherlands Meteorological Institute, KNMI) to examine the influence of external factors (Davis et al. 2010). Existing and future open source databases provided by public or governmental institutes can be accessed for this purpose. Ambient light sensor can detect environmental brightness which could impact typing behaviour. Kinematic sensors (gyroscope, motion and accelerometers) may be used to infer posture of the user (Lamonaca et al. 2015).

8.4 Withdrawal of individual subjects

Subjects can leave the study at any time for any reason if they wish to do so without any consequences. The investigator can decide to withdraw a subject from the study for urgent medical reasons.

8.4.1 Specific criteria for withdrawal (if applicable)

There are no specific criteria for withdrawal from the study.

8.5 Replacement of individual subjects after withdrawal

There will be no replacement of individual subjects after withdrawal.

8.6 Follow-up of subjects withdrawn from treatment

If a patient is withdrawn from the study, we will still perform follow-up measurements in case the patient is willing and able to cooperate.

8.7 Premature termination of the study

The procedure in case of premature termination of the study is described in section 10.4 and 12.5.

9. SAFETY REPORTING

9.1 Temporary halt for reasons of subject safety

In accordance to section 10, subsection 4, of the WMO, the sponsor will suspend the study if there is sufficient ground that continuation of the study will jeopardise subject health or safety. The sponsor will notify the accredited METC without undue delay of a temporary halt including the reason for such an action. The study will be suspended pending a further positive decision by the accredited METC. The investigator will take care that all subjects are kept informed.
More information about temporary halt for reasons of subject safety is provided in section 10.4 and 12.5.

9.2 AEs, SAEs and SUSARs

9.2.1 Adverse events (AEs)

Adverse events are defined as any undesirable experience occurring to a subject during the study, whether or not considered related to the experimental intervention. Adverse events that are reported spontaneously by the subject or observed by the investigator or his staff and that are relevant to the study will be recorded. Relevant adverse events are possible side-effects of the DMT used and the occurrence of relapses.

Adverse Device Effect (ADE)

An ADE is an adverse event related to the use of an investigational medical device. This includes any adverse event resulting from insufficiencies or inadequacies in the instructions of use, the deployment, the installation, the operation, or any malfunction of the investigational medical device. This also includes any event that is a result of a use error or intentional misuse.

9.2.2 Serious adverse events (SAEs)

A serious adverse event is any untoward medical occurrence or effect that

- results in death;
- is life threatening (at the time of the event);
- requires hospitalisation or prolongation of existing inpatients' hospitalisation;
- results in persistent or significant disability or incapacity;
- is a congenital anomaly or birth defect; or

- any other important medical event that did not result in any of the outcomes listed above due to medical or surgical intervention but could have been based upon appropriate judgement by the investigator.

A SADE is an ADE that has resulted in any of the consequences characteristic of a serious adverse event.

The investigator will report all SAEs and SADEs to the sponsor without undue delay after obtaining knowledge of the *events*. The sponsor will report the SAEs through the web portal *ToetsingOnline* to the accredited METC that approved the protocol, within 7 days of first knowledge for SAEs that result in death or are life threatening followed by a period of maximum of 8 days to complete the initial preliminary report. All other SAEs will be reported within a period of maximum 15 days after the sponsor has first knowledge of the serious adverse events.

9.2.3 Suspected unexpected serious adverse reactions (SUSARs)

This section is not applicable since this study does not investigational medicinal products.

9.3 Annual safety report

This section is not applicable since this study does not investigational medicinal products.

9.4 Follow-up of adverse events

All AEs will be followed until they have abated, or until a stable situation has been reached. Depending on the event, follow up may require additional tests or medical procedures as indicated, and/or referral to the general physician or a medical specialist.
SAEs need to be reported until the end of study, as defined in the protocol in section 9.2.2.

9.5 Data Safety Monitoring Board (DSMB)

For optimal safety we will appoint an independent data safety monitoring board (DSMB)which will monitor trial data on a regular basis. The aim of the committee is to safeguard the interests of trial participants and assess the safety of the discontinuation of therapy during the trial. The specific role of the DSMB consists of monitoring evidence for harm due to the intervention (discontinuing medication). The DSMB may advice to terminate the trial prematurely if disease activity exceeds above mentioned thresholds (see section 10.4 for interim analyses).

The DSMB consists of 4 members who do not have conflict of interest with the sponsor or the study. In addition to the permanent members of the DSMB two external members are added to the DSMB with expertise in the relevant field of research (yet to be determined).

The DSMB will conduct interim analyses in a predetermined manner and at predetermined times (see section 10.4), to see whether the relationship between clinical benefit and burden remains acceptable to the subject during the study. After each interim analysis, the DSMB reports to the study coordinator, with reports to the METc and the study sponsor, i.e. the Board of Directors (Raad van Bestuur). The DSMB can give advice to continue, change or stop (parts of) the study. The DSMB will also ensure the quality and safety of research in the participating centers.

10. STATISTICAL ANALYSIS

All data is quantitative and will be presented in tables and graphs. Baseline data will be collected to detect any potential differences between the two investigated groups for which we have not corrected with the randomization procedure (sex and age). Possible other confounders include smoking habits, vitamin D levels but also previous disease course in terms of differences in disease duration, number of relapses prior to stability, years of use of treatment.

The primary endpoint is the number of patients with return of inflammatory disease activity after 2 years based on: a clinically confirmed relapse or any emerging subclinical disease activity proven to be due to active disease/new inflammation (defined as 3 or more lesions on T2—weighted images or 2 or more gadolinium enhancing lesions on T1-weighted post-contrast MRI) in the discontinuation group. Secondary end-points are: 1. Changes in neurological functioning (EDSS/MSFC changes including individual functional systems and MSFC subtests), 2. Individual MRI parameters (T2 and T1 post-contrast lesion numbers), 3. changes in quality of life measurements (SF-36, CIS20r, TSQM, EQ5D-5L, iMCQ and iPCQ) and 4. changes in biomarker measurements (neurofilament levels).

In the case of confirmation of our hypothesis (discontinuing medication after >5 years without evidence of inflammatory disease activity does not result in return of inflammatory disease activity), we will extend the trial with a follow-up of two years where the group that continued their treatment gets offered the possibility to discontinue under similar safety measures (with similar follow-up frequencies and endpoints as the primary trial) and including the possibility to use MS sherpa and Neurokeys.

10.1 Primary study parameter(s)

For the primary endpoint, the return of inflammatory disease activity after 2 years, a2x2 contingency table will be used to estimate the risk difference for the return of inflammatory disease activity after 2 years (yes/no) between the two groups. The risk difference will be calculated for discontinuation relative to continuation. If the lower bound of the corresponding two-sided 90% confidence interval exceeds -7.5% we conclude non-inferiority of discontinuing medication For the primary endpoint both a per protocol analysis and a intention-to-treat analysis will be performed.

The primary endpoint is the number of patients with return of inflammatory disease activity after 2 years based on: a clinically confirmed relapse or any emerging subclinical disease activity proven to be due to active disease/new inflammation (defined as 3 or more lesions on T2—weighted images or 2 or more gadolinium enhancing lesions on T1-weighted post-contrast MRI suggestive of demyelination) in the discontinuation group.

A relapse is defined according to the definition most often used in MS phase-III trials: the onset of new or recurrent symptoms that last > 24 hours, that are accompanied by new objective abnormalities on a neurological examination and that are not explained by non-MS processes such as fever, infection, severe stress or drug toxicity[21]

Furthermore, a survival analysis (with an intention-to-treat approach) regarding the time to return of inflammatory activity will be included.

10.2 Secondary study parameter(s)

For all the secondary endpoints correlation and regression analysis (either linear of logistic, depending on the type of variable) will be performed correcting for possible confounders. On the app data, classification analyses and machine learning models will be used next to the more conventional analyses.

10.3 Other study parameters

N/A

10.4 Interim analysis

We will build in a safety-strategy (go-no-go strategy) to control for emerging disease activity (and patients safety). Interim analyses will be done after inclusion of the 40th, 70th and 100th patient. In both the continuation and discontinuation group, the proportion of patients that showed return of inflammatory disease activity – defined as an objectified MS relapse or 3 or more lesions on T2-weighted MRI-images or 2 or more gadolinium enhancing lesions on T1-weighted post-contrast MRI suggestive of demyelination – will be counted and compared between the treatment arms.

If there are more patients with return of inflammatory disease activity (according to the above mentioned definition) in the discontinuation group than in the continuation group, and the 95% confidence interval of the difference in the proportion of patients with return of disease

activity between both groups does not include 0, we will discuss premature ending of the study with the DSMB.

For optimal safety the DSMB will monitor the decision making on premature termination every 3 months. The DSMB may advice to terminate the trial prematurely if disease activity exceeds above mentioned thresholds. See section 9.5 for a more detailed description of the DSMB.

The procedure in case of premature termination of the study is described in section 12.5.

10.5 Cost-analysis

Alongside this trial, we will conduct a cost-effectiveness analysis and a cost-utility analysis over a 2-year follow-up. These will be performed in accordance with the recommendations of the Dutch guideline for health economic evaluations. For the cost-effectiveness analysis, the return of inflammatory disease activity (either relapse or new of enlarging lesions) will serve as the effectiveness measure. The cost-utility analysis will focus on quality of life measured with the EQ-5D-5L, which is often used in MS research. Quality-adjusted life-years (QALYs) will be calculated by multiplying the utility scores belonging to a health state by the amount of time spent in this health state using linear interpolation between time points.

In both analyses, costs will be measured from a societal perspective including health-care costs (such as the costs for a year of DMT, costs for the extra surveillance including additional medical tests as MRI, costs for visits to other medical doctors etc), direct nonmedical costs (costs that patients make for travelling to and from the hospital, private payments for extra health-care consumption etc) and indirect nonmedical costs (costs due to loss of production and short or long-term sick absence). The latter is important as previous research has shown that productivity losses are an important cost driver in severe MS. Health-care costs and direct nonmedical costs will be measured using the iMTA Medical Consumption Questionnaire (iMCQ) at 3 months, 6, months, 12 months, 18 months and 24 months. The iMCQ measures the health-care costs in the last three months. As these patients are assumed to be stable regarding their disease progression, these follow-up moments will provide an adequate estimation of their health-care use. Primary and secondary health-care costs will be valued using Dutch standard costs. If unavailable, tariffs or costs reported by the literature will be used. Medication will be valued using www.medicijnkosten.nl whereas informal care will be valued based on the costs of household care as reported by the Centraal Administratie Kantoor.

For indirect nonmedical costs, patients will be asked for their employment situation and both short-term and long-term sick absence because of MS (or MS related treatment) using the iMTA Productivity Cost Questionnaire (iPCQ) at 3 months, 6, months, 12 months, 18 months and 24 months. The iPCQ measures productivity loss in the last four weeks which will give an adequate overview of the productivity losses between each time point as these patients are assumed to be stable. Costs of absenteeism from paid work will be calculated according to both the human capital and friction cost approach. Costs of presenteeism will be calculated by asking participants how many working hours should have been replaced due to less productivity at work. Lost productivity due to either absenteeism or presenteeism will be valued using the mean age-, and sex specific income of the Dutch population. Costs of productivity losses due to absenteeism from unpaid work and informal care will be calculated using the standard wage of a professional housekeeper. All costs will be indexed to the year at which the trial ended. Missing data on costs and effects will be imputed using multiple imputations. In addition, costs and effects will both be discounted using a 3% discount rate.

For the cost-effectiveness analysis, we will calculate incremental cost-effectiveness ratios (ICERs) which is defined as the difference in mean costs divided by the difference in mean effects between the treatment continuation group and the treatment discontinuation group. For the cost-utility analysis, we will calculate incremental cost-utility ratios (ICURs) which is the difference in mean costs divided by the difference in mean QALYs. Bootstrapping with 5,000 replications will be used to estimate 95% credibility intervals around the ICERs and ICURs. The bootstrapped cost-effect pairs will be plotted on a cost-effectiveness plane and used to estimate cost-effectiveness acceptability curves (CEACs). CEACs show the probability that the intervention is cost-effective in comparison with the control treatment for a range of ceiling ratios. The ceiling ratio is defined as the willingness-to-pay, which is the amount of money society is willing to pay to gain one unit of effect.

In a sensitivity analysis, we will repeat all analyses using a healthcare payer perspective. In this analysis, only direct healthcare costs will be included. In addition, we will conduct subgroup analysis in which we will stratify individuals based on the presence of blood-based markers predictive for return of inflammatory disease activity.

Furthermore, we will conduct a budget impact analysis. A budget impact analysis (BIA) focuses on the expected changes in the expenditure of a health care system after the adoption of a new standard of care. In this BIA, we aim to estimate the future yearly budget impact of discontinuation of first line medication in patients with long-term stable relapsing-onset from a Dutch perspective. The BIA will be performed according to the BIA framework

of the International Society for Pharmacoeconomics and Outcomes Research (ISPOR). This framework consists of several standard aspects: target population, scenario distribution based on hospital types, resource utilisation, costs per unit, total costs, and sensitivity analyses.

11. ETHICAL CONSIDERATIONS

11.1 Regulation statement

The study will be conducted according to the principles of the Declaration of Helsinki (World Medical Association, 2013, Brazil) and in accordance with the Medical Research Involving Human Subjects Act (WMO) and the Good Clinical Practice guidelines.

11.2 Recruitment and consent

Patients will be informed about the study in different ways. Patients can be notified by their treating doctor during outpatient consultations at the VUmc or one of the other participating centres. In addition, a notification will be placed on the website of the MS Centre Amsterdam and the websites of the MS Vereniging Nederland (MSVN) and MS Web with information about the study. Patients can then discuss potential interest in the study with their treating neurologist. Inclusion is possible in one of the participating centers. Potential participants who express their wish to participate will receive additional information on paper about the purpose, intervention, duration and content of the study. They will also receive an informed consent form with careful and extensive information about the possible risks (according to local ethical review board requirements). In case there are any questions about the study, the study coordinator can be contacted. Moreover, an independent doctor (dr. Pijnenburg) can be contacted for additional questions.

Inclusion will take place after the written informed consent form has been returned to the trial coordinator. A copy of the informed consent form will be given to the participant and to the responsible physician in one of the participating centers in case the patient is recruited through one of the centers outside the VUmc. This consent can be revoked at any time without citing reasons. Patients will be given a minimum of 2 weeks to consider their decision. The maximum time will be as long as the inclusion of patients is ongoing and inclusion and exclusion criteria are met.

11.3 Objection by minors or incapacitated subjects (if applicable)

Not applicable, all participants will be adult and legally competent

11.4 Benefits and risks assessment, group relatedness

In the non-intervention group (continuation group) participants are expected to remain stable throughout the study period based on their proven stable status for 5 consecutive years prior to enrolment. Therefore, this group will face no potential risks and no direct benefit other than the usual medical care. The potential value of the outcome of the research will outweigh the

burden of participation for the study. Also, patients in the continuation group are offered the possibility to discontinue their medication under similar surveillance measures as the discontinuation group after 24-month follow-up period has ended.

The data on the discontinuation of therapy in long-term inflammatory stable MS patients is reassuring. One large retrospective cohort study has shown in 1200 patients that stable RRMS patients who stop treatment don’t have an increased relapse rate compared to patients who continue treatment[25]. There was a slightly higher disability progression of the discontinuation group, presumably based on the discontinuation of treatment in patients with progressive MS. Also, there seems to be no rebound inflammation after discontinuation of therapy in progressive MS, and a similar relapse rate as the years prior to discontinuation[26]. Lastly, one study has showed that patients 45 years or older, or patients with a DMT intake of 4 or more years without evidence of clinical or radiological disease activity showed a high likelihood of remaining relapse-free after discontinuation and absence of contrast enhancing lesions[27]

11.5 Compensation for injury

According to article 7 from the ‘Wet medisch-wetenschappelijk onderzoek met mensen’ (Staatsblad 1998, 161) an insurance is obtained by the VUmc. In case of injury or death of the participants because of the study, this insurance will compensate for injury or cover the cost caused by death or injury from the participants. The insurance is obtained by the Onderlinge Waarborgmaatschappij Centramed b.a., Postbus 7374, 2701 AJ Zoetermeer. The insurance company and the insurance accede to the decree mandatory insurance for ‘medisch-wetenschappelijk onderzoek met mensen (Staatsblad 2003, 266). Written information about the insurance will be provided for the participants.

11.6 Incentives (if applicable)

According to the current standard of care, the evaluation of every patient treated with immunomodulating therapy is at baseline, 12 and 24 months. Since visits are also scheduled at 3, 6 and 18 months, travel expenses and parking costs for these extra visits will be compensated. Participants will be compensated for their time and effort for study participation: they will receive a gift certificate of €25 upon study completion.

12. ADMINISTRATIVE ASPECTS, MONITORING AND PUBLICATION

12.1 Handling and storage of data and documents

Data will be handled confidentially . After collection, all data will be correctly labeled and securely stored. A subject identification code (SIC) will be used to link data to the subject. The SIC will consist of numbers and will not be based on the patient initials and birth-date. The key to the code will be kept separately from the coded data. The only people who have access to this code will be the principal investigator, the coordinating investigator and the corresponding investigator. No other people will have access to the link information. Great care will be taken to ensure that there is no link between SIC and information on which an individual can be identified. The handling of personal data in the database complies with the General Data Protection Regulation (De Algemene Verordening Gegevensbescherming) Potential data exchange with other countries will only take place after consent of the patient and handling of data will comply with the General Data Protection Regulation. The procedure for handling data includes data encryption, coding, secure storage, establishing limited access or varying levels of access to the biobank, removing identifying information from bio specimens and data. The infrastructure will consist of both hardware and software components, to prevent unauthorized access to databases.

An electronic case report form (CRF) will be developed to document the data collected in the study. This database will include demographic and patients characteristics (without birth date) and all outcomes of the study measures. Other investigators can request permission to get access to (a part) of this database for the purpose of research only, and only when the principal investigator gives permission. These investigators will not get access to the separate database which includes the participants' names, other identifiers and the SIC. All data will be stored on a computer protected with a password on the VUmc computer network. And access to the database will also be secured by a code. Only the trial coordinator and the principal investigator will know the code that gives access to the database with the link information.

After finalizing the study, the originals of all source documents will be stored for a period of 15 years in a locked room. Data that is collected and stored for the Biobank, will be stored for a period of 50 years. This period of storage has been determined to ensure that a follow-up study might be possible. In case of a follow-up study, a new protocol will be submitted to the METC and participants will have to sign a new informed consent form. Importantly, participants will only be approached for a follow-up study if they have indicated on the

informed consent form of the current study that they can be approached for a follow-up study.

The collection of data for medical research in the Netherlands is subject to the Personal Data Protection Act and in particular to the Medical Treatment Contracts Act.

Data storage Neurokeys

Data collected by NeuroKeys will be stored using Amazon AWS S3. There will be no identifiable information in this database, all keystroke and sensor data are logged with ID numbers only. A separate database, Amazon RDS (SQL server), will be used to store the verification code and personal information such as gender and year of birth, which can be used to send users push notifications. Both Amazon AWS S3 and Amazon RDS databases are located in Frankfurt, Germany, and are ISO 27001/27017/27018 compliant. All data is AES-256 encrypted in transit and at rest, a tokenization approach is used in which a sensitive data element is replaced by a non-sensitive equivalent and send by using a secure SSL link. Decryption keys are stored in a private encrypted environment. For iOS users, each time the NeuroKeys' keyboard is 'activated' (i.e. a new message is started), data of the previous keyboard session is uploaded to the database. Data of only one message is saved on the mobile phone, until a new message has started. For Android users data is uploaded to the database approximately every 4 hours.

Data storage MS sherpa

Data collected by MS sherpa will be stored using MongoDB Atlas, whose infrastructure runs on top of Amazon Web Services (ISO 27001/27017/27018 compliant) in Dublin, Ireland. General profile information (such as gender, age, length, weight, and e-mail address) is directly saved on AWS servers, in Dublin and AWS S3 in Frankfurt, Germany. Auth0 is used for user authentication and authorisation (ISO 27001/27018 compliant) and its EU office is based in London, UK. MongoDB Atlas and Auth0 achieved key compliance controls and objectives, as demonstrated by the completion of a Type 1 SOC 2 Report: Security. MongoDB, Inc. and Auth0 are also certified under the EU-US Privacy Shield. Data gathered via MS sherpa will be transferred to the database immediately after the data has been collected.

12.2 Monitoring and Quality Assurance

An independent monitor, the Clinical Research Bureau (CRB) of the VUmc, will monitor the proposed study according to Good Clinical Practice (GCP). For a selection of candidates

Informed consent is to be checked by the CRB. Besides that, source data verification is performed during the onsite monitoring. The conformity of the data used for analysis and the information in the patient files will be checked by the CRB. The intensity of the verification will be related to the risk arisen by the research. Inclusion and exclusion criteria will be checked as well as the main outcome measures. The CRB will check if the (S)AE's and SUSAR's are reported conforming the schedule as required by laws and regulations.

The quality assurance team under the leadership of a quality assurance manager (QAM) is responsible for providing an effective and efficient quality assurance system and counsel for the clinical research sites. In this quality assurance system, the QAM is responsible for ensuring appropriate global and affiliate-specific quality documents are developed and tracked, making sure they maintain an up-to-date overall inventory of all quality documents. Furthermore, the QAM and its team are responsible for ensuring all personnel involved in the clinical trial are properly qualified and trained for the job roles for which they are responsible. They are responsible for giving the personnel trainings and constantly assessing further opportunities for education and additional training. The quality assurance team is also responsible for checking compliance with the protocol, SOPs, GCP, and/or applicable regulatory requirement(s) and checking of the quality in all stages of data handling to ensure that all data are reliable and have been processed correctly. Moreover, the quality assurance team is responsible for auditing the various investigational sites.
If noncompliance with the protocol, SOPs, GCP, and/or applicable regulatory requirement(s) by and investigator/institution, or by member(s) or the sponsor's staff is detected during a quality assurance activity or audit, it is the responsibility of the QAM to report this to the trial's sponsor and principal investigator.

Quality assurance procedures:

Quality assurance is the systematic and independent examination of all clinical trial-related activities and documentations. The quality assurance procedure focuses on clinical investigator audits and audits of clinical trial documentation.

1. Document audits:

During the document audits, the quality assurance team oversees the documents that are generated before, during or at the end of the conduct of the clinical trial. For each document, a checklist is developed based on the relevant regulatory and organizational standards and SOPs. The aim of the audits is to ensure that the information and data in the documents are

complete, clear, reliable and consistent. Documents reviewed in the document audits include the clinical study protocol, the investigator's brochure and the clinical study report.

2. Clinical investigator audits:

The clinical investigator audits concern audits of the different research sites of the trial. They are performed to assess the site's regulatory compliance and clinical data quality (including adherence to the protocol). Paragraph 3 will address the procedures concerning these audits.

12.3 Amendments

Amendments are changes made to the research after a favourable opinion by the accredited METC has been given. All amendments will be notified to the METC that gave a favourable opinion. All amendments will be notified to the METC and to the competent authority.

12.4 Annual progress report

The investigator will submit a summary of the progress of the trial to the accredited METC once a year. Information will be provided on the date of inclusion of the first subject, numbers of subjects included and numbers of subjects that have completed the trial, serious adverse events/ serious adverse reactions, other problems, and amendments. The METC will also be informed on the start and end date of the trial.

12.5 Temporary halt and (prematurely) end of study report

The investigator/sponsor will notify the accredited METC of the end of the study within a period of 8 weeks. The end of the study is defined as the last patient's last visit.
The sponsor will notify the METC immediately of a temporary halt of the study, including the reason of such an action.
In case the study is ended prematurely, the sponsor will notify the accredited METC within 15 days, including the reasons for the premature termination.
Within one year after the end of the study, the investigator/sponsor will submit a final study report with the results of the study, including any publications/abstracts of the study, to the accredited METC.

12.6 Public disclosure and publication policy

This study will be registered in het Nederlands Trial Register (NTR) http://www.trialregister.nl and www.clinicaltrials.gov. Publication will be in accordance with the basic principles of

CCMO statement on publication policy. The results will be presented at (inter)national scientific meetings. The results will be published in a medical scientific journal. In none of the publication forms, participant identity will be disclosed.

13. STRUCTURED RISK ANALYSIS

13.1 Potential issues of concern

Paragraph 13.1 is not applicable.

13.2 Synthesis

The intervention in this study is the discontinuation of previously used DMT. No new products or agents are administered, nor will there be any dosage adjustments in the group that will continue their therapy. The specific DMT's that patients use prior to discontinuation, and that are used in the control group are all registered with the authorities and widely used for this specific indication. Although previous studies suggest that the risk of return of inflammatory activity after discontinuing DMT will be low in long-term stable RRMS patients (as also described in section 1), this is the main risk of the intervention. To monitor return of inflammatory activity, a safety strategy is built in, which is described in section 10.4. If safety criteria are exceeded, the study will be discontinued and DMT's will be reinitiated (in one patient group or in all patients, see section 10.4). A DSMB is appointed that will monitor the decision making on premature termination every 3 months (section 9.5).

14. REFERENCES

1 Goodin, D. S. The epidemiology of multiple sclerosis: insights to disease pathogenesis. *Handb Clin Neurol* **122**, 231-266, doi:10.1016/B978-0-444-52001-2.00010-8 (2014).

2 Weinshenker, B. G. Natural history of multiple sclerosis. *Ann Neurol* **36 Suppl**, S6-11 (1994).

3 Reich, D. S., Lucchinetti, C. F. & Calabresi, P. A. Multiple Sclerosis. *N Engl J Med* **378**, 169-180, doi:10.1056/NEJMra1401483 (2018).

4 Giovannoni, G. *et al.* Is it time to target no evident disease activity (NEDA) in multiple sclerosis? *Mult Scler Relat Disord* **4**, 329-333, doi:10.1016/j.msard.2015.04.006 (2015).

5 Arnold, D. L. *et al.* Peginterferon beta-1a improves MRI measures and increases the proportion of patients with no evidence of disease activity in relapsing-remitting multiple sclerosis: 2-year results from the ADVANCE randomized controlled trial. *BMC Neurol* **17**, 29, doi:10.1186/s12883-017-0799-0 (2017).

6 Havrdova, E. *et al.* Effect of delayed-release dimethyl fumarate on no evidence of disease activity in relapsing-remitting multiple sclerosis: integrated analysis of the phase III DEFINE and CONFIRM studies. *Eur J Neurol* **24**, 726-733, doi:10.1111/ene.13272 (2017).

7 Miller, A. E. *et al.* Oral teriflunomide for patients with a first clinical episode suggestive of multiple sclerosis (TOPIC): a randomised, double-blind, placebo-controlled, phase 3 trial. *Lancet Neurol* **13**, 977-986, doi:10.1016/S1474-4422(14)70191-7 (2014).

8 Freedman, M. S. *et al.* Moving toward earlier treatment of multiple sclerosis: Findings from a decade of clinical trials and implications for clinical practice. *Mult Scler Relat Disord* **3**, 147-155, doi:10.1016/j.msard.2013.07.001 (2014).

9 Ramsaransing, G. S. & De Keyser, J. Benign course in multiple sclerosis: a review. *Acta Neurol Scand* **113**, 359-369, doi:10.1111/j.1600-0404.2006.00637.x (2006).

10 Sartori, A., Abdoli, M. & Freedman, M. S. Can we predict benign multiple sclerosis? Results of a 20-year long-term follow-up study. *J Neurol* **264**, 1068-1075, doi:10.1007/s00415-017-8487-y (2017).

11 Benedikz, J. *et al.* The natural history of untreated multiple sclerosis in Iceland. A total population-based 50 year prospective study. *Clin Neurol Neurosurg* **104**, 208-210 (2002).

12 Perini, P., Tagliaferri, C., Belloni, M., Biasi, G. & Gallo, P. The HLA-DR13 haplotype is associated with "benign" multiple sclerosis in northeast Italy. *Neurology* **57**, 158-159 (2001).

13 Johnson, K. M., Zhou, H., Lin, F., Ko, J. J. & Herrera, V. Real-World Adherence and Persistence to Oral Disease-Modifying Therapies in Multiple Sclerosis Patients Over 1 Year. *J Manag Care Spec Pharm* **23**, 844-852, doi:10.18553/jmcp.2017.23.8.844 (2017).

14 Lattanzi, S. *et al.* Persistence to oral disease-modifying therapies in multiple sclerosis patients. *Journal of neurology* **264**, 2325-2329, doi:10.1007/s00415-017-8595-8 (2017).

15 Lanzillo, R. *et al.* A multicentRE observational analysiS of PErsistenCe to Treatment in the new multiple sclerosis era: the RESPECT study. *Journal of neurology* **265**, 1174-1183, doi:10.1007/s00415-018-8831-x (2018).

16 Vermersch, P. *et al.* Teriflunomide versus subcutaneous interferon beta-1a in patients with relapsing multiple sclerosis: a randomised, controlled phase 3 trial. *Multiple sclerosis (Houndmills, Basingstoke, England)* **20**, 705-716, doi:10.1177/1352458513507821 (2014).

17 Balak, D. M. *et al.* Prevalence of cutaneous adverse events associated with long-term disease-modifying therapy and their impact on health-related quality of life in patients with multiple sclerosis: a cross-sectional study. *BMC neurology* **13**, 146, doi:10.1186/1471-2377-13-146 (2013).

18 Rommer, P. S. & Zettl, U. K. Managing the side effects of multiple sclerosis therapy: pharmacotherapy options for patients. *Expert opinion on pharmacotherapy* **19**, 483-498, doi:10.1080/14656566.2018.1446944 (2018).

19 Lee Mortensen, G. & Rasmussen, P. V. The impact of quality of life on treatment preferences in multiple sclerosis patients. *Patient Prefer Adherence* **11**, 1789-1796, doi:10.2147/ppa.S142373 (2017).

20 La Mantia, L., Munari, L. M. & Lovati, R. Glatiramer acetate for multiple sclerosis. *Cochrane Database Syst Rev*, Cd004678, doi:10.1002/14651858.CD004678.pub2 (2010).

21 Gold, R. *et al.* Placebo-controlled phase 3 study of oral BG-12 for relapsing multiple sclerosis. *N Engl J Med* **367**, 1098-1107, doi:10.1056/NEJMoa1114287 (2012).

22 Fox, R. J. *et al.* Placebo-controlled phase 3 study of oral BG-12 or glatiramer in multiple sclerosis. *N Engl J Med* **367**, 1087-1097, doi:10.1056/NEJMoa1206328 (2012).
23 Sejbaek, T., Nybo, M., Petersen, T. & Illes, Z. Real-life persistence and tolerability with dimethyl fumarate. *Mult Scler Relat Disord* **24**, 42-46, doi:10.1016/j.msard.2018.05.007 (2018).
24 CIBG; ministerie van Volksgezondheid, W. e. S. *Prijsvorming*, <www.farmatec.nl> (z.d.).
25 Kister, I. *et al.* Discontinuing disease-modifying therapy in MS after a prolonged relapse-free period: a propensity score-matched study. *J Neurol Neurosurg Psychiatry* **87**, 1133-1137, doi:10.1136/jnnp-2016-313760 (2016).
26 Bonenfant, J. *et al.* Can we stop immunomodulatory treatments in secondary progressive multiple sclerosis? *Eur J Neurol* **24**, 237-244, doi:10.1111/ene.13181 (2017).
27 Bsteh, G. *et al.* Discontinuation of disease-modifying therapies in multiple sclerosis - Clinical outcome and prognostic factors. *Mult Scler* **23**, 1241-1248, doi:10.1177/1352458516675751 (2017).
28 O'Rourke, K. E. & Hutchinson, M. Stopping beta-interferon therapy in multiple sclerosis: an analysis of stopping patterns. *Mult Scler* **11**, 46-50, doi:10.1191/1352458505ms1131oa (2005).
29 Rio, J. *et al.* Factors related with treatment adherence to interferon beta and glatiramer acetate therapy in multiple sclerosis. *Mult Scler* **11**, 306-309, doi:10.1191/1352458505ms1173oa (2005).
30 CBO richtlijn (2012) Immunomodulerende en immunosuppressieve behandeling bij multipele sclerose. (2012).
31 Kennisagenda neurologie 2017: https://gallery.mailchimp.com/29087cdad5c58a12bd346e83f/files/3b5692f1-3840-48e2-99e7-405edb9a895f/Kennisagenda_Neurologie_eindversie_16_12_2017.pdf.
32 Kister, I. & Corboy, J. R. Reducing costs while enhancing quality of care in MS. *Neurology* **87**, 1617-1622, doi:10.1212/WNL.0000000000003113 (2016).
33 Uher, T. *et al.* Is no evidence of disease activity an achievable goal in MS patients on intramuscular interferon beta-1a treatment over long-term follow-up? *Mult Scler* **23**, 242-252, doi:10.1177/1352458516650525 (2017).
34 Rotstein, D. L., Healy, B. C., Malik, M. T., Chitnis, T. & Weiner, H. L. Evaluation of no evidence of disease activity in a 7-year longitudinal multiple sclerosis cohort. *JAMA Neurol* **72**, 152-158, doi:10.1001/jamaneurol.2014.3537 (2015).
35 De Stefano, N. *et al.* Long-term assessment of no evidence of disease activity in relapsing-remitting MS. *Neurology* **85**, 1722-1723, doi:10.1212/WNL.0000000000002105 (2015).
36 Gray, O., McDonnell, G. & Hawkins, S. Tried and tested: the psychometric properties of the multiple sclerosis impact scale (MSIS-29) in a population-based study. *Mult Scler* **15**, 75-80, doi:10.1177/1352458508096872 (2009).
37 Pfennings, L. E. *et al.* A health-related quality of life questionnaire for multiple sclerosis patients. *Acta Neurol Scand* **100**, 148-155 (1999).
38 Rietberg, M. B., Van Wegen, E. E. & Kwakkel, G. Measuring fatigue in patients with multiple sclerosis: reproducibility, responsiveness and concurrent validity of three Dutch self-report questionnaires. *Disabil Rehabil* **32**, 1870-1876, doi:10.3109/09638281003734458 (2010).
39 Eagle, T. *et al.* Treatment satisfaction across injectable, infusion, and oral disease-modifying therapies for multiple sclerosis. *Mult Scler Relat Disord* **18**, 196-201, doi:10.1016/j.msard.2017.10.002 (2017).
40 Kobelt, G., Berg, J., Lindgren, P. & Jonsson, B. Costs and quality of life in multiple sclerosis in Europe: method of assessment and analysis. *Eur J Health Econ* **7 Suppl 2**, S5-13, doi:10.1007/s10198-006-0365-y (2006).
41 Janssen, M. F. *et al.* Measurement properties of the EQ-5D-5L compared to the EQ-5D-3L across eight patient groups: a multi-country study. *Qual Life Res* **22**, 1717-1727, doi:10.1007/s11136-012-0322-4 (2013).
42 Bouwmans, C. *et al.* The iMTA Productivity Cost Questionnaire: A Standardized Instrument for Measuring and Valuing Health-Related Productivity Losses. *Value Health* **18**, 753-758, doi:10.1016/j.jval.2015.05.009 (2015).

RESEARCH PROTOCOL

The safety and cost-effectiveness of discontinuing disease-modifying therapies in stable relapsing-onset multiple sclerosis (DOT-MS): a randomized rater-blinded multicenter trial.

Version 6, February 2021

TABLE OF CONTENTS

1. INTRODUCTION AND RATIONALE 11
2. OBJECTIVES 15
3. STUDY DESIGN 17
4. STUDY POPULATION 18
4.1 Population (base) 18
4.2 Inclusion criteria 19
4.3 Exclusion criteria 19
4.4 Sample size calculation 20
5. TREATMENT OF SUBJECTS 21
5.1 Investigational product/treatment 21
5.2 Use of co-intervention 21
5.3 Escape medication 21
6. INVESTIGATIONAL PRODUCT 21
7. NON-INVESTIGATIONAL PRODUCT 21
8. METHODS 21
8.1 Study parameters/endpoints 21
8.1.1 Main study parameter/endpoint 21
8.1.2 Secondary study parameters/endpoints (if applicable) 22
8.1.3 Other study parameters (if applicable) 23
8.2 Randomisation, blinding and treatment allocation 23
8.3 Study procedures 24
8.4 Withdrawal of individual subjects 28
8.4.1 Specific criteria for withdrawal (if applicable) 28
8.5 Replacement of individual subjects after withdrawal 28
8.6 Follow-up of subjects withdrawn from treatment 28
8.7 Premature termination of the study 28
9. SAFETY REPORTING 29
9.1 Temporary halt for reasons of subject safety 29
9.2 AEs, SAEs and SUSARs 29
9.2.1 Adverse events (AEs) 29
9.2.2 Serious adverse events (SAEs) 29
9.2.3 Suspected unexpected serious adverse reactions (SUSARs) 30
9.3 Annual safety report 30
9.4 Follow-up of adverse events 30
9.5 Data Safety Monitoring Board (DSMB) 30
10. STATISTICAL ANALYSIS 32
10.1 Primary study parameter(s) 32
10.2 Secondary study parameter(s) 33
10.3 Other study parameters 33
10.4 Interim analysis 33
11. ETHICAL CONSIDERATIONS 37

11.1 Regulation statement 37
11.2 Recruitment and consent....37
11.3 Objection by minors or incapacitated subjects (if applicable)....37
11.4 Benefits and risks assessment, group relatedness....37
11.5 Compensation for injury 38
11.6 Incentives (if applicable)....38
12. ADMINISTRATIVE ASPECTS, MONITORING AND PUBLICATION 39
12.1 Handling and storage of data and documents 39
12.2 Monitoring and Quality Assurance....40
12.3 Amendments....42
12.4 Annual progress report....42
12.5 Temporary halt and (prematurely) end of study report....42
12.6 Public disclosure and publication policy....42
13. STRUCTURED RISK ANALYSIS....44
13.1 Potential issues of concern....44
13.2 Synthesis 44
14. REFERENCES 45

LIST OF ABBREVIATIONS AND RELEVANT DEFINITIONS

9HPT	9-Hole Peg Test
ABR	General Assessment and Registration form (ABR form), the application form that is required for submission to the accredited Ethics Committee; in Dutch: Algemeen Beoordelings- en Registratieformulier (ABR-formulier)
AE	Adverse Event
AR	Adverse Reaction
CA	Competent Authority
CCMO	Central Committee on Research Involving Human Subjects; in Dutch: Centrale Commissie Mensgebonden Onderzoek
CIS	Clinically Isolated Syndrome
CIS20r	Checklist Individual Strength
CV	Curriculum Vitae
DMT	Disease Modifying Therapy
DSMB	Data Safety Monitoring Board
EDSS	Expanded Disability Status Scale
EQ-5D-5L	EuroQol 5 Dimensions Questionnaire
EU	European Union
FLAIR	Fluid Attenuation Inversion Recovery
GCP	Good Clinical Practice
GDPR	General Data Protection Regulation; in Dutch: Algemene Verordening Gegevensbescherming (AVG)
IC	Informed Consent
iMCQ	Medical Consumption Questionnaire
iPCQ	Productivity Costs Questionnaire
METC	Medical research ethics committee (MREC); in Dutch: medisch-ethische toetsingscommissie (METC)
MRI	Magnetic Resonance Imaging
MS	Multiple Sclerosis
MSFC	Multiple Sclerosis Functional Composite
MSIS-29	Multiple Sclerosis Impact Scale
RRMS	Relapsing Remitting Multiple Sclerosis
(S)AE	(Serious) Adverse Event
SDMT	Symbol Digits Modalities Test

**Sponsor**	**The sponsor is the party that commissions the organisation or performance of the research, for example a pharmaceutical company, academic hospital, scientific organisation or investigator. A party that provides funding for a study but does not commission it is not regarded as the sponsor, but referred to as a subsidising party.**
**SF-36**	**Short Form Health Survey**
**SPMS**	**Secondary Progressive Multiple Sclerosis**
**SUSAR**	**Suspected Unexpected Serious Adverse Reaction**
**T25fW**	**Timed 25-foot Walk**
**TSQM**	**Treatment Satisfaction Questionnaire for Medication**
**UAVG**	**Dutch Act on Implementation of the General Data Protection Regulation; in Dutch: Uitvoeringswet AVG**
**WMO**	**Medical Research Involving Human Subjects Act; in Dutch: Wet Medisch-wetenschappelijk Onderzoek met Mensen**

SUMMARY

Rationale: The past few years, several new effective drugs have come onto the market for the treatment of relapsing remitting MS (RRMS), all of which have potentially serious side effects. The arrival of these drugs has led to a new aim for treating MS patients: achieving a status of complete clinical and radiological control of inflammatory events, also described as a status of no evident disease activity (NEDA-3). With these adjusted goals, medication is often started at an earlier stage and the disease is treated more aggressively. This leads to better control of the disease, but also to increased exposure to possible (serious) side effects. A considerable group of patients with a fully stable-disease under treatment merely have a benign or less inflammatory disease course rather than a necessity for treatment to prevent inflammation. This raises the question whether and when patients who have been stable under medication for years can safely discontinue the treatment. The hypothesis of this study is that discontinuing medication after >5 years without evidence of inflammatory disease activity does not result in return of inflammatory disease activity.

Objective: The aim of this study is to identify whether it is possible to safely discontinue treatment in MS patients who have shown no evidence of active inflammation in the years prior to inclusion clinically and/or radiologically. The secondary objectives address the questions whether the discontinuation of first-line treatment has an effect on disability progression and whether the discontinuation of first-line treatment improves the quality of life for the patient and if this can be measured in a daily setting using digital biomarkers.. Furthermore, blood collections will be included to assess whether it is possible to retrospectively predict possible return of inflammatory activity with biomarkers such as neurofilament light (NFL) or patient characteristics such as disease activity prior to disease modifying therapy (DMT). In case of emerging disease activity after the cessation of therapy we will assess if reinitiation will lead to NEDA again, and if there are long-term consequences. If possible, post-hoc analysis are performed for the different types of treatment compounds.

Study design: Multi-center randomized and controlled, rater-blinded trial in the Netherlands. 130 patients with relapse onset MS will be assigned to either discontinue the previously used DMT or to continue their DMT.

Study population: MS patients who are treated with one of the first-line treatments (any of the interferons, glatiramer acetate, dimethylfumarate, teriflunomide) and who had a complete absence of inflammatory activity (no relapses, no new-T2 lesions and no contrast-enhancing lesions) for 5 consecutive years under first-line treatment will be eligible for inclusion.

Intervention (if applicable): discontinuation of the previously used DMT.

Main study parameters/endpoints: The primary endpoint is the return of inflammatory disease activity after 2 years: either relapses, new or enlarging lesions on T2-weighted MRI

and gadolinium-enhancing lesions on post-contrast T1-weighted MRI. Secondary end-points are EDSS and MSFC progression (combined: EDSS plus), number of relapses, individual MRI-parameters (such as lesion numbers), quality of life measurements, optical coherence tomography (OCT) and eye movement measurements, and (digital) biomarker measurements.

Nature and extent of the burden and risks associated with participation, benefit and group relatedness: The burden of participation consists of assessments during visits at baseline, 3, 6, 12, 18 and 24 months. Every follow-up visit of both patient groups will consist of clinical and radiological measurements, quality of life questionnaires and blood collection. Additional data will be collected via mobile applications MS sherpa and Neurokeys, that will be installed on patients' smartphones. For this, patients will be asked to perform tasks on their phones, measuring walking ability, hand function, cognition and fatigue. This takes approximately five minutes every two weeks and is performed at home. The data on the discontinuation of therapy in long-term inflammatory stable MS patients is reassuring. One large retrospective cohort study has shown in 1200 patients that stable RRMS patients who stop treatment don't have an increased relapse rate compared to patients who continue treatment (Kister et al). There was a slightly higher disability progression of the discontinuation group, presumably based on the discontinuation of treatment in patients with progressive MS. Also, there seems to be no rebound inflammation after discontinuation of therapy in progressive MS, and a similar relapse rate as the years prior to discontinuation (Bonenfant et al). When disease activity emerges, patients are treated according to the current standard of care. The discontinuation of medication can be beneficial for the patient, as the side effects of medications can be significant for some patients.

1. INTRODUCTION AND RATIONALE

In recent decades, the therapeutic landscape of multiple sclerosis (MS) has changed dramatically. Coming from an era where virtually no therapies were available, there are currently more than 12 first- and second-line disease modifying treatment (DMT) options for the prevention of focal inflammatory demyelinating lesions in the brain and spinal cord. Clinically MS can be devastating; it affects roughly 1 in 1000 persons in the Netherlands usually diagnosed in the prime of their lives with a mean age at diagnosis around 30[1]. Two major disease phenotypes exist. The most important is relapsing-onset MS (80% MS patients), including patients with a clinically isolated syndrome (CIS), relapsing remitting MS (RRMS) and secondary progressive MS (SPMS). The other 20% suffers from a primary progressive MS (PPMS), a disease phenotype with more distinct neurodegeneration. Untreated, 50% of patients will need assistance walking small distances after 10-20 years after diagnosis[2].The main pathological hallmark in the first stages (CIS and RRMS) is recurrent focal inflammation of the brain and spinal cord leading to demyelination[3]. The first years after the diagnosis patients usually experience 2-3 relapses annually. The severity of neurological disability depends on the localisation of the inflammation. With increasing age, the amount of inflammation tends to diminish and an unknown neurodegenerative pathology drives the disease course. Clinically there is a progressive decline in neurological functioning; i.e. the “progressive” phase (or the secondary progressive (SPMS) disease course).

There has been great change in the timing of diagnosing MS and evaluating disease activity with the introduction of magnetic resonance imaging (MRI). In the early days the disease course was solely evaluated based on new relapses and/or progression of disability. Now, the arrival of MRI has led to a revised aim for treating MS patients: achieving a status of “no evident disease activity” (NEDA-3); complete clinical and radiological control of inflammatory events and no significant increase in diseability[4]. To date an estimated 10% of patients have a status of NEDA-3 under long-term first-line therapy, implying a full control of focal inflammation in these patients[5-7]. In recent years there has been a growing trend of starting treatment earlier and to treat more aggressively, partly based on the concept of NEDA. Treatment is almost always initiated directly after diagnosis but sometimes even before a definite diagnosis of RRMS is made[8]. There is however a substantial percentage of patients with a more benign disease course, described in a very broad range of 6-64% of MS patients[9-12]. At the moment of diagnosis, it is not known how the disease course will develop and based on the substantial group of “benign” MS cases, it is likely that a considerable group of patients that have long-term and fully stable-disease under DMT is unnecessarily

treated. In addition, there is the group of patients who experience disease progression despite their therapy. Also, these patients probably do not benefit from their therapy.

Exposure to treatment is not without risks and costs. Side-effects of MS medication are frequently present. Data on the proportion of patients discontinuing first-line DMT's demonstrates a discontinuation rate of 20-40% during an observation period over 1 year, with the occurrence of side effects and poor tolerability as the most common reason for drug withdrawal[13-15]. A large proportion of the patient population is confronted with side effects for both oral (teriflunomide, dimethylfumarate) and injectable (interferons, glatiramer acetate) DMT's. For example, the results of the post-approval clinical trials on the safety of teriflunomide compared to interferon beta-1a showed that 93-96% of the patients experienced side effects[16]. Each injectable first-line DMT can lead to mild cutaneous adverse events such as erythema and swelling, but also to more severe and persisting effects such as lipoatrophy, infections and even necrosis. Patients with a cutaneous reaction appeared to have a lower dermatology-specific health-related quality of life[17]. Serious events rates were also high ranging from 7% (interferon beta-1a) to 12% (teriflunomide)[16].

Each first-line DMT has a different mode of administration and specific side-effects. Interferons are administered biweekly s.c. (Plegridy), weekly i.m. (Avonex), second daily s.c. (Betaferon) or thrice weekly s.c. (Rebif). Flu-like symptoms are the most often reported side effects of interferon ß injections and are particularly challenging for MS patients[18,19]. But also allergic reactions, elevated liver enzymes leading to severe hepatic injury, thyroid autoimmunity, hypothyroidism and hematologic abnormalities might occur[18]. Glatiramer acetate (Copaxone) is injected subcutaneously daily or thrice weekly. Patterned reactions are most commonly reported in patients using glatiramer acetate, consisting of flushing, chest pain, palpitations, urticaria, anxiety and dyspnoea with a relative risk of 3.27. This patterned reaction unpredictably occur within minutes of injection and spontaneously resolve before 30 minutes[20]. Teriflunomide (Aubagio) is an oral drug and is administered daily. Hair thinning, increased blood pressure, fatigue, diarrhoea, sensory disturbances, elevated liver enzymes, and renal failure are adverse effects that have been reported in patients using teriflunomide[18]. The fourth agent in the first-line DMT group, dimethylfumarate (Tecfidera) is taken orally twice daily. Clinical phase 3 trials reported mild or moderate flushing and gastrointestinal (GI) adverse events, 36% and 42%, respectively as most common adverse effects[21-23]. Consequently, this led to treatment discontinuation in both trials. Serious side effects include urosepsis (interferons), hepatotoxicity (glatiramer acetate), but also progressive multifocal leukoencephalopathy (dimethyl fumarate), which are all potentially lethal.

Furthermore, there is a great burden of costs to society. The costs for a year of first-line immunomodulating drugs range from 12.000 – 15.000 euros annually[24]. The discontinuation of therapy in appropriate patients therefore also has a very significant effect with a potential cost-reduction of 2-2.5 million euros annually in The Netherlands. Costs due to side-effects (such as treatment and absence of work) are not even included here.

The data on the discontinuation of therapy in long-term inflammatory stable MS patients is reassuring. One large retrospective cohort study has shown in 1200 patients that stable RRMS patients who stop treatment don't have an increased relapse rate compared to patients who continue treatment[25]. There was a slightly higher disability progression of the discontinuation group, presumably based on the discontinuation of treatment in patients with progressive MS. Also, there seems to be no rebound inflammation after discontinuation of therapy in progressive MS, and a similar relapse rate as the years prior to discontinuation[26]. Lastly, one study has showed that patients 45 years or older, or patients with a DMT intake of 4 or more years without evidence of clinical or radiological disease activity showed a high likelihood of remaining relapse-free after discontinuation and absence of contrast enhancing lesions[27]. All studies where however hampered by either its retrospective nature, or incompleteness on for examples reasons for discontinuation or a sufficiently matched control group. Obviously, definite conclusions can only be drawn after a well-designed controlled trial. Currently, two trials with a similar question to ours are underway (DISCOMS; NCT03073603 and STOP-I-SEP; NCT03653273). These studies are different compared to our proposal in that the inclusion criteria only allow for older patients (older than 55 years and older than 50 years respectively) to be included.

The question of whether or not to discontinue therapy is increasingly present during our outpatient clinics [28,29]. As stated, an estimated 10% of patients have a status of NEDA-3 under long-term first-line DMT and could benefit from the results of this study. To give an indication of the number of patients concerned: in the Netherlands alone an estimated number of 7000-7500 patients with MS use first-line DMT, which means 700-750 patients would be eligible for this study and would benefit from results of this study (numbers are based on market shares and sales of each DMT, data not published). An enquiry amongst Dutch MS neurologists (data not published) shows that every neurologist struggles with the question on average 5-10 times per year. The Dutch 2012 CBO guideline " Multiple Sclerosis" recommends the discontinuation of DMT in patients who experience severe side-effects and who had secondary progressive MS for at least 3 years[30] but this is solely based on expert opinion. Also, internationally there are no guidelines guiding patients and neurologists in this question. Very recently in 2017, the committee of care evaluation of

neurology (‘Zorgevaluatie Neurologie’ (ZEN), part of the ‘Dutch society of Neurologists (Nederlandse Vereniging voor Neurologie; NVN) and supported by the Dutch Federation of medical specialists (FMS) and the Dutch Federation of Patients) has ranked this lack of evidence regarding the discontinuation of immunomodulating drugs in MS one of the most important science/knowledge gaps within daily neurological practice in the Netherlands. It listed this topic in its “ Kennisagenda 2018-2022” which prioritizes the 12 most urgent topics for scientific research in the field of neurology in the coming 4 years to improve the effectiveness and safety of our daily medical practice[31]. We have validated this support by means of a questionnaire amongst all 88 members of the MS Taskforce (Part of the NVN). All (100%) respondents (response-rate was 49%) indicated that they found the issue frequently present in their clinics and important tot research. Also, internationally there is strong call for more evidence-based guidelines and consensus regarding the discontinuation of treatment in long-term stable MS patients[32]. This underlines the national (and international) need for systematic research regarding this subject.

2. OBJECTIVES

With this study we will bring first evidence to this important issue by identifying whether it is possible to safely discontinue treatment in MS patients who have shown no evidence of active inflammation in the years prior to inclusion. Importantly, we include quality of life measurements to evaluate if this also translates to an increased perception of health. If present, it will optimize the treatment paradigm for individual MS patients through identifying unnecessary exposure to medication while proving the discontinuation of medication to be safe, and beneficial for the daily lives of individual patients in terms of an increased quality of life. In addition, it greatly benefits the general society since it also provides a more efficient use of funds with the significant cost-reduction it brings. The impact of the trial is immediate, within 5 years, and since all large MS centers will participate there is a direct line into the daily offices of treating neurologists, and into the development of adjusted guidelines regarding the treatment of MS. Most importantly, every possible result deriving from this trial will have a significant impact on (inter)national treatment strategies. Besides the primary question of the evaluation of safety we also incorporate validated measures of quality of life and disease perception to evaluate potential changes in quality of life. Furthermore, we include blood collections for the monitoring of neurofilament light (currently the only validated and clinically applied biomarker for the return of inflammatory activity) that could potentially serve as indicator for subclinical return of inflammatory activity. In addition, we will include optical coherence tomography (OCT) and eye movement measurements for patients participating in Amsterdam UMC. OCT measurements (especially retinal nerve fiber layer (RNFL) thickness) are known to be associated with disability in MS patients, and thus can be seen as a measure for disease progression.

Primary outcome measures used in this study are clinical and radiological outcome measures that are already part of routine clinical practice, which ensures that results of this trial can be directly and easily implemented in standard clinical care. In addition to these outcome measures, it would be of interest to determine optimal measurements for potential return of disease activity. A promising method in this regard is the use of mobile applications on patients' smartphones, because of their non-invasive nature (in contrast to for example MRI-scans) and the potential for more continuous measurements in a real-world setting (i.e. at home). Measurements with mobile applications MS sherpa and Neurokeys will be included for a subgroup of patients (depending on the availability of a smartphone), to investigate if return of inflammatory disease activity can be measured with these applications. These mobile applications will be installed on patients' smartphones.

See also methods section for a full description.

Research questions

Primary research question:

Can we safely discontinue first-line medication in MS patients with long-term absence of inflammation, without the return of *inflammatory disease activity* clinically and radiologically?

Other research questions:

- Does the discontinuation of first-line treatment have an effect on disability progression?
- Does the discontinuation of first-line treatment improve the quality of life for the patient?
- What is the effect of discontinuation of first-line treatment on individual MRI outcome measures such as lesion load and atrophy measurements?
- Is it possible to predict possible return of inflammatory activity with biomarkers such as neurofilament light (NFL) or patient characteristics such as disease activity prior to DMT?
- In case of emerging disease activity after cessation of DMT, will a restart of DMT result in NEDA again and if so, how long does it take?
- In case of emerging disease activity after treatment cessation, are there any differences between the different DMT compounds?
- What is the cost-effectiveness of discontinuation of DMT in The Netherlands?
- Is discontinuation of first-line DMT associated with OCT measurements and eye movement measurements?
- Is it possible to detect and predict (return of) inflammatory disease activity and disease progression with digital biomarkers using mobile applications such as MS sherpa and Neurokeys?

3. STUDY DESIGN

The study design is a multi-center rater-blinded randomized controlled trial in the Netherlands. The project will take place over period of 5 years (60 months) depending on the progress of inclusion. The lead and monitoring of the trial will be performed by the MS center Amsterdam (MSCA). Participating centers are listed in an appendix (I1).

Study-population: 130 relapse onset MS patients who are treated with one of the first-line treatments (any of the interferons, glatiramer acetate, dimethylfumarate, teriflunomide) *and* who had a complete absence of objective inflammatory activity (no objectified relapses, no significant number (2 or more) of new-T2 lesions and no contrast-enhancing lesions) for 5 consecutive years under first-line treatment will be eligible for inclusion. Patients may not have switched between first-line drugs over the two years prior to inclusion. If a switch has occurred this should not have been due to ineffectivity of the first DMT but due to side-effects or by a personal preference of the patient (such as the wish to switch to oral therapies). In the case of previous use of interferons patients must be negative for neutralizing antibodies (NAbs). Inclusion will take place after informed consent. This will be obtained after careful and extensive information about the possible risks according to local ethical review board requirements.

Intervention: The intervention is the discontinuation of the previously used DMT. Based on the pharmacological profile of the abovementioned drugs that are eligible for inclusion, there is no need for a tapering of dosage before complete discontinuation

Follow-up frequency: Patients in the continuation group are expected to remain stable throughout the study period based on their proven stable status for 5 consecutive years prior to enrolment. The minimum evaluation that must take place should have a frequency according to the current standard of care (yearly evaluation of every patient treated with immunomodulating therapy; i.e. baseline (BL), 12 and 24 months). Timely recognition of recurrence of any (subclinical) disease activity in the discontinuation group is secured by more frequent clinical and radiological assessment during extra routine study visits at 3, 6 and 18 months. For optimal comparability and to overcome potential bias, both groups will be followed with a complete assessment including MRI at BL, 3, 6, 12, 18 and 24 months.

After these two years of follow-up, the patients in the continuation group are offered to discontinue their DMT with a follow-up similar to the discontinuation group.

4. STUDY POPULATION

4.1 Population (base)

We will include 130 participants with the diagnosis relapse onset MS with a minimum age of 18 years. 65 patients will be assigned to the discontinuation group and 65 patients will be assigned to the continuation group. MS patients will be recruited through the VUmc outpatient clinic and the outpatient neurology departments of all participating centers. Because this research has a multicenter national design, MS patients of the majority of regions in the Netherlands will be included, including urban as well as rural areas. We believe that represents a valid cross-section of the average MS patient population. It is important to note however, as MS affects 2-2.5x as many women as men we expect to include more women than men. We will include a randomization algorithm to match both groups for sex to exclude potential bias.

We have estimated 700-750 patients to be potentially eligible for inclusion in The Netherlands. Currently an estimated number of 7000-7500 patients use first-line DMT for their MS. This number is estimated on data that was provided by all different pharmaceutical companies of each of the first-line therapies. Numbers are based on market shares and actual sales of each of the treatments (data not published).

The percentage of “stability” derives from the treatment effects described in the pivotal phase III trials of the currently available first-line treatments and 3 cohort studies with a longer follow-up that the average 2 years in the phase III trials. For our calculation of possible eligible patients, we have used the most “negative” scenario based on the results of these trials. The most recent phase III studies report on NEDA as outcome measure (Havrdova et al 2017, Arnold et al 2017), reflecting the new treatment concept of MS: complete stabilization of the disease process. In those cohorts NEDA ranges from 25-40% (Havrdova et al 2017, Arnold et al 2014 & 2017, Miller et al 2014, Nygaard et al 2015). It is important to note however, that the concept of NEDA also includes progressive neurological decline (such as is due to progressive disease and neurodegeneration). The percentage of patients that is free from inflammation is therefore somewhat underestimated in the numbers of these studies. Furthermore, most studies comprise on average a relatively short observation period of 2 years.

Three cohort studies exist with a longer follow-up. De Stefano et al, Uher et al and Rotstein et al published cohort studies that describe a longitudinal follow-up of NEDA status of ~ 200 patients for 7-10 years[33-35]. They showed that a fairly low percentage of patients remained

NEDA after 5 years (10-15%). In the cases of emerging inflammatory activity under treatment, most patients showed disease activity in the first 1-2 years after the start of treatment. Only a small minority of patients lose their NEDA status after 5 years (4%). It is very important to note that in the Rotstein-study the loss of NEDA status was in a large proportion due to disease progression rather than new inflammation. This causes a relative underestimation of patients who remain inflammatory stable. Taken together and based on this data, we assume that in the of all first-line DMT users 10% show no signs of inflammation for 5 years, and only a very small percentage of patients shows additional disease activity while staying on treatment (5%). The majority (95%) of patients remain inflammatory stable while continuing their medication.

This makes 700-750 patients eligible for inclusion.

4.2 Inclusion criteria

In order to be eligible to participate in this study, a subject must meet all of the following criteria:

1. A minimum age of 18 years
2. Ability to understand the purpose and risks of the study and provide signed and dated informed consent and authorization to use protected health information (PHI) in accordance with national and local privacy regulations.
3. Definite diagnosis of relapsing-onset MS according to the revised McDonald 2017 criteria
4. All relapsing-onset MS patients treated with one of the first-line treatments: any of the interferons, glatiramer acetate, dimethylfumarate, teriflunomide
5. Complete absence of inflammatory activity (no objectively defined and confirmed relapses, no significant number (2 or more) of new-T2 lesions suggestive of demyelination and no contrast-enhancing lesions) suggestive of demyelination for 5 consecutive years under first-line treatment. In case the last available MRI-scan was conducted 10 or more years ago, no more than 3 new T2-lesions suggestive of demyelination in the last 10 years are accepted.

4.3 Exclusion criteria

A potential subject who meets any of the following criteria will be excluded from participation in this study:

1. A switch between first-line disease modifying therapy over two years prior to inclusion, in case the switch has been due to in effectivity of the first DMT. In case the switch has been due to side-effects or by a personal preference of the patient (such as the wish to switch to oral therapies), this is not considered as an exclusion criterium.
2. Women who want to discontinue medication because of a pregnancy wish and women who are pregnant or expect to become pregnant during the study period
3. Patients that have previously used interferon-beta and have been tested positive for neutralizing antibodies (NAbs). This is determined by measuring MxA-bioactivity and is a test that is part of routine follow-up in patients that use interferon-beta. The reason for this is that development of NAbs has been shown to affect interferon-beta treatment efficacy.

4.4 Sample size calculation

The stability of patients in the continuation group is estimated to be at least 97,5%. Based on a non-inferiority margin of 7,5%, a preliminary power-calculation based on the non-inferiority principle was performed (PASS v12, one-sided Z-test (unpooled), significance level 0.05) and showed a necessary sample size of 54 per group to achieve 80% power. Taking 20% drop out into consideration, the total sample size needed for this study is 130.

The applications MS Sherpa and Neurokeys will be added to collect digital biomarkers regarding (return of) inflammatory disease activity. Based on previous experience, our estimate is that the current sample-size can provide some conclusive trends on the association between digital biomarkers and (return of) inflammatory disease activity.

5. TREATMENT OF SUBJECTS

5.1 Investigational product/treatment

The intervention consists of the discontinuation of the previously used DMT (either interferons, glatiramer acetate, dimethylfumarate or teriflunomide) Based on the pharmacological profile of the abovementioned drugs that are eligible for inclusion, there is no need for a tapering of dosage before complete discontinuation.

5.2 Use of co-intervention

During the intervention period patients are asked not to participate in any other scientific studies. Patients are allowed to use all types of co-medication, except for immunomodulating drugs such as prescribed for the treatment of multiple sclerosis and/or other auto-immune diseases.

5.3 Escape medication

When disease activity emerges, patients are treated according to the current standard of care (including intravenous methylprednisolone if deemed necessary) and disease modifying treatment will be reinitiated. Unscheduled visits including an MRI-scan are planned for each patient with any new neurological complaints, as is standard clinical procedure.

6. INVESTIGATIONAL PRODUCT

N/A

7. NON-INVESTIGATIONAL PRODUCT

MS Sherpa and Neurokeys are CE-certified medical devices. See for the relevant details appendices “D6 - Aanvullende productgegevens MS sherpa” and “D6 – Aanvullende productgegevens Neurokeys”.

8. METHODS

8.1 Study parameters/endpoints

8.1.1 Main study parameter/endpoint

The primary endpoint is number of patients with return of inflammatory disease activity after 2 years based on: a clinically confirmed relapse (defined according to the definition most often used in MS phase-III trials: the onset of new or recurrent symptoms that last > 24 hours, that are accompanied by new objective abnormalities on a neurological examination and that are not explained by non-MS processes such as fever, infection, severe stress or

drug toxicity (Gold et al NEJM 2012)) , or any emerging subclinical disease activity proven to be due to active disease/new inflammation (defined as 3 or more lesions on T2—weighted images or 2 or more gadolinium enhancing lesions on T1-weighted post-contrast MRI suggestive of demyelination) in the discontinuation group.

8.1.2 Secondary study parameters/endpoints (if applicable)

Secondary end-points are

- Changes in neurological functioning
  - EDSS change (Including individual functional systems)
  - MSFC changes
    - Timed 25-foot Walk (T25fW)
    - 9-Hole Peg Test (9HPT)
    - Symbol digits modalities test (SDMT)
- Individual MRI-parameters
  - T1 post-contrast lesion numbers and volumes
  - T2 lesion numbers and volumes
  - Whole brain volume
  - Normalized white matter volume
  - Grey matter volume
- Changes in quality of life measurements
  - Multiple Sclerosis Impact Scale (MSIS-29)
  - Short Form health survey (SF-36)
  - Multiple Sclerosis Self-Efficacy scale (MSSE)
  - Checklist Individual Strength (CIS20r)
  - Treatment Satisfaction Questionnaire for Medication (TSQM)
- Cost measurements
  - EuroQol 5 dimensions questionnaire (EQ-5D-5L)
  - Medical consumption questionnaire (iMCQ)
  - Productivity costs questionnaire (iPCQ)
- Changes in biomarker measurements
  - Neurofilament levels
- OCT and eye movement measurements
  - Peri-papillary retinal nerve fiber (RNFL) thickness
  - Macular ganglion cell-layer inner plexiform layer (GCL-IPL) thickness
  - Eye movement measurements

- Changes in digital biomarkers using the NeuroKeys (CE) and MS sherpa (CE) mobile applications that measure:
  - o Walking test (2-minute walking test)
  - o Cognition test (similar to SDMT)
  - o MS sherpa questionnaires (including fatigue)
  - o Keystroke data

8.1.3 Other study parameters (if applicable)

Vitamin D, smoking high body-mass index (BMI) are considered as potential confounders. Therefore, vitamin D will be determined and patients will be asked about smoking behaviour during every visit. To avoid any potential bias, we will also collect data (if present in the individual patients) on disease activity prior to the initiation of DMT, such date of diagnosis, time from first symptoms to diagnosis, EDSS scores/MS severity at the moment of DMT initiation.

8.2 Randomisation, blinding and treatment allocation

Since MS affects 2-2.5x as many women as men, we expect to include more women than men. A randomization algorithm will be included to match both groups for sex and age to exclude potential bias.

Outcome measurements will be performed by assessors who are blind to the intervention assignment. For reasons of consistency and to exclude possible bias, all scans will be centrally reviewed in VUmc by a radiologist blinded to allocation to the intervention group. In the current set-up of the trial patients and their treating neurologists are unblinded to the randomization group. The currently available first-line disease modifying treatments consist of the various (peg)interferons, glatiramer acetate, teriflunomide and dimethylfumarate. It comes in a total of more 10 different forms with different packaging, different modes of injection (some subcutaneously, some intramuscular and some oral) and different frequencies of intake (ranging from twice daily to once every two weeks). We have set up the trial so that it is rater-blinded; all MRI-scan and clinical evaluations (such as the neurological examination) will be performed blinded to “treatment” allocation. The primary and majority of secondary outcome measures are derived from these blinded assessments. This approach is frequently chosen in MS research (even in the phase-III trials) for 2 very important reasons. Due to the very distinct nature of side-effects (flushing, gastro-intestinal problems, flu-like symptoms), patients know when they receive placebo instead of the actual active compound. The invasive nature of receiving placebo-injections would greatly enhance the possibility of patients not participating. In addition, we believe it is too costly to create a placebo-control for each of the 10 different forms of medication.

8.3 Study procedures

No study procedures will take place before having obtained informed consent which will be gained following current METc/CCMO standards.

Follow-up frequency

Patients in the continuation group are expected to remain stable throughout the study period based on their proven stable status for 5 consecutive years prior to enrolment. The minimum evaluation that must take place should have a frequency according to the current standard of care (yearly evaluation of every patient treated with immunomodulating therapy; i.e. baseline (BL), 12 and 24 months). Timely recognition of recurrence of any (subclinical) disease activity in the discontinuation group is secured by more frequent clinical and radiological assessment during extra routine study visits at 3, 6 and 18 months. For optimal comparability and to overcome potential bias , both groups will be followed with a complete assessment including MRI at BL, 3, 6, 12, 18 and 24 moths. For the patients in both groups mobile applications MS sherpa and Neurokeys will be installed. With the MS sherpa application, patients will be asked to perform tests every two weeks during the 24 months of follow-up. The Neurokeys application will collect data on the background of the standard use of mobile phones during this time period.

Clinical evaluation

Duration: 1 hour

Clinical evaluation will consist of a careful medical history: current and past medication, adverse events, number of intravenous methylprednisolone treatments, number of relapses, date of last relapse and signs of symptom progression. A relapse is defined according to the definition most often used in MS phase-III trials: the onset of new or recurrent symptoms that last > 24 hours, that are accompanied by new objective abnormalities on a neurological examination and that are not explained by non-MS processes such as fever, infection, severe stress or drug toxicity[21]. Furthermore, the Expanded Disability Status Scale (EDSS) and Multiple Sclerosis Functional Composite (MSFC)-measurements will be performed, consisting of the Timed 25-foot Walk (T25fW), 9-Hole Peg Test (9HPT) and the symbol digits modalities test (SDMT). This will be done by a blinded investigator.

Radiological evaluation

Duration: 45 min

Radiological evaluation will consist of repeated brain MRI investigations that consist of conventional pre- and post-contrast (T2-weighted, T1-weighted pre and post contrast, FLAIR) images. All scan protocols are available in general and academic hospitals since they form the basis on which MS is diagnosed and treatment is monitored. Although it is expected that new inflammatory lesions can be captured by repeated T2-weighted/FLAIR MRI-scans, a gadolinium-scan is included to not miss any contrast enhancement in previously present lesions. A 3DT1 and 3DFLAIR image will also be made for atrophy measurements. For reasons of consistency and to exclude possible bias, all scans will be centrally reviewed in VUmc by a radiologist blinded to allocation to the intervention group.

Questionnaires

Duration: 1 hour

For the evaluation of disease burden and MS related symptoms, we will use the validated and in clinical studies often used Multiple Sclerosis Impact Scale (MSIS-29)[36] Short Form health survey (SF-36)[37] and CIS20r[38]. The perceived impact of treatment, convenience, satisfaction and side-effects will be monitored using the Treatment Satisfaction Questionnaire for Medication (TSQM)[39]. For the evaluation of health related quality of life (HRQoL) and the link between symptoms HRQoL and costs, we use the EDSS for the objective measurement of changes in neurological functioning (which has a clear relation to HRQoL assessed as utility and costs[40]) and the number of patients with return of inflammatory activity. We also use the EQ5D-5L) for cost-utility analysis[41]. Furthermore, we will ask patients to keep a diary describing changes in healthcare consumption (which will be defined per item in a questionnaire (such as hospitalisations, consultations with doctors, use of care at home, use of specialized transportation etc). Lastly patients will be asked for their employment situation and short-term and long-term sick absence because of MS (or MS related treatment) using the iMCQ and iPCQ questionnaires[42]. Questionnaires are filled in digitally either at home or during the hospital visit. Help of a carer or the study-nurse is allowed in cases of the inability using a computer.

Samples

Duration: 15 minutes

Blood collection will take place in both patient groups at every visit. It will consist blood collection for the purpose of biobanking and for diagnostics in the case of any –unforeseen- clinical events. Furthermore, we will retrospectively measure neurofilament light in serum using Simoa.

All participants will visit the hospital for 6 times over a time frame of 2 years. Each of these visits that take place will take approximately 2,5-3 hours (clinical assessment and MRI-scan). Also 1-hour questionnaires which can be completed at home digitally or at the hospital on paper will take place before/during each visit.

All samples will be collected, processed and stored according to the Standard Operating Procedures (SOP's) as described in the Parelsnoer Biobankprotocol version 8.0. (15) Samples will be stored at the Biobank VUmc. To ensure patient privacy all samples will be coded. A peripheral blood sample will be collected, in total 8 tubes of blood will be drawn (5x EDTA 4 ml for plasma, cells and DNA isolation, 2x serum 5 ml, 1x PAXgene tube 2 ml), adding up to a total volume of 32 ml. Blood will be centrifuged, divided in aliquots of 0.5 ml and then stored at -80 °C.

Optical coherence tomography (OCT)

Duration: 15 minutes

Frequency: yearly (at baseline or month 3, month 12 and month 24)

All OCT measurements will be performed by a certified person (not necessarily a physician) on a spectral-domain OCT machine (Spectralis by Heidelberg engineering). Scans to be performed in both eyes:

- Circular scan, centered on optic nerve head (ONH)
- Volume scan, centered on macula.
- Volume scan, centered on optic nerve head (ONH)

Eye movement examination

Frequency: yearly (at baseline or month 3, month 12 and month 24)

Duration: 15 minutes

Eye movement measurements will be performed using Eyelink 1000 Plus Eye Tracker (SR Research). Built-in algorithms provided by the eye tracker are used for calibration and validation procedures. The experiment consists of the following assessments:

1. Fixation
2. Pro-saccades
3. Anti-saccades

Mobile applications measurements

Duration: 5 minutes

Frequency: once every two weeks.

For eligible patients (based on regular smartphone use), mobile applications MS-Sherpa and Neurokeys will be installed on their smartphones and data will be collected via these applications.

MS sherpa mobile application

Via the MS sherpa application the patient will be asked to perform walking tests, cognition tests and the MS sherpa questionnaire (including fatigue).

- Cognitive task: similar to the SDMT, the participant is requested to assign numbers to corresponding symbols according to a specific displayed coding.
- Motor task: 2-minute walk test (2MWT): the participant is requested to walk (either unassisted or with a walking aid) for two minutes. The walking distance is measured through the location data. Patients who are unable to walk for two minutes will not be requested to do the 2MWT.
- MS sherpa questionnaire: patient reported outcomes on Likert scales (amongst others about fatigue and the impact of MS on daily activities).

These tests can be completed in approximately 5 minutes and patients will be asked to complete these tests once every two weeks.

NeuroKeys mobile application

NeuroKeys replaces the standard keyboard of the patient's smartphone. Data is collected from regular use of the keyboard, and no additional action from patients is needed. After 24 hours of inactivity of the keyboard of NeuroKeys, either intentionally or unintentionally, a push notification is automatically send utilizing Amazon Simple Notification Service (SNS). NeuroKeys will collect keystroke data general profile information (gender, year of birth, and MS type), and metadata (iOS/Android version and NeuroKeys version). The data will be collected from the phone continuously in a retrospective fashion, when the keyboard is activated the data from the previous keyboard typing session is sent.

- Keystroke data: the start of a message is defined as the keyboard flipping up and the end of a message is marked when the keyboard flips down. Specific keys will be logged and timestamped to be able to accurately calculate parameters expected to be associated to fatigue. The keys logged are: delete or backspace key, dot key, space bar, semicolon, colon, parentheses, capitalized characters, numbers and punctuation marks denouncing the ending of a sentence. All numbers and all punctuation marks will be logged as the same number or punctuation event (e.g. we do not make a distinction

between the number 3 and 8 or the comma and dollar sign). Parameters such as word count, amount of words comprised of six characters or more and latency between key presses will be calculated on the basis of the logged keys. In addition to ASCII keys, the unicodes of emojis will also be logged.

- Sensor data: Data from the location sensor, ambient light sensor, gyroscope, motion and accelerometers of the smartphone will be collected when the keyboard is in use. With the location sensor (longitude and latitude), the keystroke data can be combined to open source databases (e.g. weather data from the Royal Netherlands Meteorological Institute, KNMI) to examine the influence of external factors (Davis et al. 2010). Existing and future open source databases provided by public or governmental institutes can be accessed for this purpose. Ambient light sensor can detect environmental brightness which could impact typing behaviour. Kinematic sensors (gyroscope, motion and accelerometers) may be used to infer posture of the user (Lamonaca et al. 2015).

8.4 Withdrawal of individual subjects

Subjects can leave the study at any time for any reason if they wish to do so without any consequences. The investigator can decide to withdraw a subject from the study for urgent medical reasons.

8.4.1 Specific criteria for withdrawal (if applicable)

There are no specific criteria for withdrawal from the study.

8.5 Replacement of individual subjects after withdrawal

There will be no replacement of individual subjects after withdrawal.

8.6 Follow-up of subjects withdrawn from treatment

If a patient is withdrawn from the study, we will still perform follow-up measurements in case the patient is willing and able to cooperate.

8.7 Premature termination of the study

The procedure in case of premature termination of the study is described in section 10.4 and 12.5.

9. SAFETY REPORTING

9.1 Temporary halt for reasons of subject safety

In accordance to section 10, subsection 4, of the WMO, the sponsor will suspend the study if there is sufficient ground that continuation of the study will jeopardise subject health or safety. The sponsor will notify the accredited METC without undue delay of a temporary halt including the reason for such an action. The study will be suspended pending a further positive decision by the accredited METC. The investigator will take care that all subjects are kept informed.

More information about temporary halt for reasons of subject safety is provided in section 10.4 and 12.5.

9.2 AEs, SAEs and SUSARs

9.2.1 Adverse events (AEs)

Adverse events are defined as any undesirable experience occurring to a subject during the study, whether or not considered related to the experimental intervention. Adverse events that are reported spontaneously by the subject or observed by the investigator or his staff and that are relevant to the study will be recorded. Relevant adverse events are possible side-effects of the DMT used and the occurrence of relapses.

Adverse Device Effect (ADE)

An ADE is an adverse event related to the use of an investigational medical device. This includes any adverse event resulting from insufficiencies or inadequacies in the instructions of use, the deployment, the installation, the operation, or any malfunction of the investigational medical device. This also includes any event that is a result of a use error or intentional misuse.

9.2.2 Serious adverse events (SAEs)

A serious adverse event is any untoward medical occurrence or effect that

- results in death;
- is life threatening (at the time of the event);
- requires hospitalisation or prolongation of existing inpatients' hospitalisation;
- results in persistent or significant disability or incapacity;
- is a congenital anomaly or birth defect; or

- any other important medical event that did not result in any of the outcomes listed above due to medical or surgical intervention but could have been based upon appropriate judgement by the investigator.

A SADE is an ADE that has resulted in any of the consequences characteristic of a serious adverse event.

The investigator will report all SAEs and SADEs to the sponsor without undue delay after obtaining knowledge of the *events*. The sponsor will report the SAEs through the web portal *ToetsingOnline* to the accredited METC that approved the protocol, within 7 days of first knowledge for SAEs that result in death or are life threatening followed by a period of maximum of 8 days to complete the initial preliminary report. All other SAEs will be reported within a period of maximum 15 days after the sponsor has first knowledge of the serious adverse events.

9.2.3 Suspected unexpected serious adverse reactions (SUSARs)

This section is not applicable since this study does not investigational medicinal products.

9.3 Annual safety report

This section is not applicable since this study does not investigational medicinal products.

9.4 Follow-up of adverse events

All AEs will be followed until they have abated, or until a stable situation has been reached. Depending on the event, follow up may require additional tests or medical procedures as indicated, and/or referral to the general physician or a medical specialist.
SAEs need to be reported until the end of study, as defined in the protocol in section 9.2.2.

9.5 Data Safety Monitoring Board (DSMB)

For optimal safety we will appoint an independent data safety monitoring board (DSMB)which will monitor trial data on a regular basis. The aim of the committee is to safeguard the interests of trial participants and assess the safety of the discontinuation of therapy during the trial. The specific role of the DSMB consists of monitoring evidence for harm due to the intervention (discontinuing medication). The DSMB may advice to terminate the trial prematurely if disease activity exceeds above mentioned thresholds (see section 10.4 for interim analyses).

The DSMB consists of 4 members who do not have conflict of interest with the sponsor or the study. In addition to the permanent members of the DSMB two external members are added to the DSMB with expertise in the relevant field of research (yet to be determined).

The DSMB will conduct interim analyses in a predetermined manner and at predetermined times (see section 10.4), to see whether the relationship between clinical benefit and burden remains acceptable to the subject during the study. After each interim analysis, the DSMB reports to the study coordinator, with reports to the METc and the study sponsor, i.e. the Board of Directors (Raad van Bestuur). The DSMB can give advice to continue, change or stop (parts of) the study. The DSMB will also ensure the quality and safety of research in the participating centers.

10. STATISTICAL ANALYSIS

All data is quantitative and will be presented in tables and graphs. Baseline data will be collected to detect any potential differences between the two investigated groups for which we have not corrected with the randomization procedure (sex and age). Possible other confounders include smoking habits, vitamin D levels but also previous disease course in terms of differences in disease duration, number of relapses prior to stability, years of use of treatment.

The primary endpoint is the number of patients with return of inflammatory disease activity after 2 years based on: a clinically confirmed relapse or any emerging subclinical disease activity proven to be due to active disease/new inflammation (defined as 3 or more lesions on T2—weighted images or 2 or more gadolinium enhancing lesions on T1-weighted post-contrast MRI) in the discontinuation group. Secondary end-points are: 1. Changes in neurological functioning (EDSS/MSFC changes including individual functional systems and MSFC subtests), 2. Individual MRI parameters (T2 and T1 post-contrast lesion numbers), 3. changes in quality of life measurements (SF-36, CIS20r, TSQM, EQ5D-5L, iMCQ and iPCQ) and 4. changes in biomarker measurements (neurofilament levels).

In the case of confirmation of our hypothesis (discontinuing medication after >5 years without evidence of inflammatory disease activity does not result in return of inflammatory disease activity), we will extend the trial with a follow-up of two years where the group that continued their treatment gets offered the possibility to discontinue under similar safety measures (with similar follow-up frequencies and endpoints as the primary trial) and including the possibility to use MS sherpa and Neurokeys.

10.1 Primary study parameter(s)

For the primary endpoint, the return of inflammatory disease activity after 2 years, a2x2 contingency table will be used to estimate the risk difference for the return of inflammatory disease activity after 2 years (yes/no) between the two groups. The risk difference will be calculated for discontinuation relative to continuation. If the lower bound of the corresponding two-sided 90% confidence interval exceeds -7.5% we conclude non-inferiority of discontinuing medication For the primary endpoint both a per protocol analysis and a intention-to-treat analysis will be performed.

The primary endpoint is the number of patients with return of inflammatory disease activity after 2 years based on: a clinically confirmed relapse or any emerging subclinical disease activity proven to be due to active disease/new inflammation (defined as 3 or more lesions on T2—weighted images or 2 or more gadolinium enhancing lesions on T1-weighted post-contrast MRI suggestive of demyelination) in the discontinuation group.

A relapse is defined according to the definition most often used in MS phase-III trials: the onset of new or recurrent symptoms that last > 24 hours, that are accompanied by new objective abnormalities on a neurological examination and that are not explained by non-MS processes such as fever, infection, severe stress or drug toxicity[21]

Furthermore, a survival analysis (with an intention-to-treat approach) regarding the time to return of inflammatory activity will be included.

10.2 Secondary study parameter(s)

For all the secondary endpoints correlation and regression analysis (either linear of logistic, depending on the type of variable) will be performed correcting for possible confounders. On the app data, classification analyses and machine learning models will be used next to the more conventional analyses.

10.3 Other study parameters

N/A

10.4 Interim analysis

We will build in a safety-strategy (go-no-go strategy) to control for emerging disease activity (and patients safety). Interim analyses will be done after inclusion of the 40th, 70th and 100th patient. In both the continuation and discontinuation group, the proportion of patients that showed return of inflammatory disease activity – defined as an objectified MS relapse or 3 or more lesions on T2-weighted MRI-images or 2 or more gadolinium enhancing lesions on T1-weighted post-contrast MRI suggestive of demyelination – will be counted and compared between the treatment arms.

If there are more patients with return of inflammatory disease activity (according to the above mentioned definition) in the discontinuation group than in the continuation group, <u>and</u> the 95% confidence interval of the difference in the proportion of patients with return of disease

activity between both groups does not include 0, we will discuss premature ending of the study with the DSMB.

For optimal safety the DSMB will monitor the decision making on premature termination every 3 months. The DSMB may advice to terminate the trial prematurely if disease activity exceeds above mentioned thresholds. See section 9.5 for a more detailed description of the DSMB.

The procedure in case of premature termination of the study is described in section 12.5.

10.5 Cost-analysis

Alongside this trial, we will conduct a cost-effectiveness analysis and a cost-utility analysis over a 2-year follow-up. These will be performed in accordance with the recommendations of the Dutch guideline for health economic evaluations. For the cost-effectiveness analysis, the return of inflammatory disease activity (either relapse or new of enlarging lesions) will serve as the effectiveness measure. The cost-utility analysis will focus on quality of life measured with the EQ-5D-5L, which is often used in MS research. Quality-adjusted life-years (QALYs) will be calculated by multiplying the utility scores belonging to a health state by the amount of time spent in this health state using linear interpolation between time points.

In both analyses, costs will be measured from a societal perspective including health-care costs (such as the costs for a year of DMT, costs for the extra surveillance including additional medical tests as MRI, costs for visits to other medical doctors etc), direct nonmedical costs (costs that patients make for travelling to and from the hospital, private payments for extra health-care consumption etc) and indirect nonmedical costs (costs due to loss of production and short or long-term sick absence). The latter is important as previous research has shown that productivity losses are an important cost driver in severe MS. Health-care costs and direct nonmedical costs will be measured using the iMTA Medical Consumption Questionnaire (iMCQ) at 3 months, 6, months, 12 months, 18 months and 24 months. The iMCQ measures the health-care costs in the last three months. As these patients are assumed to be stable regarding their disease progression, these follow-up moments will provide an adequate estimation of their health-care use. Primary and secondary health-care costs will be valued using Dutch standard costs. If unavailable, tariffs or costs reported by the literature will be used. Medication will be valued using www.medicijnkosten.nl whereas informal care will be valued based on the costs of household care as reported by the Centraal Administratie Kantoor.

For indirect nonmedical costs, patients will be asked for their employment situation and both short-term and long-term sick absence because of MS (or MS related treatment) using the iMTA Productivity Cost Questionnaire (iPCQ) at 3 months, 6, months, 12 months, 18 months and 24 months. The iPCQ measures productivity loss in the last four weeks which will give an adequate overview of the productivity losses between each time point as these patients are assumed to be stable. Costs of absenteeism from paid work will be calculated according to both the human capital and friction cost approach. Costs of presenteeism will be calculated by asking participants how many working hours should have been replaced due to less productivity at work. Lost productivity due to either absenteeism or presenteeism will be valued using the mean age-, and sex specific income of the Dutch population. Costs of productivity losses due to absenteeism from unpaid work and informal care will be calculated using the standard wage of a professional housekeeper. All costs will be indexed to the year at which the trial ended. Missing data on costs and effects will be imputed using multiple imputations. In addition, costs and effects will both be discounted using a 3% discount rate.

For the cost-effectiveness analysis, we will calculate incremental cost-effectiveness ratios (ICERs) which is defined as the difference in mean costs divided by the difference in mean effects between the treatment continuation group and the treatment discontinuation group. For the cost-utility analysis, we will calculate incremental cost-utility ratios (ICURs) which is the difference in mean costs divided by the difference in mean QALYs. Bootstrapping with 5,000 replications will be used to estimate 95% credibility intervals around the ICERs and ICURs. The bootstrapped cost-effect pairs will be plotted on a cost-effectiveness plane and used to estimate cost-effectiveness acceptability curves (CEACs). CEACs show the probability that the intervention is cost-effective in comparison with the control treatment for a range of ceiling ratios. The ceiling ratio is defined as the willingness-to-pay, which is the amount of money society is willing to pay to gain one unit of effect.

In a sensitivity analysis, we will repeat all analyses using a healthcare payer perspective. In this analysis, only direct healthcare costs will be included. In addition, we will conduct subgroup analysis in which we will stratify individuals based on the presence of blood-based markers predictive for return of inflammatory disease activity.

Furthermore, we will conduct a budget impact analysis. A budget impact analysis (BIA) focuses on the expected changes in the expenditure of a health care system after the adoption of a new standard of care. In this BIA, we aim to estimate the future yearly budget impact of discontinuation of first line medication in patients with long-term stable relapsing-onset from a Dutch perspective. The BIA will be performed according to the BIA framework

of the International Society for Pharmacoeconomics and Outcomes Research (ISPOR). This framework consists of several standard aspects: target population, scenario distribution based on hospital types, resource utilisation, costs per unit, total costs, and sensitivity analyses.

11. ETHICAL CONSIDERATIONS

11.1 Regulation statement

The study will be conducted according to the principles of the Declaration of Helsinki (World Medical Association, 2013, Brazil) and in accordance with the Medical Research Involving Human Subjects Act (WMO) and the Good Clinical Practice guidelines.

11.2 Recruitment and consent

Patients will be informed about the study in different ways. Patients can be notified by their treating doctor during outpatient consultations at the VUmc or one of the other participating centres. In addition, a notification will be placed on the website of the MS Centre Amsterdam and the websites of the MS Vereniging Nederland (MSVN) and MS Web with information about the study. Patients can then discuss potential interest in the study with their treating neurologist. Inclusion is possible in one of the participating centers. Potential participants who express their wish to participate will receive additional information on paper about the purpose, intervention, duration and content of the study. They will also receive an informed consent form with careful and extensive information about the possible risks (according to local ethical review board requirements). In case there are any questions about the study, the study coordinator can be contacted. Moreover, an independent doctor (dr. Pijnenburg) can be contacted for additional questions.

Inclusion will take place after the written informed consent form has been returned to the trial coordinator. A copy of the informed consent form will be given to the participant and to the responsible physician in one of the participating centers in case the patient is recruited through one of the centers outside the VUmc. This consent can be revoked at any time without citing reasons. Patients will be given a minimum of 2 weeks to consider their decision. The maximum time will be as long as the inclusion of patients is ongoing and inclusion and exclusion criteria are met.

11.3 Objection by minors or incapacitated subjects (if applicable)

Not applicable, all participants will be adult and legally competent

11.4 Benefits and risks assessment, group relatedness

In the non-intervention group (continuation group) participants are expected to remain stable throughout the study period based on their proven stable status for 5 consecutive years prior to enrolment. Therefore, this group will face no potential risks and no direct benefit other than the usual medical care. The potential value of the outcome of the research will outweigh the

burden of participation for the study. Also, patients in the continuation group are offered the possibility to discontinue their medication under similar surveillance measures as the discontinuation group after 24-month follow-up period has ended.

The data on the discontinuation of therapy in long-term inflammatory stable MS patients is reassuring. One large retrospective cohort study has shown in 1200 patients that stable RRMS patients who stop treatment don't have an increased relapse rate compared to patients who continue treatment[25]. There was a slightly higher disability progression of the discontinuation group, presumably based on the discontinuation of treatment in patients with progressive MS. Also, there seems to be no rebound inflammation after discontinuation of therapy in progressive MS, and a similar relapse rate as the years prior to discontinuation[26]. Lastly, one study has showed that patients 45 years or older, or patients with a DMT intake of 4 or more years without evidence of clinical or radiological disease activity showed a high likelihood of remaining relapse-free after discontinuation and absence of contrast enhancing lesions[27]

11.5 Compensation for injury

According to article 7 from the 'Wet medisch-wetenschappelijk onderzoek met mensen' (Staatsblad 1998, 161) an insurance is obtained by the VUmc. In case of injury or death of the participants because of the study, this insurance will compensate for injury or cover the cost caused by death or injury from the participants. The insurance is obtained by the Onderlinge Waarborgmaatschappij Centramed b.a., Postbus 7374, 2701 AJ Zoetermeer. The insurance company and the insurance accede to the decree mandatory insurance for 'medisch-wetenschappelijk onderzoek met mensen (Staatsblad 2003, 266). Written information about the insurance will be provided for the participants.

11.6 Incentives (if applicable)

According to the current standard of care, the evaluation of every patient treated with immunomodulating therapy is at baseline, 12 and 24 months. Since visits are also scheduled at 3, 6 and 18 months, travel expenses and parking costs for these extra visits will be compensated. Participants will be compensated for their time and effort for study participation: they will receive a gift certificate of €25 upon study completion.

12. ADMINISTRATIVE ASPECTS, MONITORING AND PUBLICATION

12.1 Handling and storage of data and documents

Data will be handled confidentially . After collection, all data will be correctly labeled and securely stored. A subject identification code (SIC) will be used to link data to the subject. The SIC will consist of numbers and will not be based on the patient initials and birth-date. The key to the code will be kept separately from the coded data. The only people who have access to this code will be the principal investigator, the coordinating investigator and the corresponding investigator. No other people will have access to the link information. Great care will be taken to ensure that there is no link between SIC and information on which an individual can be identified. The handling of personal data in the database complies with the General Data Protection Regulation (De Algemene Verordening Gegevensbescherming) Potential data exchange with other countries will only take place after consent of the patient and handling of data will comply with the General Data Protection Regulation. The procedure for handling data includes data encryption, coding, secure storage, establishing limited access or varying levels of access to the biobank, removing identifying information from bio specimens and data. The infrastructure will consist of both hardware and software components, to prevent unauthorized access to databases.

An electronic case report form (CRF) will be developed to document the data collected in the study. This database will include demographic and patients characteristics (without birth date) and all outcomes of the study measures. Other investigators can request permission to get access to (a part) of this database for the purpose of research only, and only when the principal investigator gives permission. These investigators will not get access to the separate database which includes the participants' names, other identifiers and the SIC. All data will be stored on a computer protected with a password on the VUmc computer network. And access to the database will also be secured by a code. Only the trial coordinator and the principal investigator will know the code that gives access to the database with the link information.

After finalizing the study, the originals of all source documents will be stored for a period of 15 years in a locked room. Data that is collected and stored for the Biobank, will be stored for a period of 50 years. This period of storage has been determined to ensure that a follow-up study might be possible. In case of a follow-up study, a new protocol will be submitted to the METC and participants will have to sign a new informed consent form. Importantly, participants will only be approached for a follow-up study if they have indicated on the

informed consent form of the current study that they can be approached for a follow-up study.

The collection of data for medical research in the Netherlands is subject to the Personal Data Protection Act and in particular to the Medical Treatment Contracts Act.

Data storage Neurokeys

Data collected by NeuroKeys will be stored using Amazon AWS S3. There will be no identifiable information in this database, all keystroke and sensor data are logged with ID numbers only. A separate database, Amazon RDS (SQL server), will be used to store the verification code and personal information such as gender and year of birth, which can be used to send users push notifications. Both Amazon AWS S3 and Amazon RDS databases are located in Frankfurt, Germany, and are ISO 27001/27017/27018 compliant. All data is AES-256 encrypted in transit and at rest, a tokenization approach is used in which a sensitive data element is replaced by a non-sensitive equivalent and send by using a secure SSL link. Decryption keys are stored in a private encrypted environment. For iOS users, each time the NeuroKeys' keyboard is 'activated' (i.e. a new message is started), data of the previous keyboard session is uploaded to the database. Data of only one message is saved on the mobile phone, until a new message has started. For Android users data is uploaded to the database approximately every 4 hours.

Data storage MS sherpa

Data collected by MS sherpa will be stored using MongoDB Atlas, whose infrastructure runs on top of Amazon Web Services (ISO 27001/27017/27018 compliant) in Dublin, Ireland. General profile information (such as gender, age, length, weight, and e-mail address) is directly saved on AWS servers, in Dublin and AWS S3 in Frankfurt, Germany. Auth0 is used for user authentication and authorisation (ISO 27001/27018 compliant) and its EU office is based in London, UK. MongoDB Atlas and Auth0 achieved key compliance controls and objectives, as demonstrated by the completion of a Type 1 SOC 2 Report: Security. MongoDB, Inc. and Auth0 are also certified under the EU-US Privacy Shield. Data gathered via MS sherpa will be transferred to the database immediately after the data has been collected.

12.2 Monitoring and Quality Assurance

An independent monitor, the Clinical Research Bureau (CRB) of the VUmc, will monitor the proposed study according to Good Clinical Practice (GCP). For a selection of candidates

Informed consent is to be checked by the CRB. Besides that, source data verification is performed during the onsite monitoring. The conformity of the data used for analysis and the information in the patient files will be checked by the CRB. The intensity of the verification will be related to the risk arisen by the research. Inclusion and exclusion criteria will be checked as well as the main outcome measures. The CRB will check if the (S)AE's and SUSAR's are reported conforming the schedule as required by laws and regulations.

The quality assurance team under the leadership of a quality assurance manager (QAM) is responsible for providing an effective and efficient quality assurance system and counsel for the clinical research sites. In this quality assurance system, the QAM is responsible for ensuring appropriate global and affiliate-specific quality documents are developed and tracked, making sure they maintain an up-to-date overall inventory of all quality documents. Furthermore, the QAM and its team are responsible for ensuring all personnel involved in the clinical trial are properly qualified and trained for the job roles for which they are responsible. They are responsible for giving the personnel trainings and constantly assessing further opportunities for education and additional training. The quality assurance team is also responsible for checking compliance with the protocol, SOPs, GCP, and/or applicable regulatory requirement(s) and checking of the quality in all stages of data handling to ensure that all data are reliable and have been processed correctly. Moreover, the quality assurance team is responsible for auditing the various investigational sites.
If noncompliance with the protocol, SOPs, GCP, and/or applicable regulatory requirement(s) by and investigator/institution, or by member(s) or the sponsor's staff is detected during a quality assurance activity or audit, it is the responsibility of the QAM to report this to the trial's sponsor and principal investigator.

Quality assurance procedures:

Quality assurance is the systematic and independent examination of all clinical trial-related activities and documentations. The quality assurance procedure focuses on clinical investigator audits and audits of clinical trial documentation.

1. Document audits:

During the document audits, the quality assurance team oversees the documents that are generated before, during or at the end of the conduct of the clinical trial. For each document, a checklist is developed based on the relevant regulatory and organizational standards and SOPs. The aim of the audits is to ensure that the information and data in the documents are

complete, clear, reliable and consistent. Documents reviewed in the document audits include the clinical study protocol, the investigator's brochure and the clinical study report.

2. Clinical investigator audits:

The clinical investigator audits concern audits of the different research sites of the trial. They are performed to assess the site's regulatory compliance and clinical data quality (including adherence to the protocol). Paragraph 3 will address the procedures concerning these audits.

12.3 Amendments

Amendments are changes made to the research after a favourable opinion by the accredited METC has been given. All amendments will be notified to the METC that gave a favourable opinion. All amendments will be notified to the METC and to the competent authority.

12.4 Annual progress report

The investigator will submit a summary of the progress of the trial to the accredited METC once a year. Information will be provided on the date of inclusion of the first subject, numbers of subjects included and numbers of subjects that have completed the trial, serious adverse events/ serious adverse reactions, other problems, and amendments. The METC will also be informed on the start and end date of the trial.

12.5 Temporary halt and (prematurely) end of study report

The investigator/sponsor will notify the accredited METC of the end of the study within a period of 8 weeks. The end of the study is defined as the last patient's last visit.
The sponsor will notify the METC immediately of a temporary halt of the study, including the reason of such an action.
In case the study is ended prematurely, the sponsor will notify the accredited METC within 15 days, including the reasons for the premature termination.
Within one year after the end of the study, the investigator/sponsor will submit a final study report with the results of the study, including any publications/abstracts of the study, to the accredited METC.

12.6 Public disclosure and publication policy

This study will be registered in het Nederlands Trial Register (NTR) http://www.trialregister.nl and www.clinicaltrials.gov. Publication will be in accordance with the basic principles of

CCMO statement on publication policy. The results will be presented at (inter)national scientific meetings. The results will be published in a medical scientific journal. In none of the publication forms, participant identity will be disclosed.

13. STRUCTURED RISK ANALYSIS

13.1 Potential issues of concern

Paragraph 13.1 is not applicable.

13.2 Synthesis

The intervention in this study is the discontinuation of previously used DMT. No new products or agents are administered, nor will there be any dosage adjustments in the group that will continue their therapy. The specific DMT’s that patients use prior to discontinuation, and that are used in the control group are all registered with the authorities and widely used for this specific indication. Although previous studies suggest that the risk of return of inflammatory activity after discontinuing DMT will be low in long-term stable RRMS patients (as also described in section 1), this is the main risk of the intervention. To monitor return of inflammatory activity, a safety strategy is built in, which is described in section 10.4. If safety criteria are exceeded, the study will be discontinued and DMT’s will be reinitiated (in one patient group or in all patients, see section 10.4). A DSMB is appointed that will monitor the decision making on premature termination every 3 months (section 9.5).

14. REFERENCES

1 Goodin, D. S. The epidemiology of multiple sclerosis: insights to disease pathogenesis. *Handb Clin Neurol* **122**, 231-266, doi:10.1016/B978-0-444-52001-2.00010-8 (2014).

2 Weinshenker, B. G. Natural history of multiple sclerosis. *Ann Neurol* **36 Suppl**, S6-11 (1994).

3 Reich, D. S., Lucchinetti, C. F. & Calabresi, P. A. Multiple Sclerosis. *N Engl J Med* **378**, 169-180, doi:10.1056/NEJMra1401483 (2018).

4 Giovannoni, G. *et al.* Is it time to target no evident disease activity (NEDA) in multiple sclerosis? *Mult Scler Relat Disord* **4**, 329-333, doi:10.1016/j.msard.2015.04.006 (2015).

5 Arnold, D. L. *et al.* Peginterferon beta-1a improves MRI measures and increases the proportion of patients with no evidence of disease activity in relapsing-remitting multiple sclerosis: 2-year results from the ADVANCE randomized controlled trial. *BMC Neurol* **17**, 29, doi:10.1186/s12883-017-0799-0 (2017).

6 Havrdova, E. *et al.* Effect of delayed-release dimethyl fumarate on no evidence of disease activity in relapsing-remitting multiple sclerosis: integrated analysis of the phase III DEFINE and CONFIRM studies. *Eur J Neurol* **24**, 726-733, doi:10.1111/ene.13272 (2017).

7 Miller, A. E. *et al.* Oral teriflunomide for patients with a first clinical episode suggestive of multiple sclerosis (TOPIC): a randomised, double-blind, placebo-controlled, phase 3 trial. *Lancet Neurol* **13**, 977-986, doi:10.1016/S1474-4422(14)70191-7 (2014).

8 Freedman, M. S. *et al.* Moving toward earlier treatment of multiple sclerosis: Findings from a decade of clinical trials and implications for clinical practice. *Mult Scler Relat Disord* **3**, 147-155, doi:10.1016/j.msard.2013.07.001 (2014).

9 Ramsaransing, G. S. & De Keyser, J. Benign course in multiple sclerosis: a review. *Acta Neurol Scand* **113**, 359-369, doi:10.1111/j.1600-0404.2006.00637.x (2006).

10 Sartori, A., Abdoli, M. & Freedman, M. S. Can we predict benign multiple sclerosis? Results of a 20-year long-term follow-up study. *J Neurol* **264**, 1068-1075, doi:10.1007/s00415-017-8487-y (2017).

11 Benedikz, J. *et al.* The natural history of untreated multiple sclerosis in Iceland. A total population-based 50 year prospective study. *Clin Neurol Neurosurg* **104**, 208-210 (2002).

12 Perini, P., Tagliaferri, C., Belloni, M., Biasi, G. & Gallo, P. The HLA-DR13 haplotype is associated with "benign" multiple sclerosis in northeast Italy. *Neurology* **57**, 158-159 (2001).

13 Johnson, K. M., Zhou, H., Lin, F., Ko, J. J. & Herrera, V. Real-World Adherence and Persistence to Oral Disease-Modifying Therapies in Multiple Sclerosis Patients Over 1 Year. *J Manag Care Spec Pharm* **23**, 844-852, doi:10.18553/jmcp.2017.23.8.844 (2017).

14 Lattanzi, S. *et al.* Persistence to oral disease-modifying therapies in multiple sclerosis patients. *Journal of neurology* **264**, 2325-2329, doi:10.1007/s00415-017-8595-8 (2017).

15 Lanzillo, R. *et al.* A multicentRE observational analysiS of PErsistenCe to Treatment in the new multiple sclerosis era: the RESPECT study. *Journal of neurology* **265**, 1174-1183, doi:10.1007/s00415-018-8831-x (2018).

16 Vermersch, P. *et al.* Teriflunomide versus subcutaneous interferon beta-1a in patients with relapsing multiple sclerosis: a randomised, controlled phase 3 trial. *Multiple sclerosis (Houndmills, Basingstoke, England)* **20**, 705-716, doi:10.1177/1352458513507821 (2014).

17 Balak, D. M. *et al.* Prevalence of cutaneous adverse events associated with long-term disease-modifying therapy and their impact on health-related quality of life in patients with multiple sclerosis: a cross-sectional study. *BMC neurology* **13**, 146, doi:10.1186/1471-2377-13-146 (2013).

18 Rommer, P. S. & Zettl, U. K. Managing the side effects of multiple sclerosis therapy: pharmacotherapy options for patients. *Expert opinion on pharmacotherapy* **19**, 483-498, doi:10.1080/14656566.2018.1446944 (2018).

19 Lee Mortensen, G. & Rasmussen, P. V. The impact of quality of life on treatment preferences in multiple sclerosis patients. *Patient Prefer Adherence* **11**, 1789-1796, doi:10.2147/ppa.S142373 (2017).

20 La Mantia, L., Munari, L. M. & Lovati, R. Glatiramer acetate for multiple sclerosis. *Cochrane Database Syst Rev*, Cd004678, doi:10.1002/14651858.CD004678.pub2 (2010).

21 Gold, R. *et al.* Placebo-controlled phase 3 study of oral BG-12 for relapsing multiple sclerosis. *N Engl J Med* **367**, 1098-1107, doi:10.1056/NEJMoa1114287 (2012).

22 Fox, R. J. *et al.* Placebo-controlled phase 3 study of oral BG-12 or glatiramer in multiple sclerosis. *N Engl J Med* **367**, 1087-1097, doi:10.1056/NEJMoa1206328 (2012).
23 Sejbaek, T., Nybo, M., Petersen, T. & Illes, Z. Real-life persistence and tolerability with dimethyl fumarate. *Mult Scler Relat Disord* **24**, 42-46, doi:10.1016/j.msard.2018.05.007 (2018).
24 CIBG; ministerie van Volksgezondheid, W. e. S. *Prijsvorming*, <www.farmatec.nl> (z.d.).
25 Kister, I. *et al.* Discontinuing disease-modifying therapy in MS after a prolonged relapse-free period: a propensity score-matched study. *J Neurol Neurosurg Psychiatry* **87**, 1133-1137, doi:10.1136/jnnp-2016-313760 (2016).
26 Bonenfant, J. *et al.* Can we stop immunomodulatory treatments in secondary progressive multiple sclerosis? *Eur J Neurol* **24**, 237-244, doi:10.1111/ene.13181 (2017).
27 Bsteh, G. *et al.* Discontinuation of disease-modifying therapies in multiple sclerosis - Clinical outcome and prognostic factors. *Mult Scler* **23**, 1241-1248, doi:10.1177/1352458516675751 (2017).
28 O'Rourke, K. E. & Hutchinson, M. Stopping beta-interferon therapy in multiple sclerosis: an analysis of stopping patterns. *Mult Scler* **11**, 46-50, doi:10.1191/1352458505ms1131oa (2005).
29 Rio, J. *et al.* Factors related with treatment adherence to interferon beta and glatiramer acetate therapy in multiple sclerosis. *Mult Scler* **11**, 306-309, doi:10.1191/1352458505ms1173oa (2005).
30 CBO richtlijn (2012) Immunomodulerende en immunosuppressieve behandeling bij multipele sclerose. (2012).
31 Kennisagenda neurologie 2017: https://gallery.mailchimp.com/29087cdad5c58a12bd346e83f/files/3b5692f1-3840-48e2-99e7-405edb9a895f/Kennisagenda_Neurologie_eindversie_16_12_2017.pdf.
32 Kister, I. & Corboy, J. R. Reducing costs while enhancing quality of care in MS. *Neurology* **87**, 1617-1622, doi:10.1212/WNL.0000000000003113 (2016).
33 Uher, T. *et al.* Is no evidence of disease activity an achievable goal in MS patients on intramuscular interferon beta-1a treatment over long-term follow-up? *Mult Scler* **23**, 242-252, doi:10.1177/1352458516650525 (2017).
34 Rotstein, D. L., Healy, B. C., Malik, M. T., Chitnis, T. & Weiner, H. L. Evaluation of no evidence of disease activity in a 7-year longitudinal multiple sclerosis cohort. *JAMA Neurol* **72**, 152-158, doi:10.1001/jamaneurol.2014.3537 (2015).
35 De Stefano, N. *et al.* Long-term assessment of no evidence of disease activity in relapsing-remitting MS. *Neurology* **85**, 1722-1723, doi:10.1212/WNL.0000000000002105 (2015).
36 Gray, O., McDonnell, G. & Hawkins, S. Tried and tested: the psychometric properties of the multiple sclerosis impact scale (MSIS-29) in a population-based study. *Mult Scler* **15**, 75-80, doi:10.1177/1352458508096872 (2009).
37 Pfennings, L. E. *et al.* A health-related quality of life questionnaire for multiple sclerosis patients. *Acta Neurol Scand* **100**, 148-155 (1999).
38 Rietberg, M. B., Van Wegen, E. E. & Kwakkel, G. Measuring fatigue in patients with multiple sclerosis: reproducibility, responsiveness and concurrent validity of three Dutch self-report questionnaires. *Disabil Rehabil* **32**, 1870-1876, doi:10.3109/09638281003734458 (2010).
39 Eagle, T. *et al.* Treatment satisfaction across injectable, infusion, and oral disease-modifying therapies for multiple sclerosis. *Mult Scler Relat Disord* **18**, 196-201, doi:10.1016/j.msard.2017.10.002 (2017).
40 Kobelt, G., Berg, J., Lindgren, P. & Jonsson, B. Costs and quality of life in multiple sclerosis in Europe: method of assessment and analysis. *Eur J Health Econ* **7 Suppl 2**, S5-13, doi:10.1007/s10198-006-0365-y (2006).
41 Janssen, M. F. *et al.* Measurement properties of the EQ-5D-5L compared to the EQ-5D-3L across eight patient groups: a multi-country study. *Qual Life Res* **22**, 1717-1727, doi:10.1007/s11136-012-0322-4 (2013).
42 Bouwmans, C. *et al.* The iMTA Productivity Cost Questionnaire: A Standardized Instrument for Measuring and Valuing Health-Related Productivity Losses. *Value Health* **18**, 753-758, doi:10.1016/j.jval.2015.05.009 (2015).

RESEARCH PROTOCOL

The safety and cost-effectiveness of discontinuing disease-modifying therapies in stable relapsing-onset multiple sclerosis (DOT-MS): a randomized rater-blinded multicenter trial.

Version 7, February 2023

TABLE OF CONTENTS

1. INTRODUCTION AND RATIONALE 11
2. OBJECTIVES 15
3. STUDY DESIGN 17
4. STUDY POPULATION 18
4.1 Population (base) 18
4.2 Inclusion criteria 19
4.3 Exclusion criteria 19
4.4 Sample size calculation 20
5. TREATMENT OF SUBJECTS 21
5.1 Investigational product/treatment 21
5.2 Use of co-intervention 21
5.3 Escape medication 21
6. INVESTIGATIONAL PRODUCT 21
7. NON-INVESTIGATIONAL PRODUCT 21
8. METHODS 21
8.1 Study parameters/endpoints 21
8.1.1 Main study parameter/endpoint 21
8.1.2 Secondary study parameters/endpoints (if applicable) 22
8.1.3 Other study parameters (if applicable) 23
8.2 Randomisation, blinding and treatment allocation 23
8.3 Study procedures 24
8.4 Withdrawal of individual subjects 28
8.4.1 Specific criteria for withdrawal (if applicable) 28
8.5 Replacement of individual subjects after withdrawal 28
8.6 Follow-up of subjects withdrawn from treatment 28
8.7 Premature termination of the study 28
9. SAFETY REPORTING 29
9.1 Temporary halt for reasons of subject safety 29
9.2 AEs, SAEs and SUSARs 29
9.2.1 Adverse events (AEs) 29
9.2.2 Serious adverse events (SAEs) 29
9.2.3 Suspected unexpected serious adverse reactions (SUSARs) 30
9.3 Annual safety report 30
9.4 Follow-up of adverse events 30
9.5 Data Safety Monitoring Board (DSMB) 30
10. STATISTICAL ANALYSIS 32
10.1 Primary study parameter(s) 32
10.2 Secondary study parameter(s) 33
10.3 Other study parameters 33
10.4 Interim analysis 33
11. ETHICAL CONSIDERATIONS 37

11.1 Regulation statement 37
11.2 Recruitment and consent..37
11.3 Objection by minors or incapacitated subjects (if applicable)..................................37
11.4 Benefits and risks assessment, group relatedness 37
11.5 Compensation for injury 38
11.6 Incentives (if applicable)...38
12. ADMINISTRATIVE ASPECTS, MONITORING AND PUBLICATION 39
12.1 Handling and storage of data and documents 39
12.2 Monitoring and Quality Assurance...40
12.3 Amendments...42
12.4 Annual progress report..42
12.5 Temporary halt and (prematurely) end of study report..42
12.6 Public disclosure and publication policy...42
13. STRUCTURED RISK ANALYSIS...44
13.1 Potential issues of concern...44
13.2 Synthesis 44
14. REFERENCES 45

LIST OF ABBREVIATIONS AND RELEVANT DEFINITIONS

9HPT	9-Hole Peg Test
ABR	General Assessment and Registration form (ABR form), the application form that is required for submission to the accredited Ethics Committee; in Dutch: Algemeen Beoordelings- en Registratieformulier (ABR-formulier)
AE	Adverse Event
AR	Adverse Reaction
CA	Competent Authority
CCMO	Central Committee on Research Involving Human Subjects; in Dutch: Centrale Commissie Mensgebonden Onderzoek
CIS	Clinically Isolated Syndrome
CIS20r	Checklist Individual Strength
CV	Curriculum Vitae
DMT	Disease Modifying Therapy
DSMB	Data Safety Monitoring Board
EDSS	Expanded Disability Status Scale
EQ-5D-5L	EuroQol 5 Dimensions Questionnaire
EU	European Union
FLAIR	Fluid Attenuation Inversion Recovery
GCP	Good Clinical Practice
GDPR	General Data Protection Regulation; in Dutch: Algemene Verordening Gegevensbescherming (AVG)
IC	Informed Consent
iMCQ	Medical Consumption Questionnaire
iPCQ	Productivity Costs Questionnaire
METC	Medical research ethics committee (MREC); in Dutch: medisch-ethische toetsingscommissie (METC)
MRI	Magnetic Resonance Imaging
MS	Multiple Sclerosis
MSFC	Multiple Sclerosis Functional Composite
MSIS-29	Multiple Sclerosis Impact Scale
RRMS	Relapsing Remitting Multiple Sclerosis
(S)AE	(Serious) Adverse Event
SDMT	Symbol Digits Modalities Test

**Sponsor**	**The sponsor is the party that commissions the organisation or performance of the research, for example a pharmaceutical company, academic hospital, scientific organisation or investigator. A party that provides funding for a study but does not commission it is not regarded as the sponsor, but referred to as a subsidising party.**
**SF-36**	**Short Form Health Survey**
**SPMS**	**Secondary Progressive Multiple Sclerosis**
**SUSAR**	**Suspected Unexpected Serious Adverse Reaction**
**T25fW**	**Timed 25-foot Walk**
**TSQM**	**Treatment Satisfaction Questionnaire for Medication**
**UAVG**	**Dutch Act on Implementation of the General Data Protection Regulation; in Dutch: Uitvoeringswet AVG**
**WMO**	**Medical Research Involving Human Subjects Act; in Dutch: Wet Medisch-wetenschappelijk Onderzoek met Mensen**

SUMMARY

Rationale: The past few years, several new effective drugs have come onto the market for the treatment of relapsing remitting MS (RRMS), all of which have potentially serious side effects. The arrival of these drugs has led to a new aim for treating MS patients: achieving a status of complete clinical and radiological control of inflammatory events, also described as a status of no evident disease activity (NEDA-3). With these adjusted goals, medication is often started at an earlier stage and the disease is treated more aggressively. This leads to better control of the disease, but also to increased exposure to possible (serious) side effects. A considerable group of patients with a fully stable-disease under treatment merely have a benign or less inflammatory disease course rather than a necessity for treatment to prevent inflammation. This raises the question whether and when patients who have been stable under medication for years can safely discontinue the treatment. The hypothesis of this study is that discontinuing medication after >5 years without evidence of inflammatory disease activity does not result in return of inflammatory disease activity.

Objective: The aim of this study is to identify whether it is possible to safely discontinue treatment in MS patients who have shown no evidence of active inflammation in the years prior to inclusion clinically and/or radiologically. The secondary objectives address the questions whether the discontinuation of first-line treatment has an effect on disability progression and whether the discontinuation of first-line treatment improves the quality of life for the patient and if this can be measured in a daily setting using digital biomarkers.. Furthermore, blood collections will be included to assess whether it is possible to retrospectively predict possible return of inflammatory activity with biomarkers such as neurofilament light (NFL) or patient characteristics such as disease activity prior to disease modifying therapy (DMT). In case of emerging disease activity after the cessation of therapy we will assess if reinitiation will lead to NEDA again, and if there are long-term consequences. If possible, post-hoc analysis are performed for the different types of treatment compounds.

Study design: Multi-center randomized and controlled, rater-blinded trial in the Netherlands. 130 patients with relapse onset MS will be assigned to either discontinue the previously used DMT or to continue their DMT.

Study population: MS patients who are treated with one of the first-line treatments (any of the interferons, glatiramer acetate, dimethylfumarate, teriflunomide) and who had a complete absence of inflammatory activity (no relapses, no new-T2 lesions and no contrast-enhancing lesions) for 5 consecutive years under first-line treatment will be eligible for inclusion.

Intervention (if applicable): discontinuation of the previously used DMT.

Main study parameters/endpoints: The primary endpoint is the return of inflammatory disease activity after 2 years: either relapses, new or enlarging lesions on T2-weighted MRI

and gadolinium-enhancing lesions on post-contrast T1-weighted MRI. Secondary end-points are EDSS and MSFC progression (combined: EDSS plus), number of relapses, individual MRI-parameters (such as lesion numbers), quality of life measurements, optical coherence tomography (OCT) and eye movement measurements, and (digital) biomarker measurements.

Nature and extent of the burden and risks associated with participation, benefit and group relatedness: The burden of participation consists of assessments during visits at baseline, 3, 6, 12, 18 and 24 months. Every follow-up visit of both patient groups will consist of clinical and radiological measurements, quality of life questionnaires and blood collection. Additional data will be collected via mobile applications MS sherpa and Neurokeys, that will be installed on patients' smartphones. For this, patients will be asked to perform tasks on their phones, measuring walking ability, hand function, cognition and fatigue. This takes approximately five minutes every two weeks and is performed at home. The data on the discontinuation of therapy in long-term inflammatory stable MS patients is reassuring. One large retrospective cohort study has shown in 1200 patients that stable RRMS patients who stop treatment don't have an increased relapse rate compared to patients who continue treatment (Kister et al). There was a slightly higher disability progression of the discontinuation group, presumably based on the discontinuation of treatment in patients with progressive MS. Also, there seems to be no rebound inflammation after discontinuation of therapy in progressive MS, and a similar relapse rate as the years prior to discontinuation (Bonenfant et al). When disease activity emerges, patients are treated according to the current standard of care. The discontinuation of medication can be beneficial for the patient, as the side effects of medications can be significant for some patients.

1. INTRODUCTION AND RATIONALE

In recent decades, the therapeutic landscape of multiple sclerosis (MS) has changed dramatically. Coming from an era where virtually no therapies were available, there are currently more than 12 first- and second-line disease modifying treatment (DMT) options for the prevention of focal inflammatory demyelinating lesions in the brain and spinal cord. Clinically MS can be devastating; it affects roughly 1 in 1000 persons in the Netherlands usually diagnosed in the prime of their lives with a mean age at diagnosis around 30[1]. Two major disease phenotypes exist. The most important is relapsing-onset MS (80% MS patients), including patients with a clinically isolated syndrome (CIS), relapsing remitting MS (RRMS) and secondary progressive MS (SPMS). The other 20% suffers from a primary progressive MS (PPMS), a disease phenotype with more distinct neurodegeneration. Untreated, 50% of patients will need assistance walking small distances after 10-20 years after diagnosis[2].The main pathological hallmark in the first stages (CIS and RRMS) is recurrent focal inflammation of the brain and spinal cord leading to demyelination[3]. The first years after the diagnosis patients usually experience 2-3 relapses annually. The severity of neurological disability depends on the localisation of the inflammation. With increasing age, the amount of inflammation tends to diminish and an unknown neurodegenerative pathology drives the disease course. Clinically there is a progressive decline in neurological functioning; i.e. the “progressive” phase (or the secondary progressive (SPMS) disease course).

There has been great change in the timing of diagnosing MS and evaluating disease activity with the introduction of magnetic resonance imaging (MRI). In the early days the disease course was solely evaluated based on new relapses and/or progression of disability. Now, the arrival of MRI has led to a revised aim for treating MS patients: achieving a status of “no evident disease activity” (NEDA-3); complete clinical and radiological control of inflammatory events and no significant increase in diseability[4]. To date an estimated 10% of patients have a status of NEDA-3 under long-term first-line therapy, implying a full control of focal inflammation in these patients[5-7]. In recent years there has been a growing trend of starting treatment earlier and to treat more aggressively, partly based on the concept of NEDA. Treatment is almost always initiated directly after diagnosis but sometimes even before a definite diagnosis of RRMS is made[8]. There is however a substantial percentage of patients with a more benign disease course, described in a very broad range of 6-64% of MS patients[9-12]. At the moment of diagnosis, it is not known how the disease course will develop and based on the substantial group of “benign” MS cases, it is likely that a considerable group of patients that have long-term and fully stable-disease under DMT is unnecessarily

treated. In addition, there is the group of patients who experience disease progression despite their therapy. Also, these patients probably do not benefit from their therapy.

Exposure to treatment is not without risks and costs. Side-effects of MS medication are frequently present. Data on the proportion of patients discontinuing first-line DMT's demonstrates a discontinuation rate of 20-40% during an observation period over 1 year, with the occurrence of side effects and poor tolerability as the most common reason for drug withdrawal[13-15]. A large proportion of the patient population is confronted with side effects for both oral (teriflunomide, dimethylfumarate) and injectable (interferons, glatiramer acetate) DMT's. For example, the results of the post-approval clinical trials on the safety of teriflunomide compared to interferon beta-1a showed that 93-96% of the patients experienced side effects[16]. Each injectable first-line DMT can lead to mild cutaneous adverse events such as erythema and swelling, but also to more severe and persisting effects such as lipoatrophy, infections and even necrosis. Patients with a cutaneous reaction appeared to have a lower dermatology-specific health-related quality of life[17]. Serious events rates were also high ranging from 7% (interferon beta-1a) to 12% (teriflunomide)[16].

Each first-line DMT has a different mode of administration and specific side-effects. Interferons are administered biweekly s.c. (Plegridy), weekly i.m. (Avonex), second daily s.c. (Betaferon) or thrice weekly s.c. (Rebif). Flu-like symptoms are the most often reported side effects of interferon ß injections and are particularly challenging for MS patients[18,19]. But also allergic reactions, elevated liver enzymes leading to severe hepatic injury, thyroid autoimmunity, hypothyroidism and hematologic abnormalities might occur[18]. Glatiramer acetate (Copaxone) is injected subcutaneously daily or thrice weekly. Patterned reactions are most commonly reported in patients using glatiramer acetate, consisting of flushing, chest pain, palpitations, urticaria, anxiety and dyspnoea with a relative risk of 3.27. This patterned reaction unpredictably occur within minutes of injection and spontaneously resolve before 30 minutes[20]. Teriflunomide (Aubagio) is an oral drug and is administered daily. Hair thinning, increased blood pressure, fatigue, diarrhoea, sensory disturbances, elevated liver enzymes, and renal failure are adverse effects that have been reported in patients using teriflunomide[18]. The fourth agent in the first-line DMT group, dimethylfumarate (Tecfidera) is taken orally twice daily. Clinical phase 3 trials reported mild or moderate flushing and gastrointestinal (GI) adverse events, 36% and 42%, respectively as most common adverse effects[21-23]. Consequently, this led to treatment discontinuation in both trials. Serious side effects include urosepsis (interferons), hepatotoxicity (glatiramer acetate), but also progressive multifocal leukoencephalopathy (dimethyl fumarate), which are all potentially lethal.

Furthermore, there is a great burden of costs to society. The costs for a year of first-line immunomodulating drugs range from 12.000 – 15.000 euros annually[24]. The discontinuation of therapy in appropriate patients therefore also has a very significant effect with a potential cost-reduction of 2-2.5 million euros annually in The Netherlands. Costs due to side-effects (such as treatment and absence of work) are not even included here.

The data on the discontinuation of therapy in long-term inflammatory stable MS patients is reassuring. One large retrospective cohort study has shown in 1200 patients that stable RRMS patients who stop treatment don't have an increased relapse rate compared to patients who continue treatment[25]. There was a slightly higher disability progression of the discontinuation group, presumably based on the discontinuation of treatment in patients with progressive MS. Also, there seems to be no rebound inflammation after discontinuation of therapy in progressive MS, and a similar relapse rate as the years prior to discontinuation[26]. Lastly, one study has showed that patients 45 years or older, or patients with a DMT intake of 4 or more years without evidence of clinical or radiological disease activity showed a high likelihood of remaining relapse-free after discontinuation and absence of contrast enhancing lesions[27]. All studies where however hampered by either its retrospective nature, or incompleteness on for examples reasons for discontinuation or a sufficiently matched control group. Obviously, definite conclusions can only be drawn after a well-designed controlled trial. Currently, two trials with a similar question to ours are underway (DISCOMS; NCT03073603 and STOP-I-SEP; NCT03653273). These studies are different compared to our proposal in that the inclusion criteria only allow for older patients (older than 55 years and older than 50 years respectively) to be included.

The question of whether or not to discontinue therapy is increasingly present during our outpatient clinics [28,29]. As stated, an estimated 10% of patients have a status of NEDA-3 under long-term first-line DMT and could benefit from the results of this study. To give an indication of the number of patients concerned: in the Netherlands alone an estimated number of 7000-7500 patients with MS use first-line DMT, which means 700-750 patients would be eligible for this study and would benefit from results of this study (numbers are based on market shares and sales of each DMT, data not published). An enquiry amongst Dutch MS neurologists (data not published) shows that every neurologist struggles with the question on average 5-10 times per year. The Dutch 2012 CBO guideline " Multiple Sclerosis" recommends the discontinuation of DMT in patients who experience severe side-effects and who had secondary progressive MS for at least 3 years[30] but this is solely based on expert opinion. Also, internationally there are no guidelines guiding patients and neurologists in this question. Very recently in 2017, the committee of care evaluation of

neurology (‘Zorgevaluatie Neurologie’ (ZEN), part of the ‘Dutch society of Neurologists (Nederlandse Vereniging voor Neurologie; NVN) and supported by the Dutch Federation of medical specialists (FMS) and the Dutch Federation of Patients) has ranked this lack of evidence regarding the discontinuation of immunomodulating drugs in MS one of the most important science/knowledge gaps within daily neurological practice in the Netherlands. It listed this topic in its “ Kennisagenda 2018-2022” which prioritizes the 12 most urgent topics for scientific research in the field of neurology in the coming 4 years to improve the effectiveness and safety of our daily medical practice[31]. We have validated this support by means of a questionnaire amongst all 88 members of the MS Taskforce (Part of the NVN). All (100%) respondents (response-rate was 49%) indicated that they found the issue frequently present in their clinics and important tot research. Also, internationally there is strong call for more evidence-based guidelines and consensus regarding the discontinuation of treatment in long-term stable MS patients[32]. This underlines the national (and international) need for systematic research regarding this subject.

2. OBJECTIVES

With this study we will bring first evidence to this important issue by identifying whether it is possible to safely discontinue treatment in MS patients who have shown no evidence of active inflammation in the years prior to inclusion. Importantly, we include quality of life measurements to evaluate if this also translates to an increased perception of health. If present, it will optimize the treatment paradigm for individual MS patients through identifying unnecessary exposure to medication while proving the discontinuation of medication to be safe, and beneficial for the daily lives of individual patients in terms of an increased quality of life. In addition, it greatly benefits the general society since it also provides a more efficient use of funds with the significant cost-reduction it brings. The impact of the trial is immediate, within 5 years, and since all large MS centers will participate there is a direct line into the daily offices of treating neurologists, and into the development of adjusted guidelines regarding the treatment of MS. Most importantly, every possible result deriving from this trial will have a significant impact on (inter)national treatment strategies. Besides the primary question of the evaluation of safety we also incorporate validated measures of quality of life and disease perception to evaluate potential changes in quality of life. Furthermore, we include blood collections for the monitoring of neurofilament light (currently the only validated and clinically applied biomarker for the return of inflammatory activity) that could potentially serve as indicator for subclinical return of inflammatory activity. In addition, we will include optical coherence tomography (OCT) and eye movement measurements for patients participating in Amsterdam UMC. OCT measurements (especially retinal nerve fiber layer (RNFL) thickness) are known to be associated with disability in MS patients, and thus can be seen as a measure for disease progression.

Primary outcome measures used in this study are clinical and radiological outcome measures that are already part of routine clinical practice, which ensures that results of this trial can be directly and easily implemented in standard clinical care. In addition to these outcome measures, it would be of interest to determine optimal measurements for potential return of disease activity. A promising method in this regard is the use of mobile applications on patients' smartphones, because of their non-invasive nature (in contrast to for example MRI-scans) and the potential for more continuous measurements in a real-world setting (i.e. at home). Measurements with mobile applications MS sherpa and Neurokeys will be included for a subgroup of patients (depending on the availability of a smartphone), to investigate if return of inflammatory disease activity can be measured with these applications. These mobile applications will be installed on patients' smartphones.

See also methods section for a full description.

Research questions

Primary research question:

Can we safely discontinue first-line medication in MS patients with long-term absence of inflammation, without the return of *inflammatory disease activity* clinically and radiologically?

Other research questions:

- Does the discontinuation of first-line treatment have an effect on disability progression?
- Does the discontinuation of first-line treatment improve the quality of life for the patient?
- What is the effect of discontinuation of first-line treatment on individual MRI outcome measures such as lesion load and atrophy measurements?
- Is it possible to predict possible return of inflammatory activity with biomarkers such as neurofilament light (NFL) or patient characteristics such as disease activity prior to DMT?
- In case of emerging disease activity after cessation of DMT, will a restart of DMT result in NEDA again and if so, how long does it take?
- In case of emerging disease activity after treatment cessation, are there any differences between the different DMT compounds?
- What is the cost-effectiveness of discontinuation of DMT in The Netherlands?
- Is discontinuation of first-line DMT associated with OCT measurements and eye movement measurements?
- Is it possible to detect and predict (return of) inflammatory disease activity and disease progression with digital biomarkers using mobile applications such as MS sherpa and Neurokeys?

3. STUDY DESIGN

The study design is a multi-center rater-blinded randomized controlled trial in the Netherlands. The project will take place over period of 5 years (60 months) depending on the progress of inclusion. The lead and monitoring of the trial will be performed by the MS center Amsterdam (MSCA). Participating centers are listed in an appendix (I1).

Study-population: 130 relapse onset MS patients who are treated with one of the first-line treatments (any of the interferons, glatiramer acetate, dimethylfumarate, teriflunomide) *and* who had a complete absence of objective inflammatory activity (no objectified relapses, no significant number (2 or more) of new-T2 lesions and no contrast-enhancing lesions) for 5 consecutive years under first-line treatment will be eligible for inclusion. Patients may not have switched between first-line drugs over the two years prior to inclusion. If a switch has occurred this should not have been due to ineffectivity of the first DMT but due to side-effects or by a personal preference of the patient (such as the wish to switch to oral therapies). In the case of previous use of interferons patients must be negative for neutralizing antibodies (NAbs). Inclusion will take place after informed consent. This will be obtained after careful and extensive information about the possible risks according to local ethical review board requirements.

Intervention: The intervention is the discontinuation of the previously used DMT. Based on the pharmacological profile of the abovementioned drugs that are eligible for inclusion, there is no need for a tapering of dosage before complete discontinuation

Follow-up frequency: Patients in the continuation group are expected to remain stable throughout the study period based on their proven stable status for 5 consecutive years prior to enrolment. The minimum evaluation that must take place should have a frequency according to the current standard of care (yearly evaluation of every patient treated with immunomodulating therapy; i.e. baseline (BL), 12 and 24 months). Timely recognition of recurrence of any (subclinical) disease activity in the discontinuation group is secured by more frequent clinical and radiological assessment during extra routine study visits at 3, 6 and 18 months. For optimal comparability and to overcome potential bias, both groups will be followed with a complete assessment including MRI at BL, 3, 6, 12, 18 and 24 months.

After these two years of follow-up, the patients in the continuation group are offered to discontinue their DMT with a follow-up similar to the discontinuation group.

4. STUDY POPULATION

4.1 Population (base)

We will include 130 participants with the diagnosis relapse onset MS with a minimum age of 18 years. 65 patients will be assigned to the discontinuation group and 65 patients will be assigned to the continuation group. MS patients will be recruited through the VUmc outpatient clinic and the outpatient neurology departments of all participating centers. Because this research has a multicenter national design, MS patients of the majority of regions in the Netherlands will be included, including urban as well as rural areas. We believe that represents a valid cross-section of the average MS patient population. It is important to note however, as MS affects 2-2.5x as many women as men we expect to include more women than men. We will include a randomization algorithm to match both groups for sex to exclude potential bias.

We have estimated 700-750 patients to be potentially eligible for inclusion in The Netherlands. Currently an estimated number of 7000-7500 patients use first-line DMT for their MS. This number is estimated on data that was provided by all different pharmaceutical companies of each of the first-line therapies. Numbers are based on market shares and actual sales of each of the treatments (data not published).

The percentage of “stability” derives from the treatment effects described in the pivotal phase III trials of the currently available first-line treatments and 3 cohort studies with a longer follow-up that the average 2 years in the phase III trials. For our calculation of possible eligible patients, we have used the most “negative” scenario based on the results of these trials. The most recent phase III studies report on NEDA as outcome measure (Havrdova et al 2017, Arnold et al 2017), reflecting the new treatment concept of MS: complete stabilization of the disease process. In those cohorts NEDA ranges from 25-40% (Havrdova et al 2017, Arnold et al 2014 & 2017, Miller et al 2014, Nygaard et al 2015). It is important to note however, that the concept of NEDA also includes progressive neurological decline (such as is due to progressive disease and neurodegeneration). The percentage of patients that is free from inflammation is therefore somewhat underestimated in the numbers of these studies. Furthermore, most studies comprise on average a relatively short observation period of 2 years.

Three cohort studies exist with a longer follow-up. De Stefano et al, Uher et al and Rotstein et al published cohort studies that describe a longitudinal follow-up of NEDA status of ~ 200 patients for 7-10 years[33-35]. They showed that a fairly low percentage of patients remained

NEDA after 5 years (10-15%). In the cases of emerging inflammatory activity under treatment, most patients showed disease activity in the first 1-2 years after the start of treatment. Only a small minority of patients lose their NEDA status after 5 years (4%). It is very important to note that in the Rotstein-study the loss of NEDA status was in a large proportion due to disease progression rather than new inflammation. This causes a relative underestimation of patients who remain inflammatory stable. Taken together and based on this data, we assume that in the of all first-line DMT users 10% show no signs of inflammation for 5 years, and only a very small percentage of patients shows additional disease activity while staying on treatment (5%). The majority (95%) of patients remain inflammatory stable while continuing their medication.

This makes 700-750 patients eligible for inclusion.

4.2 Inclusion criteria

In order to be eligible to participate in this study, a subject must meet all of the following criteria:

1. A minimum age of 18 years
2. Ability to understand the purpose and risks of the study and provide signed and dated informed consent and authorization to use protected health information (PHI) in accordance with national and local privacy regulations.
3. Definite diagnosis of relapsing-onset MS according to the revised McDonald 2017 criteria
4. All relapsing-onset MS patients treated with one of the first-line treatments: any of the interferons, glatiramer acetate, dimethylfumarate, teriflunomide
5. Complete absence of inflammatory activity (no objectively defined and confirmed relapses, no significant number (2 or more) of new-T2 lesions suggestive of demyelination and no contrast-enhancing lesions) suggestive of demyelination for 5 consecutive years under first-line treatment. In case the last available MRI-scan was conducted 10 or more years ago, no more than 3 new T2-lesions suggestive of demyelination in the last 10 years are accepted.

4.3 Exclusion criteria

A potential subject who meets any of the following criteria will be excluded from participation in this study:

1. A switch between first-line disease modifying therapy over two years prior to inclusion, in case the switch has been due to in effectivity of the first DMT. In case the switch has been due to side-effects or by a personal preference of the patient (such as the wish to switch to oral therapies), this is not considered as an exclusion criterium.
2. Women who want to discontinue medication because of a pregnancy wish and women who are pregnant or expect to become pregnant during the study period
3. Patients that have previously used interferon-beta and have been tested positive for neutralizing antibodies (NAbs). This is determined by measuring MxA-bioactivity and is a test that is part of routine follow-up in patients that use interferon-beta. The reason for this is that development of NAbs has been shown to affect interferon-beta treatment efficacy.

4.4 Sample size calculation

The stability of patients in the continuation group is estimated to be at least 97,5%. Based on a non-inferiority margin of 7,5%, a preliminary power-calculation based on the non-inferiority principle was performed (PASS v12, one-sided Z-test (unpooled), significance level 0.05) and showed a necessary sample size of 54 per group to achieve 80% power. Taking 20% drop out into consideration, the total sample size needed for this study is 130.

The applications MS Sherpa and Neurokeys will be added to collect digital biomarkers regarding (return of) inflammatory disease activity. Based on previous experience, our estimate is that the current sample-size can provide some conclusive trends on the association between digital biomarkers and (return of) inflammatory disease activity.

Update inclusion status February 2023

In accordance with the most recent DSMB meeting that took place in January 2023, from the 26th of January 2023 onwards no new patients will be included in the study. New inclusions will be paused for six months, and after these six months the inclusion pause will be re-evaluated.

TREATMENT OF SUBJECTS

4.5 Investigational product/treatment

The intervention consists of the discontinuation of the previously used DMT (either interferons, glatiramer acetate, dimethylfumarate or teriflunomide) Based on the pharmacological profile of the abovementioned drugs that are eligible for inclusion, there is no need for a tapering of dosage before complete discontinuation.

4.6 Use of co-intervention

During the intervention period patients are asked not to participate in any other scientific studies. Patients are allowed to use all types of co-medication, except for immunomodulating drugs such as prescribed for the treatment of multiple sclerosis and/or other auto-immune diseases.

4.7 Escape medication

When disease activity emerges, patients are treated according to the current standard of care (including intravenous methylprednisolone if deemed necessary) and disease modifying treatment will be reinitiated. Unscheduled visits including an MRI-scan are planned for each patient with any new neurological complaints, as is standard clinical procedure.

5. INVESTIGATIONAL PRODUCT

N/A

6. NON-INVESTIGATIONAL PRODUCT

MS Sherpa and Neurokeys are CE-certified medical devices. See for the relevant details appendices “D6 - Aanvullende productgegevens MS sherpa” and “D6 – Aanvullende productgegevens Neurokeys”.

7. METHODS

7.1 Study parameters/endpoints

7.1.1 Main study parameter/endpoint

The primary endpoint is number of patients with return of inflammatory disease activity after 2 years based on: a clinically confirmed relapse (defined according to the definition most often used in MS phase-III trials: the onset of new or recurrent symptoms that last > 24 hours, that are accompanied by new objective abnormalities on a neurological examination and that are not explained by non-MS processes such as fever, infection, severe stress or

drug toxicity (Gold et al NEJM 2012)) , or any emerging subclinical disease activity proven to be due to active disease/new inflammation (defined as 3 or more lesions on T2—weighted images or 2 or more gadolinium enhancing lesions on T1-weighted post-contrast MRI suggestive of demyelination) in the discontinuation group.

7.1.2 Secondary study parameters/endpoints (if applicable)

Secondary end-points are

- Changes in neurological functioning
    - EDSS change (Including individual functional systems)
    - MSFC changes
        - Timed 25-foot Walk (T25fW)
        - 9-Hole Peg Test (9HPT)
        - Symbol digits modalities test (SDMT)
- Individual MRI-parameters
    - T1 post-contrast lesion numbers and volumes
    - T2 lesion numbers and volumes
    - Whole brain volume
    - Normalized white matter volume
    - Grey matter volume
- Changes in quality of life measurements
    - Multiple Sclerosis Impact Scale (MSIS-29)
    - Short Form health survey (SF-36)
    - Multiple Sclerosis Self-Efficacy scale (MSSE)
    - Checklist Individual Strength (CIS20r)
    - Treatment Satisfaction Questionnaire for Medication (TSQM)
- Cost measurements
    - EuroQol 5 dimensions questionnaire (EQ-5D-5L)
    - Medical consumption questionnaire (iMCQ)
    - Productivity costs questionnaire (iPCQ)
- Changes in biomarker measurements
    - Neurofilament levels
- OCT and eye movement measurements
    - Peri-papillary retinal nerve fiber (RNFL) thickness
    - Macular ganglion cell-layer inner plexiform layer (GCL-IPL) thickness
    - Eye movement measurements

- Changes in digital biomarkers using the NeuroKeys (CE) and MS sherpa (CE) mobile applications that measure:
  - Walking test (2-minute walking test)
  - Cognition test (similar to SDMT)
  - MS sherpa questionnaires (including fatigue)
  - Keystroke data

7.1.3 Other study parameters (if applicable)

Vitamin D, smoking high body-mass index (BMI) are considered as potential confounders. Therefore, vitamin D will be determined and patients will be asked about smoking behaviour during every visit. To avoid any potential bias, we will also collect data (if present in the individual patients) on disease activity prior to the initiation of DMT, such date of diagnosis, time from first symptoms to diagnosis, EDSS scores/MS severity at the moment of DMT initiation.

7.2 Randomisation, blinding and treatment allocation

Since MS affects 2-2.5x as many women as men, we expect to include more women than men. A randomization algorithm will be included to match both groups for sex and age to exclude potential bias.

Outcome measurements will be performed by assessors who are blind to the intervention assignment. For reasons of consistency and to exclude possible bias, all scans will be centrally reviewed in VUmc by a radiologist blinded to allocation to the intervention group. In the current set-up of the trial patients and their treating neurologists are unblinded to the randomization group. The currently available first-line disease modifying treatments consist of the various (peg)interferons, glatiramer acetate, teriflunomide and dimethylfumarate. It comes in a total of more 10 different forms with different packaging, different modes of injection (some subcutaneously, some intramuscular and some oral) and different frequencies of intake (ranging from twice daily to once every two weeks). We have set up the trial so that it is rater-blinded; all MRI-scan and clinical evaluations (such as the neurological examination) will be performed blinded to “treatment” allocation. The primary and majority of secondary outcome measures are derived from these blinded assessments. This approach is frequently chosen in MS research (even in the phase-III trials) for 2 very important reasons. Due to the very distinct nature of side-effects (flushing, gastro-intestinal problems, flu-like symptoms), patients know when they receive placebo instead of the actual active compound. The invasive nature of receiving placebo-injections would greatly enhance the possibility of patients not participating. In addition, we believe it is too costly to create a placebo-control for each of the 10 different forms of medication.

7.3 Study procedures

No study procedures will take place before having obtained informed consent which will be gained following current METc/CCMO standards.

Follow-up frequency

Patients in the continuation group are expected to remain stable throughout the study period based on their proven stable status for 5 consecutive years prior to enrolment. The minimum evaluation that must take place should have a frequency according to the current standard of care (yearly evaluation of every patient treated with immunomodulating therapy; i.e. baseline (BL), 12 and 24 months). Timely recognition of recurrence of any (subclinical) disease activity in the discontinuation group is secured by more frequent clinical and radiological assessment during extra routine study visits at 3, 6 and 18 months. For optimal comparability and to overcome potential bias , both groups will be followed with a complete assessment including MRI at BL, 3, 6, 12, 18 and 24 moths. For the patients in both groups mobile applications MS sherpa and Neurokeys will be installed. With the MS sherpa application, patients will be asked to perform tests every two weeks during the 24 months of follow-up. The Neurokeys application will collect data on the background of the standard use of mobile phones during this time period.

Clinical evaluation

Duration: 1 hour

Clinical evaluation will consist of a careful medical history: current and past medication, adverse events, number of intravenous methylprednisolone treatments, number of relapses, date of last relapse and signs of symptom progression. A relapse is defined according to the definition most often used in MS phase-III trials: the onset of new or recurrent symptoms that last > 24 hours, that are accompanied by new objective abnormalities on a neurological examination and that are not explained by non-MS processes such as fever, infection, severe stress or drug toxicity[21]. Furthermore, the Expanded Disability Status Scale (EDSS) and Multiple Sclerosis Functional Composite (MSFC)-measurements will be performed, consisting of the Timed 25-foot Walk (T25fW), 9-Hole Peg Test (9HPT) and the symbol digits modalities test (SDMT). This will be done by a blinded investigator.

Radiological evaluation

Duration: 45 min

Radiological evaluation will consist of repeated brain MRI investigations that consist of conventional pre- and post-contrast (T2-weighted, T1-weighted pre and post contrast, FLAIR) images. All scan protocols are available in general and academic hospitals since they form the basis on which MS is diagnosed and treatment is monitored. Although it is expected that new inflammatory lesions can be captured by repeated T2-weighted/FLAIR MRI-scans, a gadolinium-scan is included to not miss any contrast enhancement in previously present lesions. A 3DT1 and 3DFLAIR image will also be made for atrophy measurements. For reasons of consistency and to exclude possible bias, all scans will be centrally reviewed in VUmc by a radiologist blinded to allocation to the intervention group.

Questionnaires

Duration: 1 hour

For the evaluation of disease burden and MS related symptoms, we will use the validated and in clinical studies often used Multiple Sclerosis Impact Scale (MSIS-29)[36] Short Form health survey (SF-36)[37] and CIS20r[38]. The perceived impact of treatment, convenience, satisfaction and side-effects will be monitored using the Treatment Satisfaction Questionnaire for Medication (TSQM)[39]. For the evaluation of health related quality of life (HRQoL) and the link between symptoms HRQoL and costs, we use the EDSS for the objective measurement of changes in neurological functioning (which has a clear relation to HRQoL assessed as utility and costs[40]) and the number of patients with return of inflammatory activity. We also use the EQ5D-5L) for cost-utility analysis[41]. Furthermore, we will ask patients to keep a diary describing changes in healthcare consumption (which will be defined per item in a questionnaire (such as hospitalisations, consultations with doctors, use of care at home, use of specialized transportation etc). Lastly patients will be asked for their employment situation and short-term and long-term sick absence because of MS (or MS related treatment) using the iMCQ and iPCQ questionnaires[42]. Questionnaires are filled in digitally either at home or during the hospital visit. Help of a carer or the study-nurse is allowed in cases of the inability using a computer.

Samples

Duration: 15 minutes

Blood collection will take place in both patient groups at every visit. It will consist blood collection for the purpose of biobanking and for diagnostics in the case of any –unforeseen-clinical events. Furthermore, we will retrospectively measure neurofilament light in serum using Simoa.

All participants will visit the hospital for 6 times over a time frame of 2 years. Each of these visits that take place will take approximately 2,5-3 hours (clinical assessment and MRI-scan). Also 1-hour questionnaires which can be completed at home digitally or at the hospital on paper will take place before/during each visit.

All samples will be collected, processed and stored according to the Standard Operating Procedures (SOP's) as described in the Parelsnoer Biobankprotocol version 8.0. (15) Samples will be stored at the Biobank VUmc. To ensure patient privacy all samples will be coded. A peripheral blood sample will be collected, in total 8 tubes of blood will be drawn (5x EDTA 4 ml for plasma, cells and DNA isolation, 2x serum 5 ml, 1x PAXgene tube 2 ml), adding up to a total volume of 32 ml. Blood will be centrifuged, divided in aliquots of 0.5 ml and then stored at -80 °C.

Optical coherence tomography (OCT)

Duration: 15 minutes

Frequency: yearly (at baseline or month 3, month 12 and month 24)

All OCT measurements will be performed by a certified person (not necessarily a physician) on a spectral-domain OCT machine (Spectralis by Heidelberg engineering). Scans to be performed in both eyes:

- Circular scan, centered on optic nerve head (ONH)
- Volume scan, centered on macula.
- Volume scan, centered on optic nerve head (ONH)

Eye movement examination

Frequency: yearly (at baseline or month 3, month 12 and month 24)

Duration: 15 minutes

Eye movement measurements will be performed using Eyelink 1000 Plus Eye Tracker (SR Research). Built-in algorithms provided by the eye tracker are used for calibration and validation procedures. The experiment consists of the following assessments:

1. Fixation
2. Pro-saccades
3. Anti-saccades

Mobile applications measurements

Duration: 5 minutes

Frequency: once every two weeks.

For eligible patients (based on regular smartphone use), mobile applications MS-Sherpa and Neurokeys will be installed on their smartphones and data will be collected via these applications.

MS sherpa mobile application

Via the MS sherpa application the patient will be asked to perform walking tests, cognition tests and the MS sherpa questionnaire (including fatigue).

- Cognitive task: similar to the SDMT, the participant is requested to assign numbers to corresponding symbols according to a specific displayed coding.
- Motor task: 2-minute walk test (2MWT): the participant is requested to walk (either unassisted or with a walking aid) for two minutes. The walking distance is measured through the location data. Patients who are unable to walk for two minutes will not be requested to do the 2MWT.
- MS sherpa questionnaire: patient reported outcomes on Likert scales (amongst others about fatigue and the impact of MS on daily activities).

These tests can be completed in approximately 5 minutes and patients will be asked to complete these tests once every two weeks.

NeuroKeys mobile application

NeuroKeys replaces the standard keyboard of the patient's smartphone. Data is collected from regular use of the keyboard, and no additional action from patients is needed. After 24 hours of inactivity of the keyboard of NeuroKeys, either intentionally or unintentionally, a push notification is automatically send utilizing Amazon Simple Notification Service (SNS). NeuroKeys will collect keystroke data general profile information (gender, year of birth, and MS type), and metadata (iOS/Android version and NeuroKeys version). The data will be collected from the phone continuously in a retrospective fashion, when the keyboard is activated the data from the previous keyboard typing session is sent.

- Keystroke data: the start of a message is defined as the keyboard flipping up and the end of a message is marked when the keyboard flips down. Specific keys will be logged and timestamped to be able to accurately calculate parameters expected to be associated to fatigue. The keys logged are: delete or backspace key, dot key, space bar, semicolon, colon, parentheses, capitalized characters, numbers and punctuation marks denouncing the ending of a sentence. All numbers and all punctuation marks will be logged as the same number or punctuation event (e.g. we do not make a distinction

between the number 3 and 8 or the comma and dollar sign). Parameters such as word count, amount of words comprised of six characters or more and latency between key presses will be calculated on the basis of the logged keys. In addition to ASCII keys, the unicodes of emojis will also be logged.

- Sensor data: Data from the location sensor, ambient light sensor, gyroscope, motion and accelerometers of the smartphone will be collected when the keyboard is in use. With the location sensor (longitude and latitude), the keystroke data can be combined to open source databases (e.g. weather data from the Royal Netherlands Meteorological Institute, KNMI) to examine the influence of external factors (Davis et al. 2010). Existing and future open source databases provided by public or governmental institutes can be accessed for this purpose. Ambient light sensor can detect environmental brightness which could impact typing behaviour. Kinematic sensors (gyroscope, motion and accelerometers) may be used to infer posture of the user (Lamonaca et al. 2015).

7.4 Withdrawal of individual subjects

Subjects can leave the study at any time for any reason if they wish to do so without any consequences. The investigator can decide to withdraw a subject from the study for urgent medical reasons.

7.4.1 Specific criteria for withdrawal (if applicable)

There are no specific criteria for withdrawal from the study.

7.5 Replacement of individual subjects after withdrawal

There will be no replacement of individual subjects after withdrawal.

7.6 Follow-up of subjects withdrawn from treatment

If a patient is withdrawn from the study, we will still perform follow-up measurements in case the patient is willing and able to cooperate.

7.7 Premature termination of the study

The procedure in case of premature termination of the study is described in section 10.4 and 12.5.

8. SAFETY REPORTING

8.1 Temporary halt for reasons of subject safety

In accordance to section 10, subsection 4, of the WMO, the sponsor will suspend the study if there is sufficient ground that continuation of the study will jeopardise subject health or safety. The sponsor will notify the accredited METC without undue delay of a temporary halt including the reason for such an action. The study will be suspended pending a further positive decision by the accredited METC. The investigator will take care that all subjects are kept informed.
More information about temporary halt for reasons of subject safety is provided in section 10.4 and 12.5.

8.2 AEs, SAEs and SUSARs

8.2.1 Adverse events (AEs)

Adverse events are defined as any undesirable experience occurring to a subject during the study, whether or not considered related to the experimental intervention. Adverse events that are reported spontaneously by the subject or observed by the investigator or his staff and that are relevant to the study will be recorded. Relevant adverse events are possible side-effects of the DMT used and the occurrence of relapses.

Adverse Device Effect (ADE)
An ADE is an adverse event related to the use of an investigational medical device. This includes any adverse event resulting from insufficiencies or inadequacies in the instructions of use, the deployment, the installation, the operation, or any malfunction of the investigational medical device. This also includes any event that is a result of a use error or intentional misuse.

8.2.2 Serious adverse events (SAEs)

A serious adverse event is any untoward medical occurrence or effect that

- results in death;
- is life threatening (at the time of the event);
- requires hospitalisation or prolongation of existing inpatients' hospitalisation;
- results in persistent or significant disability or incapacity;
- is a congenital anomaly or birth defect; or

- any other important medical event that did not result in any of the outcomes listed above due to medical or surgical intervention but could have been based upon appropriate judgement by the investigator.

A SADE is an ADE that has resulted in any of the consequences characteristic of a serious adverse event.

The investigator will report all SAEs and SADEs to the sponsor without undue delay after obtaining knowledge of the *events*. The sponsor will report the SAEs through the web portal *ToetsingOnline* to the accredited METC that approved the protocol, within 7 days of first knowledge for SAEs that result in death or are life threatening followed by a period of maximum of 8 days to complete the initial preliminary report. All other SAEs will be reported within a period of maximum 15 days after the sponsor has first knowledge of the serious adverse events.

8.2.3 Suspected unexpected serious adverse reactions (SUSARs)

This section is not applicable since this study does not investigational medicinal products.

8.3 Annual safety report

This section is not applicable since this study does not investigational medicinal products.

8.4 Follow-up of adverse events

All AEs will be followed until they have abated, or until a stable situation has been reached. Depending on the event, follow up may require additional tests or medical procedures as indicated, and/or referral to the general physician or a medical specialist.
SAEs need to be reported until the end of study, as defined in the protocol in section 9.2.2.

8.5 Data Safety Monitoring Board (DSMB)

For optimal safety we will appoint an independent data safety monitoring board (DSMB)which will monitor trial data on a regular basis. The aim of the committee is to safeguard the interests of trial participants and assess the safety of the discontinuation of therapy during the trial. The specific role of the DSMB consists of monitoring evidence for harm due to the intervention (discontinuing medication). The DSMB may advice to terminate the trial prematurely if disease activity exceeds above mentioned thresholds (see section 10.4 for interim analyses).

The DSMB consists of 4 members who do not have conflict of interest with the sponsor or the study. In addition to the permanent members of the DSMB two external members are added to the DSMB with expertise in the relevant field of research (yet to be determined).

The DSMB will conduct interim analyses in a predetermined manner and at predetermined times (see section 10.4), to see whether the relationship between clinical benefit and burden remains acceptable to the subject during the study. After each interim analysis, the DSMB reports to the study coordinator, with reports to the METc and the study sponsor, i.e. the Board of Directors (Raad van Bestuur). The DSMB can give advice to continue, change or stop (parts of) the study. The DSMB will also ensure the quality and safety of research in the participating centers.

9. STATISTICAL ANALYSIS

All data is quantitative and will be presented in tables and graphs. Baseline data will be collected to detect any potential differences between the two investigated groups for which we have not corrected with the randomization procedure (sex and age). Possible other confounders include smoking habits, vitamin D levels but also previous disease course in terms of differences in disease duration, number of relapses prior to stability, years of use of treatment.

The primary endpoint is the number of patients with return of inflammatory disease activity after 2 years based on: a clinically confirmed relapse or any emerging subclinical disease activity proven to be due to active disease/new inflammation (defined as 3 or more lesions on T2—weighted images or 2 or more gadolinium enhancing lesions on T1-weighted post-contrast MRI) in the discontinuation group. Secondary end-points are: 1. Changes in neurological functioning (EDSS/MSFC changes including individual functional systems and MSFC subtests), 2. Individual MRI parameters (T2 and T1 post-contrast lesion numbers), 3. changes in quality of life measurements (SF-36, CIS20r, TSQM, EQ5D-5L, iMCQ and iPCQ) and 4. changes in biomarker measurements (neurofilament levels).

In the case of confirmation of our hypothesis (discontinuing medication after >5 years without evidence of inflammatory disease activity does not result in return of inflammatory disease activity), we will extend the trial with a follow-up of two years where the group that continued their treatment gets offered the possibility to discontinue under similar safety measures (with similar follow-up frequencies and endpoints as the primary trial) and including the possibility to use MS sherpa and Neurokeys.

9.1 Primary study parameter(s)

For the primary endpoint, the return of inflammatory disease activity after 2 years, a2x2 contingency table will be used to estimate the risk difference for the return of inflammatory disease activity after 2 years (yes/no) between the two groups. The risk difference will be calculated for discontinuation relative to continuation. If the lower bound of the corresponding two-sided 90% confidence interval exceeds -7.5% we conclude non-inferiority of discontinuing medication For the primary endpoint both a per protocol analysis and a intention-to-treat analysis will be performed.

The primary endpoint is the number of patients with return of inflammatory disease activity after 2 years based on: a clinically confirmed relapse or any emerging subclinical disease

activity proven to be due to active disease/new inflammation (defined as 3 or more lesions on T2—weighted images or 2 or more gadolinium enhancing lesions on T1-weighted post-contrast MRI suggestive of demyelination) in the discontinuation group.

A relapse is defined according to the definition most often used in MS phase-III trials: the onset of new or recurrent symptoms that last > 24 hours, that are accompanied by new objective abnormalities on a neurological examination and that are not explained by non-MS processes such as fever, infection, severe stress or drug toxicity[21]

Furthermore, a survival analysis (with an intention-to-treat approach) regarding the time to return of inflammatory activity will be included.

9.2 Secondary study parameter(s)

For all the secondary endpoints correlation and regression analysis (either linear of logistic, depending on the type of variable) will be performed correcting for possible confounders. On the app data, classification analyses and machine learning models will be used next to the more conventional analyses.

9.3 Other study parameters

N/A

9.4 Interim analysis

We will build in a safety-strategy (go-no-go strategy) to control for emerging disease activity (and patients safety). Interim analyses will be done after inclusion of the 40th, 70th and 100th patient. In both the continuation and discontinuation group, the proportion of patients that showed return of inflammatory disease activity – defined as an objectified MS relapse or 3 or more lesions on T2-weighted MRI-images or 2 or more gadolinium enhancing lesions on T1-weighted post-contrast MRI suggestive of demyelination – will be counted and compared between the treatment arms.

If there are more patients with return of inflammatory disease activity (according to the above mentioned definition) in the discontinuation group than in the continuation group, and the 95% confidence interval of the difference in the proportion of patients with return of disease activity between both groups does not include 0, we will discuss premature ending of the study with the DSMB.

For optimal safety the DSMB will monitor the decision making on premature termination every 3 months. The DSMB may advice to terminate the trial prematurely if disease activity exceeds above mentioned thresholds. See section 9.5 for a more detailed description of the DSMB.

The procedure in case of premature termination of the study is described in section 12.5.

In accordance with the most recent DSMB meeting that took place in January 2023, from the 26th of January 2023 onwards no new patients will be included in the study. New inclusions will be paused for six months, and after these six months the inclusion pause will be re-evaluated. All new significant inflammatory disease activity (relapses and/or 3 or more lesions on T2-weighted MRI-images or 2 or more gadolinium enhancing lesions on T1-weighted post-contrast MRI suggestive of demyelination) will be reported to the DSMB directly after the sponsor became aware of it.

9.5 Cost-analysis

Alongside this trial, we will conduct a cost-effectiveness analysis and a cost-utility analysis over a 2-year follow-up. These will be performed in accordance with the recommendations of the Dutch guideline for health economic evaluations. For the cost-effectiveness analysis, the return of inflammatory disease activity (either relapse or new of enlarging lesions) will serve as the effectiveness measure. The cost-utility analysis will focus on quality of life measured with the EQ-5D-5L, which is often used in MS research. Quality-adjusted life-years (QALYs) will be calculated by multiplying the utility scores belonging to a health state by the amount of time spent in this health state using linear interpolation between time points.

In both analyses, costs will be measured from a societal perspective including health-care costs (such as the costs for a year of DMT, costs for the extra surveillance including additional medical tests as MRI, costs for visits to other medical doctors etc), direct nonmedical costs (costs that patients make for travelling to and from the hospital, private payments for extra health-care consumption etc) and indirect nonmedical costs (costs due to loss of production and short or long-term sick absence). The latter is important as previous research has shown that productivity losses are an important cost driver in severe MS. Health-care costs and direct nonmedical costs will be measured using the iMTA Medical Consumption Questionnaire (iMCQ) at 3 months, 6, months, 12 months, 18 months and 24 months. The iMCQ measures the health-care costs in the last three months. As these patients are assumed to be stable regarding their disease progression, these follow-up

moments will provide an adequate estimation of their health-care use. Primary and secondary health-care costs will be valued using Dutch standard costs. If unavailable, tariffs or costs reported by the literature will be used. Medication will be valued using www.medicijnkosten.nl whereas informal care will be valued based on the costs of household care as reported by the Centraal Administratie Kantoor.
For indirect nonmedical costs, patients will be asked for their employment situation and both short-term and long-term sick absence because of MS (or MS related treatment) using the iMTA Productivity Cost Questionnaire (iPCQ) at 3 months, 6, months, 12 months, 18 months and 24 months. The iPCQ measures productivity loss in the last four weeks which will give an adequate overview of the productivity losses between each time point as these patients are assumed to be stable. Costs of absenteeism from paid work will be calculated according to both the human capital and friction cost approach. Costs of presenteeism will be calculated by asking participants how many working hours should have been replaced due to less productivity at work. Lost productivity due to either absenteeism or presenteeism will be valued using the mean age-, and sex specific income of the Dutch population. Costs of productivity losses due to absenteeism from unpaid work and informal care will be calculated using the standard wage of a professional housekeeper. All costs will be indexed to the year at which the trial ended. Missing data on costs and effects will be imputed using multiple imputations. In addition, costs and effects will both be discounted using a 3% discount rate.

For the cost-effectiveness analysis, we will calculate incremental cost-effectiveness ratios (ICERs) which is defined as the difference in mean costs divided by the difference in mean effects between the treatment continuation group and the treatment discontinuation group. For the cost-utility analysis, we will calculate incremental cost-utility ratios (ICURs) which is the difference in mean costs divided by the difference in mean QALYs. Bootstrapping with 5,000 replications will be used to estimate 95% credibility intervals around the ICERs and ICURs. The bootstrapped cost-effect pairs will be plotted on a cost-effectiveness plane and used to estimate cost-effectiveness acceptability curves (CEACs). CEACs show the probability that the intervention is cost-effective in comparison with the control treatment for a range of ceiling ratios. The ceiling ratio is defined as the willingness-to-pay, which is the amount of money society is willing to pay to gain one unit of effect.

In a sensitivity analysis, we will repeat all analyses using a healthcare payer perspective. In this analysis, only direct healthcare costs will be included. In addition, we will conduct subgroup analysis in which we will stratify individuals based on the presence of blood-based markers predictive for return of inflammatory disease activity.

Furthermore, we will conduct a budget impact analysis. A budget impact analysis (BIA) focuses on the expected changes in the expenditure of a health care system after the adoption of a new standard of care. In this BIA, we aim to estimate the future yearly budget impact of discontinuation of first line medication in patients with long-term stable relapsing-onset from a Dutch perspective. The BIA will be performed according to the BIA framework of the International Society for Pharmacoeconomics and Outcomes Research (ISPOR). This framework consists of several standard aspects: target population, scenario distribution based on hospital types, resource utilisation, costs per unit, total costs, and sensitivity analyses.

10. ETHICAL CONSIDERATIONS

10.1 Regulation statement

The study will be conducted according to the principles of the Declaration of Helsinki (World Medical Association, 2013, Brazil) and in accordance with the Medical Research Involving Human Subjects Act (WMO) and the Good Clinical Practice guidelines.

10.2 Recruitment and consent

Patients will be informed about the study in different ways. Patients can be notified by their treating doctor during outpatient consultations at the VUmc or one of the other participating centres. In addition, a notification will be placed on the website of the MS Centre Amsterdam and the websites of the MS Vereniging Nederland (MSVN) and MS Web with information about the study. Patients can then discuss potential interest in the study with their treating neurologist. Inclusion is possible in one of the participating centers. Potential participants who express their wish to participate will receive additional information on paper about the purpose, intervention, duration and content of the study. They will also receive an informed consent form with careful and extensive information about the possible risks (according to local ethical review board requirements). In case there are any questions about the study, the study coordinator can be contacted. Moreover, an independent doctor (dr. Pijnenburg) can be contacted for additional questions.

Inclusion will take place after the written informed consent form has been returned to the trial coordinator. A copy of the informed consent form will be given to the participant and to the responsible physician in one of the participating centers in case the patient is recruited through one of the centers outside the VUmc. This consent can be revoked at any time without citing reasons. Patients will be given a minimum of 2 weeks to consider their decision. The maximum time will be as long as the inclusion of patients is ongoing and inclusion and exclusion criteria are met.

10.3 Objection by minors or incapacitated subjects (if applicable)

Not applicable, all participants will be adult and legally competent

10.4 Benefits and risks assessment, group relatedness

In the non-intervention group (continuation group) participants are expected to remain stable throughout the study period based on their proven stable status for 5 consecutive years prior to enrolment. Therefore, this group will face no potential risks and no direct benefit other than the usual medical care. The potential value of the outcome of the research will outweigh the

burden of participation for the study. Also, patients in the continuation group are offered the possibility to discontinue their medication under similar surveillance measures as the discontinuation group after 24-month follow-up period has ended.

The data on the discontinuation of therapy in long-term inflammatory stable MS patients is reassuring. One large retrospective cohort study has shown in 1200 patients that stable RRMS patients who stop treatment don’t have an increased relapse rate compared to patients who continue treatment[25]. There was a slightly higher disability progression of the discontinuation group, presumably based on the discontinuation of treatment in patients with progressive MS. Also, there seems to be no rebound inflammation after discontinuation of therapy in progressive MS, and a similar relapse rate as the years prior to discontinuation[26]. Lastly, one study has showed that patients 45 years or older, or patients with a DMT intake of 4 or more years without evidence of clinical or radiological disease activity showed a high likelihood of remaining relapse-free after discontinuation and absence of contrast enhancing lesions[27]

10.5 Compensation for injury

According to article 7 from the ‘Wet medisch-wetenschappelijk onderzoek met mensen’ (Staatsblad 1998, 161) an insurance is obtained by the VUmc. In case of injury or death of the participants because of the study, this insurance will compensate for injury or cover the cost caused by death or injury from the participants. The insurance is obtained by the Onderlinge Waarborgmaatschappij Centramed b.a., Postbus 7374, 2701 AJ Zoetermeer. The insurance company and the insurance accede to the decree mandatory insurance for ‘medisch-wetenschappelijk onderzoek met mensen (Staatsblad 2003, 266). Written information about the insurance will be provided for the participants.

10.6 Incentives (if applicable)

According to the current standard of care, the evaluation of every patient treated with immunomodulating therapy is at baseline, 12 and 24 months. Since visits are also scheduled at 3, 6 and 18 months, travel expenses and parking costs for these extra visits will be compensated. Participants will be compensated for their time and effort for study participation: they will receive a gift certificate of €25 upon study completion.

11. ADMINISTRATIVE ASPECTS, MONITORING AND PUBLICATION

11.1 Handling and storage of data and documents

Data will be handled confidentially . After collection, all data will be correctly labeled and securely stored. A subject identification code (SIC) will be used to link data to the subject. The SIC will consist of numbers and will not be based on the patient initials and birth-date. The key to the code will be kept separately from the coded data. The only people who have access to this code will be the principal investigator, the coordinating investigator and the corresponding investigator. No other people will have access to the link information. Great care will be taken to ensure that there is no link between SIC and information on which an individual can be identified. The handling of personal data in the database complies with the General Data Protection Regulation (De Algemene Verordening Gegevensbescherming) Potential data exchange with other countries will only take place after consent of the patient and handling of data will comply with the General Data Protection Regulation. The procedure for handling data includes data encryption, coding, secure storage, establishing limited access or varying levels of access to the biobank, removing identifying information from bio specimens and data. The infrastructure will consist of both hardware and software components, to prevent unauthorized access to databases.

An electronic case report form (CRF) will be developed to document the data collected in the study. This database will include demographic and patients characteristics (without birth date) and all outcomes of the study measures. Other investigators can request permission to get access to (a part) of this database for the purpose of research only, and only when the principal investigator gives permission. These investigators will not get access to the separate database which includes the participants' names, other identifiers and the SIC. All data will be stored on a computer protected with a password on the VUmc computer network. And access to the database will also be secured by a code. Only the trial coordinator and the principal investigator will know the code that gives access to the database with the link information.

After finalizing the study, the originals of all source documents will be stored for a period of 15 years in a locked room. Data that is collected and stored for the Biobank, will be stored for a period of 50 years. This period of storage has been determined to ensure that a follow-up study might be possible. In case of a follow-up study, a new protocol will be submitted to the METC and participants will have to sign a new informed consent form. Importantly, participants will only be approached for a follow-up study if they have indicated on the

informed consent form of the current study that they can be approached for a follow-up study.

The collection of data for medical research in the Netherlands is subject to the Personal Data Protection Act and in particular to the Medical Treatment Contracts Act.

Data storage Neurokeys

Data collected by NeuroKeys will be stored using Amazon AWS S3. There will be no identifiable information in this database, all keystroke and sensor data are logged with ID numbers only. A separate database, Amazon RDS (SQL server), will be used to store the verification code and personal information such as gender and year of birth, which can be used to send users push notifications. Both Amazon AWS S3 and Amazon RDS databases are located in Frankfurt, Germany, and are ISO 27001/27017/27018 compliant. All data is AES-256 encrypted in transit and at rest, a tokenization approach is used in which a sensitive data element is replaced by a non-sensitive equivalent and send by using a secure SSL link. Decryption keys are stored in a private encrypted environment. For iOS users, each time the NeuroKeys' keyboard is 'activated' (i.e. a new message is started), data of the previous keyboard session is uploaded to the database. Data of only one message is saved on the mobile phone, until a new message has started. For Android users data is uploaded to the database approximately every 4 hours.

Data storage MS sherpa

Data collected by MS sherpa will be stored using MongoDB Atlas, whose infrastructure runs on top of Amazon Web Services (ISO 27001/27017/27018 compliant) in Dublin, Ireland. General profile information (such as gender, age, length, weight, and e-mail address) is directly saved on AWS servers, in Dublin and AWS S3 in Frankfurt, Germany. Auth0 is used for user authentication and authorisation (ISO 27001/27018 compliant) and its EU office is based in London, UK. MongoDB Atlas and Auth0 achieved key compliance controls and objectives, as demonstrated by the completion of a Type 1 SOC 2 Report: Security. MongoDB, Inc. and Auth0 are also certified under the EU-US Privacy Shield. Data gathered via MS sherpa will be transferred to the database immediately after the data has been collected.

11.2 Monitoring and Quality Assurance

An independent monitor, the Clinical Research Bureau (CRB) of the VUmc, will monitor the proposed study according to Good Clinical Practice (GCP). For a selection of candidates

Informed consent is to be checked by the CRB. Besides that, source data verification is performed during the onsite monitoring. The conformity of the data used for analysis and the information in the patient files will be checked by the CRB. The intensity of the verification will be related to the risk arisen by the research. Inclusion and exclusion criteria will be checked as well as the main outcome measures. The CRB will check if the (S)AE's and SUSAR's are reported conforming the schedule as required by laws and regulations.

The quality assurance team under the leadership of a quality assurance manager (QAM) is responsible for providing an effective and efficient quality assurance system and counsel for the clinical research sites. In this quality assurance system, the QAM is responsible for ensuring appropriate global and affiliate-specific quality documents are developed and tracked, making sure they maintain an up-to-date overall inventory of all quality documents. Furthermore, the QAM and its team are responsible for ensuring all personnel involved in the clinical trial are properly qualified and trained for the job roles for which they are responsible. They are responsible for giving the personnel trainings and constantly assessing further opportunities for education and additional training. The quality assurance team is also responsible for checking compliance with the protocol, SOPs, GCP, and/or applicable regulatory requirement(s) and checking of the quality in all stages of data handling to ensure that all data are reliable and have been processed correctly. Moreover, the quality assurance team is responsible for auditing the various investigational sites.
If noncompliance with the protocol, SOPs, GCP, and/or applicable regulatory requirement(s) by and investigator/institution, or by member(s) or the sponsor's staff is detected during a quality assurance activity or audit, it is the responsibility of the QAM to report this to the trial's sponsor and principal investigator.

Quality assurance procedures:

Quality assurance is the systematic and independent examination of all clinical trial-related activities and documentations. The quality assurance procedure focuses on clinical investigator audits and audits of clinical trial documentation.

1. Document audits:

During the document audits, the quality assurance team oversees the documents that are generated before, during or at the end of the conduct of the clinical trial. For each document, a checklist is developed based on the relevant regulatory and organizational standards and SOPs. The aim of the audits is to ensure that the information and data in the documents are

complete, clear, reliable and consistent. Documents reviewed in the document audits include the clinical study protocol, the investigator’s brochure and the clinical study report.

2. Clinical investigator audits:

The clinical investigator audits concern audits of the different research sites of the trial. They are performed to assess the site’s regulatory compliance and clinical data quality (including adherence to the protocol). Paragraph 3 will address the procedures concerning these audits.

11.3 Amendments

Amendments are changes made to the research after a favourable opinion by the accredited METC has been given. All amendments will be notified to the METC that gave a favourable opinion. All amendments will be notified to the METC and to the competent authority.

11.4 Annual progress report

The investigator will submit a summary of the progress of the trial to the accredited METC once a year. Information will be provided on the date of inclusion of the first subject, numbers of subjects included and numbers of subjects that have completed the trial, serious adverse events/ serious adverse reactions, other problems, and amendments. The METC will also be informed on the start and end date of the trial.

11.5 Temporary halt and (prematurely) end of study report

The investigator/sponsor will notify the accredited METC of the end of the study within a period of 8 weeks. The end of the study is defined as the last patient’s last visit.
The sponsor will notify the METC immediately of a temporary halt of the study, including the reason of such an action.
In case the study is ended prematurely, the sponsor will notify the accredited METC within 15 days, including the reasons for the premature termination.
Within one year after the end of the study, the investigator/sponsor will submit a final study report with the results of the study, including any publications/abstracts of the study, to the accredited METC.

11.6 Public disclosure and publication policy

This study will be registered in het Nederlands Trial Register (NTR) http://www.trialregister.nl and www.clinicaltrials.gov. Publication will be in accordance with the basic principles of

CCMO statement on publication policy. The results will be presented at (inter)national scientific meetings. The results will be published in a medical scientific journal. In none of the publication forms, participant identity will be disclosed.

12. STRUCTURED RISK ANALYSIS

12.1 Potential issues of concern

Paragraph 13.1 is not applicable.

12.2 Synthesis

The intervention in this study is the discontinuation of previously used DMT. No new products or agents are administered, nor will there be any dosage adjustments in the group that will continue their therapy. The specific DMT's that patients use prior to discontinuation, and that are used in the control group are all registered with the authorities and widely used for this specific indication. Although previous studies suggest that the risk of return of inflammatory activity after discontinuing DMT will be low in long-term stable RRMS patients (as also described in section 1), this is the main risk of the intervention. To monitor return of inflammatory activity, a safety strategy is built in, which is described in section 10.4. If safety criteria are exceeded, the study will be discontinued and DMT's will be reinitiated (in one patient group or in all patients, see section 10.4). A DSMB is appointed that will monitor the decision making on premature termination every 3 months (section 9.5).

13. REFERENCES

1 Goodin, D. S. The epidemiology of multiple sclerosis: insights to disease pathogenesis. *Handb Clin Neurol* **122**, 231-266, doi:10.1016/B978-0-444-52001-2.00010-8 (2014).

2 Weinshenker, B. G. Natural history of multiple sclerosis. *Ann Neurol* **36 Suppl**, S6-11 (1994).

3 Reich, D. S., Lucchinetti, C. F. & Calabresi, P. A. Multiple Sclerosis. *N Engl J Med* **378**, 169-180, doi:10.1056/NEJMra1401483 (2018).

4 Giovannoni, G. *et al.* Is it time to target no evident disease activity (NEDA) in multiple sclerosis? *Mult Scler Relat Disord* **4**, 329-333, doi:10.1016/j.msard.2015.04.006 (2015).

5 Arnold, D. L. *et al.* Peginterferon beta-1a improves MRI measures and increases the proportion of patients with no evidence of disease activity in relapsing-remitting multiple sclerosis: 2-year results from the ADVANCE randomized controlled trial. *BMC Neurol* **17**, 29, doi:10.1186/s12883-017-0799-0 (2017).

6 Havrdova, E. *et al.* Effect of delayed-release dimethyl fumarate on no evidence of disease activity in relapsing-remitting multiple sclerosis: integrated analysis of the phase III DEFINE and CONFIRM studies. *Eur J Neurol* **24**, 726-733, doi:10.1111/ene.13272 (2017).

7 Miller, A. E. *et al.* Oral teriflunomide for patients with a first clinical episode suggestive of multiple sclerosis (TOPIC): a randomised, double-blind, placebo-controlled, phase 3 trial. *Lancet Neurol* **13**, 977-986, doi:10.1016/S1474-4422(14)70191-7 (2014).

8 Freedman, M. S. *et al.* Moving toward earlier treatment of multiple sclerosis: Findings from a decade of clinical trials and implications for clinical practice. *Mult Scler Relat Disord* **3**, 147-155, doi:10.1016/j.msard.2013.07.001 (2014).

9 Ramsaransing, G. S. & De Keyser, J. Benign course in multiple sclerosis: a review. *Acta Neurol Scand* **113**, 359-369, doi:10.1111/j.1600-0404.2006.00637.x (2006).

10 Sartori, A., Abdoli, M. & Freedman, M. S. Can we predict benign multiple sclerosis? Results of a 20-year long-term follow-up study. *J Neurol* **264**, 1068-1075, doi:10.1007/s00415-017-8487-y (2017).

11 Benedikz, J. *et al.* The natural history of untreated multiple sclerosis in Iceland. A total population-based 50 year prospective study. *Clin Neurol Neurosurg* **104**, 208-210 (2002).

12 Perini, P., Tagliaferri, C., Belloni, M., Biasi, G. & Gallo, P. The HLA-DR13 haplotype is associated with "benign" multiple sclerosis in northeast Italy. *Neurology* **57**, 158-159 (2001).

13 Johnson, K. M., Zhou, H., Lin, F., Ko, J. J. & Herrera, V. Real-World Adherence and Persistence to Oral Disease-Modifying Therapies in Multiple Sclerosis Patients Over 1 Year. *J Manag Care Spec Pharm* **23**, 844-852, doi:10.18553/jmcp.2017.23.8.844 (2017).

14 Lattanzi, S. *et al.* Persistence to oral disease-modifying therapies in multiple sclerosis patients. *Journal of neurology* **264**, 2325-2329, doi:10.1007/s00415-017-8595-8 (2017).

15 Lanzillo, R. *et al.* A multicentRE observational analysiS of PErsistenCe to Treatment in the new multiple sclerosis era: the RESPECT study. *Journal of neurology* **265**, 1174-1183, doi:10.1007/s00415-018-8831-x (2018).

16 Vermersch, P. *et al.* Teriflunomide versus subcutaneous interferon beta-1a in patients with relapsing multiple sclerosis: a randomised, controlled phase 3 trial. *Multiple sclerosis (Houndmills, Basingstoke, England)* **20**, 705-716, doi:10.1177/1352458513507821 (2014).

17 Balak, D. M. *et al.* Prevalence of cutaneous adverse events associated with long-term disease-modifying therapy and their impact on health-related quality of life in patients with multiple sclerosis: a cross-sectional study. *BMC neurology* **13**, 146, doi:10.1186/1471-2377-13-146 (2013).

18 Rommer, P. S. & Zettl, U. K. Managing the side effects of multiple sclerosis therapy: pharmacotherapy options for patients. *Expert opinion on pharmacotherapy* **19**, 483-498, doi:10.1080/14656566.2018.1446944 (2018).

19 Lee Mortensen, G. & Rasmussen, P. V. The impact of quality of life on treatment preferences in multiple sclerosis patients. *Patient Prefer Adherence* **11**, 1789-1796, doi:10.2147/ppa.S142373 (2017).

20 La Mantia, L., Munari, L. M. & Lovati, R. Glatiramer acetate for multiple sclerosis. *Cochrane Database Syst Rev*, Cd004678, doi:10.1002/14651858.CD004678.pub2 (2010).

21 Gold, R. *et al.* Placebo-controlled phase 3 study of oral BG-12 for relapsing multiple sclerosis. *N Engl J Med* **367**, 1098-1107, doi:10.1056/NEJMoa1114287 (2012).

22 Fox, R. J. *et al.* Placebo-controlled phase 3 study of oral BG-12 or glatiramer in multiple sclerosis. *N Engl J Med* **367**, 1087-1097, doi:10.1056/NEJMoa1206328 (2012).

23 Sejbaek, T., Nybo, M., Petersen, T. & Illes, Z. Real-life persistence and tolerability with dimethyl fumarate. *Mult Scler Relat Disord* **24**, 42-46, doi:10.1016/j.msard.2018.05.007 (2018).

24 CIBG; ministerie van Volksgezondheid, W. e. S. *Prijsvorming*, <www.farmatec.nl> (z.d.).

25 Kister, I. *et al.* Discontinuing disease-modifying therapy in MS after a prolonged relapse-free period: a propensity score-matched study. *J Neurol Neurosurg Psychiatry* **87**, 1133-1137, doi:10.1136/jnnp-2016-313760 (2016).

26 Bonenfant, J. *et al.* Can we stop immunomodulatory treatments in secondary progressive multiple sclerosis? *Eur J Neurol* **24**, 237-244, doi:10.1111/ene.13181 (2017).

27 Bsteh, G. *et al.* Discontinuation of disease-modifying therapies in multiple sclerosis - Clinical outcome and prognostic factors. *Mult Scler* **23**, 1241-1248, doi:10.1177/1352458516675751 (2017).

28 O'Rourke, K. E. & Hutchinson, M. Stopping beta-interferon therapy in multiple sclerosis: an analysis of stopping patterns. *Mult Scler* **11**, 46-50, doi:10.1191/1352458505ms1131oa (2005).

29 Rio, J. *et al.* Factors related with treatment adherence to interferon beta and glatiramer acetate therapy in multiple sclerosis. *Mult Scler* **11**, 306-309, doi:10.1191/1352458505ms1173oa (2005).

30 CBO richtlijn (2012) Immunomodulerende en immunosuppressieve behandeling bij multipele sclerose. (2012).

31 Kennisagenda neurologie 2017: https://gallery.mailchimp.com/29087cdad5c58a12bd346e83f/files/3b5692f1-3840-48e2-99e7-405edb9a895f/Kennisagenda_Neurologie_eindversie_16_12_2017.pdf.

32 Kister, I. & Corboy, J. R. Reducing costs while enhancing quality of care in MS. *Neurology* **87**, 1617-1622, doi:10.1212/WNL.0000000000003113 (2016).

33 Uher, T. *et al.* Is no evidence of disease activity an achievable goal in MS patients on intramuscular interferon beta-1a treatment over long-term follow-up? *Mult Scler* **23**, 242-252, doi:10.1177/1352458516650525 (2017).

34 Rotstein, D. L., Healy, B. C., Malik, M. T., Chitnis, T. & Weiner, H. L. Evaluation of no evidence of disease activity in a 7-year longitudinal multiple sclerosis cohort. *JAMA Neurol* **72**, 152-158, doi:10.1001/jamaneurol.2014.3537 (2015).

35 De Stefano, N. *et al.* Long-term assessment of no evidence of disease activity in relapsing-remitting MS. *Neurology* **85**, 1722-1723, doi:10.1212/WNL.0000000000002105 (2015).

36 Gray, O., McDonnell, G. & Hawkins, S. Tried and tested: the psychometric properties of the multiple sclerosis impact scale (MSIS-29) in a population-based study. *Mult Scler* **15**, 75-80, doi:10.1177/1352458508096872 (2009).

37 Pfennings, L. E. *et al.* A health-related quality of life questionnaire for multiple sclerosis patients. *Acta Neurol Scand* **100**, 148-155 (1999).

38 Rietberg, M. B., Van Wegen, E. E. & Kwakkel, G. Measuring fatigue in patients with multiple sclerosis: reproducibility, responsiveness and concurrent validity of three Dutch self-report questionnaires. *Disabil Rehabil* **32**, 1870-1876, doi:10.3109/09638281003734458 (2010).

39 Eagle, T. *et al.* Treatment satisfaction across injectable, infusion, and oral disease-modifying therapies for multiple sclerosis. *Mult Scler Relat Disord* **18**, 196-201, doi:10.1016/j.msard.2017.10.002 (2017).

40 Kobelt, G., Berg, J., Lindgren, P. & Jonsson, B. Costs and quality of life in multiple sclerosis in Europe: method of assessment and analysis. *Eur J Health Econ* **7 Suppl 2**, S5-13, doi:10.1007/s10198-006-0365-y (2006).

41 Janssen, M. F. *et al.* Measurement properties of the EQ-5D-5L compared to the EQ-5D-3L across eight patient groups: a multi-country study. *Qual Life Res* **22**, 1717-1727, doi:10.1007/s11136-012-0322-4 (2013).

42 Bouwmans, C. *et al.* The iMTA Productivity Cost Questionnaire: A Standardized Instrument for Measuring and Valuing Health-Related Productivity Losses. *Value Health* **18**, 753-758, doi:10.1016/j.jval.2015.05.009 (2015).

RESEARCH PROTOCOL

The safety and cost-effectiveness of discontinuing disease-modifying therapies in stable relapsing-onset multiple sclerosis (DOT-MS): a randomized rater-blinded multicenter trial.

Version 8, March 2023

TABLE OF CONTENTS

1. INTRODUCTION AND RATIONALE 12
2. OBJECTIVES 16
3. STUDY DESIGN 19
4. STUDY POPULATION 20
4.1 Population (base) 20
4.2 Inclusion criteria 21
4.3 Exclusion criteria 21
4.4 Sample size calculation 22
5. TREATMENT OF SUBJECTS 23
5.1 Investigational product/treatment 23
5.2 Use of co-intervention 23
5.3 Escape medication 23
6. INVESTIGATIONAL PRODUCT 23
7. NON-INVESTIGATIONAL PRODUCT 23
8. METHODS 23
8.1 Study parameters/endpoints 23
8.1.1 Main study parameter/endpoint 23
8.1.2 Secondary study parameters/endpoints (if applicable) 24
8.1.3 Other study parameters (if applicable) 25
8.2 Randomisation, blinding and treatment allocation 25
8.3 Study procedures 26
8.4 Withdrawal of individual subjects 30
8.4.1 Specific criteria for withdrawal (if applicable) 30
8.5 Replacement of individual subjects after withdrawal 30
8.6 Follow-up of subjects withdrawn from treatment 30
8.7 Premature termination of the study 30
9. SAFETY REPORTING 32
9.1 Temporary halt for reasons of subject safety 32
9.2 AEs, SAEs and SUSARs 32
9.2.1 Adverse events (AEs) 32
9.2.2 Serious adverse events (SAEs) 32
9.2.3 Suspected unexpected serious adverse reactions (SUSARs) 33
9.3 Annual safety report 33
9.4 Follow-up of adverse events 33
9.5 Data Safety Monitoring Board (DSMB) 33
10. STATISTICAL ANALYSIS 35
10.1 Primary study parameter(s) 35
10.2 Secondary study parameter(s) 36
10.3 Other study parameters 36
10.4 Interim analysis 36
11. ETHICAL CONSIDERATIONS 40

11.1 Regulation statement 40
11.2 Recruitment and consent..40
11.3 Objection by minors or incapacitated subjects (if applicable)..40
11.4 Benefits and risks assessment, group relatedness..40
11.5 Compensation for injury 41
11.6 Incentives (if applicable)..41
12. ADMINISTRATIVE ASPECTS, MONITORING AND PUBLICATION 42
12.1 Handling and storage of data and documents 42
12.2 Monitoring and Quality Assurance..43
12.3 Amendments..45
12.4 Annual progress report..45
12.5 Temporary halt and (prematurely) end of study report..45
12.6 Public disclosure and publication policy..45
13. STRUCTURED RISK ANALYSIS..47
13.1 Potential issues of concern..47
13.2 Synthesis 47
14. REFERENCES 48

LIST OF ABBREVIATIONS AND RELEVANT DEFINITIONS

9HPT	9-Hole Peg Test
ABR	General Assessment and Registration form (ABR form), the application form that is required for submission to the accredited Ethics Committee; in Dutch: Algemeen Beoordelings- en Registratieformulier (ABR-formulier)
AE	Adverse Event
AR	Adverse Reaction
CA	Competent Authority
CCMO	Central Committee on Research Involving Human Subjects; in Dutch: Centrale Commissie Mensgebonden Onderzoek
CIS	Clinically Isolated Syndrome
CIS20r	Checklist Individual Strength
CV	Curriculum Vitae
DMT	Disease Modifying Therapy
DSMB	Data Safety Monitoring Board
EDSS	Expanded Disability Status Scale
EQ-5D-5L	EuroQol 5 Dimensions Questionnaire
EU	European Union
FLAIR	Fluid Attenuation Inversion Recovery
GCP	Good Clinical Practice
GDPR	General Data Protection Regulation; in Dutch: Algemene Verordening Gegevensbescherming (AVG)
IC	Informed Consent
iMCQ	Medical Consumption Questionnaire
iPCQ	Productivity Costs Questionnaire
METC	Medical research ethics committee (MREC); in Dutch: medisch-ethische toetsingscommissie (METC)
MRI	Magnetic Resonance Imaging
MS	Multiple Sclerosis
MSFC	Multiple Sclerosis Functional Composite
MSIS-29	Multiple Sclerosis Impact Scale
RRMS	Relapsing Remitting Multiple Sclerosis
(S)AE	(Serious) Adverse Event
SDMT	Symbol Digits Modalities Test

**Sponsor**	**The sponsor is the party that commissions the organisation or performance of the research, for example a pharmaceutical company, academic hospital, scientific organisation or investigator. A party that provides funding for a study but does not commission it is not regarded as the sponsor, but referred to as a subsidising party.**
**SF-36**	**Short Form Health Survey**
**SPMS**	**Secondary Progressive Multiple Sclerosis**
**SUSAR**	**Suspected Unexpected Serious Adverse Reaction**
**T25fW**	**Timed 25-foot Walk**
**TSQM**	**Treatment Satisfaction Questionnaire for Medication**
**UAVG**	**Dutch Act on Implementation of the General Data Protection Regulation; in Dutch: Uitvoeringswet AVG**
**WMO**	**Medical Research Involving Human Subjects Act; in Dutch: Wet Medisch-wetenschappelijk Onderzoek met Mensen**

SUMMARY

Rationale: The past few years, several new effective drugs have come onto the market for the treatment of relapsing remitting MS (RRMS), all of which have potentially serious side effects. The arrival of these drugs has led to a new aim for treating MS patients: achieving a status of complete clinical and radiological control of inflammatory events, also described as a status of no evident disease activity (NEDA-3). With these adjusted goals, medication is often started at an earlier stage and the disease is treated more aggressively. This leads to better control of the disease, but also to increased exposure to possible (serious) side effects. A considerable group of patients with a fully stable-disease under treatment merely have a benign or less inflammatory disease course rather than a necessity for treatment to prevent inflammation. This raises the question whether and when patients who have been stable under medication for years can safely discontinue the treatment. The hypothesis of this study is that discontinuing medication after >5 years without evidence of inflammatory disease activity does not result in return of inflammatory disease activity.

Objective: The aim of this study is to identify whether it is possible to safely discontinue treatment in MS patients who have shown no evidence of active inflammation in the years prior to inclusion clinically and/or radiologically. The secondary objectives address the questions whether the discontinuation of first-line treatment has an effect on disability progression and whether the discontinuation of first-line treatment improves the quality of life for the patient and if this can be measured in a daily setting using digital biomarkers. Furthermore, blood collections will be included to assess whether it is possible to retrospectively predict possible return of inflammatory activity with biomarkers such as neurofilament light (NFL) or patient characteristics such as disease activity prior to disease modifying therapy (DMT). In case of emerging disease activity after the cessation of therapy we will assess if reinitiation will lead to NEDA again, and if there are long-term consequences. If possible, post-hoc analysis are performed for the different types of treatment compounds.

Study design: Multi-center observational extension of a randomized and controlled, rater-blinded trial in the Netherlands. Currently, 89 patients with relapse onset MS were included in the DOT-MS trial and were randomized to either discontinue the previously used DMT or to continue their DMT. From March 2023, the study is observational and patients were no longer randomized. Patients that discontinued their DMT were advised to reinitiate their DMT in consultation with their neurologist. All patients currently included in the DOT-MS trial will be followed up.

Study population: MS patients who are treated with one of the first-line treatments (any of the interferons, glatiramer acetate, dimethylfumarate, teriflunomide) and who had a complete

absence of inflammatory activity (no relapses, no new-T2 lesions and no contrast-enhancing lesions) for 5 consecutive years under first-line treatment.

Intervention (if applicable): Discontinuation of the previously used DMT till March 2023. From this date, all patients were no longer randomized and patients that discontinued their DMT were advised to reinitiate their DMT. All patients currently included in the DOT-MS trial will be followed up.

Main study parameters/endpoints: The primary endpoint is the return of inflammatory disease activity after 2 years: either relapses, new or enlarging lesions on T2-weighted MRI and gadolinium-enhancing lesions on post-contrast T1-weighted MRI. Secondary end-points are EDSS and MSFC progression (combined: EDSS plus), number of relapses, individual MRI-parameters (such as lesion numbers), quality of life measurements, optical coherence tomography (OCT) and eye movement measurements, and (digital) biomarker measurements.

Nature and extent of the burden and risks associated with participation, benefit and group relatedness: The burden of participation consists of assessments during visits at baseline, 3, 6, 12, 18 and 24 months. Follow-up visit will consist of clinical and radiological measurements, quality of life questionnaires and blood collection (**Figure 1**). Additional data will be collected via mobile applications MS sherpa and Neurokeys, that will be installed on patients' smartphones. For this, patients will be asked to perform tasks on their phones, measuring walking ability, hand function, cognition and fatigue. This takes approximately five minutes every two weeks and is performed at home. When disease activity emerges, patients are treated according to the current standard of care.

Groups:<br>Discontinuation group with restart X<br>Continuation group O<br>Discontinuation group (<6 months) without restart Z<br>Discontinuation group (>6 months) without restart Y	Visit 1 (month 0)	Visit 2 (month 3)	Visit 3 (month 6)	Visit 4 (month 12)	Visit 5 (month 18)	Visit 6 (month 24)
Interview with researcher	X O Z Y	X Z	X O Z Y	X O Z Y	X O Z Y	X O Z Y
EDSS	X O Z Y	X Z	X O Z Y	X O Z Y	X O Z Y	X O Z Y
MSFC	X O Z Y	X Z	X O Z Y	X O Z Y	X O Z Y	X O Z Y
Blood collection	X O Z Y	X Z	X O Z Y	X O Z Y	X O Z Y	X O Z Y
Questionnaires	X O Z Y	X Z	X O Z Y	X O Z Y	X O Z Y	X O Z Y
OCT-scan	X O Z Y			X O Z Y		X O Z Y
Eye movement measurements	X O Z Y			X O Z Y		X O Z Y
MRI-scan	X O Z Y	X Z	X Z Y	X O Z Y	Z Y	X O Z Y
MS sherpa app (optional)	Every two weeks					
Neurokeys app (optional)	Continuous					

Figure 1. Visit moments, including the measurements, for different groups. First group consist of patients that were randomized to stop their MS treatment and has restarted their medication. Second group consists of patients that were in the continuation group. Third group consists of patients that were in the discontinuation group for less than 6 months and have decided to remain discontinued of their treatment. Last group consists of patients that were in the discontinuation group for more than 6 months and have decided to remain discontinued of their treatment.

1. INTRODUCTION AND RATIONALE

In recent decades, the therapeutic landscape of multiple sclerosis (MS) has changed dramatically. Coming from an era where virtually no therapies were available, there are currently more than 12 first- and second-line disease modifying treatment (DMT) options for the prevention of focal inflammatory demyelinating lesions in the brain and spinal cord. Clinically MS can be devastating; it affects roughly 1 in 1000 persons in the Netherlands usually diagnosed in the prime of their lives with a mean age at diagnosis around 30[1]. Two major disease phenotypes exist. The most important is relapsing-onset MS (80% MS patients), including patients with a clinically isolated syndrome (CIS), relapsing remitting MS (RRMS) and secondary progressive MS (SPMS). The other 20% suffers from a primary progressive MS (PPMS), a disease phenotype with more distinct neurodegeneration. Untreated, 50% of patients will need assistance walking small distances after 10-20 years after diagnosis[2].The main pathological hallmark in the first stages (CIS and RRMS) is recurrent focal inflammation of the brain and spinal cord leading to demyelination[3]. The first years after the diagnosis patients usually experience 2-3 relapses annually. The severity of neurological disability depends on the localisation of the inflammation. With increasing age, the amount of inflammation tends to diminish and an unknown neurodegenerative pathology drives the disease course. Clinically there is a progressive decline in neurological functioning; i.e. the “progressive” phase (or the secondary progressive (SPMS) disease course).

There has been great change in the timing of diagnosing MS and evaluating disease activity with the introduction of magnetic resonance imaging (MRI). In the early days the disease course was solely evaluated based on new relapses and/or progression of disability. Now, the arrival of MRI has led to a revised aim for treating MS patients: achieving a status of “no evident disease activity” (NEDA-3); complete clinical and radiological control of inflammatory events and no significant increase in disability[4]. To date an estimated 10% of patients have a status of NEDA-3 under long-term first-line therapy, implying a full control of focal inflammation in these patients[5-7]. In recent years there has been a growing trend of starting treatment earlier and to treat more aggressively, partly based on the concept of NEDA. Treatment is almost always initiated directly after diagnosis but sometimes even before a definite diagnosis of RRMS is made[8]. There is however a substantial percentage of patients with a more benign disease course, described in a very broad range of 6-64% of MS patients[9-12]. At the moment of diagnosis, it is not known how the disease course will develop and based on the substantial group of “benign” MS cases, it is likely that a considerable group of patients that have long-term and fully stable-disease under DMT is unnecessarily

treated. In addition, there is the group of patients who experience disease progression despite their therapy. Also, these patients probably do not benefit from their therapy.

Exposure to treatment is not without risks and costs. Side-effects of MS medication are frequently present. Data on the proportion of patients discontinuing first-line DMT's demonstrates a discontinuation rate of 20-40% during an observation period over 1 year, with the occurrence of side effects and poor tolerability as the most common reason for drug withdrawal[13-15]. A large proportion of the patient population is confronted with side effects for both oral (teriflunomide, dimethylfumarate) and injectable (interferons, glatiramer acetate) DMT's. For example, the results of the post-approval clinical trials on the safety of teriflunomide compared to interferon beta-1a showed that 93-96% of the patients experienced side effects[16]. Each injectable first-line DMT can lead to mild cutaneous adverse events such as erythema and swelling, but also to more severe and persisting effects such as lipoatrophy, infections and even necrosis. Patients with a cutaneous reaction appeared to have a lower dermatology-specific health-related quality of life[17]. Serious events rates were also high ranging from 7% (interferon beta-1a) to 12% (teriflunomide)[16].

Each first-line DMT has a different mode of administration and specific side-effects. Interferons are administered biweekly s.c. (Plegridy), weekly i.m. (Avonex), second daily s.c. (Betaferon) or thrice weekly s.c. (Rebif). Flu-like symptoms are the most often reported side effects of interferon ß injections and are particularly challenging for MS patients[18,19]. But also allergic reactions, elevated liver enzymes leading to severe hepatic injury, thyroid autoimmunity, hypothyroidism and hematologic abnormalities might occur[18]. Glatiramer acetate (Copaxone) is injected subcutaneously daily or thrice weekly. Patterned reactions are most commonly reported in patients using glatiramer acetate, consisting of flushing, chest pain, palpitations, urticaria, anxiety and dyspnoea with a relative risk of 3.27. This patterned reaction unpredictably occur within minutes of injection and spontaneously resolve before 30 minutes[20]. Teriflunomide (Aubagio) is an oral drug and is administered daily. Hair thinning, increased blood pressure, fatigue, diarrhoea, sensory disturbances, elevated liver enzymes, and renal failure are adverse effects that have been reported in patients using teriflunomide[18]. The fourth agent in the first-line DMT group, dimethylfumarate (Tecfidera) is taken orally twice daily. Clinical phase 3 trials reported mild or moderate flushing and gastrointestinal (GI) adverse events, 36% and 42%, respectively as most common adverse effects[21-23]. Consequently, this led to treatment discontinuation in both trials. Serious side effects include urosepsis (interferons), hepatotoxicity (glatiramer acetate), but also progressive multifocal leukoencephalopathy (dimethyl fumarate), which are all potentially lethal.

Furthermore, there is a great burden of costs to society. The costs for a year of first-line immunomodulating drugs range from 12.000 – 15.000 euros annually[24]. The discontinuation of therapy in appropriate patients therefore also has a very significant effect with a potential cost-reduction of 2-2.5 million euros annually in The Netherlands. Costs due to side-effects (such as treatment and absence of work) are not even included here.

The data on the discontinuation of therapy in long-term inflammatory stable MS patients is reassuring. One large retrospective cohort study has shown in 1200 patients that stable RRMS patients who stop treatment don't have an increased relapse rate compared to patients who continue treatment[25]. There was a slightly higher disability progression of the discontinuation group, presumably based on the discontinuation of treatment in patients with progressive MS. Also, there seems to be no rebound inflammation after discontinuation of therapy in progressive MS, and a similar relapse rate as the years prior to discontinuation[26]. Lastly, one study has showed that patients 45 years or older, or patients with a DMT intake of 4 or more years without evidence of clinical or radiological disease activity showed a high likelihood of remaining relapse-free after discontinuation and absence of contrast enhancing lesions[27]. All studies where however hampered by either its retrospective nature, or incompleteness on for examples reasons for discontinuation or a sufficiently matched control group. Obviously, definite conclusions can only be drawn after a well-designed controlled trial. Currently, two trials with a similar question to ours are underway (DISCOMS; NCT03073603 and STOP-I-SEP; NCT03653273). These studies are different compared to our proposal in that the inclusion criteria only allow for older patients (older than 55 years and older than 50 years respectively) to be included.

The question of whether or not to discontinue therapy is increasingly present during our outpatient clinics [28,29]. As stated, an estimated 10% of patients have a status of NEDA-3 under long-term first-line DMT and could benefit from the results of this study. To give an indication of the number of patients concerned: in the Netherlands alone an estimated number of 7000-7500 patients with MS use first-line DMT, which means 700-750 patients would be eligible for this study and would benefit from results of this study (numbers are based on market shares and sales of each DMT, data not published). An enquiry amongst Dutch MS neurologists (data not published) shows that every neurologist struggles with the question on average 5-10 times per year. The Dutch 2012 CBO guideline " Multiple Sclerosis" recommends the discontinuation of DMT in patients who experience severe side-effects and who had secondary progressive MS for at least 3 years[30] but this is solely based on expert opinion. Also, internationally there are no guidelines guiding patients and neurologists in this question. Very recently in 2017, the committee of care evaluation of

neurology (‘Zorgevaluatie Neurologie’ (ZEN), part of the ‘Dutch society of Neurologists (Nederlandse Vereniging voor Neurologie; NVN) and supported by the Dutch Federation of medical specialists (FMS) and the Dutch Federation of Patients) has ranked this lack of evidence regarding the discontinuation of immunomodulating drugs in MS one of the most important science/knowledge gaps within daily neurological practice in the Netherlands. It listed this topic in its “ Kennisagenda 2018-2022” which prioritizes the 12 most urgent topics for scientific research in the field of neurology in the coming 4 years to improve the effectiveness and safety of our daily medical practice[31]. We have validated this support by means of a questionnaire amongst all 88 members of the MS Taskforce (Part of the NVN). All (100%) respondents (response-rate was 49%) indicated that they found the issue frequently present in their clinics and important tot research. Also, internationally there is strong call for more evidence-based guidelines and consensus regarding the discontinuation of treatment in long-term stable MS patients[32]. This underlines the national (and international) need for systematic research regarding this subject.

2. OBJECTIVES

With this study we will bring first evidence to this important issue by identifying whether it is possible to safely discontinue treatment in MS patients who have shown no evidence of active inflammation in the years prior to inclusion. Importantly, we include quality of life measurements to evaluate if this also translates to an increased perception of health. If present, it will optimize the treatment paradigm for individual MS patients through identifying unnecessary exposure to medication while proving the discontinuation of medication to be safe, and beneficial for the daily lives of individual patients in terms of an increased quality of life. In addition, it greatly benefits the general society since it also provides a more efficient use of funds with the significant cost-reduction it brings. The impact of the trial is immediate, within 5 years, and since all large MS centers will participate there is a direct line into the daily offices of treating neurologists, and into the development of adjusted guidelines regarding the treatment of MS. Most importantly, every possible result deriving from this trial will have a significant impact on (inter)national treatment strategies. Besides the primary question of the evaluation of safety we also incorporate validated measures of quality of life and disease perception to evaluate potential changes in quality of life. Furthermore, we include blood collections for the monitoring of neurofilament light (currently the only validated and clinically applied biomarker for the return of inflammatory activity) that could potentially serve as indicator for subclinical return of inflammatory activity. In addition, we will include optical coherence tomography (OCT) and eye movement measurements for patients participating in Amsterdam UMC. OCT measurements (especially retinal nerve fiber layer (RNFL) thickness) are known to be associated with disability in MS patients, and thus can be seen as a measure for disease progression.

Primary outcome measures used in this study are clinical and radiological outcome measures that are already part of routine clinical practice, which ensures that results of this trial can be directly and easily implemented in standard clinical care. In addition to these outcome measures, it would be of interest to determine optimal measurements for potential return of disease activity. A promising method in this regard is the use of mobile applications on patients' smartphones, because of their non-invasive nature (in contrast to for example MRI-scans) and the potential for more continuous measurements in a real-world setting (i.e. at home). Measurements with mobile applications MS sherpa and Neurokeys will be included for a subgroup of patients (depending on the availability of a smartphone), to investigate if return of inflammatory disease activity can be measured with these applications. These mobile applications will be installed on patients' smartphones.

See also methods section for a full description.

Research questions

Primary research question:

Can we safely discontinue first-line medication in MS patients with long-term absence of inflammation, without the return of *inflammatory disease activity* clinically and radiologically?

Other research questions:

- Does the discontinuation of first-line treatment have an effect on disability progression?
- Does the discontinuation of first-line treatment improve the quality of life for the patient?
- What is the effect of discontinuation of first-line treatment on individual MRI outcome measures such as lesion load and atrophy measurements?
- Is it possible to predict possible return of inflammatory activity with biomarkers such as neurofilament light (NFL) or patient characteristics such as disease activity prior to DMT?
- In case of emerging disease activity after cessation of DMT, will a restart of DMT result in NEDA again and if so, how long does it take?
- In case of emerging disease activity after treatment cessation, are there any differences between the different DMT compounds?
- What is the cost-effectiveness of discontinuation of DMT in The Netherlands?
- Is discontinuation of first-line DMT associated with OCT measurements and eye movement measurements?
- Is it possible to detect and predict (return of) inflammatory disease activity and disease progression with digital biomarkers using mobile applications such as MS sherpa and Neurokeys?

From March 2023 on, patients will no longer be randomized into the intervention (discontinuation of DMT) or control arm. All patients were advised to reinitiate their previously used DMT. Patients will be followed up in the observational extended phase for a period of 2 years It is of scientific significance to conduct long-term follow-up studies on participants who have discontinued medication, as well as those who have resumed medication, in order to examine the potential long-term consequences of these actions. At present, it remains uncertain whether participants who were assigned to the cessation group and subsequently experienced disease activity will exhibit sustained stability in the future. Similarly, this question applies to the group that discontinued medication but has since resumed. Thus, it

would be highly informative to conduct further follow-up investigations to assess disease development in these participants.

Other objectives from March 2023 on:

- Return of inflammatory disease activity clinically and radiologically in patients that continued DMT and in patients that discontinued their previously used DMT (and may have restarted DMT).
- Other outcomes include aforementioned measurements such as disability progression, quality of life, MRI outcome measures, biomarkers (NFL, mobile applications).

3. STUDY DESIGN

The study design is a multi-center observational extended rater-blinded randomized controlled trial in the Netherlands. The project will take place over period of 2 years (24 months). The lead and monitoring of the trial will be performed by the MS center Amsterdam (MSCA). Participating centers are listed in an appendix (I1).

Study-population: A total of 89 relapse onset MS patients who are treated with one of the first-line treatments (any of the interferons, glatiramer acetate, dimethylfumarate, teriflunomide) *and* who had a complete absence of objective inflammatory activity (no objectified relapses, no significant number (2 or more) of new-T2 lesions and no contrast-enhancing lesions) for 5 consecutive years under first-line treatment were included in the DOT-MS trial and were randomized either to discontinue or continue their DMT. Patients may not have switched between first-line drugs over the two years prior to inclusion. If a switch has occurred this should not have been due to ineffectivity of the first DMT but due to side-effects or by a personal preference of the patient (such as the wish to switch to oral therapies). In the case of previous use of interferons patients must be negative for neutralizing antibodies (NAbs). In March 2023, all included patients were advised to reinitiate their previously used DMT. Continuation of participation in the DOT-MS trial will take place after informed consent. This will be obtained after careful and extensive information about the possible risks according to local ethical review board requirements.

Intervention: The intervention, the discontinuation of the previously used DMT, is no longer applicable. From March 2023, all included patients were no longer randomized and patients that discontinued their DMT were advised to reinitiate their DMT. All patients currently included in the DOT-MS trial will be followed up.

Follow-up frequency: Patients in the continuation group are expected to remain stable throughout the study period based on their proven stable status for 5 consecutive years prior to enrolment. The minimum evaluation that must take place should have a frequency according to the current standard of care (yearly evaluation of every patient treated with immunomodulating therapy; i.e. baseline (BL), 12 and 24 months and 3 months (rebaseline) when immunomodulating therapy is restarted). Timely recognition of recurrence of any (subclinical) disease activity in the discontinuation group (that may have reinitiated their DMT) is secured by more frequent clinical and radiological assessment during extra routine study visits at 3, 6 and 18 months. Patients that were in the discontinuation group and have restarted their DMT will receive clinical and radiological assessments at 6 and 18 months next to the current standard of care (BL, 3, 12 and 24 months). Moreover, patients that were

in the discontinuation group for less than 6 months and have decided to remain discontinued of their treatment will receive extra clinical and radiological assessments at visit 3, 6 and 18 months. For patients that were in the discontinuation group for more than 6 months and have decided to remain discontinued of their treatment will receive extra clinical and radiological assessments at visit 6 and visit 18 months. Patients that were in the continuation group will receive MRI assessments according to the current standard of care (BL, 12 and 24 months) and extra clinical assessment at 6 months and 18 months for optimal comparability between all groups.

4. STUDY POPULATION

4.1 Population (base)

We included 89 participants with the diagnosis relapse onset MS with a minimum age of 18 years. 44 patients were assigned to the discontinuation group and were advised to reinitiate their therapy, and 45 patients were assigned to the continuation group. The currently included patients will be followed and no new patients will be included.

Currently an estimated number of 7000-7500 patients use first-line DMT for their MS. This number is estimated on data that was provided by all different pharmaceutical companies of each of the first-line therapies. Numbers are based on market shares and actual sales of each of the treatments (data not published).

The percentage of “stability” derives from the treatment effects described in the pivotal phase III trials of the currently available first-line treatments and 3 cohort studies with a longer follow-up that the average 2 years in the phase III trials. For our calculation of possible eligible patients, we have used the most “negative” scenario based on the results of these trials. The most recent phase III studies report on NEDA as outcome measure (Havrdova et al 2017, Arnold et al 2017), reflecting the new treatment concept of MS: complete stabilization of the disease process. In those cohorts NEDA ranges from 25-40% (Havrdova et al 2017, Arnold et al 2014 & 2017, Miller et al 2014, Nygaard et al 2015). It is important to note however, that the concept of NEDA also includes progressive neurological decline (such as is due to progressive disease and neurodegeneration). The percentage of patients that is free from inflammation is therefore somewhat underestimated in the numbers of these studies. Furthermore, most studies comprise on average a relatively short observation period of 2 years.

Three cohort studies exist with a longer follow-up. De Stefano et al, Uher et al and Rotstein et al published cohort studies that describe a longitudinal follow-up of NEDA status of ~ 200 patients for 7-10 years[33-35]. They showed that a fairly low percentage of patients remained NEDA after 5 years (10-15%). In the cases of emerging inflammatory activity under treatment, most patients showed disease activity in the first 1-2 years after the start of treatment. Only a small minority of patients lose their NEDA status after 5 years (4%). It is very important to note that in the Rotstein-study the loss of NEDA status was in a large proportion due to disease progression rather than new inflammation. This causes a relative underestimation of patients who remain inflammatory stable. Taken together and based on this data, we assume that in the of all first-line DMT users 10% show no signs of inflammation for 5 years, and only a very small percentage of patients shows additional disease activity while staying on treatment (5%). The majority (95%) of patients remain inflammatory stable while continuing their medication.

4.2 Inclusion criteria

In order to be eligible to participate in this study, a subject must meet all of the following criteria:

1. A minimum age of 18 years
2. Ability to understand the purpose and risks of the study and provide signed and dated informed consent and authorization to use protected health information (PHI) in accordance with national and local privacy regulations.
3. Definite diagnosis of relapsing-onset MS according to the revised McDonald 2017 criteria
4. All relapsing-onset MS patients treated with one of the first-line treatments: any of the interferons, glatiramer acetate, dimethylfumarate, teriflunomide
5. Complete absence of inflammatory activity (no objectively defined and confirmed relapses, no significant number (2 or more) of new-T2 lesions suggestive of demyelination and no contrast-enhancing lesions) suggestive of demyelination for 5 consecutive years under first-line treatment at start of inclusion. In case the last available MRI-scan was conducted 10 or more years ago, no more than 3 new T2-lesions suggestive of demyelination in the last 10 years are accepted.

4.3 Exclusion criteria

A potential subject who meets any of the following criteria will be excluded from participation in this study:

1. A switch between first-line disease modifying therapy over two years prior to inclusion, in case the switch has been due to in effectivity of the first DMT. In case the switch has been due to side-effects or by a personal preference of the patient (such as the wish to switch to oral therapies), this is not considered as an exclusion criterium.
2. Women who want to discontinue medication because of a pregnancy wish and women who are pregnant or expect to become pregnant during the study period
3. Patients that have previously used interferon-beta and have been tested positive for neutralizing antibodies (NAbs). This is determined by measuring MxA-bioactivity and is a test that is part of routine follow-up in patients that use interferon-beta. The reason for this is that development of NAbs has been shown to affect interferon-beta treatment efficacy.

4.4 Sample size calculation

The stability of patients in the continuation group is estimated to be at least 97,5%. Based on a non-inferiority margin of 7,5%, a preliminary power-calculation based on the non-inferiority principle was performed (PASS v12, one-sided Z-test (unpooled), significance level 0.05) and showed a necessary sample size of 54 per group to achieve 80% power. Taking 20% drop out into consideration, the total sample size needed for this study is 130.

The applications MS Sherpa and Neurokeys will be added to collect digital biomarkers regarding (return of) inflammatory disease activity. Based on previous experience, our estimate is that the current sample-size can provide some conclusive trends on the association between digital biomarkers and (return of) inflammatory disease activity.

Update inclusion status February 2023

In accordance with the DSMB meeting that took place in January 2023, from the 26th of January 2023 onwards no new patients will be included in the study. New inclusions will be paused for six months, and after these six months the inclusion pause will be re-evaluated.

Update inclusion status March 2023

In accordance with the most recent DSMB meeting that took place in March 2023, from the 20th of March 2023 onwards no new patients will be included in the study. Patients in the intervention arm (discontinuation of DMT) were advised to reinitiate their DMT in consultation with their treating neurologist. All included patients in the DOT-MS trial will be followed up.

TREATMENT OF SUBJECTS

4.5 Investigational product/treatment

The intervention, the discontinuation of the previously used DMT (either interferons, glatiramer acetate, dimethylfumarate or teriflunomide) is no longer applicable. Patients that were assigned to the intervention arm were advised to reinitiate their previously used DMT in consultation with their neurologist.

4.6 Use of co-intervention

During the intervention period patients are asked not to participate in any other scientific studies. Patients are allowed to use all types of co-medication, except for immunomodulating drugs such as prescribed for the treatment of multiple sclerosis and/or other auto-immune diseases.

4.7 Escape medication

When disease activity emerges, patients are treated according to the current standard of care (including intravenous methylprednisolone if deemed necessary) and disease modifying treatment will be reinitiated if that was not the case already. Unscheduled visits including an MRI-scan are planned for each patient with any new neurological complaints, as is standard clinical procedure.

5. INVESTIGATIONAL PRODUCT

N/A

6. NON-INVESTIGATIONAL PRODUCT

MS Sherpa and Neurokeys are CE-certified medical devices. See for the relevant details appendices “D6 - Aanvullende productgegevens MS sherpa” and “D6 – Aanvullende productgegevens Neurokeys”.

7. METHODS

7.1 Study parameters/endpoints

7.1.1 Main study parameter/endpoint

The primary endpoint is number of patients with return of inflammatory disease activity after 2 years based on: a clinically confirmed relapse (defined according to the definition most often used in MS phase-III trials: the onset of new or recurrent symptoms that last > 24 hours, that are accompanied by new objective abnormalities on a neurological examination and that are not explained by non-MS processes such as fever, infection, severe stress or drug toxicity (Gold et al NEJM 2012)) , or any emerging subclinical disease activity proven to

be due to active disease/new inflammation (defined as 3 or more lesions on T2—weighted images or 2 or more gadolinium enhancing lesions on T1-weighted post-contrast MRI suggestive of demyelination) in the discontinuation group.

7.1.2 Secondary study parameters/endpoints (if applicable)

Secondary end-points are

- Changes in neurological functioning
  - EDSS change (Including individual functional systems)
  - MSFC changes
    - Timed 25-foot Walk (T25fW)
    - 9-Hole Peg Test (9HPT)
    - Symbol digits modalities test (SDMT)
- Individual MRI-parameters
  - T1 post-contrast lesion numbers and volumes
  - T2 lesion numbers and volumes
  - Whole brain volume
  - Normalized white matter volume
  - Grey matter volume
- Changes in quality of life measurements
  - Multiple Sclerosis Impact Scale (MSIS-29)
  - Short Form health survey (SF-36)
  - Multiple Sclerosis Self-Efficacy scale (MSSE)
  - Checklist Individual Strength (CIS20r)
  - Treatment Satisfaction Questionnaire for Medication (TSQM)
- Cost measurements
  - EuroQol 5 dimensions questionnaire (EQ-5D-5L)
  - Medical consumption questionnaire (iMCQ)
  - Productivity costs questionnaire (iPCQ)
- Changes in biomarker measurements
  - Neurofilament levels
- OCT and eye movement measurements
  - Peri-papillary retinal nerve fiber (RNFL) thickness
  - Macular ganglion cell-layer inner plexiform layer (GCL-IPL) thickness
  - Eye movement measurements

- Changes in digital biomarkers using the NeuroKeys (CE) and MS sherpa (CE) mobile applications that measure:

- Walking test (2-minute walking test)
- Cognition test (similar to SDMT)
- MS sherpa questionnaires (including fatigue)
- Keystroke data

7.1.3 Other study parameters (if applicable)

Vitamin D, smoking high body-mass index (BMI) are considered as potential confounders. Therefore, vitamin D will be determined and patients will be asked about smoking behaviour during every visit. To avoid any potential bias, we will also collect data (if present in the individual patients) on disease activity prior to the initiation of DMT, such date of diagnosis, time from first symptoms to diagnosis, EDSS scores/MS severity at the moment of DMT initiation.

7.2 Randomisation, blinding and treatment allocation

Since MS affects 2-2.5x as many women as men, we expect to include more women than men. A randomization algorithm will be included to match both groups for sex and age to exclude potential bias.

Outcome measurements will be performed by assessors who are blind to the intervention assignment. For reasons of consistency and to exclude possible bias, all scans will be centrally reviewed in VUmc by a radiologist blinded to allocation to the intervention group. In the current set-up of the trial patients and their treating neurologists are unblinded to the randomization group. The currently available first-line disease modifying treatments consist of the various (peg)interferons, glatiramer acetate, teriflunomide and dimethylfumarate. It comes in a total of more 10 different forms with different packaging, different modes of injection (some subcutaneously, some intramuscular and some oral) and different frequencies of intake (ranging from twice daily to once every two weeks). We have set up the trial so that it is rater-blinded; all MRI-scan and clinical evaluations (such as the neurological examination) will be performed blinded to “treatment” allocation. The primary and majority of secondary outcome measures are derived from these blinded assessments. This approach is frequently chosen in MS research (even in the phase-III trials) for 2 very important reasons. Due to the very distinct nature of side-effects (flushing, gastro-intestinal problems, flu-like symptoms), patients know when they receive placebo instead of the actual active compound. The invasive nature of receiving placebo-injections would greatly enhance the possibility of patients not participating. In addition, we believe it is too costly to create a placebo-control for each of the 10 different forms of medication.

From March 2023 on, this study is an observational extended randomized rater-blinded multicenter trial and therefore has no randomisation, blinding or treatment allocation.

7.3 Study procedures

No study procedures will take place before having obtained informed consent which will be gained following current METc/CCMO standards.

Follow-up frequency

Patients in the continuation group are expected to remain stable throughout the study period based on their proven stable status for 5 consecutive years prior to enrolment. The minimum evaluation that must take place should have a frequency according to the current standard of care (yearly evaluation of every patient treated with immunomodulating therapy; i.e. baseline (BL), 12 and 24 months and 3 months when immunomodulating therapy is restarted). Timely recognition of recurrence of any (subclinical) disease activity in the discontinuation group (that may have reinitiated their DMT) is secured by more frequent clinical and radiological assessment during extra routine study visits at 3, 6 and 18 months. Patients that were in the discontinuation group and have restarted their DMT will receive clinical and radiological assessments at 6 and 18 months next to the current standard of care (BL, 3, 12 and 24 months). Moreover, patients that were in the discontinuation group for less than 6 months and have decided to remain discontinued of their treatment will receive extra clinical and radiological assessments at visit 3, 6 and 18 months. For patients that were in the discontinuation group for more than 6 months and have decided to remain discontinued of their treatment will receive extra clinical and radiological assessments at visit 6 and visit 18 months. Patients that were in the continuation group will receive MRI assessments according to the current standard of care (BL, 12 and 24 months) and extra clinical assessment at 6 months and 18 months for optimal comparability between all groups. For the patients in both groups mobile applications MS sherpa and Neurokeys will be installed. With the MS sherpa application, patients will be asked to perform tests every two weeks during the 24 months of follow-up. The Neurokeys application will collect data on the background of the standard use of mobile phones during this time period.

Clinical evaluation

Duration: 1 hour

Clinical evaluation will consist of a careful medical history: current and past medication, adverse events, number of intravenous methylprednisolone treatments, number of relapses, date of last relapse and signs of symptom progression. A relapse is defined according to the

definition most often used in MS phase-III trials: the onset of new or recurrent symptoms that last > 24 hours, that are accompanied by new objective abnormalities on a neurological examination and that are not explained by non-MS processes such as fever, infection, severe stress or drug toxicity[21]. Furthermore, the Expanded Disability Status Scale (EDSS) and Multiple Sclerosis Functional Composite (MSFC)-measurements will be performed, consisting of the Timed 25-foot Walk (T25fW), 9-Hole Peg Test (9HPT) and the symbol digits modalities test (SDMT).

Radiological evaluation

Duration: 45 min

Radiological evaluation will consist of repeated brain MRI investigations that consist of conventional pre- and post-contrast (T2-weighted, T1-weighted pre and post contrast, FLAIR) images. All scan protocols are available in general and academic hospitals since they form the basis on which MS is diagnosed and treatment is monitored. Although it is expected that new inflammatory lesions can be captured by repeated T2-weighted/FLAIR MRI-scans, a gadolinium-scan is included to not miss any contrast enhancement in previously present lesions. A 3DT1 and 3DFLAIR image will also be made for atrophy measurements.

Questionnaires

Duration: 1 hour

For the evaluation of disease burden and MS related symptoms, we will use the validated and in clinical studies often used Multiple Sclerosis Impact Scale (MSIS-29)[36] Short Form health survey (SF-36)[37] and CIS20r[38]. The perceived impact of treatment, convenience, satisfaction and side-effects will be monitored using the Treatment Satisfaction Questionnaire for Medication (TSQM)[39]. For the evaluation of health related quality of life (HRQoL) and the link between symptoms HRQoL and costs, we use the EDSS for the objective measurement of changes in neurological functioning (which has a clear relation to HRQoL assessed as utility and costs[40]) and the number of patients with return of inflammatory activity. We also use the EQ5D-5L) for cost-utility analysis[41]. Furthermore, we will ask patients to keep a diary describing changes in healthcare consumption (which will be defined per item in a questionnaire (such as hospitalisations, consultations with doctors, use of care at home, use of specialized transportation etc). Lastly patients will be asked for their employment situation and short-term and long-term sick absence because of MS (or MS related treatment) using the iMCQ and iPCQ questionnaires[42]. Questionnaires are filled in digitally either at home or during the hospital visit. Help of a carer or the study-nurse is allowed in cases of the inability using a computer.

Samples

Duration: 15 minutes

Blood collection will take place in both patient groups at every visit. It will consist blood collection for the purpose of biobanking and for diagnostics in the case of any –unforeseen-clinical events. Furthermore, we will retrospectively measure neurofilament light in serum using Simoa.

All participants will visit the hospital for 5 to 6 times over a time frame of 2 years. Each of these visits that take place will take approximately 2,5-3 hours (clinical assessment and MRI-scan). Also 1-hour questionnaires which can be completed at home digitally or at the hospital on paper will take place before/during each visit.

All samples will be collected, processed and stored according to the Standard Operating Procedures (SOP's) as described in the Parelsnoer Biobankprotocol version 8.0. (15) Samples will be stored at the Biobank VUmc. To ensure patient privacy all samples will be coded. A peripheral blood sample will be collected, in total 8 tubes of blood will be drawn (5x EDTA 4 ml for plasma, cells and DNA isolation, 2x serum 5 ml, 1x PAXgene tube 2 ml), adding up to a total volume of 32 ml. Blood will be centrifuged, divided in aliquots of 0.5 ml and then stored at -80 °C.

Optical coherence tomography (OCT)

Duration: 15 minutes

Frequency: yearly (at baseline, month 12 and month 24)

All OCT measurements will be performed by a certified person (not necessarily a physician) on a spectral-domain OCT machine (Spectralis by Heidelberg engineering). Scans to be performed in both eyes:

- Circular scan, centered on optic nerve head (ONH)
- Volume scan, centered on macula.
- Volume scan, centered on optic nerve head (ONH)

Eye movement examination

Frequency: yearly (at baseline, month 12 and month 24)

Duration: 15 minutes

Eye movement measurements will be performed using Eyelink 1000 Plus Eye Tracker (SR Research). Built-in algorithms provided by the eye tracker are used for calibration and validation procedures. The experiment consists of the following assessments:

1. Fixation
2. Pro-saccades

3. Anti-saccades

Mobile applications measurements
Duration: 5 minutes
Frequency: once every two weeks.

For eligible patients (based on regular smartphone use), mobile applications MS-Sherpa and Neurokeys will be installed on their smartphones and data will be collected via these applications.

<u>MS sherpa mobile application</u>
Via the MS sherpa application the patient will be asked to perform walking tests, cognition tests and the MS sherpa questionnaire (including fatigue).

- Cognitive task: similar to the SDMT, the participant is requested to assign numbers to corresponding symbols according to a specific displayed coding.
- Motor task: 2-minute walk test (2MWT): the participant is requested to walk (either unassisted or with a walking aid) for two minutes. The walking distance is measured through the location data. Patients who are unable to walk for two minutes will not be requested to do the 2MWT.
- MS sherpa questionnaire: patient reported outcomes on Likert scales (amongst others about fatigue and the impact of MS on daily activities).

These tests can be completed in approximately 5 minutes and patients will be asked to complete these tests once every two weeks.

<u>NeuroKeys mobile application</u>
NeuroKeys replaces the standard keyboard of the patient's smartphone. Data is collected from regular use of the keyboard, and no additional action from patients is needed. After 24 hours of inactivity of the keyboard of NeuroKeys, either intentionally or unintentionally, a push notification is automatically send utilizing Amazon Simple Notification Service (SNS). NeuroKeys will collect keystroke data general profile information (gender, year of birth, and MS type), and metadata (iOS/Android version and NeuroKeys version). The data will be collected from the phone continuously in a retrospective fashion, when the keyboard is activated the data from the previous keyboard typing session is sent.

- Keystroke data: the start of a message is defined as the keyboard flipping up and the end of a message is marked when the keyboard flips down. Specific keys will be logged and timestamped to be able to accurately calculate parameters expected to be associated to fatigue. The keys logged are: delete or backspace key, dot key, space bar, semicolon, colon, parentheses, capitalized characters, numbers and punctuation marks denouncing the ending of a sentence. All numbers and all punctuation marks will be logged as the same number or punctuation event (e.g. we do not make a distinction between the number 3 and 8 or the comma and dollar sign). Parameters such as word count, amount of words comprised of six characters or more and latency between key presses will be calculated on the basis of the logged keys. In addition to ASCII keys, the unicodes of emojis will also be logged.
- Sensor data: Data from the location sensor, ambient light sensor, gyroscope, motion and accelerometers of the smartphone will be collected when the keyboard is in use. With the location sensor (longitude and latitude), the keystroke data can be combined to open source databases (e.g. weather data from the Royal Netherlands Meteorological Institute, KNMI) to examine the influence of external factors (Davis et al. 2010). Existing and future open source databases provided by public or governmental institutes can be accessed for this purpose. Ambient light sensor can detect environmental brightness which could impact typing behaviour. Kinematic sensors (gyroscope, motion and accelerometers) may be used to infer posture of the user (Lamonaca et al. 2015).

7.4 Withdrawal of individual subjects

Subjects can leave the study at any time for any reason if they wish to do so without any consequences. The investigator can decide to withdraw a subject from the study for urgent medical reasons.

7.4.1 Specific criteria for withdrawal (if applicable)

There are no specific criteria for withdrawal from the study.

7.5 Replacement of individual subjects after withdrawal

There will be no replacement of individual subjects after withdrawal.

7.6 Follow-up of subjects withdrawn from treatment

If a patient is withdrawn from the study, we will still perform follow-up measurements in case the patient is willing and able to cooperate.

7.7 Premature termination of the study

The procedure in case of premature termination of the study is described in section 10.4 and 12.5.

8. SAFETY REPORTING

8.1 Temporary halt for reasons of subject safety

In accordance to section 10, subsection 4, of the WMO, the sponsor will suspend the study if there is sufficient ground that continuation of the study will jeopardise subject health or safety. The sponsor will notify the accredited METC without undue delay of a temporary halt including the reason for such an action. The study will be suspended pending a further positive decision by the accredited METC. The investigator will take care that all subjects are kept informed.

More information about temporary halt for reasons of subject safety is provided in section 10.4 and 12.5.

8.2 AEs, SAEs and SUSARs

8.2.1 Adverse events (AEs)

Adverse events are defined as any undesirable experience occurring to a subject during the study, whether or not considered related to the experimental intervention. Adverse events that are reported spontaneously by the subject or observed by the investigator or his staff and that are relevant to the study will be recorded. Relevant adverse events are possible side-effects of the DMT used and the occurrence of relapses.

Adverse Device Effect (ADE)

An ADE is an adverse event related to the use of an investigational medical device. This includes any adverse event resulting from insufficiencies or inadequacies in the instructions of use, the deployment, the installation, the operation, or any malfunction of the investigational medical device. This also includes any event that is a result of a use error or intentional misuse.

8.2.2 Serious adverse events (SAEs)

A serious adverse event is any untoward medical occurrence or effect that

- results in death;
- is life threatening (at the time of the event);
- requires hospitalisation or prolongation of existing inpatients' hospitalisation;
- results in persistent or significant disability or incapacity;
- is a congenital anomaly or birth defect; or
- any other important medical event that did not result in any of the outcomes listed above due to medical or surgical intervention but could have been based upon appropriate judgement by the investigator.

A SADE is an ADE that has resulted in any of the consequences characteristic of a serious adverse event.

The investigator will report all SAEs and SADEs to the sponsor without undue delay after obtaining knowledge of the *events*. The sponsor will report the SAEs through the web portal *ToetsingOnline* to the accredited METC that approved the protocol, within 7 days of first knowledge for SAEs that result in death or are life threatening followed by a period of maximum of 8 days to complete the initial preliminary report. All other SAEs will be reported within a period of maximum 15 days after the sponsor has first knowledge of the serious adverse events.

8.2.3 Suspected unexpected serious adverse reactions (SUSARs)

This section is not applicable since this study does not investigational medicinal products.

8.3 Annual safety report

This section is not applicable since this study does not investigational medicinal products.

8.4 Follow-up of adverse events

All AEs will be followed until they have abated, or until a stable situation has been reached. Depending on the event, follow up may require additional tests or medical procedures as indicated, and/or referral to the general physician or a medical specialist.
SAEs need to be reported until the end of study, as defined in the protocol in section 9.2.2.

8.5 Data Safety Monitoring Board (DSMB)

For optimal safety we will appoint an independent data safety monitoring board (DSMB) which will monitor trial data on a regular basis. The aim of the committee is to safeguard the interests of trial participants and assess the safety of the discontinuation of therapy during the trial. The specific role of the DSMB consists of monitoring evidence for harm due to the intervention (discontinuing medication). The DSMB may advice to terminate the trial prematurely if disease activity exceeds above mentioned thresholds (see section 10.4 for interim analyses).

The DSMB consists of 4 members who do not have conflict of interest with the sponsor or the study. In addition to the permanent members of the DSMB two external members are added to the DSMB with expertise in the relevant field of research (yet to be determined).

The DSMB will conduct interim analyses in a predetermined manner and at predetermined times (see section 10.4), to see whether the relationship between clinical benefit and burden remains acceptable to the subject during the study. After each interim analysis, the DSMB reports to the study coordinator, with reports to the METc and the study sponsor, i.e. the Board of Directors (Raad van Bestuur). The DSMB can give advice to continue, change or stop (parts of) the study. The DSMB will also ensure the quality and safety of research in the participating centers.

From March 2023 on, patients will no longer be randomized into the intervention (discontinuation of DMT) or control arm. This study design will be observational and the aforementioned interim analyses from DSMB will no longer hold.

9. STATISTICAL ANALYSIS

All data is quantitative and will be presented in tables and graphs. Baseline data will be collected to detect any potential differences between the two investigated groups for which we have not corrected with the randomization procedure (sex and age). Possible other confounders include smoking habits, vitamin D levels but also previous disease course in terms of differences in disease duration, number of relapses prior to stability, years of use of treatment.

The primary endpoint is the number of patients with return of inflammatory disease activity after 2 years based on: a clinically confirmed relapse or any emerging subclinical disease activity proven to be due to active disease/new inflammation (defined as 3 or more lesions on T2—weighted images or 2 or more gadolinium enhancing lesions on T1-weighted post-contrast MRI) in the discontinuation group. Secondary end-points are: 1. Changes in neurological functioning (EDSS/MSFC changes including individual functional systems and MSFC subtests), 2. Individual MRI parameters (T2 and T1 post-contrast lesion numbers), 3. changes in quality of life measurements (SF-36, CIS20r, TSQM, EQ5D-5L, iMCQ and iPCQ) and 4. changes in biomarker measurements (neurofilament levels).

9.1 Primary study parameter(s)

For the primary endpoint, the return of inflammatory disease activity after 2 years, a2x2 contingency table will be used to estimate the risk difference for the return of inflammatory disease activity after 2 years (yes/no) between the two groups. The risk difference will be calculated for discontinuation relative to continuation. If the lower bound of the corresponding two-sided 90% confidence interval exceeds -7.5% we conclude non-inferiority of discontinuing medication For the primary endpoint both a per protocol analysis and a intention-to-treat analysis will be performed.

The primary endpoint is the number of patients with return of inflammatory disease activity after 2 years based on: a clinically confirmed relapse or any emerging subclinical disease activity proven to be due to active disease/new inflammation (defined as 3 or more lesions on T2—weighted images or 2 or more gadolinium enhancing lesions on T1-weighted post-contrast MRI suggestive of demyelination).

A relapse is defined according to the definition most often used in MS phase-III trials: the onset of new or recurrent symptoms that last > 24 hours, that are accompanied by new

objective abnormalities on a neurological examination and that are not explained by non-MS processes such as fever, infection, severe stress or drug toxicity[21]

Furthermore, a survival analysis (with an intention-to-treat approach) regarding the time to return of inflammatory activity will be included.

9.2 Secondary study parameter(s)

For all the secondary endpoints correlation and regression analysis (either linear of logistic, depending on the type of variable) will be performed correcting for possible confounders. On the app data, classification analyses and machine learning models will be used next to the more conventional analyses.

9.3 Other study parameters

N/A

9.4 Interim analysis

We have built in a safety-strategy (go-no-go strategy) to control for emerging disease activity (and patients safety). Interim analyses will be done after inclusion of the 40th, 70th and 100th patient. In both the continuation and discontinuation group, the proportion of patients that showed return of inflammatory disease activity – defined as an objectified MS relapse or 3 or more lesions on T2-weighted MRI-images or 2 or more gadolinium enhancing lesions on T1-weighted post-contrast MRI suggestive of demyelination – will be counted and compared between the treatment arms.
If there are more patients with return of inflammatory disease activity (according to the above mentioned definition) in the discontinuation group than in the continuation group, <u>and</u> the 95% confidence interval of the difference in the proportion of patients with return of disease activity between both groups does not include 0, we will discuss premature ending of the study with the DSMB.

For optimal safety the DSMB will monitor the decision making on premature termination every 3 months. The DSMB may advice to terminate the trial prematurely if disease activity exceeds above mentioned thresholds. See section 9.5 for a more detailed description of the DSMB.

The procedure in case of premature termination of the study is described in section 12.5.

In accordance with the most recent DSMB meeting that took place in March 2023, from the 20th of March 2023 onwards no new patients will be included in the study. Currently included patients will no longer be randomized and patients that were assigned to discontinue their DMT were advised to reinitiate their DMT in consultation with their neurologist. Patients that were included in the DOT-MS trial will be followed up. As this study is observational from March 2023 and no new patients will be included and randomized, the interim analyses and DSMB meetings every three months are no longer applicable.

9.5 Cost-analysis

Alongside this trial, we will conduct a cost-effectiveness analysis and a cost-utility analysis over a 2-year follow-up. These will be performed in accordance with the recommendations of the Dutch guideline for health economic evaluations. For the cost-effectiveness analysis, the return of inflammatory disease activity (either relapse or new of enlarging lesions) will serve as the effectiveness measure. The cost-utility analysis will focus on quality of life measured with the EQ-5D-5L, which is often used in MS research. Quality-adjusted life-years (QALYs) will be calculated by multiplying the utility scores belonging to a health state by the amount of time spent in this health state using linear interpolation between time points.

In both analyses, costs will be measured from a societal perspective including health-care costs (such as the costs for a year of DMT, costs for the extra surveillance including additional medical tests as MRI, costs for visits to other medical doctors etc), direct nonmedical costs (costs that patients make for travelling to and from the hospital, private payments for extra health-care consumption etc) and indirect nonmedical costs (costs due to loss of production and short or long-term sick absence). The latter is important as previous research has shown that productivity losses are an important cost driver in severe MS. Health-care costs and direct nonmedical costs will be measured using the iMTA Medical Consumption Questionnaire (iMCQ) at 3 months, 6, months, 12 months, 18 months and 24 months. The iMCQ measures the health-care costs in the last three months. As these patients are assumed to be stable regarding their disease progression, these follow-up moments will provide an adequate estimation of their health-care use. Primary and secondary health-care costs will be valued using Dutch standard costs. If unavailable, tariffs or costs reported by the literature will be used. Medication will be valued using www.medicijnkosten.nl whereas informal care will be valued based on the costs of household care as reported by the Centraal Administratie Kantoor.

For indirect nonmedical costs, patients will be asked for their employment situation and both short-term and long-term sick absence because of MS (or MS related treatment) using the

iMTA Productivity Cost Questionnaire (iPCQ) at 3 months, 6, months, 12 months, 18 months and 24 months. The iPCQ measures productivity loss in the last four weeks which will give an adequate overview of the productivity losses between each time point as these patients are assumed to be stable. Costs of absenteeism from paid work will be calculated according to both the human capital and friction cost approach. Costs of presenteeism will be calculated by asking participants how many working hours should have been replaced due to less productivity at work. Lost productivity due to either absenteeism or presenteeism will be valued using the mean age-, and sex specific income of the Dutch population. Costs of productivity losses due to absenteeism from unpaid work and informal care will be calculated using the standard wage of a professional housekeeper. All costs will be indexed to the year at which the trial ended. Missing data on costs and effects will be imputed using multiple imputations. In addition, costs and effects will both be discounted using a 3% discount rate.

For the cost-effectiveness analysis, we will calculate incremental cost-effectiveness ratios (ICERs) which is defined as the difference in mean costs divided by the difference in mean effects between the treatment continuation group and the treatment discontinuation group. For the cost-utility analysis, we will calculate incremental cost-utility ratios (ICURs) which is the difference in mean costs divided by the difference in mean QALYs. Bootstrapping with 5,000 replications will be used to estimate 95% credibility intervals around the ICERs and ICURs. The bootstrapped cost-effect pairs will be plotted on a cost-effectiveness plane and used to estimate cost-effectiveness acceptability curves (CEACs). CEACs show the probability that the intervention is cost-effective in comparison with the control treatment for a range of ceiling ratios. The ceiling ratio is defined as the willingness-to-pay, which is the amount of money society is willing to pay to gain one unit of effect.

In a sensitivity analysis, we will repeat all analyses using a healthcare payer perspective. In this analysis, only direct healthcare costs will be included. In addition, we will conduct subgroup analysis in which we will stratify individuals based on the presence of blood-based markers predictive for return of inflammatory disease activity.

Furthermore, we will conduct a budget impact analysis. A budget impact analysis (BIA) focuses on the expected changes in the expenditure of a health care system after the adoption of a new standard of care. In this BIA, we aim to estimate the future yearly budget impact of discontinuation of first line medication in patients with long-term stable relapsing-onset from a Dutch perspective. The BIA will be performed according to the BIA framework of the International Society for Pharmacoeconomics and Outcomes Research (ISPOR). This framework consists of several standard aspects: target population, scenario distribution

based on hospital types, resource utilisation, costs per unit, total costs, and sensitivity analyses.

10. ETHICAL CONSIDERATIONS

10.1 Regulation statement

The study will be conducted according to the principles of the Declaration of Helsinki (World Medical Association, 2013, Brazil) and in accordance with the Medical Research Involving Human Subjects Act (WMO) and the Good Clinical Practice guidelines.

10.2 Recruitment and consent

Patients will be informed about the study in different ways. Patients can be notified by their treating doctor during outpatient consultations at the VUmc or one of the other participating centres. In addition, a notification will be placed on the website of the MS Centre Amsterdam and the websites of the MS Vereniging Nederland (MSVN) and MS Web with information about the study. Patients can then discuss potential interest to continue in the study with their treating neurologist. Continuation of the study is possible in one of the participating centers. Participants who express their wish to continue participation will receive additional information on paper about the purpose, intervention, duration and content of the study. They will also receive an informed consent form with careful and extensive information about the possible risks (according to local ethical review board requirements). In case there are any questions about the study, the study coordinator can be contacted. Moreover, an independent doctor (dr. Pijnenburg) can be contacted for additional questions.

Continuation of participation in the study will take place after the written informed consent form has been returned to the trial coordinator. A copy of the informed consent form will be given to the participant and to the responsible physician in one of the participating centers in case the patient is recruited through one of the centers outside the VUmc. This consent can be revoked at any time without citing reasons. Patients will be given a minimum of 2 weeks to consider their decision. The maximum time will be as long as the inclusion of patients is ongoing and inclusion and exclusion criteria are met.

10.3 Objection by minors or incapacitated subjects (if applicable)

Not applicable, all participants will be adult and legally competent

10.4 Benefits and risks assessment, group relatedness

Participants that were assigned to continue their medication are expected to remain stable throughout the study period based on their proven stable status for 5 consecutive years prior to enrolment. Therefore, this group will face no potential risks and no direct benefit other than

the usual medical care. The potential value of the outcome of the research will outweigh the burden of participation for the study.

The data on the discontinuation of therapy in long-term inflammatory stable MS patients is reassuring. One large retrospective cohort study has shown in 1200 patients that stable RRMS patients who stop treatment don't have an increased relapse rate compared to patients who continue treatment[25]. There was a slightly higher disability progression of the discontinuation group, presumably based on the discontinuation of treatment in patients with progressive MS. Also, there seems to be no rebound inflammation after discontinuation of therapy in progressive MS, and a similar relapse rate as the years prior to discontinuation[26]. Lastly, one study has showed that patients 45 years or older, or patients with a DMT intake of 4 or more years without evidence of clinical or radiological disease activity showed a high likelihood of remaining relapse-free after discontinuation and absence of contrast enhancing lesions[27].

From March 2023, we adjusted our protocol based on the interim analyses, which show a higher percentage of disease activity in patients that discontinued DMT. For this reason, patients that were assigned to discontinue the previously used DMT were advised to reinitiate their DMT in consultation with their treating neurologist.

10.5 Compensation for injury

According to article 7 from the 'Wet medisch-wetenschappelijk onderzoek met mensen' (Staatsblad 1998, 161) an insurance is obtained by the VUmc. In case of injury or death of the participants because of the study, this insurance will compensate for injury or cover the cost caused by death or injury from the participants. The insurance is obtained by the Onderlinge Waarborgmaatschappij Centramed b.a., Postbus 7374, 2701 AJ Zoetermeer. The insurance company and the insurance accede to the decree mandatory insurance for 'medisch-wetenschappelijk onderzoek met mensen (Staatsblad 2003, 266). Written information about the insurance will be provided for the participants.

10.6 Incentives (if applicable)

According to the current standard of care, the evaluation of every patient treated with immunomodulating therapy is at baseline, 12 and 24 months. Since visits are also scheduled at 3, 6 and 18 months, travel expenses and parking costs for these extra visits will be compensated. Participants will be compensated for their time and effort for study participation: they will receive a gift certificate of €25 upon study completion.

11. ADMINISTRATIVE ASPECTS, MONITORING AND PUBLICATION

11.1 Handling and storage of data and documents

Data will be handled confidentially . After collection, all data will be correctly labeled and securely stored. A subject identification code (SIC) will be used to link data to the subject. The SIC will consist of numbers and will not be based on the patient initials and birth-date. The key to the code will be kept separately from the coded data. The only people who have access to this code will be the principal investigator, the coordinating investigator and the corresponding investigator. No other people will have access to the link information. Great care will be taken to ensure that there is no link between SIC and information on which an individual can be identified. The handling of personal data in the database complies with the General Data Protection Regulation (De Algemene Verordening Gegevensbescherming) Potential data exchange with other countries will only take place after consent of the patient and handling of data will comply with the General Data Protection Regulation. The procedure for handling data includes data encryption, coding, secure storage, establishing limited access or varying levels of access to the biobank, removing identifying information from bio specimens and data. The infrastructure will consist of both hardware and software components, to prevent unauthorized access to databases.

An electronic case report form (CRF) will be developed to document the data collected in the study. This database will include demographic and patients characteristics (without birth date) and all outcomes of the study measures. Other investigators can request permission to get access to (a part) of this database for the purpose of research only, and only when the principal investigator gives permission. These investigators will not get access to the separate database which includes the participants' names, other identifiers and the SIC. All data will be stored on a computer protected with a password on the VUmc computer network. And access to the database will also be secured by a code. Only the trial coordinator and the principal investigator will know the code that gives access to the database with the link information.

After finalizing the study, the originals of all source documents will be stored for a period of 15 years in a locked room. Data that is collected and stored for the Biobank, will be stored for a period of 50 years. This period of storage has been determined to ensure that a follow-up study might be possible. In case of a follow-up study, a new protocol will be submitted to the METC and participants will have to sign a new informed consent form. Importantly, participants will only be approached for a follow-up study if they have indicated on the

informed consent form of the current study that they can be approached for a follow-up study.

The collection of data for medical research in the Netherlands is subject to the Personal Data Protection Act and in particular to the Medical Treatment Contracts Act.

Data storage Neurokeys

Data collected by NeuroKeys will be stored using Amazon AWS S3. There will be no identifiable information in this database, all keystroke and sensor data are logged with ID numbers only. A separate database, Amazon RDS (SQL server), will be used to store the verification code and personal information such as gender and year of birth, which can be used to send users push notifications. Both Amazon AWS S3 and Amazon RDS databases are located in Frankfurt, Germany, and are ISO 27001/27017/27018 compliant. All data is AES-256 encrypted in transit and at rest, a tokenization approach is used in which a sensitive data element is replaced by a non-sensitive equivalent and send by using a secure SSL link. Decryption keys are stored in a private encrypted environment. For iOS users, each time the NeuroKeys' keyboard is 'activated' (i.e. a new message is started), data of the previous keyboard session is uploaded to the database. Data of only one message is saved on the mobile phone, until a new message has started. For Android users data is uploaded to the database approximately every 4 hours.

Data storage MS sherpa

Data collected by MS sherpa will be stored using MongoDB Atlas, whose infrastructure runs on top of Amazon Web Services (ISO 27001/27017/27018 compliant) in Dublin, Ireland. General profile information (such as gender, age, length, weight, and e-mail address) is directly saved on AWS servers, in Dublin and AWS S3 in Frankfurt, Germany. Auth0 is used for user authentication and authorisation (ISO 27001/27018 compliant) and its EU office is based in London, UK. MongoDB Atlas and Auth0 achieved key compliance controls and objectives, as demonstrated by the completion of a Type 1 SOC 2 Report: Security. MongoDB, Inc. and Auth0 are also certified under the EU-US Privacy Shield. Data gathered via MS sherpa will be transferred to the database immediately after the data has been collected.

11.2 Monitoring and Quality Assurance

An independent monitor, the Clinical Research Bureau (CRB) of the VUmc, will monitor the proposed study according to Good Clinical Practice (GCP). For a selection of candidates

Informed consent is to be checked by the CRB. Besides that, source data verification is performed during the onsite monitoring. The conformity of the data used for analysis and the information in the patient files will be checked by the CRB. The intensity of the verification will be related to the risk arisen by the research. Inclusion and exclusion criteria will be checked as well as the main outcome measures. The CRB will check if the (S)AE's and SUSAR's are reported conforming the schedule as required by laws and regulations.

The quality assurance team under the leadership of a quality assurance manager (QAM) is responsible for providing an effective and efficient quality assurance system and counsel for the clinical research sites. In this quality assurance system, the QAM is responsible for ensuring appropriate global and affiliate-specific quality documents are developed and tracked, making sure they maintain an up-to-date overall inventory of all quality documents. Furthermore, the QAM and its team are responsible for ensuring all personnel involved in the clinical trial are properly qualified and trained for the job roles for which they are responsible. They are responsible for giving the personnel trainings and constantly assessing further opportunities for education and additional training. The quality assurance team is also responsible for checking compliance with the protocol, SOPs, GCP, and/or applicable regulatory requirement(s) and checking of the quality in all stages of data handling to ensure that all data are reliable and have been processed correctly. Moreover, the quality assurance team is responsible for auditing the various investigational sites.
If noncompliance with the protocol, SOPs, GCP, and/or applicable regulatory requirement(s) by and investigator/institution, or by member(s) or the sponsor's staff is detected during a quality assurance activity or audit, it is the responsibility of the QAM to report this to the trial's sponsor and principal investigator.

Quality assurance procedures:

Quality assurance is the systematic and independent examination of all clinical trial-related activities and documentations. The quality assurance procedure focuses on clinical investigator audits and audits of clinical trial documentation.

1. Document audits:

During the document audits, the quality assurance team oversees the documents that are generated before, during or at the end of the conduct of the clinical trial. For each document, a checklist is developed based on the relevant regulatory and organizational standards and SOPs. The aim of the audits is to ensure that the information and data in the documents are

complete, clear, reliable and consistent. Documents reviewed in the document audits include the clinical study protocol, the investigator's brochure and the clinical study report.

2. Clinical investigator audits:

The clinical investigator audits concern audits of the different research sites of the trial. They are performed to assess the site's regulatory compliance and clinical data quality (including adherence to the protocol). Paragraph 3 will address the procedures concerning these audits.

11.3 Amendments

Amendments are changes made to the research after a favourable opinion by the accredited METC has been given. All amendments will be notified to the METC that gave a favourable opinion. All amendments will be notified to the METC and to the competent authority.

11.4 Annual progress report

The investigator will submit a summary of the progress of the trial to the accredited METC once a year. Information will be provided on the date of inclusion of the first subject, numbers of subjects included and numbers of subjects that have completed the trial, serious adverse events/ serious adverse reactions, other problems, and amendments. The METC will also be informed on the start and end date of the trial.

11.5 Temporary halt and (prematurely) end of study report

The investigator/sponsor will notify the accredited METC of the end of the study within a period of 8 weeks. The end of the study is defined as the last patient's last visit.
The sponsor will notify the METC immediately of a temporary halt of the study, including the reason of such an action.
In case the study is ended prematurely, the sponsor will notify the accredited METC within 15 days, including the reasons for the premature termination.
Within one year after the end of the study, the investigator/sponsor will submit a final study report with the results of the study, including any publications/abstracts of the study, to the accredited METC.

11.6 Public disclosure and publication policy

This study will be registered in het Nederlands Trial Register (NTR) http://www.trialregister.nl and www.clinicaltrials.gov. Publication will be in accordance with the basic principles of

CCMO statement on publication policy. The results will be presented at (inter)national scientific meetings. The results will be published in a medical scientific journal. In none of the publication forms, participant identity will be disclosed.

12. STRUCTURED RISK ANALYSIS

12.1 Potential issues of concern

Paragraph 13.1 is not applicable.

12.2 Synthesis

The intervention in this study is the discontinuation of previously used DMT. No new products or agents are administered, nor will there be any dosage adjustments in the group that will continue their therapy. The specific DMT's that patients use prior to discontinuation, and that are used in the control group are all registered with the authorities and widely used for this specific indication. Although previous studies suggest that the risk of return of inflammatory activity after discontinuing DMT will be low in long-term stable RRMS patients (as also described in section 1), this is the main risk of the intervention. To monitor return of inflammatory activity, a safety strategy is built in, which is described in section 10.4. If safety criteria are exceeded, the study will be discontinued and DMT's will be reinitiated (in one patient group or in all patients, see section 10.4). A DSMB is appointed that will monitor the decision making on premature termination every 3 months (section 9.5).

From March 2023 on, patients will no longer be randomized into the intervention (discontinuation of DMT) or control arm. This study design will be observational and the aforementioned built-in safety strategy and interim analyses from DSMB will no longer hold.

13. REFERENCES

1 Goodin, D. S. The epidemiology of multiple sclerosis: insights to disease pathogenesis. *Handb Clin Neurol* **122**, 231-266, doi:10.1016/B978-0-444-52001-2.00010-8 (2014).

2 Weinshenker, B. G. Natural history of multiple sclerosis. *Ann Neurol* **36 Suppl**, S6-11 (1994).

3 Reich, D. S., Lucchinetti, C. F. & Calabresi, P. A. Multiple Sclerosis. *N Engl J Med* **378**, 169-180, doi:10.1056/NEJMra1401483 (2018).

4 Giovannoni, G. *et al.* Is it time to target no evident disease activity (NEDA) in multiple sclerosis? *Mult Scler Relat Disord* **4**, 329-333, doi:10.1016/j.msard.2015.04.006 (2015).

5 Arnold, D. L. *et al.* Peginterferon beta-1a improves MRI measures and increases the proportion of patients with no evidence of disease activity in relapsing-remitting multiple sclerosis: 2-year results from the ADVANCE randomized controlled trial. *BMC Neurol* **17**, 29, doi:10.1186/s12883-017-0799-0 (2017).

6 Havrdova, E. *et al.* Effect of delayed-release dimethyl fumarate on no evidence of disease activity in relapsing-remitting multiple sclerosis: integrated analysis of the phase III DEFINE and CONFIRM studies. *Eur J Neurol* **24**, 726-733, doi:10.1111/ene.13272 (2017).

7 Miller, A. E. *et al.* Oral teriflunomide for patients with a first clinical episode suggestive of multiple sclerosis (TOPIC): a randomised, double-blind, placebo-controlled, phase 3 trial. *Lancet Neurol* **13**, 977-986, doi:10.1016/S1474-4422(14)70191-7 (2014).

8 Freedman, M. S. *et al.* Moving toward earlier treatment of multiple sclerosis: Findings from a decade of clinical trials and implications for clinical practice. *Mult Scler Relat Disord* **3**, 147-155, doi:10.1016/j.msard.2013.07.001 (2014).

9 Ramsaransing, G. S. & De Keyser, J. Benign course in multiple sclerosis: a review. *Acta Neurol Scand* **113**, 359-369, doi:10.1111/j.1600-0404.2006.00637.x (2006).

10 Sartori, A., Abdoli, M. & Freedman, M. S. Can we predict benign multiple sclerosis? Results of a 20-year long-term follow-up study. *J Neurol* **264**, 1068-1075, doi:10.1007/s00415-017-8487-y (2017).

11 Benedikz, J. *et al.* The natural history of untreated multiple sclerosis in Iceland. A total population-based 50 year prospective study. *Clin Neurol Neurosurg* **104**, 208-210 (2002).

12 Perini, P., Tagliaferri, C., Belloni, M., Biasi, G. & Gallo, P. The HLA-DR13 haplotype is associated with "benign" multiple sclerosis in northeast Italy. *Neurology* **57**, 158-159 (2001).

13 Johnson, K. M., Zhou, H., Lin, F., Ko, J. J. & Herrera, V. Real-World Adherence and Persistence to Oral Disease-Modifying Therapies in Multiple Sclerosis Patients Over 1 Year. *J Manag Care Spec Pharm* **23**, 844-852, doi:10.18553/jmcp.2017.23.8.844 (2017).

14 Lattanzi, S. *et al.* Persistence to oral disease-modifying therapies in multiple sclerosis patients. *Journal of neurology* **264**, 2325-2329, doi:10.1007/s00415-017-8595-8 (2017).

15 Lanzillo, R. *et al.* A multicentRE observational analysiS of PErsistenCe to Treatment in the new multiple sclerosis era: the RESPECT study. *Journal of neurology* **265**, 1174-1183, doi:10.1007/s00415-018-8831-x (2018).

16 Vermersch, P. *et al.* Teriflunomide versus subcutaneous interferon beta-1a in patients with relapsing multiple sclerosis: a randomised, controlled phase 3 trial. *Multiple sclerosis (Houndmills, Basingstoke, England)* **20**, 705-716, doi:10.1177/1352458513507821 (2014).

17 Balak, D. M. *et al.* Prevalence of cutaneous adverse events associated with long-term disease-modifying therapy and their impact on health-related quality of life in patients with multiple sclerosis: a cross-sectional study. *BMC neurology* **13**, 146, doi:10.1186/1471-2377-13-146 (2013).

18 Rommer, P. S. & Zettl, U. K. Managing the side effects of multiple sclerosis therapy: pharmacotherapy options for patients. *Expert opinion on pharmacotherapy* **19**, 483-498, doi:10.1080/14656566.2018.1446944 (2018).

19 Lee Mortensen, G. & Rasmussen, P. V. The impact of quality of life on treatment preferences in multiple sclerosis patients. *Patient Prefer Adherence* **11**, 1789-1796, doi:10.2147/ppa.S142373 (2017).

20 La Mantia, L., Munari, L. M. & Lovati, R. Glatiramer acetate for multiple sclerosis. *Cochrane Database Syst Rev*, Cd004678, doi:10.1002/14651858.CD004678.pub2 (2010).

21 Gold, R. *et al.* Placebo-controlled phase 3 study of oral BG-12 for relapsing multiple sclerosis. *N Engl J Med* **367**, 1098-1107, doi:10.1056/NEJMoa1114287 (2012).

22 Fox, R. J. *et al.* Placebo-controlled phase 3 study of oral BG-12 or glatiramer in multiple sclerosis. *N Engl J Med* **367**, 1087-1097, doi:10.1056/NEJMoa1206328 (2012).

23 Sejbaek, T., Nybo, M., Petersen, T. & Illes, Z. Real-life persistence and tolerability with dimethyl fumarate. *Mult Scler Relat Disord* **24**, 42-46, doi:10.1016/j.msard.2018.05.007 (2018).

24 CIBG; ministerie van Volksgezondheid, W. e. S. *Prijsvorming*, <www.farmatec.nl> (z.d.).

25 Kister, I. *et al.* Discontinuing disease-modifying therapy in MS after a prolonged relapse-free period: a propensity score-matched study. *J Neurol Neurosurg Psychiatry* **87**, 1133-1137, doi:10.1136/jnnp-2016-313760 (2016).

26 Bonenfant, J. *et al.* Can we stop immunomodulatory treatments in secondary progressive multiple sclerosis? *Eur J Neurol* **24**, 237-244, doi:10.1111/ene.13181 (2017).

27 Bsteh, G. *et al.* Discontinuation of disease-modifying therapies in multiple sclerosis - Clinical outcome and prognostic factors. *Mult Scler* **23**, 1241-1248, doi:10.1177/1352458516675751 (2017).

28 O'Rourke, K. E. & Hutchinson, M. Stopping beta-interferon therapy in multiple sclerosis: an analysis of stopping patterns. *Mult Scler* **11**, 46-50, doi:10.1191/1352458505ms1131oa (2005).

29 Rio, J. *et al.* Factors related with treatment adherence to interferon beta and glatiramer acetate therapy in multiple sclerosis. *Mult Scler* **11**, 306-309, doi:10.1191/1352458505ms1173oa (2005).

30 CBO richtlijn (2012) Immunomodulerende en immunosuppressieve behandeling bij multipele sclerose. (2012).

31 Kennisagenda neurologie 2017: https://gallery.mailchimp.com/29087cdad5c58a12bd346e83f/files/3b5692f1-3840-48e2-99e7-405edb9a895f/Kennisagenda_Neurologie_eindversie_16_12_2017.pdf.

32 Kister, I. & Corboy, J. R. Reducing costs while enhancing quality of care in MS. *Neurology* **87**, 1617-1622, doi:10.1212/WNL.0000000000003113 (2016).

33 Uher, T. *et al.* Is no evidence of disease activity an achievable goal in MS patients on intramuscular interferon beta-1a treatment over long-term follow-up? *Mult Scler* **23**, 242-252, doi:10.1177/1352458516650525 (2017).

34 Rotstein, D. L., Healy, B. C., Malik, M. T., Chitnis, T. & Weiner, H. L. Evaluation of no evidence of disease activity in a 7-year longitudinal multiple sclerosis cohort. *JAMA Neurol* **72**, 152-158, doi:10.1001/jamaneurol.2014.3537 (2015).

35 De Stefano, N. *et al.* Long-term assessment of no evidence of disease activity in relapsing-remitting MS. *Neurology* **85**, 1722-1723, doi:10.1212/WNL.0000000000002105 (2015).

36 Gray, O., McDonnell, G. & Hawkins, S. Tried and tested: the psychometric properties of the multiple sclerosis impact scale (MSIS-29) in a population-based study. *Mult Scler* **15**, 75-80, doi:10.1177/1352458508096872 (2009).

37 Pfennings, L. E. *et al.* A health-related quality of life questionnaire for multiple sclerosis patients. *Acta Neurol Scand* **100**, 148-155 (1999).

38 Rietberg, M. B., Van Wegen, E. E. & Kwakkel, G. Measuring fatigue in patients with multiple sclerosis: reproducibility, responsiveness and concurrent validity of three Dutch self-report questionnaires. *Disabil Rehabil* **32**, 1870-1876, doi:10.3109/09638281003734458 (2010).

39 Eagle, T. *et al.* Treatment satisfaction across injectable, infusion, and oral disease-modifying therapies for multiple sclerosis. *Mult Scler Relat Disord* **18**, 196-201, doi:10.1016/j.msard.2017.10.002 (2017).

40 Kobelt, G., Berg, J., Lindgren, P. & Jonsson, B. Costs and quality of life in multiple sclerosis in Europe: method of assessment and analysis. *Eur J Health Econ* **7 Suppl 2**, S5-13, doi:10.1007/s10198-006-0365-y (2006).

41 Janssen, M. F. *et al.* Measurement properties of the EQ-5D-5L compared to the EQ-5D-3L across eight patient groups: a multi-country study. *Qual Life Res* **22**, 1717-1727, doi:10.1007/s11136-012-0322-4 (2013).

42 Bouwmans, C. *et al.* The iMTA Productivity Cost Questionnaire: A Standardized Instrument for Measuring and Valuing Health-Related Productivity Losses. *Value Health* **18**, 753-758, doi:10.1016/j.jval.2015.05.009 (2015).
