# Supplementary material for: Discontinuation of First-Line Disease-Modifying Therapy in Patients With Stable Multiple Sclerosis: The DOT-MS Randomized Clinical Trial
Source: JAMA Neurol. 2024 Dec 9;82(2):123–31. doi: 10.1001/jamaneurol.2024.4164 (PMC11811793; doi:10.1001/jamaneurol.2024.4164)
Supplement: Supplement 2. — eMethods 1. Inclusion Criteria eMethods 2. Exclusion Criteria eMethods 3. Definition of Relapse eMethods 4. Secondary End Points eMethods 5. Statistical Analysis of Secondary End Points eTable 1. Incidence Rates of Disease Activity eTable 2. Length of Follow-Up eTable 3. Participants With Relapse and/or MRI Activity eTable 4. Baseline Characteristics of Participants With and Without "Significant" Disease Activity eTable 5. Case Descriptions of Participants With “Significant” Disease Activity or “Any” MRI Activity eTable 6. Baseline Characteristics of Participants With and Without "Any" MRI Activity eTable 7. Linear Mixed-Effects Model Regarding Absolute Serum NfL Level in the Continue and Discontinue Group eTable 8. Linear Mixed-Effects Model Regarding Absolute Serum NfL Level in “Significant" Disease Activity at Participant Level eTable 9. Linear Mixed-Effects Model Regarding Absolute Serum NfL Level in "Significant" Disease Activity at Visit Level eTable 10. Linear Mixed-Effects Model Regarding Absolute Serum NfL Level in "Any" MRI Activity at Participant Level eTable 11. Linear Mixed-Effects Model Regarding Absolute Serum NfL Level in "Any" MRI Activity Disease Activity at Visit Level eTable 12. Linear Mixed-Effects Model Regarding Absolute Serum GFAP Level in the Continue and Discontinue Group eTable 13. Linear Mixed-Effects Model Regarding Absolute Serum GFAP Level in "Significant" Disease Activity at Participant Level eTable 14. Linear Mixed-Effects Model Regarding Absolute Serum GFAP Level in "Significant" Disease Activity at Visit Level eTable 15. Linear Mixed-Effects Model Regarding Absolute Serum GFAP Level in “Any” MRI Activity at Participant Level eTable 16. Linear Mixed-Effects Model Regarding Absolute Serum GFAP Level in “Any” MRI Activity Disease Activity Visit Level eTable 17. Median (IQR) Expanded Disability Status Scale (EDSS) Change by Group eTable 18. Proportions of Significant Confirmed Disability Progression eTable 19. Mean Percentage (SD) Chan [file jamaneurol-e244164-s002.pdf]

Supplemental Online Content

Coerver EME, Fung WH, de Beukelaar, et al. Discontinuation of first-line disease-modifying therapy in patients with stable multiple sclerosis: the DOT-MS randomized clinical trial. *JAMA Neurol*. Published online December 9, 2024. doi:10.1001/jamaneurol.2024.4164

eMethods 1. Inclusion Criteria

eMethods 2. Exclusion Criteria

eMethods 3. Definition of Relapse

eMethods 4. Secondary End Points

eMethods 5. Statistical Analysis of Secondary End Points

eTable 1. Incidence Rates of Disease Activity

eTable 2. Length of Follow-Up

eTable 3. Participants With Relapse and/or MRI Activity

eTable 4. Baseline Characteristics of Participants With and Without "Significant" Disease Activity

eTable 5. Case Descriptions of Participants With “Significant” Disease Activity or “Any” MRI Activity

eTable 6. Baseline Characteristics of Participants With and Without "Any" MRI Activity

eTable 7. Linear Mixed-Effects Model Regarding Absolute Serum NfL Level in the Continue and Discontinue Group

eTable 8. Linear Mixed-Effects Model Regarding Absolute Serum NfL Level in “Significant" Disease Activity at Participant Level

eTable 9. Linear Mixed-Effects Model Regarding Absolute Serum NfL Level in "Significant" Disease Activity at Visit Level

eTable 10. Linear Mixed-Effects Model Regarding Absolute Serum NfL Level in "Any" MRI Activity at Participant Level

eTable 11. Linear Mixed-Effects Model Regarding Absolute Serum NfL Level in "Any" MRI Activity Disease Activity at Visit Level

eTable 12. Linear Mixed-Effects Model Regarding Absolute Serum GFAP Level in the Continue and Discontinue Group

eTable 13. Linear Mixed-Effects Model Regarding Absolute Serum GFAP Level in "Significant" Disease Activity at Participant Level

eTable 14. Linear Mixed-Effects Model Regarding Absolute Serum GFAP Level in "Significant" Disease Activity at Visit Level

© 2024 Coerver et al. *JAMA Neurology.*

eTable 15. Linear Mixed-Effects Model Regarding Absolute Serum GFAP Level in “Any” MRI Activity at Participant Level

eTable 16. Linear Mixed-Effects Model Regarding Absolute Serum GFAP Level in “Any” MRI Activity Disease Activity Visit Level

eTable 17. Median (IQR) Expanded Disability Status Scale (EDSS) Change by Group

eTable 18. Proportions of Significant Confirmed Disability Progression

eTable 19. Mean Percentage (SD) Change of NfL and GFAP Levels of Participants With Confirmed Disability Progression

eTable 20. Mean (SD) Symbol Digit Modalities Test (SDMT) Change by Group

eTable 21. Median (IQR) Timed 25-Foot Walk (T25-FW) Change by Group

eTable 22. Median (IQR) 9-Hole Peg Test (9-HPT) Change by Group

eTable 23. Median (IQR) MS Impact Scales (MSIS-29) by Group

eTable 24. Significant Change in MS Impact Scales (MSIS-29) by Group

eTable 25. Median (IQR) Checklist Individual Strength (CIS20R) by Group

eTable 26. Significant Change in Fatigue Severity in Checklist Individual Strength (CIS20R)

eTable 27. Median (IQR) Short Form Health Survey (SF-36) by Group

eTable 28. Significant Change in Short Form Health Survey (SF-36) by Group

eTable 29. Treatment Satisfaction by Group

eReferences.

This supplemental material has been provided by the authors to give readers additional information about their work.

© 2024 Coerver et al. *JAMA Neurology.*

eMethods 1. Inclusion Criteria

- A minimum age of 18 years
- Ability to understand the purpose and risks of the study and provide signed and dated informed consent and authorization to use protected health information (PHI) in accordance with national and local privacy regulations.
- Definite diagnosis of relapsing-onset MS according to the revised McDonald 2017 criteria
- All relapsing-onset MS patients treated with one of the first-line treatments: any of the interferons, glatiramer acetate, dimethylfumarate, teriflunomide
- Complete absence of inflammatory activity (no objectively defined and confirmed relapses, no significant number (2 or more) of new-T2 lesions suggestive of demyelination and no contrast-enhancing lesions) suggestive of demyelination for 5 consecutive years under first-line treatment at start of inclusion. In case the last available MRI-scan was conducted 10 or more years ago, no more than 3 new T2-lesions suggestive of demyelination in the last 10 years are accepted.

© 2024 Coerver et al. *JAMA Neurology.*

eMethods 2. Exclusion Criteria

- A switch between first-line disease modifying therapy over two years prior to inclusion, in case the switch has been due to in effectivity of the first DMT. In case the switch has been due to side-effects or by a personal preference of the patient (such as the wish to switch to oral therapies), this is not considered as an exclusion criterium.
- Women who want to discontinue medication because of a pregnancy wish and women who are pregnant or expect to become pregnant during the study period 3. Patients that have previously used interferon-beta and have been tested positive for neutralizing antibodies (NAbs). This is determined by measuring MxA-bioactivity and is a test that is part of routine follow-up in patients that use interferon-beta. The reason for this is that development of NAbs has been shown to affect interferon-beta treatment efficacy
- Medication switches between first-line DMTs in these 5 years prior to inclusion were allowed if they happened for the reason of side effects or personal preference. Participants previously using interferon-beta and testing positive for neutralizing antibodies, which has been associated with a worse disease course, were excluded.[1]

© 2024 Coerver et al. *JAMA Neurology.*

eMethods 3. Definition of Relapse

Confirmed relapse was defined according to the definition most often used in MS phase-III trials; the onset of new or recurrent symptoms that last >24 hours, that are accompanied by new objective abnormalities on a neurological examination and that are not explained by non-MS processes such as fever, infection, severe stress or drug toxicity.

© 2024 Coerver et al. *JAMA Neurology.*

eMethods 4. Secondary End Points

Significant confirmed disability progression was defined as an increase of ≥1.0 point from the baseline EDSS score if the baseline score was ≤5.5 or an increase of ≥0.5 points if the baseline score was >5.5, sustained for at least 24 weeks.

Other secondary outcome measures included EDSS, MSFC and the patient-reported outcome measures such as MSIS-29, CIS20r, SF-36 and the treatment satisfaction question. Significant change on the MSIS-29, CIS20r and SF-36 was defined as a change of at least 7.5 points, 8.0 points and 5.0 points, respectively, based on previous literature.[2-5]

© 2024 Coerver et al. *JAMA Neurology.*

eMethods 5. Statistical Analysis of Secondary End Points

Secondary endpoints were analyzed using Mann-Whitney test, two sample T-test or χ^2 test to compare EDSS, MSFC and patient-reported outcome measures between continue and discontinue group. For confirmed disability progression, MSIS-29, CIS20r and SF-36, we also compared the proportion of participants with significant change using χ^2 test. For longitudinal trajectories of NfL and GFAP levels, several linear mixed-effects models were used. We first modeled overall differences between the randomization groups (continue vs discontinue group) using participant as a random effect. Next, we compared differences between all longitudinal measurements of participants with and without "significant" disease activity or "any" MRI activity (participant level comparisons). For this we used disease activity at the participant level, randomization group and time point as fixed effects. Then we compared the presence of disease activity at individual visits of participants and within a 3-month window before and after (visit level comparisons) to study if changes in biomarker values may also solely occur at the moment of disease recurrence rather than over the complete period of observation.

© 2024 Coerver et al. *JAMA Neurology.*

eTable 1. Incidence Rates of Disease Activity

	**Continuation**	**Discontinuation**	**Incidence rate difference (95% CI)**	**p value**
Primary outcome event (relapse and/or “significant” MRI activity)	0	0.011	0.011 (0.003-0.019)	0.99
Relapse	0	0.003	0.003 (-0.001-0.007)	1.00
“Significant” MRI activity	0	0.010	0.010 (0.002-0.017)	0.99
“Any” MRI activity	0.002	0.016	0.014 (0.005-0.025)	0.02
p value from Poisson regression analysis. Incidence rate represents number of events/total follow-up duration per treatment group.				

© 2024 Coerver et al. *JAMA Neurology.*

eTable 2. Length of Follow-Up

	**Continuation (n=44)**	**Discontinuation (n=45)**	**p value**
Total follow-up, months, median (IQR)[a]	14.6 (11.6-24.0)	18.1 (11.3-23.8)	0.90
Follow-up time, n (%)			
< 6 months	3 (7.1%)	3 (7.1%)	
6-11 months	11 (26.2%)	11 (26.2%)	
12-17 months	11 (26.2%)	8 (19.0%)	
18-23 months	6 (14.3%)	16 (38.1%)	
≥24 months	11 (26.2%)	7 (16.7%)	

p value from Mann-Whitney test. The end of study visit is included in the follow-up time. [a]Two participants in the continue group were lost in follow-up.

© 2024 Coerver et al. *JAMA Neurology.*

eTable 3. Participants With Relapse and/or MRI Activity

	**Continuation (n=1)**	**Discontinuation (n=11)**[a]
**Relapse**		
1 relapse	0	2
**“Significant” MRI activity**		
3 new T2 lesions	0	2
4 new T2 lesions	0	0
≥5 new T2 lesions	0	1
2 new contrast-enhancing lesions	0	3
3 new contrast-enhancing lesions	0	2
≥5 new contrast-enhancing lesions	0	1
**Other MRI activity**		
1 new T2 lesion	0	1
2 new T2 lesions	0	1
1 new contrast-enhancing lesion	1	5
1 enlarged T2 lesion	0	3

“Significant” MRI activity was defined as ≥3 new T2 lesions or ≥2 contrast-enhancing lesions. [a]One participant had a relapse and contrast-enhancing lesion. One participant had a relapse, contrast-enhancing lesions and new T2 lesions. Three participants had both contrast-enhancing lesions, new T2 lesions and enlarged lesion. One participant had contrast-enhancing lesions and new T2 lesions.

eTable 4. Baseline Characteristics of Participants With and Without "Significant" Disease Activity

	**All participants (n=89)**	**Discontinuation without "significant" disease activity (n=37)**	**"Significant" disease activity (n=8)[c]**	**No "significant" disease activity (n=81)**
Median age (IQR), years	54 (49.0-59.0)	54 (50.0-58.0)	46 (43.5-58.5)	54 (50.0-59.0)
Sex				
Female	60 (67.4%)	27 (73.0%)	5 (62.5%)	55 (67.9%)
Male	19 (32.6%)	10 (27.0%)	3 (37.5%)	26 (32.1%)
Median time since symptom onset (IQR), years	14.0 (9.9-21.5)	14.1 (9.3-20.8)	14.5 (13.2-16.4)	14.0 (9.5-21.6)
Median time since last documented relapse (IQR), years	9.4 (6.9-13.2)	9.3 (7.1-12.6)	9.6 (7.4-11.2)	9.5 (6.9-13.2)
Multiple sclerosis subtype				
Relapsing-remitting	80 (89.9%)	33 (89.2%)	8 (100.0%)	72 (88.9%)
Secondary progressive	9 (10.1%)	4 (10.8%)	0 (0.0%)	9 (11.1)
Median total duration of disease-modifying therapy use (IQR), years	11.2 (7.7-16.0)	11.8 (7.8-15.4)	10.3 (8.8-11.4)	11.4 (7.7-16.2)
Disease-modifying therapy at randomisation				
Interferon beta	35 (39.3%)	15 (40.5%)	2 (25.0%)	33 (40.7%)
Glatiramer acetate	23 (25.8%)	11 (29.7%)	1 (12.5%)	22 (27.2%)
Teriflunomide	12 (13.5%)	2 (5.4%)	2 (25.0%)	10 (12.3%)
Dimethyl fumarate	19 (21.3%)	9 (24.3%)	3 (27.5%)	16 (19.8%)
Expanded Disability Status Scale score	3.1 (1.8)	3.0 (2.2)	3.3 (0.9)	3.1 (1.8)
Symbol Digit Modalities Test[a]	51.6 (12.3)	51.9 (14.3)	50.8 (6.9)	51.8 (12.8)
Timed 25 Foot Walk[b]	5.3 (1.2)	5.2 (1.1)	5.4 (0.6)	5.3 (1.2)
Nine Hole Peg Test	23.4 (7.0)	23.7 (8.6)	22.6 (2.5)	23.4 (7.3)
Neurofilament Light Chain (IQR), pg/mL	11.5 (5.5)	10.6 (4.7)	12.8 (6.1)	11.4 (5.4)
Glial Fibrillary Acidic Protein (IQR), pg/mL	85.6 (41.2)	83.2 (37.9)	85.8 (16.7)	85.6 (43.0)

© 2024 Coerver et al. *JAMA Neurology.*

Median follow-up (IQR), months	15.3 (11.4-23.9)	13.6 (9.3-23.2)	21.5 (18.0-24.1)	13.8 (10.3-23.7)
Median follow-up (IQR) till disease activity, months	NA	NA	12.0 (6.0-12.0)	NA

Data are n (%) or mean (SD), unless otherwise specified. [a]Baseline Symbol Digit Modalities Test (SDMT) was missing for one participant in the no "significant" disease activity group. [b]Baseline Timed 25 Foot Walk (T25-FW) was missing for two participants in the no "significant" disease activity group. [c]All participants with "significant" disease activity were in the discontinue group. NA: not applicable.

© 2024 Coerver et al. *JAMA Neurology.*

eTable 5. Case Descriptions of Participants With "Significant" Disease Activity or "Any" MRI Activity

**Case 1 - Male, 44 years, randomized in discontinue group**						
DMT at randomisation	Interferon beta					
Time since symptom onset, years	18.08					
Time since last documented relapse, years	18.08					
Total DMT duration, years	11.08					
Relapse	No relapse					
MRI activity						
Month 3	9 CEL and 3 new T2 lesions					
Month 6	1 CEL right parietal					
Month 9 (unscheduled visit)	1 enlarged T2 lesion right anterolateral pons					
Month 12	1 CEL periventricular left frontal					
Symptoms						
Month 3	Numbness in the left half of the face					
Intervention	Restart interferon beta at month 3, but switched to ozanimod after 11 months because of disease activity					
Follow-up duration, months	24					
	**Baseline**	**Month 3**	**Month 6**	**Month 9**	**Month 12**	**EoS**
EDSS	2.0	3.0	4.0	3.0	6.0	5.5
SDMT	46	47	46	48	48	50
T25-FW	4.7	5.1	5.4	8.0	6.1	6.3
NHPT	23.2	25.0	23.2	24.0	24.4	27.1
NfL, pg/mL	13.4	52.9	36.7	24.3	21.6	15.6
GFAP, pg/mL	98.6	109.0	112.0	118.0	104.0	86.5
DMT: disease-modifying therapy, CEL: contrast-enhancing lesion, EDSS: Expanded Disability Status Scale, SDMT: Symbol Digits Modalities Test, T25-FW: Timed 25-Foot Walk, NHPT: Nine-Hole Peg Test; EoS: End of study						

© 2024 Coerver et al. *JAMA Neurology.*

Case 2 - Female, 38 years, randomized in discontinue group			
DMT at randomisation	Dimethyl fumarate		
Time since symptom onset, years	11.89		
Time since last documented relapse, years	11.76		
Total DMT duration, years	11.20		
Relapse	Yes (month 6)		
MRI activity			
Month 6 (MRI myelum)	1 CEL thoracal level 2		
Symptoms	Numbness right leg and right stomach		
Intervention	3-day course of intravenous methylprednisolone 1000mg and restart dimethyl fumarate at month 6		
Follow-up duration, months	18		
	**Baseline**	**Month 6**	**EoS**
EDSS	4.5	4.5	4.5
SDMT	63	61	62
T25-FW	5.2	4.5	4.3
NHPT	20.2	18.1	16.7
NfL, pg/mL	3.9	5.8	2.7
GFAP, pg/mL	57.7	72.2	47.3

© 2024 Coerver et al. *JAMA Neurology.*

**Case 3 - Female, 47 years, randomized in discontinue group**			
DMT at randomisation	Glatiramer acetate		
Time since symptom onset, years	13.60		
Time since last documented relapse, years	11.02		
Total DMT duration, years	12.06		
Relapse	No relapse		
MRI activity			
Month 12	2 new CEL lesions right frontal and left occipital		
Symptoms	No symptoms		
Intervention	Restart glariramer acetate at month 12		
Follow-up duration, months	24		
	**Baseline**	**Month 12**	**EoS**
EDSS	4.0	4.0	4.0
SDMT	41	48	51
T25-FW	5.2	4.1	4.3
NHPT	20.2	17.7	23.5
NfL, pg/mL	11.8	10.2	12.5
GFAP, pg/mL	92.8	80.7	98.5

© 2024 Coerver et al. *JAMA Neurology.*

**Case 4 - Male, 60 years, randomized in discontinue group**			
DMT at randomisation	Dimethyl fumarate		
Time since symptom onset, years	15.34		
Time since last documented relapse, years	9.35		
Total DMT duration, years	9.56		
Relapse	No relapse		
MRI activity			
Month 12	3 CEL left deep white matter parietal, left temporal and right temporal/hippocampus; 1 new T2 lesion right temporal		
Symptoms	No symptoms		
Intervention	Restart dimethyl fumarate at month 12		
Follow-up duration, months	24		
	**Baseline**	**Month 12**	**EoS**
EDSS	3.5	2.5	3.5
SDMT	46	53	58
T25-FW	5.4	3.9	3.7
NHPT	17.8	17.8	17.5
NfL, pg/mL	19.9	29.5	20.0
GFAP, pg/mL	93.6	108.0	104.0

© 2024 Coerver et al. *JAMA Neurology.*

**Case 5 - Female, 58 years, randomized in discontinue group**				
DMT at randomisation	Teriflunomide			
Time since symptom onset, years	36.62			
Time since last documented relapse, years	7.62			
Total DMT duration, years	12.55			
Relapse	Yes (month 18)			
MRI activity				
Month 12	2 new T2 lesions right frontal deep white matter and 2 new lesions left temporal deep white matter			
Month 15 (unscheduled visit)[a]	2 CEL occipital deep white matter and right temporal periventricular			
Month 18	1 CEL right frontal periventricular; 4 new T2 lesions left temporal juxtacortical, right temporal deep white matter, left frontal deep white matter and left frontal juxtacortical			
Symptoms				
Month 18	Numbness in legs and feet, reduced distance walking, emotional labile			
Intervention	3-day course of intravenous methylprednisolone 1000mg and restart teriflunomide at month 18			
Follow-up duration, months	24			
	**Baseline**	**Month 12**	**Month 18**	**EoS**
EDSS	2.5	3.0	3.5	3.5
SDMT	57	46	45	46
T25-FW	6.0	6.0	5.6	5.6
NHPT	25.7	23.7	25.2	24.6
NfL, pg/mL	12.8	15.1	22.3	21.0
GFAP, pg/mL	75.7	59.2	97.9	72.8

[a]Only MRI was performed at month 15 (unscheduled visit)

© 2024 Coerver et al. *JAMA Neurology.*

**Case 6 - Female, 45 years, randomized in discontinue group**				
DMT at randomisation	Interferon beta			
Time since symptom onset, years	13.74			
Time since last documented relapse, years	9.81			
Total DMT duration, years	9.51			
Relapse	No relapse			
MRI activity				
Month 6	1 CEL right parietal and 1 new T2 lesion left frontal			
Month 12	1 CEL left frontal and enlarged T2 lesion left frontal			
Symptoms				
Month 12	Numbness of right leg, fatique and decreased balance			
Intervention	Restart interferon beta at month 12			
Follow-up duration, months	18			
	**Baseline**	**Month 6**	**Month 12**	**EoS**
EDSS	4.0	4.0	4.0	4.0
SDMT	50	53	48	55
T25-FW	5.0	4.7	4.1	4.0
NHPT	24.7	22.6	20.3	21.6
NfL, pg/mL	15.1	32.8	12.6	12.4
GFAP, pg/mL	106.0	71.0	63.1	71.4

© 2024 Coerver et al. *JAMA Neurology.*

Case 7 - Male, 42 years, randomized in discontinue group			
DMT at randomisation	Dimethyl fumarate		
Time since symptom onset, years	15.80		
Time since last documented relapse, years	6.93		
Total DMT duration, years	6.72		
Relapse	No relapse		
MRI activity			
Month 3	1 CEL left frontal		
Month 6	1 CEL left parietal periventricular and 3 new T2 lesions right periventricular and right cerebellar		
Symptoms	No symptoms		
Intervention	Restart dimethyl fumarate at month 3		
Follow-up duration, months	6		
	**Baseline**	**Month 3**	**Month 6 / EoS**
EDSS	2.0	1.0	0.0
SDMT	51	53	76
T25-FW	4.8	5.0	9.1
NHPT	23.0	18.8	19.6
NfL, pg/mL	4.6	6.8	12.1
GFAP, pg/mL	68.3	70.8	67.9

© 2024 Coerver et al. *JAMA Neurology.*

**Case 8 - Female, 62 years, randomized in discontinue group**					
DMT at randomisation	Teriflunomide				
Time since symptom onset, years	5.77				
Time since last documented relapse, years	5.11				
Total DMT duration, years	5.33				
Relapse	No relapse				
MRI activity					
Month 6	1 CEL left cerebellar penduncle				
Month 9 (unscheduled visit)	1 new T2 lesion left anterior pons				
Month 12	1 new T2 lesion left cerebellar and enlarged T2 lesion left anterior pons				
Symptoms	No symptoms				
Intervention	Restart teriflunomide at month 6				
Follow-up duration, months	15				
	**Baseline**	**Month 6[a]**	**Month 9**	**Month 12**	**EoS[b]**
EDSS	3.5	NA	2.5	2.0	2.5
SDMT	52	NA	51	63	50
T25-FW	5.8	NA	6.2	5.5	5.2
NHPT	23.0	NA	24.4	20.9	23.8
NfL, pg/mL	20.6	13.9	13.2	144.3	NA
GFAP, pg/mL	93.7	62.4	61.3	76.5	NA

[a]The scheduled neurological assessment appointment was canceled by the participant. [b]Blood samples were not analyzed due to a postponed EoS visit. NA: not applicable.

© 2024 Coerver et al. *JAMA Neurology.*

Case 9 - Female, 71 years, randomized in discontinue group			
DMT at randomisation	Dimethyl fumarate		
Time since symptom onset, years	12.94		
Time since last documented relapse, years	12.46		
Total DMT duration, years	12.15		
Relapse	No relapse		
MRI activity			
Month 3	1 CEL right frontal		
Symptoms	Fatique, leg cramps		
Intervention	Restart dimethyl fumarate at month 3		
Follow-up duration, months	18		
	**Baseline**	**Month 3**	**EoS**
EDSS	3.5	2.5	4.0
SDMT	48	46	49
T25-FW	3.9	4.7	4.3
NHPT	23.0	20.9	23.8
NfL, pg/mL	12.8	14.4	18.1
GFAP, pg/mL	79.9	47.4	110.0

© 2024 Coerver et al. *JAMA Neurology.*

**Case 10 - Female, 40 years, randomized in discontinue group**			
DMT at randomisation	Glatiramer acetate		
Time since symptom onset, years	9.23		
Time since last documented relapse, years	Unknown		
Total DMT duration, years	8.23		
Relapse	No relapse		
MRI activity			
Month 18	1 CEL left occipital periventricular		
Symptoms	No symptoms		
Intervention	No intervention		
Follow-up duration, months	24		
	**Baseline**	**Month 18**	**EoS**
EDSS	1.5	1.5	3.0
SDMT	54	57	60
T25-FW	3.9	4.3	4.3
NHPT	23.0	20.4	23.8
NfL, pg/mL	10.4	9.2	10.9
GFAP, pg/mL	156.0	147.0	163.0

© 2024 Coerver et al. *JAMA Neurology.*

**Case 11 - Female, 52 years, randomized in discontinue group**			
DMT at randomisation	Glatiramer acetate		
Time since symptom onset, years	6.65		
Time since last documented relapse, years	Unknown		
Total DMT duration, years	6.39		
Relapse	No relapse		
MRI activity			
Month 6	1 CEL central semiovale/right subinsulair		
Symptoms	No symptoms		
Intervention	Restart glatiramer acetate at month 6		
Follow-up duration, months	12		
	**Baseline**	**Month 6[a]**	**EoS**
EDSS	2.5	2.0	3.0
SDMT	76	67	69
T25-FW	5.8	5.8	6.0
NHPT	24.0	23.2	23.9
NfL, pg/mL	6.2	NA	7.5
GFAP, pg/mL	55.2	NA	69.9

[a]Blood samples were not collected. NA: not applicable.

© 2024 Coerver et al. *JAMA Neurology.*

**Case 12 - Female, 59 years, randomized in continue group**			
DMT at randomisation	Glatiramer acetate		
Time since symptom onset, years	13.44		
Time since last documented relapse, years	13.23		
Total DMT duration, years	12.56		
Relapse	No relapse		
MRI activity			
Month 18	1 CEL right posterior horn		
Symptoms	No symptoms		
Intervention	No intervention		
Follow-up duration, months	24		
	**Baseline**	**Month 18**	**EoS**
EDSS	1.5	1.5	1.0
SDMT	57	57	55
T25-FW	4.0	3.7	3.5
NHPT	16.7	17.0	16.6
NfL, pg/mL	12.1	12.6	18.6
GFAP, pg/mL	89.4	105.0	113.0

© 2024 Coerver et al. *JAMA Neurology.*

eTable 6. Baseline Characteristics of Participants With and Without "Any" MRI Activity

	All participants (n=89)	"Any" MRI activity (n=12)[c]	No "any" MRI activity (n=77)
Median age (IQR), years	54 (49.0-59.0)	50 (43.5-60.3)	54 (50.0-58.0)
Sex			
Female	60 (67.4%)	9 (75.0%)	51 (66.2%)
Male	19 (32.6%)	3 (25.0%)	26 (33.8%)
Median time since symptom onset (IQR), years	14.0 (9.9-21.5)	13.5 (11.2-15.5)	14.2 (9.9-23.3)
Median time since last documenten relapse (IQR), years	9.4 (6.9-13.2)	10.4 (8.1-12.3)	9.2 (8.1-12.3)
Multiple sclerosis subtype			
Relapsing-remitting	80 (89.9%)	12 (100.0%)	68 (88.3%)
Secondary progressive	9 (10.1%)	0 (0.0%)	9 (11.7%)
Median total duration of disease-modifying therapy use (IQR), years	11.2 (7.7-16.0)	10.4 (8.1-12.3)	9.4 (6.8-13.2)
Disease-modifying therapy at randomisation			
Interferon beta	35 (39.3%)	3 (25.0%)	32 (41.5%)
Glatiramer acetate	23 (25.8%)	3 (25.0%)	20 (26.0%)
Teriflunomide	12 (13.5%)	2 (16.7%)	10 (13.0%)
Dimethyl fumarate	19 (21.3%)	4 (33.3%)	15 (19.5%)
Expanded Disability Status Scale score	3.1 (1.8)	2.9 (1.0)	3.1 (1.9)
Symbol Digit Modalities Test[a]	51.6 (12.3)	53.4 (9.2)	51.4 (12.8)
Timed 25 Foot Walk[b]	5.3 (1.2)	5.1 (0.8)	5.3 (1.2)
Nine Hole Peg Test	23.4 (7.0)	22.0 (2,7)	23.6 (7.4)
Neurofilament Light Chain (IQR), pg/mL	11.5 (5.5)	12.0 (5.3)	11.4 (5.5)
Glial Fibrillary Acidic Protein (IQR), pg/mL	85.6 (41.2)	89.0 (26.6)	85.0 (43.3)
Median follow-up (IQR), months	15.3 (11.4-23.9)	21.5 (18.0-24.1)	13.6 (9.2-23.7)
Median follow-up (IQR) till disease activity, months	NA	6.0 (5.3 - 12.0)	NA

© 2024 Coerver et al. *JAMA Neurology.*

Data are n (%) or mean (SD), unless otherwise specified. [a]Baseline Symbol Digit Modalities Test (SDMT) was missing for one participant in the no “significant” disease activity group. [b]Baseline Timed 25 Foot Walk (T25-FW) was missing for two participants in the no “significant” disease activity group. [c]One participant in the continue group and 12 in the discontinue group. NA: not applicable.

© 2024 Coerver et al. *JAMA Neurology.*

eTable 7. Linear Mixed-Effects Model Regarding Absolute Serum NfL Level in the Continue and Discontinue Group

	**Beta (95% CI)**	**p value**
Randomization group[a]	1.23 (-1.45-3.92)	0.37
Time point	0.09 (-0.02-0.20)	0.12

Estimates from linear mixed-effects model. Outcome of the model is absolute serum NfL level with participant as random effect and randomization group (continue vs discontinue group) and time point as fixed effects. [a]Beta coefficient of discontinue group is given compared to continue group.

© 2024 Coerver et al. *JAMA Neurology.*

eTable 8. Linear Mixed-Effects Model Regarding Absolute Serum NfL Level in "Significant" Disease Activity at Participant Level

	**Beta (95% CI)**	**p value**
Randomization group[a]	-0.16 (-2.85-2.54)	0.91
"Significant" disease activity	6.92 (2.55-11.29)	0.003
Time point	0.08 (-0.03-0.19)	0.17

Estimates from linear mixed-effects model. Outcome of the model is absolute serum NfL level with participant as random effect and randomization group (continue vs discontinue group), "significant" disease activity and time point as fixed effects. "Significant" disease activity was defined at participant level. [a]Beta coefficient of discontinue group is given compared to continue group.

© 2024 Coerver et al. *JAMA Neurology.*

eTable 9. Linear Mixed-Effects Model Regarding Absolute Serum NfL Level in "Significant" Disease Activity at Visit Level

	**Beta (95% CI)**	**p value**
Randomization group[a]	0.3 (-2.24-2.85)	0.82
"Significant" disease activity	9.11 (5.0-13.34)	<0.001
Time point	0.1 (-0.01-0.21)	0.08
Estimates from linear mixed-effects model. Outcome of the model is absolute serum NfL level with participant as random effect and randomization group (continue vs discontinue group), "significant" disease activity and time point as fixed effects. "Significant" disease activity was defined at visit level, including a 3-month window before and after. [a]Beta coefficient of discontinue group is given compared to continue group.		

© 2024 Coerver et al. *JAMA Neurology.*

eTable 10. Linear Mixed-Effects Model Regarding Absolute Serum NfL Level in "Any" MRI Activity at Participant Level

	**Beta (95% CI)**	**p value**
Randomization group[a]	0.14 (-2.61-2.89)	0.92
"Any" MRI activity	4.66 (0.84-8.48)	0.02
Time point	0.08 (-0.04-0.19)	0.18

Estimates from linear mixed-effects model. Outcome of the model is absolute serum NfL level with participant as random effect and randomization group (continue vs discontinue group), "any" MRI activity and time point as fixed effects. "Any" MRI activity was defined at participant level. [a]Beta coefficient of discontinue group is given compared to continue group.

© 2024 Coerver et al. *JAMA Neurology.*

eTable 11. Linear Mixed-Effects Model Regarding Absolute Serum NfL Level in "Any" MRI Activity Disease Activity at Visit Level

	**Beta (95% CI)**	**p value**
Randomization group[a]	0.42 (-2.15-3.00)	0.75
"Any" MRI activity	7.22 (3.42-11.10)	<0.001
Time point	0.10 (-0.01-0.21)	0.080

Estimates from linear mixed-effects model. Outcome of the model is absolute serum NfL level with participant as random effect and randomization group (continue vs discontinue group), "any" MRI activity and time point as fixed effects. "Any" MRI activity was defined at visit level, including a 3-month window before and after.
[a]Beta coefficient of discontinue group is given compared to continue group.

© 2024 Coerver et al. *JAMA Neurology.*

eTable 12. Linear Mixed-Effects Model Regarding Absolute Serum GFAP Level in the Continue and Discontinue Group

	**Beta (95% CI)**	**p value**
Randomization group[a]	-0.75 (-16.79-15.30)	0.93
Time point	0.11 (-0.10-0.32)	0.32

Estimates from linear mixed-effects model. Outcome of the model is absolute serum GFAP level with participant as random effect and randomization group (continue vs discontinue group) and time point as fixed effects. [a]Beta coefficient of discontinue group is given compared to continue group.

© 2024 Coerver et al. *JAMA Neurology.*

eTable 13. Linear Mixed-Effects Model Regarding Absolute Serum GFAP Level in "Significant" Disease Activity at Participant Level

	**Beta (95% CI)**	**p value**
Randomization group[a]	0.18 (-16.70-17.05)	0.98
"Significant" disease activity	-5.12 (-34.25-24.00)	0.73
Time point	0.11 (-0.10-0.32)	0.32

Estimates from linear mixed-effects model. Outcome of the model is absolute serum GFAP level with participant as random effect and randomization group (continue vs discontinue group), "significant" disease activity and time point as fixed effects. "Significant" disease activity was defined at participant level. [a]Beta coefficient of discontinue group is given compared to continue group.

© 2024 Coerver et al. *JAMA Neurology.*

eTable 14. Linear Mixed-Effects Model Regarding Absolute Serum GFAP Level in "Significant" Disease Activity at Visit Level

	**Beta (95% CI)**	**p value**
Randomization groupa	-1.36 (-18.30-15.60)	0.88
"Any" MRI activity	2.74 (-21.83-27.29)	0.83
Time point	0.11 (-0.10-0.32)	0.33

Estimates from linear mixed-effects model. Outcome of the model is absolute serum GFAP level with participant as random effect and randomization group (continue vs discontinue group), "significant" disease activity and time point as fixed effects "Significant" disease activity was defined at visit level, including a 3-month window before and after. [a]Beta coefficient of discontinue group is given compared to continue group.

© 2024 Coerver et al. *JAMA Neurology.*

eTable 15. Linear Mixed-Effects Model Regarding Absolute Serum GFAP Level in “Any” MRI Activity at Participant Level

	**Beta (95% CI)**	**p value**
Randomization group[a]	-0.87 (-16.94-15.21)	0.92
"Significant" disease activity	1.20 (-7.8-10.22)	0.79
Time point	0.11 (-0.10-0.32)	0.31

Estimates from linear mixed-effects model. Outcome of the model is absolute serum GFAP level with participant as random effect and randomization group (continue vs discontinue group), “any” MRI activity and time point as fixed effects. "Any" MRI activity was defined at participant level. [*]Beta coefficient of discontinue group is given compared to continue group. [a]Beta coefficient of discontinue group is given compared to continue group.

© 2024 Coerver et al. *JAMA Neurology.*

eTable 16. Linear Mixed-Effects Model Regarding Absolute Serum GFAP Level in "Any" MRI Activity Disease Activity Visit Level

	**Beta (95% CI)**	**p value**
Randomization group[a]	-0.58 (-16.65-15.49)	0.94
"Any" MRI activity	-1.52 (-9.50-6.46)	0.71
Time point	0.10 (-0.11-0.32)	0.34

Estimates from linear mixed-effects model. Outcome of the model is absolute serum GFAP level with participant as random effect and randomization group (continue vs discontinue group), "any" MRI activity and time point as fixed effects. "Any" MRI activity was defined at visit level, including a 3-month window before and after. [a]Beta coefficient of discontinue group is given compared to continue group.

© 2024 Coerver et al. *JAMA Neurology.*

eTable 17. Median (IQR) Expanded Disability Status Scale (EDSS) Change by Group

Period	Continuation (n=44)	Discontinuation (n=45)	p value
Baseline	3.5 (2.0-4.0)	2.5 (1.5-4.0)	0.40
EoS visit	3.0 (2.0-4.0)	2.5 (2.0-3.5)	0.54
Change	0.0 (-0.50-0.50)	0.0 (0.0-0.50)	0.95
p value from Mann-Whitney test. Latest available visit was used when an end of study (EoS) visit was not available.			

© 2024 Coerver et al. *JAMA Neurology.*

eTable 18. Proportions of Significant Confirmed Disability Progression

	**Continuation (n=41)**	**Discontinuation (n=43)**	**p value**
Confirmed disability progression	7	7	0.92
No confirmed disability progression	34	36	
	**“Significant” disease activity (n=8)**	**No “significant” disease activity (n=76)**	**p value**
Confirmed disability progression	1	13	0.74
No confirmed disability progression	7	63	
	**“Any” MRI activity (n=12)**	**No “any” MRI activity (n=72)**	**p value**
Confirmed disability progression	2	12	1.00
No confirmed disability progression	10	60	

p value from χ^2 test. Significant confirmed disability progression was defined as an increase of ≥1.0 point from the baseline EDSS score if the baseline score was ≤5.5 or an increase of ≥0.5 points if the baseline score was >5.5, sustained for at least 24 weeks. Only participants with a follow-up of minimal 24 weeks were included.

© 2024 Coerver et al. *JAMA Neurology.*

eTable 19. Mean Percentage (SD) Change of NfL and GFAP Levels of Participants With Confirmed Disability Progression

	**Confirmed disability progression (n=14)**	**p value**
NfL change	1.36% (SD 31.76)	0.91
GFAP change	0.72% (SD 9.28)	0.83
	**Prior confirmed disability progression (n=14)**	**p value**
NfL change	28.20% (SD 108.99)	0.07
GFAP change	-2.45% (SD 11.98)	0.58
p value from one sample T-test. Percentage change was calculated form baseline till confirmed disability progression as well as the percentage change from baseline till the visit prior confirmed disability progression.		

© 2024 Coerver et al. *JAMA Neurology.*

eTable 20. Mean (SD) Symbol Digit Modalities Test (SDMT) Change by Group

Period	Continuation (n=44)	Discontinuation (n=45)	p value
Baseline	51.6 (11.4)	51.7 (13.3)	0.98
EoS visit	54.4 (15.0)	56.6 (14.1)	0.51
Change	3.0 (8.7)	5.2 (9.7)	0.29
p value from two-sample t-test. Latest available visit was used when an end of study visit (EoS) was not available.			

© 2024 Coerver et al. *JAMA Neurology.*

eTable 21. Median (IQR) Timed 25-Foot Walk (T25-FW) Change by Group

Period	Continuation (n=44)	Discontinuation (n=45)	p value
Baseline	5.12 (4.44-6.0)	5.05 (4.51-5.94)	0.80
EoS visit	5.03 (4.38-5.94)	4.78 (4.43-5.85)	0.48
Change	-0.01 (-0.29-0.30)	0.10 (-0.34-0.48)	0.37
p value from Mann-Whitney test. Latest available visit was used when an end of study (EoS) visit was not available.			

© 2024 Coerver et al. *JAMA Neurology.*

eTable 22. Median (IQR) 9-Hole Peg Test (9-HPT) Change by Group

Period	Continuation (n=44)	Discontinuation (n=45)	p value
Baseline	21.98 (19.54-25.46)	21.37 (19.29-24.85)	0.69
EoS visit	20.35 (18.25-24.52)	21.33 (17.48-23.42)	0.76
Change	-0.95 (-2.40- -0.13)	-0.85 (-2.29-0.31)	0.50
p value from Mann-Whitney test. Latest available visit was used when en end of study (EoS) visit was not available.			

© 2024 Coerver et al. *JAMA Neurology.*

eTable 23. Median (IQR) MS Impact Scales (MSIS-29) by Group

Scale	Period	Continuation (n=44)	Discontinuation (n=45)	p value
Physical impact	Baseline	32.0 (25.0-45.0)	27.5 (22.5-37.3)	0.20
	EoS visit	33.0 (23.0-45.0)	28.0 (23.0-41.3)	0.50
	Change	0.0 (-4.0-4.0)	0.5 (-1.0-3.3)	0.32
Psychological impact	Baseline	14.0 (11.0-18.0)	13.0 (10.0-15.0)	0.16
	EoS visit	14.0 (11.0-17.0)	12.0 (10.8-16.0)	0.55
	Change	0.0 (-2.0-1.0)	0.0 (-1.0-2.3)	0.31

p value from Mann-Whitney test. Latest available visit was used when an end of study (EoS) visit was not available. The physical impact ranges from 20-100 and the psychological impact ranges from 9-45, where higher score indicates worse outcome. 33/44 in the continue and 36/45 in the discontinue group filled in the questionnaire during follow-up.

© 2024 Coerver et al. *JAMA Neurology.*

eTable 24. Significant Change in MS Impact Scales (MSIS-29) by Group

Scale		Continuation (n=44)	Discontinuation (n=45)	p value
Physical Impact	Significant change, n (%)	8 (24.2)	8 (22.2)	0.84
	No significant change, n (%)	25 (75.8)	28 (77.8)	
Psychological impact	Significant change, n (%)	3 (9.1)	1 (2.8)	0.26
	No significant change, n (%)	30 (90.9)	35 (97.2)	

p value from χ^2 test. Latest available visit was used when an end of study (EoS) visit was not available. Significant change defined as a change of 7.5 points or greater between baseline and EoS visit.[2] 33/44 in the continue and 36/45 discontinue group filled in the questionnaire during follow-up.

© 2024 Coerver et al. *JAMA Neurology.*

eTable 25. Median (IQR) Checklist Individual Strength (CIS20R) by Group

Domain	Period	Continuation (n=44)	Discontinuation (n=45)	p value
Total	Baseline	62.0 (36.0-86.0)	55.0 (39.5-73.3)	0.49
	EoS visit	64.0 (45.0-87.0)	55.0 (37.0-82.0)	0.27
	Change	1.0 (-5.0-12.0)	0.0 (-8.8-11.0)	0.61
Fatique	Baseline	32.0 (16.0-40.0)	24.0 (16.0-29.5)	0.39
	EoS visit	31.0 (17.0-37.0)	21.5 (14.8-37.0)	0.32
	Change	0.0 (-5.0-7.0)	0.0 (-4.0-6.3)	0.86
Concentration	Baseline	13.0 (9.0-20.0)	13.0 (8.3-21.0)	0.79
	EoS visit	13.0 (10.0-23.0)	13.5 (8.8-17.0)	0.48
	Change	0.0 (-5.0-3.0)	0.0 (-4.0-2.5)	0.80
Motivation	Baseline	11.0 (7.0-16.0)	9.0 (6.0-14.5)	0.48
	EoS visit	11.0 (8.0-17.0)	10.0 (7.0-15.3)	0.21
	Change	1.0 (-1.0-2.0)	1.0 (-2.0-3.0)	0.70
Activity	Baseline	10.0 (4.0-14.0)	7.0 (5.8-11.3)	0.77
	EoS visit	10.0 (6.0-15.0)	6.5 (4.8-13.3)	0.18
	Change	1.0 (0.0-2.0)	0.0 (-2.0-2.0)	0.16

p value from Mann-Whitney test. Latest available visit was used when an end of study (EoS) was not available. The total score is the sum of the different statements, ranging from 20-140, where higher score indicates worse outcome. 33/44 in the continue and 36/45 in the discontinue group filled in the questionnaire during follow-up.

© 2024 Coerver et al. *JAMA Neurology.*

eTable 26. Significant Change in Fatigue Severity in Checklist Individual Strength (CIS20R)

	**Continuation (n=44)**	**Discontinuation (n=45)**	**p value**
Significant change, n (%)	22 (66.7%)	25 (69.4%)	0.81
No significant change, n (%)	11 (33.3%)	11 (30.6%)	

p value from χ^2 test. Latest available visit was used when an end of study (EoS) visit was not available. Significant change defined as a change of 8.0 points or greater between baseline and EoS visit.[3] 33/44 in the continue and 36/45 in the discontinue group filled in the questionnaire during follow-up.

© 2024 Coerver et al. *JAMA Neurology.*

eTable 27. Median (IQR) Short Form Health Survey (SF-36) by Group

Scale	Period	Continuation (n=44)	Discontinuation (n=45)	p value
Physical component score	Baseline	43.90 (36.47-52.14)	47.12 (42.65-50.78)	0.50
	EoS visit	43.06 (36.88-50.14)	45.10 (40.48-51.38)	0.42
	Change	-0.45 (-3.72-1.11)	-0.42 (-4.25-1.45)	0.93
Mental component score	Baseline	44.96 (40.16-48.83)	48.30 (43.56-51.52)	0.10
	EoS visit	46.05 (39.16-48.83)	49.14 (42.65-51.60)	0.11
	Change	0.84 (-4.05-3.27)	0.85 (-3.03-1.86)	0.79

p value from Mann-Whitney test. Latest available visit was used when an end of study (EoS) visit was not available. Physical and mental component score each ranges from 0-100, where higher score indicates better outcome. 34/44 in the continue and 33/45 in the discontinue group filled in the questionnaire during follow-up.

© 2024 Coerver et al. *JAMA Neurology.*

eTable 28. Significant Change in Short Form Health Survey (SF-36) by Group

**Scale**		**Continuation (n=44)**	**Discontinuation (n=45)**	**p value**
Physical component score	Significant change, n (%)	7 (25.0%)	11 (33.3%)	0.48
	No significant change, n (%)	21 (75.0%)	22 (66.7%)	
Mental component score	Significant change, n (%)	4 (14.3%)	5 (15.2%)	0.92
	No significant change, n (%)	24 (85.7%)	28 (84.8%)	

p value from χ^2 test. Latest available visit was used when an end of study (EoS) visit was not available. Significant change defined as a change of 5.0 points or greater between baseline and EoS visit.[4] 28/44 in the continue and 33/45 in the discontinue group filled in the questionnaire during follow-up.

© 2024 Coerver et al. *JAMA Neurology.*

eTable 29. Treatment Satisfaction by Group

Period	Continuation (n=44)	Discontinuation (n=45)	p value
Baseline, n (%)	27 (79.4%)	33 (89.2%)	0.27
EoS visit, n (%)	27 (79.4%)	31 (83.8%)	0.63

p value from χ^2 test. Latest available visit was used when an end of study (EoS) visit was not available. Participants also responded to the question “How satisfied are you with your present DMT or lack of DMT?”. Proportion of participants answering "satisfied", "very satisfied" or "extremely satisfied" on a 7-point Likert scale. 34/44 in the continue and 37/45 in the discontinue group filled in the questionnaire during follow-up.

© 2024 Coerver et al. *JAMA Neurology.*

eReferences.

1. van der Voort LF, Gilli F, Bertolotto A, et al. Clinical Effect of Neutralizing Antibodies to Interferon Beta That Persist Long After Cessation of Therapy for Multiple Sclerosis. Arch Neurol-Chicago 2010;67(4):402-407.
2. Phillips GA, Wyrwich KW, Guo S, et al. Responder definition of the Multiple Sclerosis Impact Scale physical impact subscale for patients with physical worsening. Mult Scler 2014;20(13):1753-60.
3. Beckerman H, Blikman LJ, Heine M, et al. The effectiveness of aerobic training, cognitive behavioural therapy, and energy conservation management in treating MS-related fatigue: the design of the TREFAMS-ACE programme. Trials 2013;14:250.
4. Norman GR, Sloan JA, Wyrwich KW. Interpretation of changes in health-related quality of life - The remarkable universality of half a standard deviation. Med Care 2003;41(5):582-592.
5. Goldman MD, LaRocca NG, Rudick RA, et al. Evaluation of multiple sclerosis disability outcome measures using pooled clinical trial data. Neurology 2019;93(21):e1921-e1931.

© 2024 Coerver et al. *JAMA Neurology.*
