# Supplementary material for: Discontinuation of First-Line Disease-Modifying Therapy in Patients With Stable Multiple Sclerosis: The DOT-MS Randomized Clinical Trial
Source: JAMA Neurol. 2024 Dec 9;82(2):123–31. doi: 10.1001/jamaneurol.2024.4164 (PMC11811793; doi:10.1001/jamaneurol.2024.4164)
Supplement: Supplement 3. — Data Sharing Statement [file jamaneurol-e244164-s003.pdf]

Data Sharing Statement

Coerver. Discontinuation of First-Line Disease-Modifying Therapy in Patients With Stable Multiple Sclerosis. *JAMA Neurol*. Published December 09, 2024.
doi:10.1001/jamaneurol.2024.4164

Data
Additional Information: ClinicalTrials.gov, https://clinicaltrials.gov/study/NCT04260711, NCT04260711.
Data available: Yes
Data types: Participant data with identifiers
How to access data: Corresponding author: E.M.M. Strijbis, MD PhD Amsterdam UMC, location VUmc MS Center Amsterdam T: +3120 4441966 E: e.strijbis@amsterdamumc.nl
When available: With publication

Supporting Documents
Document types: None

Additional Information
Who can access the data: DOT-MS clinical trial data and documents can be requested from the corresponding author, immediately following publication and with no end date, by any qualified researchers who engage in rigorous independent scientific research.
Types of analyses: for scientific research
Mechanisms of data availability: with investigator support, after approval of proposal and with a signed data access agreement/data sharing agreement
